# Dropping the baton: Cognitive biases in emergency physicians

**DOI:** 10.1371/journal.pone.0316361

**Published:** 2025-01-02

**Authors:** Mingwei Ng, Evelyn Wong, Guek Gwee Sim, Pek Jen Heng, Gareth Terry, Foo Yang Yann

**Affiliations:** 1 Department of Emergency Medicine, Singapore General Hospital, Singapore, Singapore; 2 Accident and Emergency Department, Changi General Hospital, Singapore, Singapore; 3 Department of Emergency Medicine, Sengkang General Hospital, Singapore, Singapore; 4 School of Psychology, Massey University, Auckland, New Zealand; 5 Academic Medicine Education Institute, Duke-NUS Medical School, Singapore, Singapore; Universiti Sains Malaysia - Kampus Kesihatan, MALAYSIA

## Abstract

**Introduction:**

Clinical medicine is becoming more complex and increasingly requires a team-based approach to deliver healthcare needs. This dispersion of cognitive reasoning across individuals, teams and systems (termed “distributed cognition”) means that our understanding of cognitive biases and errors must expand beyond traditional “in-the-head” individual mental models and focus on a broader “out-in-the-world” context instead. To our knowledge, no qualitative studies thus far have examined cognitive biases in clinical settings from a team-based sociocultural perspective. Our study therefore seeks to explore how cognitive biases and errors among emergency physicians (EPs) arise due to sociocultural influences and lapses in team cognition.

**Methodology:**

Our study team comprised four EPs of different seniorities from three different institutions and local and international academics who provided qualitative methodological guidance. We adopted a constructivist paradigm and employed a reflexive thematic analysis approach which acknowledged our researcher reflexivity. We conducted seven focus group discussions with 25 EPs who were purposively sampled for maximum variation. Our research question was: How do sociocultural factors lead to cognitive biases and medical errors among EPs?

**Results:**

Our themes coalesce around sociocultural pressures related to team psychology. In theme one, the EP is compelled by sociocultural pressures to blindly trust colleagues. In the second, the EP is obliged by cultural norms to be “nice” and neatly summarise cases into illness scripts during handovers. In the last, the EP is under immense pressure to follow conventional wisdom, comply with clinical protocols and not challenge inpatient specialists.

**Conclusion:**

Cognitive biases and errors in clinical decision-making can arise due to lapses in distributed team cognition. Although this study focuses on emergency medicine, these pitfalls in team-based cognition are relevant across the entire continuum of care and across all specialties of medicine. The hyperacute nature of emergency medicine merely exacerbates and condenses these into a compressed timeframe. Indeed, similar relays are run every day in every discipline of medicine, with the same unified goal of doing the best for our patients while not committing cognitive errors and dropping the baton.

## Introduction

Cognitive biases and errors have long been recognised as serious threats to patient safety [1]. Despite decades of research, cognitive errors remain wicked problems with no clear solution in sight [2, 3]. Focus thus far has mainly concentrated on exploring contemporary theories of individual clinical reasoning (such as dual process theory [4]), cataloguing the various types of flaws in individual mental models (such as anchoring, confirmation bias and premature diagnostic closure [5]), and understanding the predisposing factors leading to errors (such as fatigue, sleep deprivation [6] and extended-duration shifts [7]).

However, clinical medicine is becoming more complex. As healthcare needs are increasingly delivered by teams rather than individuals [8], cognitive biases and errors may arise not only from pitfalls in individual thinking but from lapses during interactions within healthcare teams as well. While concepts such as “distributed cognition” [9] which provide an ‘out-in-the-world’ perspective of clinical reasoning have become more relevant [10], these still largely remain as social cognitive theories illustrated by hypothetical examples rather than real-world, “flesh-and-blood” experiences [9]. To our knowledge, no qualitative studies thus far have examined cognitive biases in clinical settings from a broader ‘out-in-the-world’ perspective [11]. Our study therefore seeks to explore how cognitive biases and errors among emergency physicians (EPs) may arise due to sociocultural influences and lapses in team cognition.

## Methods

### Study design

Our qualitative study is informed by a constructivist paradigm which acknowledges the importance of researcher reflexivity. Given our research beliefs, we chose a reflexive thematic analysis [12] approach as our data analysis method. Please see **S1 File** for our reflexivity statement.

Participant recruitment was conducted from 5 February 2023 to 7 August 2023. We invited EPs from three different institutions to participate in focus group discussions to share their perspectives and experiences. We adopted focused group discussions as our primary method of data collection as this would encourage greater participant reflection on their own shared experiences and stimulate richer and more robust conversation as participants debate or build on each other’s ideas.

Participants from the same institution and of similar seniority were placed in homogeneous groups to ensure familiarity among the participants as we recognised that cognitive biases and errors are potentially sensitive issues to discuss. While the choice of focused group discussions could have introduced biases by promoting groupthink or pressuring participants to conform to social desirability, this homogeneity facilitated open discussion and encouraged participants to challenge each other freely, reducing the influence of hierarchal differences.

### Study setting

We conducted our study at three affiliated but separate institutions. Public hospitals are structured into three major healthcare clusters in Singapore–this study was conducted in the three emergency departments (EDs) of the largest cluster, with an overall annual attendance of nearly 369,000 visits.

### Study participants

Our study inclusion criteria included accredited emergency medicine (EM) specialists from any of the three institutions. Our participants were purposively sampled for maximum variation and therefore span a range of seniority: newly minted specialists, residency program directors and core faculty and former and current head-of-departments. Please see **S2 File** for more information on participant characteristics.

### Data collection

We used a semi-structured research topic guide for the focus group discussions, which was modified iteratively in response to emerging study findings. This iterative process allowed us to probe into areas that were initially unanticipated–such as the relevance of team dynamics to cognitive biases, which had not surfaced in our literature review–and generate a richer discussion. Our research question was: Based on the perceptions and experiences of EPs, how do sociocultural factors lead to cognitive biases and errors among EPs?

Focus group discussions comprising three to five participants each and lasting approximately 90 minutes each were conducted over videoconferencing from 28 February 2023 to 29 September 2023. All participants were anonymised and identifiable only by their unique participant number. Each discussion was facilitated by a moderator from the same institution and co-moderator from a different institution. Participant names were not revealed to the moderators. Audio-recordings were transcribed verbatim. We ceased data collection after seven focus groups (total 25 participants) based on the richness of the discussions and dense sampling specificity (Malterud *et al*., 2016) [13].

### Data analysis

We read through the transcripts repeatedly to develop familiarity with the dataset and took casual observational notes about the content. All four EPs coded independently to add the value of different persspectives to the analysis–first at the descriptive (semantic) level and subsequently at the interpretative (latent) level.

We collectively constructed candidate themes from our different sets of codes. Using thematic maps developed in the Miro visual collaboration platform (*www.miro.com*), we reviewed each candidate themes’ utility in telling a compelling story. Analysis was sensitised by the transtheoretical model of clinical reasoning proposed by Daniel *et al*. [14], which posits that information processing occurs across multiple teams and systems and broad social cognitive theories like distributed cognition are necessary to capture this complex interplay between individuals, other people and their surroundings during clinical reasoning [15]. We then finalised these into three themes which revolve around the central narrative that medicine relies on team cognition.

### Ethical considerations

This study was approved by SingHealth Centralised Institutional Review Board (CIRB Reference Number 2022/2684). Funding was received from the SingHealth Duke-NUS Academic Medicine Education Institute (AMEI) Grant (EING 2302). The funders had no role in study design, data collection and analysis, decision to publish or preparation of the manuscript. Written informed consent was obtained from all participants.

## Results

**Fig 1:**
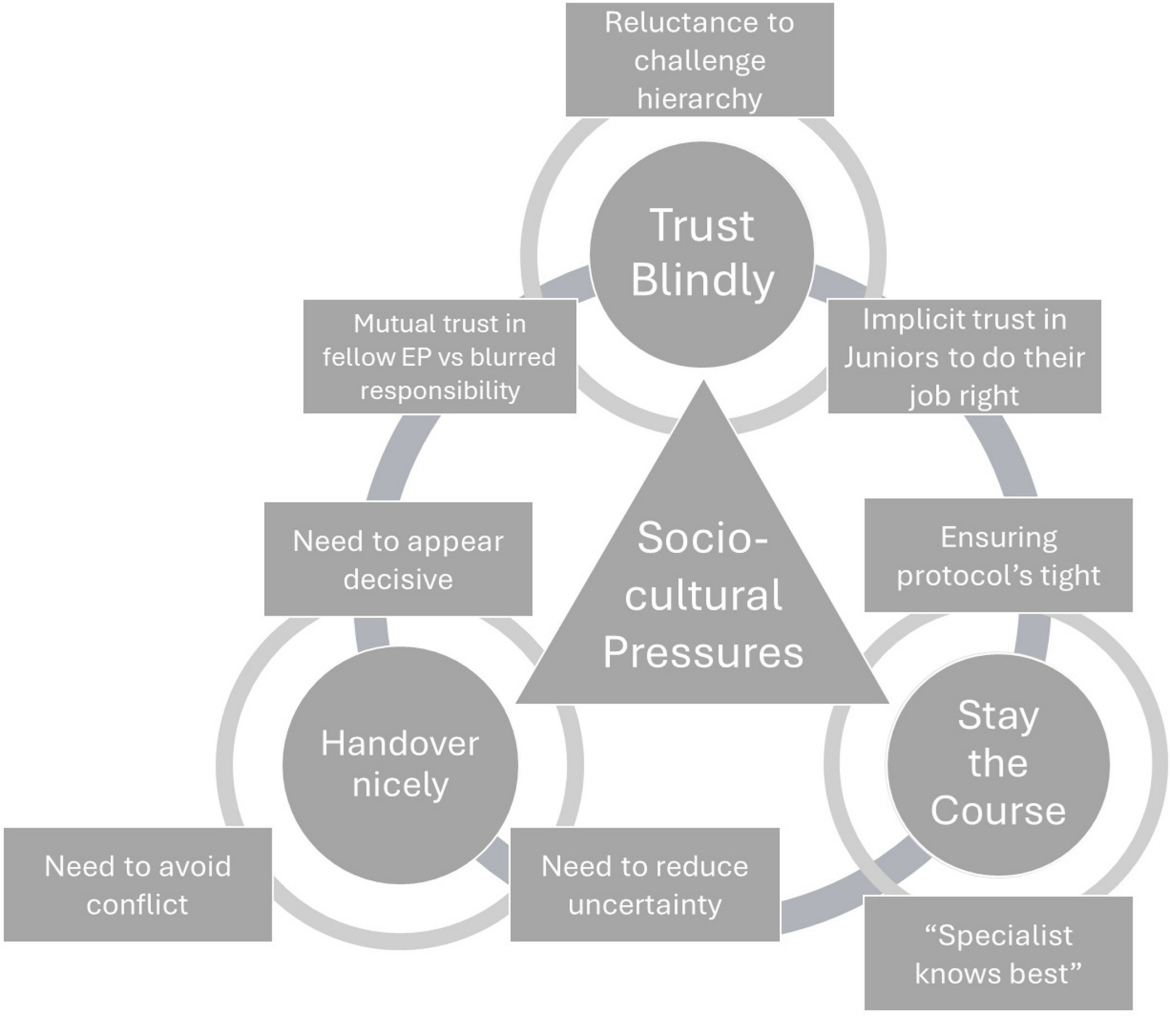
Diagrammatic representation of themes.

Our themes coalesce around sociocultural pressures related to team psychology. In theme one, the EP is compelled by sociocultural pressures to blindly trust one’s colleagues. In the second, the EP is obliged by cultural norms to be nice and neatly summarise cases into illness scripts during handovers. In the last, the EP is under immense pressure to follow conventional wisdom, comply to clinical protocols and not challenge the authority of specialists. These themes have been represented diagrammatically in **Fig 1**.

### Theme 1: The sociocultural pressure to trust blindly

Some EPs place unquestioning faith in their colleagues when they receive information–setting themselves up for potential failure when they inherit their colleagues’ biases and errors.

Healthcare workers increasingly work in teams rather than individual silos, with different team members performing different tasks (triage, data gathering, data analysis) that are collectively required to arrive at a diagnosis and manage the patient. Several participants argued that this interprofessional sense-making in teams conferred protection from cognitive biases:

That’s why we work in teams to reduce biases. That’s why we talk, we discuss with the team. Everybody has different views and we add our information together to reduce these errors. Medicine is moving towards team-based, instead of solo-physician-led, to combat cognitive issues. (P22, Study 6, Senior consultant)

While working in teams should theoretically provide safety in numbers and more accountability, participants recognise that they may not scrutinise the accuracy of information received closely enough.

This may be because workload and time pressures make re-evaluating data from scratch for oneself difficult. However, this unquestioning culture may also be attributed to an implicit trust in colleagues to do their job right and not need a senior to *“retake the entire history and redo the physical exam”* (P3, Study 1, Senior consultant).

When the shift is really busy and I don’t have enough time to allocate to each patient under my care, I trust the junior doctors more… And sometimes we become anchored, especially if we don’t have time to eyeball the patient and see them ourselves. (P8, Study 2, Junior consultant)

But it is not just the accuracy of the information but the adequacy that matters. Participants highlighted that omissions can be just as misleading, because failure of a colleague to report pivotal data suggests the absence of critical information. The expectation is that any data of relevance would have been searched for and presented for consideration on a platter–even if this is premised on the assumption that the junior appreciates what is of significance to the EP. One participant spoke of this in emotionally intense framing:

If later on you find out there was some very crucial information that was documented or on the system that was not highlighted verbally to you, it makes you feel very betrayed. [laughs] (P14, Study 4, Junior consultant)

Another participant concurred that being presented with a framed perspective amounts to being “*set-up*” given that EPs would trust their colleagues to report objectively and completely:

If the patient’s condition is already heuristically framed in a way that leads you towards a cognitive error, then of course it’s a set-up… (P3, Study 1, Senior consultant)

However, this unquestioning blind trust works both ways. A junior’s reluctance to challenge a senior colleague’s plans stems from trust that the latter knows what they are doing. Questioning a senior who must be correct even when things do not quite seem to add up would only expose one’s ignorance or worse, cause the senior to take offense in the local conservative Asian culture. For instance:

Feedback from seniors is common but questioning or feedback from juniors is very uncommon in our culture. … No junior will dare tell you, you are not very good or clear. (P14, Study 4, Junior consultant)

Yet receiving feedback from junior colleagues is crucial for the growth of any EP regardless of seniority. Some participants suggested overcoming this challenge by being deliberately candid about uncertainties or knowledge gaps and actively soliciting the opinions of juniors. This potentially flattens the power hierarchy and demonstrates openness to consultative discussion and feedback. For instance, P2 commented:

I try to have a discussion, rather than “I tell you to do this”. I ask for their opinions and bounce things off them … Even though they’re not my peer, they saw the patient first-hand. They may have something valuable to add, something I wouldn’t have realised. (P2, Study 1, Junior consultant)

Nothing, though, exemplifies this concept of implicit trust more than mutual trust among EPs–EPs expect their fellow EPs to be competent. This might lead an EP to disregard a screaming, unruly patient that another EP has assessed and labelled as uncooperative or “*doing some psychiatric thing*” (P13, Study 3, Senior consultant):

Patients who irritate you make you even more likely to ignore them, once you feel you or someone else has done due diligence on your behalf… (P16, Study 4, Junior consultant)

P16 cited a patient with post-ictal drowsiness who started shouting but was ignored by three seniors for an hour because “*one of us had seen him and thought he didn’t look that bad*”. This patient was deemed, in P16’s words, “*weird*” because he did not make sense (*“Don’t touch me*, *I have pain*!*” ‘Where’s your pain*?*’ “I don’t have pain*, *I just need to shout*!*”*) until he was eventually found to have had a seizure complicated by a cervical fracture leading to urinary retention and delirium.

Does a duty of care fall on an EP who witnesses a patient screaming but chooses to avoid contact because another EP has assumed responsibility? Such blurred lines of responsibility can lead to awkward situations where multiple EPs witness but deliberately ignore the emotional intensity and become bystanders.

### Theme 2: The sociocultural pressure to handover “nicely”

EPs may unwittingly propagate and handover biases while handing over care. Participants highlighted a perceived intrinsic need to be “*nice*” by simplifying handovers. This involves synthesizing and consolidating as much information as possible so that cases are neatly summarised during handover.

Because we are very busy, I just wanted to get everything done on time, before I handover, because it’s not nice otherwise. (P18, Study 5, Junior consultant)

This “*niceness*” could be borne out of sheer consideration–fewer cases to handover certainly means less cognitive load, especially on a busy shift. Another reason could be a sense of shame–shame at shirking one’s responsibility by signing out and burdening one’s colleague to takeover unfinished work. Yet another contributing factor is the decisiveness that EPs pride themselves on (and have come to demand of each other) during handovers. This leads to a dangerous self-perpetuating cycle where EPs not only fail to recognise the potential set-up for bias but also expect cases to be summarised with decisive plans when receiving handovers.

This instinctive need for decisiveness likely stems from the nature of this field which attracts particular character and personality types. P16 spoke to this:

It’s part of the character of being an EP. Part of the job description. If you are slow and take history like a two-hour geriatrician, you won’t be in emergency medicine already. [Laughs]. It’s just like surgery, if you can’t do appendicectomy in one hour, then how do you call yourself a specialist? (P16, Study 4, Junior Consultant)

EM training also further hardwires decisiveness–unfortunately, this hardwiring means that this decisiveness can become independent of the context and detailed information, even in the rare absence of time pressure:

When you learn to ride a bicycle in a certain way, even when the situation allows it, you are unlikely to change the way you ride a bicycle. If you’re trained to think fast with limited data, you will still fall back on how you were trained and how you were doing things all this while, even when you have a lot of time. (P3, Study 1, Senior consultant)

As P14 (Study 4, Junior consultant) described, EM is ultimately the art of “*weighing the odds*” and constantly making conscious decisions; whether to act on heuristics despite incomplete information or hold back and await more data at the expense of time and resources.

So since the puzzle is five-piece and we only have three pieces, let’s just make a call in the interest of balancing and weighing (the odds). So, we inadvertently commit a cognitive error. You would perhaps even say knowingly. (P2, Study 1, Junior consultant)

P4 highlighted fears that this focus on “*nice*” clean handovers in EM normalizes cognitive errors. We fail to acknowledge our biases and instead use external factors like time constraints and resource limitations as “*convenient excuses to accept sub-par quality of care*”–leading to a form of learned helplessness over time.

Therefore we easily make cognitive errors, thinking this is expected. We think this is normal so it becomes normalised. We think we can’t help but make it. (P4, Study 1, Junior consultant)

Another reason why handovers may potentiate team cognition errors stems from most EPs’ desire to avoid conflict. Handovers represent high-stress intersections when different colleagues, who inevitably have differing clinical thresholds, interact and opposing opinions clash. Worries that the receiving EP may express surprise or even masked displeasure at one’s plans are a concern, especially when passing to a senior colleague and this increases as one approaches handover time. This anticipatory fear may prompt the EP to second-guess their incoming colleague, attempt to prematurely close the case to avoid handing over altogether, or defer critical decision-making until changeover to minimise conflict.

There’s also a little bit of expectation from others, especially whom I’m handing over to. Do you feel I am over-investigating? … Although, there’s no rudeness and unpleasantness about it, sometimes there can be some expression of surprise that a patient has not been disposed earlier–even if I feel I really need a little bit more information first. (P3, Study 1, Senior consultant)

This rush to make sense of and summarise the case prior to handover whilst constrained by limited available information creates the perfect hotbed for cognitive errors. Premature closure risks clouding the receiving team’s judgment: it dissuades them from searching for differentials and encourages them to fixate on earlier diagnoses made with incomplete information–sometimes even “*rejecting new (contrarian) data points that present later”* and *“choosing to cling on to the initial diagnosis*” as P4 (Study 1, Junior consultant) described.

When somebody tells you: “This is the diagnosis”, we anchor onto it. Sometimes it can be totally wrong but we would never think about other things because we are already down that line. (P13, Study 3, Senior consultant)

Ironically, a handover exists precisely because information is incomplete–any assumption that the information, provisional diagnoses and plans presented during a handover are infallible is therefore clearly fallacious.

The problem with summarizing cases neatly to achieve a “*nice*” handover becomes compounded when EPs feel the need to justify clinical decisions made by selling a key narrative during the handover. These narratives are often crafted together by cherry-picking certain details (and downplaying details that do not fit) to better match the classical illness scripts taught in medical school and reduce clinical uncertainty.

There’s a bit of a cultural thing going on, because if (you) are not able to make things fit in a way, then you have a problem after that. (P3, Study 1, Senior consultant)

While data points are objective, data interpretation ultimately remains very much subjective. Indeed, semantic qualifiers like ‘tearing chest pain’ or ‘worst headache of my life’ are important catchphrases during handover that would immediately prompt the receiving clinician to commit to a certain track. Yet these terms are artificial, binary and require the reporting EP to commit when reality is seldom so clean–as P3 asked, if a patient reports “*no chest pain but a little chest discomfort*, *maybe a 0*.*5 out of 10 –would you call that pain or no pain*?”

### Theme 3: The sociocultural pressure to stay the course

This theme moves beyond intra-team communication to inter-team interactions between EPs and specialists from other departments. It describes how EPs are compelled to adhere to clinical protocols, scopes of practice, and accept specialist opinion–essentially, to stay the course–because these are perceived as standard of care.

When our brain hits a certain load, we take shortcuts. We try to short-circuit the process. We try to fit things into protocols to simplify them so that we can move on to our next task. (P10, Study 3, Senior consultant)

Clinical protocols help EPs reduce cognitive load by providing a structured framework for making certain decisions like when to trigger specialist consultations. They can never replace clinical gestalt because they have specific caveats and cannot encompass all situations and every patient’s unique circumstances. Despite these limitations, some EPs may invariably regard protocols as infallible rules rather than guidance, because they represent institution-endorsed best practices. To ensure patient management remains watertight and defensible, some participants felt compelled to rigidly adhere to departmental protocols and seek specialist opinion regardless of appropriateness, since deviation or delay sets oneself up for inquiry and increased medicolegal culpability when outcomes go awry.

Because of all these time-sensitive protocols that we have, sometimes we are pressured—because if there’s a delay in activating I’ll get asked: “Why was there a delay?” (P12, Study 3, Senior consultant)

In most cases, the decision to activate the protocol sets in motion a cascade of actions such as calling specialists from other departments. The fast flurry of events worsens the diagnostic momentum to stay the course and follow through with the protocol–even if new information subsequently emerges that suggests the possibility of another condition. Applying clinical protocols inappropriately may also cause tunnel vision and lead one astray.

Sometimes you are very satisfied with a certain diagnostic test that confirms your suspicion of a diagnosis, and you just stop there. I had this patient with a large-vessel occlusion stroke seen on CT. But actually the CT multiphasic stroke protocol covers all the way down to the root of aorta, and we totally missed the dissection there. (P17, Study 5, Junior consultant)

Likewise, while overall responsibility of patient care in the ED ultimately rests with the EP, the EP oftentimes feels trapped to defer to specialist opinion, which is ultimately deemed standard of care. Unclear lines of responsibility–coupled with the widely-held perception that specialists are the subject matter experts and know best–leaves the EP intimidated with the uncomfortable dilemma of deciding whether to pursue the path of least resistance and follow specialist opinion despite being in silent disagreement, or to object but assume increased responsibility if wrong.

All this unclear responsibility contributes… The fact that this protocolised care happens because of time sensitivity will just make you make errors. You have to throw the dice. (P10, Study 3, Senior consultant)

One participant cited a patient with limb weakness suspicious for stroke who also complained of neck pain. The EP repeatedly expressed concerns that “*this is a very weird presentation for a stroke*” but the neurologist pressured the EP by offering thrombolysis regardless. The patient was eventually discovered to have had a spontaneous epidural hematoma worsened by thrombolysis days later.

The neurologist says to thrombolyse, right? But if something bad happens, the neurologist will say, “You are the doctor on the ground. You can still refuse.” Which is the thing: you get all the credit, while I get all the blame. (P13, Study 3, Senior consultant)

Specialists unsurprisingly tend to fixate on diagnoses within their domain knowledge–after all, as another participant phrased it: “*if you are a hammer right*, *everything will look like a nail*” (P3, Study 1, Senior consultant). This phenomenon becomes exacerbated in the ED because specialists are consulted mostly for unforgiving, high-stakes, time-sensitive cases like strokes and acute myocardial infarctions where decisions must be made fast even with limited information available.

## Discussion

The practice of clinical medicine is becoming increasingly complex, necessitating transformation into a team-based model for healthcare delivery. This has put into focus “distributed cognition” which is based upon interactions between individuals and requires a common mental model to be shared among members *(Green G*. *(2020)*. *Examining interprofessional team decision making through a distributed cognition lens (Unpublished doctoral thesis)*. *University of Calgary*, *Calgary*, *AB*. *http://hdl.handle.net/1880/111423)*. With multiple individuals, teams and systems involved and each assuming crucial roles, the concept of “distributed cognition” can be likened to a relay race where failure by any runner to perform or handover will ripple into downstream effects and result in team letdown.

Trust among team-mates is vital, yet blind trust can be dangerous. A relay team that blindly believes their trust in each other is sufficient to ensure a smooth transition and negate the need to practice baton handoffs is headed for catastrophe. Our findings in theme one of blind trust parallel observations made by Tschan *et al*. [16] who found that most simulation teams depend on a single person to consult the patient charts each time. Most shockingly, this critical role was often entrusted to whoever the confederate randomly picked to first receive the chart. This becomes a catastrophic, single point-of-failure because neglect by the “chart-holder” to report seemingly mundane but crucial details on the charts will lead other team members to assume this information was absent.

One participant spoke of how one might feel “*betrayed*” when trust in one’s teammates is misplaced, which another participant alluded to this as a “*set-up*”. Examples of this kind of “*betrayal*” are in fact not uncommon in daily clinical practice. For instance, a classic pitfall is that a junior may fail to elicit or neglect to report a significant history of splenectomy in a patient with an animal bite wound (Croskerry, 2003) [17]. This error of omission may have arisen because the physician may not necessarily appreciate the direct relevance to the patient’s current complaint.

The question to the EP might be framed even more innocuously–perhaps not as a request to proceed with a primary repair, but as a request for advice (“Could you advise me on what suture type to use please?”) or even supervision (“Could you guide me as I am not confident of suturing?”). Without being provided relevant critical details of the patient’s comorbidities, the EP may not be able to discern that this seemingly sensible request–which may appear difficult to decline at face value–is loaded with the wrong assumption that a primary repair is the correct course of action.

Being deliberately candid about knowledge gaps and adopting a consultative approach with juniors was suggested by one participant as a strategy to encourage colleagues to share any concerns or uncertainties. This tension between being authentic and exposing vulnerability vis-a-vis maintaining credibility as a faculty dovetails neatly with the concept of embracing “*intellectual candour*” as presented by Molloy and Bearman [18].

While our findings seem to suggest that blind trust in team-mates is culpable for cognitive biases, the “cognitive miser” theory offers another plausible competing explanation for unquestioning acceptance of a colleague’s assessment [19]. Just as an EP would hesitate to reevaluate a junior colleague’s work from scratch, so would an EP prefer to conserve time and energy instead of reassessing a case that another EP has seen. Interestingly, however, this “cognitive miser” theory would be at odds with our second theme where participants expressed the urge to do a “*nice*” handover to the incoming team and go beyond the due diligence required of them–even if this means an increased risk of cognitive error.

The second theme of the need to be “*nice*” during handovers is analogous to how a runner handing over the baton must slow down and time his speed to match his receiving teammate who is starting from rest. This intrinsic desire to be “*nice*” during ED handovers was also alluded to in a qualitative study done by Park *et al*.–in fact, one of the participants coined the mantra, “*it’s nice to be nice*” [20]–and in Lawrence *et al*.’s grounded theory study which described the general reluctance of EPs to handover as a way of expressing “*consideration*” [21].

Of course, one can stay “*nice*” by maintaining civil and collegial without being overtly helpful or seeking to please. These sociocultural expectations and pressures can make EPs struggle to reconcile with the notion that, sometimes, slowing down during handovers (or even ruthlessly leaving new cases to the incoming team to evaluate from scratch) may be safer than trying to be helpful. Such attitudinal changes would represent a paradigm shift given that strategies to optimise handover effectiveness have traditionally focused on other aspects of communication (such as use of standardised reporting templates [22], limiting interruptions [23] and harnessing technology [24]). Cheung *et al*. also highlighted how our innate desire to reduce the burden in handovers–while itself a valuable strategy for optimizing handover efficiency–must be balanced against prematurely forcing a diagnosis or disposition “*for the sake of expediency*” [25].

At other times, framing a patient’s condition “*nicely*” is seen as essential to ensure coherence when justifying a clinical decision or request. This behavior does not appear to be unique to our participants: Horwitz described how clinical uncertainty may be downplayed during handovers to *“‘prove’ the patient requires admission*” [26]. Power *et al*. noted this same phenomenon occurring during inter-specialty referrals to intensive care: non-intensive care consultants conceded to sometimes presenting information in different ways to try to *“sell”* an admission as “*there’s always a way of presenting stuff to improve the chances of getting the answer you want*” [27]. Power termed this practice of crafting narratives that align with the expectations of the receiving inpatient specialist to influence the latter’s decision or secure a request: “*game-playing*”. Ultimately, “game-playing” stems from the need of the EP to live up to the sociocultural expectation that EPs demonstrate competence and not make unwarranted requests. While it provides the EP a way out of justifying a somewhat equivocal clinical request and therefore allows the EP to “*save face*”, there are severe and detrimental consequences. Selective framing causes cognitive biases to creep into clinical decision-making by disrupting the open and unbiased sharing of information within the distributed cognition model. These “half-truths” also weaken the professional trust and relationship between specialties and foster an adversarial mentality.

Inter-specialty referrals–the crux of the third theme–can be compared to summoning a sprint coach to guide the relay team. Relays demand slightly different skillsets from an individual sprint. Inpatient specialists thrive best in controlled settings where information is replete and problem lists are clearly defined (the home stretch); while EPs thrive in making sense of chaos in the fog of war, negotiating the bends and constantly revising the most likely diagnosis even when time pressures inexorably challenge deliberate reflection. Rigid adherence to rules constrains the ability to adapt to dynamic evolving situations, yet reacting to every situation as if it is novel rapidly leads to cognitive overload [28].

The third theme is particularly relevant because of the tension between ED and inpatient teams: Klingberg *et al*. found that the highest rates of incivility occurred during communications between EPs and physicians from other departments [29]. Most literature on interdepartmental conflict between EPs and inpatient specialists traditionally revolve around other factors like how inpatient teams perceive the ED workup to be subpar [30, 31] while EPs prioritise stabilization, swift disposition and early inpatient transfer [32]. Campbell *et al*. presented a case with a similar theme as Participant 13 –a patient presented with lower limb weakness post-seizure which was attributed to pain. A referral was made to Neurology but neither the EP nor the neurologist considered an alternative diagnosis, leading to delayed discovery of a traumatic spinal epidural hematoma [5]. The potential for specialists to be biased by their narrow subspecialty domain expertise and neglect the bigger picture was also identified by Hashem [33] and Power [27], illustrating how EPs need to be mindful of these pitfalls and challenge the conventional wisdom that specialists provide the most definitive and comprehensive care.

## Limitations

Years ago, the role of the EP was primarily to stabilize critically ill patients and determine the appropriate disposition and acuity of care [34]. However, the global bed crunch has fundamentally altered this dynamic as admitted patients are increasingly boarded in the ED for prolonged periods. This places new demands on EPs to not only resuscitate, triage and determine disposition, but also manage and discharge patients with protocolised conditions in emergency observation units without inpatient specialist reassessment. Other emerging roles like telehealth, mobile integrated healthcare and home-based community services have also been entrusted on Eps [35]. As the scope and roles of the EP expand, so too would the nature of cognitive biases. This study, while illuminating how sociocultural factors influence EP cognitive biases, primarily focuses on decision-making within ED teams in a traditional ED-inpatient interface rather than interactions with the broader healthcare system such as allied health professionals and community care providers. It also does not fully address how the evolving scope of EP responsibilities lead to not just predominantly diagnostic but also management-type errors, representing a limitation in understanding the full spectrum of cognitive biases among EPs.

While determining reasons why cognitive biases can occur even amongst experienced EPs is valuable, identifying actionable mitigation strategies is even more instrumental to improve patient outcomes. The efficacy of debiasing strategies remains much debated [3]—as Campbell pointed out, simply telling individuals or teams to “*be aware of*” and *“apply willpower”* to manage biases does not guarantee effective debiasing [36] given the insidious and subconscious nature of implicit biases [37]. Despite prompting us to reevaluate how we approach team-based cognitive reasoning within a complex real-world clinical environment, our work does not yet grant us solutions. Future work can explore how strategies to address lapses in team cognition can be developed to combat cognitive errors and how to better integrate these into medical education curricula.

## Conclusion

Cognitive biases and errors in clinical decision-making arise not only due to pitfalls in individual thinking but also due to interactions within healthcare teams. We explore how our decisions are shaped by the information we receive from our colleagues, how we decide what information to hand over which in turn frames our colleagues’ decisions, and how other specialists’ decisions influence ours. Collectively, these themes explain how insidious cognitive biases and errors can occur even amongst experienced attending emergency physicians.

Although this study focuses on emergency medicine, we postulate that pitfalls in team-based cognition are relevant across the entire continuum of care and across all specialties of medicine. The hyperacute nature of EM merely exacerbates and condenses these into a compressed timeframe, making the ED *“a natural laboratory for the study of errors”* [38] (as Croskerry describes) where diagnostic errors are most likely to occur [39]. Indeed, similar relays are run every day in every discipline of medicine–some short like in EM, some longer like in rehabilitative care–but all with the same goal of doing the best for the patient while not committing cognitive errors and dropping the baton.

## Supporting information

S1 File Reflexivity statement. (DOCX)

S2 File Participant characteristics. (DOCX)

S3 File Focused group discussion transcripts. (ZIP)
