# Supplementary material for: Dropping the baton: Cognitive biases in emergency physicians
Source: PLoS One. 2025 Jan 2;20(1):e0316361. doi: 10.1371/journal.pone.0316361 (PMC11694980; doi:10.1371/journal.pone.0316361)
Supplement: S1 File — (DOCX) [file pone.0316361.s001.docx]

**Appendix A: Reflexivity statement**

Our study team comprised four EPs of different seniorities (EW, NM, SGG, PJH) from three different institutions. Our principal investigator EW is a residency teaching faculty and former head-of-department whose interest in cognitive biases was motivated by patient safety concerns. She noted that although much research in cognitive reasoning had focused on medical students and residents, there was scarce understanding of the impact of cognitive biases on specialists who are not invulnerable to such errors despite having a large fund of knowledge and extensive clinical experience^[[1]](#endnote-1)^.

NM is a junior consultant and he was able to appreciate how hierarchial differences impacted individual insights: he observed that junior consultant participants tend to contribute a practical, “flesh-and-blood” perspective to the discussion while senior consultants focused on macro systemic issues like department policies and culture and teaching faculty sought to foster growth in junior consultants.

As the patient safety officer of her department, SGG was afforded a front-row seat to the minefield of ED medical errors. She was particularly intrigued by the intricate and complicated interactions between human thoughts and actions, circumstances and environmental factors that lead to cognitive biases.

PJH is uniquely sensitized by his role as current head-of-department and residency teaching faculty to better appreciate the issues which can impact cognitive biases beyond the individual. Through this work, he seeks to bridge the gap between learning and doing, as well as connecting the individual to the system.

Local (FYY) and international (GT) academics with qualitative research expertise complemented our team and provided methodological guidance to ensure rigour in qualitative study design.

1. Elstein AS (2009) Thinking about diagnostic thinking: a 30-year perspective. Adv Health Sci Educ Theory Pract. 2009 Sep;14 Suppl 1:7-18. doi: 10.1007/s10459-009-9184-0. Epub 2009 Aug 11. PMID: 19669916 [↑](#endnote-ref-1)
