# Supplementary material for: Dropping the baton: Cognitive biases in emergency physicians
Source: PLoS One. 2025 Jan 2;20(1):e0316361. doi: 10.1371/journal.pone.0316361 (PMC11694980; doi:10.1371/journal.pone.0316361)
Supplement: S2 File — (DOCX) [file pone.0316361.s002.docx]

**Appendix B: Participant Characteristics**

Our study inclusion criteria included any accredited emergency medicine specialist (associate consultant and above) in one of the three institutions.

All emergency medicine specialists would have undergone a robust five-year residency programme (three years of junior residency and two of senior residency undertaking supervisory duties with specialist oversight) prior to specialist accreditation.

|  | *n* | % |
| --- | --- | --- |
| Rank  Senior consultants  Junior consultants* | 14  11 | 56.0  44.0 |
| Position  Head-of-department (former/current)  Residency teaching core faculty (former/current) | 6  7 | 24.0  28.0 |
| Sex  Male  Female | 15  10 | 60.0  40.0 |
| Race  Chinese  Others | 19  6 | 76.0  24.0 |
| Institution  Tertiary hospital  Peripheral hospital | 11  14 | 44.0  56.0 |

*For this study, we collectively termed participants with the rank of associate consultants and consultants as “junior consultants” to distinguish them from senior consultants who would have minimally 12 years’ of career experience (five years of residency, two as associate consultant, five more as consultant).
