# Supplementary material for: Dropping the baton: Cognitive biases in emergency physicians
Source: PLoS One. 2025 Jan 2;20(1):e0316361. doi: 10.1371/journal.pone.0316361 (PMC11694980; doi:10.1371/journal.pone.0316361)
Supplement: S3 File — (ZIP) [file pone.0316361.s003.zip › Transcripts/FGD 1.docx]

FGD 1

Speaker Key:

EW Evelyn Wong

SGG Sim Guek Gwee

NM Ng Mingwei

PA Participant/s

00:00:00

EW All right. Okay, thanks. Okay, um, yeah, thanks everyone for your time uh, in participating in this study lah. So, um, yeah. We are a group of uh, emergency physicians uh, using qualitative methods to learn about cognitive factors uh, that lead emergency physicians to commit errors.

00:00:36

So, we understand that um, cognitive errors and biases can potentially be a very sensitive topic but we sincerely hope that you can still contribute uh, freely and honestly. And uh, as we need to maintain uh, anonymity given the sensitive nature of this topic, uh, please avoid revealing, revealing your personal ID uh, when you refer to yourself. So you use your participant ID instead, okay?

Um, so we seek your understanding that we will need to record this discussion to maintain the accuracy of the transcription and data collection process. Um, however, please be assured that this a safe environment and your responses will be kept confidential and anonymised. All information will only be used for the purpose of the study and your bosses or HOD will not be privy to any data collected. Nor uh, or whatever you say uh, will be used uh, in, during your appraisal. So be um, assured.

And uh, lastly, uh, please be respectful and treat everyone as equals. We will need to take turns to talk. Uh, wait for each other to finish and not talk over one another. In view of the time constraints and to give everyone equal opportunities to share, we may have to stop you but we will definitely come back to you if time permits.

Uh, I just want to introduce that the, we have two um, moderators here and you know um, and uh, they will be assisting me should I uh, need uh, clarification or missed certain points during this interview, okay? All right. All right. So, uh, if possible also uh, perhaps you could uh, use um, um, your headphones so that um, it is uh, your speech may be recorded um, more accurately. All right.

00:02:29

So, to start off, um, I would like to ask uh, each of you um, perhaps from participant one to um, four, um, how long you’ve been an emergency physician. Participant 1.

PA1 I’ve been in full time at training and practice of emergency medicine since uh, I think 2003. Uh, my first uh, EM posting as a medical officer was in 1999. Um, so yeah, there we go.

EW Okay.

PA1 It’s been a while.

EW All right, it’s been a while. Okay. All right uh, but when were you uh, um, you exit so you got your.

PA1 Um, I exited uh, my fellowship training in 2010.

EW Okay, thanks. Next. Participant 2. You need to unmute.

PA2 I’m Participant 2. Uh, I, I need to do my maths. I cannot remember when I um, AST is uh, 2008. So exit, I will get back to you. Let Participant 3, let me do the maths. Participant 3 first.

00:04:07

PA3 Participant 3 is looking at CV. I also need to calculate.

EW And Participant 4? [Laughs].

PA4 Okay so um, I ended med school in 2012, traineeship all the way until 2017 where I exited. And uh, it’s probably around six years that I’ve been practicing as an emergency physician.

EW Okay, thanks. Two and three?

PA2 I’m Participant 2. Maybe 12 years after exit. 11 to 12 years. Okay, more than 10, less than 15.

EW Right.

PA2 After exit. Okay.

EW And three.

PA3 I think it’s 12.

EW Okay, great. All right so uh, yeah so you guys have been um, emergency physicians for quite a while now. And um, so um, we’re going to um, continue with the, the topic. And um, you can think of yourselves as uh, emergency physicians and this is also about uh, um, practicing as an emergency physician rather than referring to any residence or your previous, your previous self as a trainee. Okay?

00:05:53

So before we um, share more about uh, what cognitive errors are, perhaps you could share with us briefly you know um, about a minute or less, what is your understanding of cognitive errors.

PA1 Errors as I understand is a, is a uh, framework of understanding how errors are made during decision-making processes. And it’s looking at the variety of different methods where um, uh, the, uh, the process flaws within the process or perception um, or uh, data interpretation may lead to error. There we go.

EW Okay, right. Um, who else would like to share what they um, uh, understand by cognitive errors?

PA3 Maybe I’ll give it a go. Um, it is errors made when um, because your, your brain works in a certain way and uh, to digest uh, information data points and all that before reaching a conclusion. And uh, when your brain practices this for a few times more, it tends to um, have certain landmarks or there’s a name for it. Um, heuristic landmarks I think.

That means certain data points that you take for granted and uh, you, you short circuit it because you’ve done it so many times that you feel that certain things are not so important and certain things are more important.. And uh, it falls towards your um, system one type of a thinking. It becomes a little bit reflexive. Once you see something, you conclude something. And because of that, you may not take into account all the data points that’s available.. And uh, sometimes when you do that, uh, you make certain errors and to me, that’s cognitive errors.

00:08:06

EW Participant 2?

PA2 Okay, Participant 2 is me. Uh, I think uh, I’ll give it a shot but probably more layman. Um, jumping to conclusions prematurely with the pre-existing knowledge and experiences of, of the one. Yeah, yeah, yeah, who currently pos, possess, yeah. That’s, that’s yeah. More often than not, leading to negative outcome because there’s an error, right? Cognitive error, yeah. Okay, that’s all.

EW Okay. And uh, participant 4? You want to say something?

PA4 Um, so in my opinion I think cognitive errors are lapses in clinical judgement which are due to deficits in how we process information. . And uh, it might be a matter of how the information is presented to you, how the information is available to you. At the same time, there’s intrinsic factors in terms of how the uh, the clinician’s past experience contribute to this information processing.

EW Okay, great. So just share with you um, uh, um, little um, sort of like uh, explanation of what cognitive uh, errors are. Cognitive short cuts are often used in uh, to help us with our decision-making and to make complex decisions easier to manage. However, these short cuts or heuristics can sometimes uh, lead us astray and uh, um, make us commit uh, serious errors.

00:10:00

So cognitive errors are very common in clinical medicine and everyone, regardless of seniority, is vulnerable to them. Uh, moreover, they may be insidious and difficult to recognise which makes uh, overcoming them not so easy. So we hope today we can work together with all of you to better understand um, what we can do to mitigate errors uh, as emergency physicians.

Um, so I’d like to ask uh, what cognitive uh, errors are most common among emergency physicians.

PA3 Can I start?

EW Yes, yes.

PA3 Um, so at least from uh, my limited experience so far, I think one of the common ones that I encounter is uh, premature closure. Uh, in which uh, an EP will um, there, there are a few. One is that and uh, uh, uh, it also comes together with uh, anchoring wires in a sense that um, they see data patterns of ABC and then they uh, go straight to a conclusion of uh, uh, D. And so, so they tend to close prematurely.

I think also partly because of the, the time sensitive, as well as uh, the stress that we go through because of the numerous patients that we have to see in a very short amount of time. So, once we see three pieces of jigsaw, we tend to try to fix it towards a picture which may or may not be complete because um, patient’s condition change and data points, more and more data points get available uh, uh, as we move along a patient’s progress..

00:11:48

So what happens is that when we prematurely reach a conclusion, we may have a, a, a fixation that this is it and we don’t need to look further. Um, even if things do happen and even if you know, someone else looks further and presents the data to you, you may be so fixated on your conclusion uh, of D that you refuse or you are unable to accept or listen to more information that comes in and you, you are not able to progress after that. .

EW Okay, great. Does anybody else have um, anything to add? Yes, Participant 4.

PA4 So, I’d like to echo what Participant 3 mentioned just now. I do think that fixating on a particular diagnosis, something that is very commonly observed at least in most of my colleagues, and uh, there is a tendency to reject new data points that present later. Uh, and choosing to cling on to the initial diagnosis.

The other thing I’d like to add on that might be slightly different would be perhaps what would be considered availability bias whereby your own personal experiences formulate a diagnosis. So um, what happens at least in our line of work is that we do shifts quite a few times per week.

And therefore in each shift, you might see an interesting case and that might uh, be a very uh, interesting thing that latches on to your memory and therefore you apply this particular case to almost every case that you see subsequently, which might be wrong. So, instead of taking the next case as a fresh one, you tap on what you uh, considered in your previous cases into your new case. Thank you.

00:13:30

EW Thanks. And does any other participant um, want to share what they think is uh, what cognitive errors are more common?

PA1 Um, Participant 1, no, nothing to add. I think the others have said it all.

EW Okay, great. Then we’ll move on. Okay, so um, you might have encountered uh, some examples of uh, medical errors committed by other emergency physicians or yourself, um, after exiting as a specialist. So to what extent do you think these were due to cognitive errors?

So maybe I would repeat. Maybe the sentence was a bit long. So you might have encountered an, some examples of uh, medical errors committed by other physicians or yourself. Um, so to what extent do you think these were due to cognitive errors?

PA1 Um, speaking for errors I’ve done, pretty much most of them um, are, may have either been uh, they’ve either been anchored in um, or there’s been the uh, sort of the, I think it’s called a first pass effect. You see one thing uh, you see one abnormality on an investigation and you miss the other abnormality.. Um, uh, and yeah, that’s, that’s pretty much a lot of the errors I’ve, I’ve, I’ve done. Yeah, that’s been the, those have been, it’s been anchoring and um, uh, premature closure. . Yeah, definitely.

EW Thanks for sharing. Does anybody else uh, want to share?

00:15:40

PA3 Uh, Participant 3 here. I, I, I think based on personal experience as well as from others, um, the tendency is that when I, I, I think most errors are committed because of a few reasons. One is um, more of uh, premature closure. At, at least in my own experience lah. So, so premature closure where you um, reach a conclusion maybe too quickly without considering the rest of the data points..

The second part of errors could be um, maybe less of a cognitive error but uh, inability to um, uh, uh, connect with what you actually know. Um, I’m not sure whether that constitutes as a cognitive error but uh, one example would be that uh, you, you, you assimilate, it’s not a failure to pick up information. You do pick up that information but it may not click with what you actually know.

Um, that means you might notice, you, you might um, uh, uh, uh, you, you might pick up that abnormality and, but somehow it just doesn’t click in your brain that um, uh, uh, the prior knowledge you already know. And because of that right, you are unable to pick up uh, the problem.

You may be able to pick up the abnormality. For example, you may pick up certain clinical signs but you may not be able to link it to the patient’s um, perhaps past history or the rest of the data points. Uh, I’m not sure whether that constitutes as, as a cognitive error or is that just a failure to link with knowledge that you know.

00:17:44

And the third category would be that of um, uh, uh, not knowing, not knowing at all. That means you don’t have that knowledge to actually digest that information and, and, and no matter how you pick up, you just cannot make it click. So that, that is the other category that, that uh, I feel contributes to errors lah. May or may not be cognitive. Because if you don’t know right, then I can’t call it cognitive. So maybe it’s a knowledge gap. Yeah.

PA4 Uh, my apologies, Can you just repeat the question one more time?

EW Um, okay so uh, I said that you might have encountered any um, med, medical errors committed by physicians uh, or yourself as a specialist. But to what extent uh, do you think these were due to cognitive errors?

PA4 So I think personally uh, at least after I’ve exited a large proportion of the errors I would’ve made are due to cognitive errors. I guess thinking from a non-cognitive aspect, that also contributes a fair bit meaning availability of equipment. Sometimes that could result in my inability to make a decision or carry out intervention. And that would contribute too.

The other thing I will also want to consider is the, uh, the teamwork and availability of manpower during my shifts. And sometimes due to the lack of manpower, I would have to uh, carry out other actions that might not be ideal. So those are the non-cognitive aspects. Thank you.

00:19:40

PA2 Um, I’m Participant 2. I echo Participant 4. With regards to cognitive errors, I think maybe the errors that I see in myself and in other people and presented through M&M, would be a lot of premature uh, closure. And fixation. .

Uh, so very commonly is the failure to assimilate new information and change track when patient is dynamically uh, changing the course of the progress the, the estimated or rather the um, the planned progress as expected, rather expected progress. So then you’re still trying to fit the pear shape in a box. Um, so that’s kind of like what Participant 3 is saying.

But what I’m trying to echo Participant 4 is I feel that a lot of restraints um, restrictions such as time pressures, or lack of um, even a computer terminal for us to scroll through the past medical history, it forces us to use what we have available.. And therefore echoing what Participant 3 says, the data points are not complete.

So since the puzzle is five-piece, and we only have three pieces, let’s just make a call in the, you know, in the interest of balancing and weighing. So we inadvertently commit a cognitive error in that sense. You would say perhaps even knowingly. Yeah. My, my take. Yep.

EW Okay, yup uh, thanks for sharing this uh, rather sensitive uh, um, issue, uh, especially your personal experiences. Um, is there other reason why you think uh, emergency physicians commit cognitive uh, errors besides the ones that uh, you have uh, brought up? All, all of you have brought up. Or you could repeat some of them as well.

00:22:08

PA2 Participant 2. Sorry, I go first. So I feel that uh, I think as part of training and as part of the nature of our work, um, clinical service has always been, providing service has always been quite high. So we are trained that way and we move that way. So unfortunately, or fortunately, I don’t know, that’s, that’s the way perhaps we are wired. .

Even in our training, basic training, advanced training and that’s why eventually when we exit, we are like that. Our brain is wired like that to, to, to put it simply, take the risk of premature closure sometimes and therefore uh, performing a cognitive error. I, I feel it’s got to do with uh, that.

We are just wired that way from years of training and perhaps even before training. A certain personality type would choose EM. I don’t know. I’m just postulating. Yeah. And that’s why we are chop, chop, very fast, just closure. Yes. My take. Thank you.

EW Thanks. Yes uh, Participant 1.

PA1 [Unclear] of, uh, uh, of things will lead to um, cognitive errors particularly in EM. Um, uh, echoing what the previous participants said, um, uh, the environment of emergency departments is such that there are many distractions um, that uh, so there’s distractions and there’s some degree of uh, resource um, uh, lack of resources to some extent. Um, uh, in terms of both manpower time. And so those lead to distracters that may um, facilitate errors.

00:24:24

Um, and yes, again uh, there is a uh, there are some features of uh, trainees in emergency medicine um, uh, that we tend to because of the time-critical nature of emergency medicine. We tend to be trained to make decisions fairly quickly. There tends to be a degree of viscerality um, uh, to some of our decisions.

Um, and often we do get a positive feedback that most of time that there’s that little voice that you listen to. Um, and so the, the occasions where it gets most of the time, uh, your preconceptions will, will see you right. But it’s just those occasional times where it will get you wrong.

Um, and so part of it is uh, part of the challenge um, I feel in emergency medicine is, is recognising those, those moments where you actually need to step back and, and think about cognitive errors.. So yeah. But certainly I think the uh, one of the main issues is the uh, the distracters available in the emergency department that lead to a lot of errors. Thank you.

EW Thanks. Anybody else? Yes, uh, Participant 4.

PA4 So uh, I guess I just wanted to add in terms of what has been said so far. Uh, my take on why we are predisposed, at least the profession is predisposed is mainly due to the time constraint which many of us have mentioned already, the scarcity of resources in our profession, and the setting that we practice at.

00:26:27

And I think these two points being the time constraint and resource scarce setting, can be a real reason but at the same time it becomes a very convenient excuse for us to accept sub-par quality of care. And, and therefore we easily make cognitive errors, thinking that this is expected. We think it is normal so it becomes normalised. We think we can’t help but make it.

On that same note, I think uh, in our profession there’s an emphasis on knowing a breadth of clinical diagnoses and clinical conditions but not the depth. Therefore we do not deep dive into it. And therefore we don’t take the time to slow down and think deeply about each thing that we have. We lack the accuracy in diagnosis.

The last thing is I think we all pride ourselves as emergency physicians but because in that pride, we can become complacent. I, I think that’s quite specific for EPs also because we, we feel that we can do a lot of things because we know in terms of breadth, so many things. And uh, that, that ego perhaps will mask us to be reflective uh, and in the end, commit cognitive errors. Thank you.

PA3 I just want to add on uh, a few comments. One is that just uh, a more specific subset of the, that time constraint that we’ve been mentioning about. Uh, um, those very time-sensitive conditions like CVL activation, and sometimes you don’t really have a lot of time to take in all the information. And you are just forced to make that call in a very short time with very limited information.

So one is CVL, two is uh, stroke activation as well which can be very tricky. And uh, um, uh, uh, yeah, where, where you need to make that call uh, in a very short amount of time.

00:28:26

The other point I wanted to make is also the interaction with our colleagues as well. So especially during um, like for example, handovers, and the, um, you know there will be, although you know, there’s no, no rudeness about it and there’s no um, uh, unpleasantness about it, but sometimes there can be some expression of uh, surprise that a patient has not been disposed earlier for example – even if you may feel that um, uh, you need a little bit more information first and the, the worry is that whether if you dispose early, the patient might end up upstairs without you knowing.

Um, secondly uh, it’s, it’s, is um, also with regards to uh, like for example, how far you investigate. Some people may have reservations in the sense that oh, why are you searching for something that is very unlikely to happen? Uh, maybe one example would be like a D-dimer for example, in, in a patient with chest pain.

So it, although you feel that maybe you might search for PE but the person you are handing over to might question why are you looking for PE in this patient, for example. So, so there’s also a little bit of uh, um, uh, expectations from other uh, people, especially whom you’re handing over to. Uh, whether or not um, uh, you feel that oh, am I over investigating? Should I be looking for these things which is I don’t think is likely based on the data points ABC that we have.

00:29:58

And sometimes also we tend to move a little bit faster than um, then we should. For example, in a patient with a septic shock, you know um, I think we have different thresholds of moving towards uh, CVC and uh, uh, IV noradrenaline, that kind of thing. Some people might, might move towards it a lot faster while others might think that okay, I, I’m just going to give fluids a chance first and see whether that helps so that we prevent uh, putting the patient through unnecessary uh, uh, invasive procedures right at the beginning. We give it a chance and see if it works.

Um, so, so different people have different uh, time constraint, the value of time and, and that kind of thing. While some people might want to move to it, towards it really, really quickly so that we can get the patient out ASAP. So in case we get more patients coming in, which is perfectly fine. And on the other hand, we might be trying to avoid uh, invasive procedures. So I see a dichotomy with regards to that end.

Depending on how um, uh, uh, how strong that, that time pressure is, you might opt to limit yourself with regards to the data points that you want to accept or you are willing to accept. Yup, that’s it from me.

EW Thanks, Participant 3. Okay. Um, it is interesting that um, many of you talk about time constraints. Uh, I was um, wondering without time constraints um, then for example, if you never had time constraints, would cognitive errors be totally you know, um, ex, uh, I mean, eliminated? You know, would there be, you know, what do you think, you know? Is it mainly because of that or you know, or without time constraints um, you know, cognitive errors uh, you know, will not happen for example.

00:31:58

PA1 Uh, Participant 1. Um, uh, I think if you took away the time constraint uh, you might reduce the cognitive errors. I don’t think you’re going to get rid of them completely. Um, some of the errors are not facilitated by the time constraints. Um, a reasonable proportion of them probably are um, just, I mean I have no data for that. It’s obviously just an observation. Um, but uh, yeah, some of them certainly are facilitated by the time restraint. Uh, but quite a lot of them aren’t. It’ll reduce it a lot, inevitably. Thank you.

EW Participant 2.

PA2 Yes so I think uh, echoing Participant 1, I think cognitive errors cannot be completely eliminated even if you give uh, you know, a complete ideal environment. Uh, because I think intrinsically uh, there’s still going ons which will defer from individual to individual. So these are the external factors that I think can uh, can perhaps make, I don’t know, worsen the situation, or make it better by mitigating but it doesn’t totally eliminate.

So externally, I think time constraint and what Participant 1 mentioned very uh, accurately I think is distractions. Uh, in the environment of emergency medicine, distractions is uh, I find the number one thing that will really stop your train of thought uh, in whatever you are doing. So that for me uh, is, is, is even higher than the time constraint that I actually uh, am dealing with. The distractions of uh, the whole place. Yup. Thank you.

00:34:14

EW Does anybody else want to add uh, to this?

PA3 Yeah. Maybe I’ll just add in. Uh, I agree with Participant 1 that it will probably be reduced but not eliminated completely. And I think there are a few reasons to this. One is because we have been trained in an environment where there’s already a lot of time constraints. When you learn to ride a bicycle in a certain way um, even when the situation allows it, you are unlikely going to change the way you ride a bicycle.

So what I’m saying is that if you’re trained to think fast with limited amount of data, even when you have a lot of time, you will still fall back on, you know, how you were trained and how you were doing things all this while, even when you have a lot of time. So um, uh, it might reduce it but I think it won’t eliminate it completely.

And also not all errors are cognitive in nature. So, so the example that I gave if you don’t, if it’s a knowledge gap right, no matter how much time you have, you may still make that same uh, error. Um, uh, so yeah.

The, the other part of things is that sometimes right, it also takes uh, experience for somebody to know when to stop and think a little bit more and when to accept a system one way of thinking. That means you just um, uh, accept that limited data and you go forth.

So, I think it takes experience for you to know when to slow down and in which case then you have this balanced concept of system one and system two lah where you know when you should employ more system two and when it’s okay to just carry on uh, uh, um, you know, being reflex. Uh, uh, being uh, reflexive in nature in making your decisions. Yup.

00:36:03

EW Okay, thanks.

SGG Can I just get uh, Participant 3, just now you were talking about system one and system two.

PA3 Mhm.

SGG Uh, would it be possible to um, explain the term.

PA3 Elaborate?

SGG A little bit more, yeah, because I’m not sure everybody knows what you are talking.

PA3 Sure, sure. So no problem. So system one is a bit um, it’s, it’s a thinking where you use a reflex type of um, methodology. Like the, the example is riding a bicycle where you don’t think what you are going to do when you ride a bicycle because you already know how to ride a bicycle very well. You don’t need to think I need to look forward, I need to keep my CG, centre of gravity, low. I don’t need to put one leg in front of the other. Uh, uh, so once you can ride a bicycle well, when you ride, you don’t think about riding the bicycle.

00:36:54

So system two is where you are more deliberate and you have to think every step of the way. Uh, I, I, I need to consider uh, I need to think deeper, I need to consider other things. I need to look at all aspects of a problem. Whereas a, so that’s system two.

So the, the balance um, uh, method of thinking is where you, where, where you feel that you can just offload your bandwidth to system one, you just leave it to system one. So that you have uh, uh, and, and in situations where you need to think deeper, then you use system two.

Because system one is very quick, it’s very fast, and it’s very time efficient. Whereas system two takes up a lot more time but it helps you to um, uh, consider more aspect of the problem. And uh, when things change or when things become more complicated, that is uh, when you need system two to help you to navigate yourself out of that difficult or more complicated uh, situation.

Uh, I don’t know whether that’s clear. What do the rest of the participants think? Am I okay? Do you know what I’m trying to say?

PA2 Yes, Participant 2 answering.

PA1 Yep, Participant 1 as well. Clarified, thank you.

PA4 Yup.

00:38:17

EW Okay. Great.

SGG Okay.

EW So we understand what uh, you’re talking about with regards to system one and two. So I was just wondering uh, so you think um, so which, do you think that um, you know, if we use system two most of the time uh, then there would not be cognitive errors as well? What do the um, you think?

PA1 Uh, Participant 1 here. If we use system two all the time uh, our time constraints would be even worse.. Um, uh, system one, I mean, uh, for, uh, a clear example. Um, uh, cannulation. Intravenous cannulation. That’s for most of us now is system one um, uh, procedure. Occasionally if there’s difficulties, um, we’ll have to move on to system two approach. Actually okay, let me have a look at the anatomy, what’s going on.

Um, uh, for other procedures, more advanced procedures, um, you could argue okay, for central line insertion. Um, uh, is that a system one or a system two process for most of us? Um, uh, and again part of it comes when you are doing it or when you are overseeing it or you’ve been asked to trouble shoot. So certainly in terms of procedural um, uh, processes and errors during procedures, um, you can look at seeing at whether it was, whether it’s a system one task, or whether it was a difficult task um, uh and it should’ve gone on to a system two but didn’t.

00:40:11

Um, but certainly if you try to do everything on system two, as in take everything back to first principles, work out everything, every decision that we make, um, that would be first of all, I’m not sure it would completely remove all the errors because we still have the anchoring, the um, the confirmation bias, all of that sort of stuff going on.

Um, it may reduce some of it but uh, the other issue would be that the timing, time constraint. Um, and just uh, speaking personally for myself, if I have to take everything back to first principles and try and work it out, I’d, I’d be a wreck at the end of a shift. Um, uh, that’s, that’s difficult. Thank you.

EW Thanks. Yup. Yes, Participant 4.

PA4 So unfortunately there, there, there won’t be an absolute where we take up a system two form of thinking and therefore there will not be cognitive errors. Um, admittedly um, what I’m thinking off the top of my head is even if we were to try to employ system two, we won’t be able to employ system two effectively due to the constant distractions. So you won’t even be able to take time to think about it properly.

Uh, and the other thing is that if we were to employ system two, like what Participant 1 has mentioned, there will be quite a fair bit of collateral damage to the rest of our operations in a clinical shift. Because uh, if the turnover is so high, you need to think faster than just the system two kind of a thinking. Thank you.

EW Does anybody else have anything else to add with regards to this?

00:42:03

PA2 Nothing to add, participant 2.

EW All right.

PA3 Sorry yeah, can I hear the question one more time?

EW No, I just asked whether there’s anything else.

PA3 Oh, no, no, no. I mean the original question.

EW Oh, no, the original question, question was that um, because um, most of you were talking about system one um, uh, causing uh, leading to errors. So I asked whether uh, you know, if we were to operate on system two most of the time, then would you know, would that eliminate errors? Um, yeah.

PA3 It won’t lah because um, some part.

EW I mean will it uh, eliminate cognitive errors. Yeah.

PA3 I think probably not all because system two right, all though you adopt a more in depth method of thinking and you try to consider as many things as possible right, uh, if the way you think is still heuristically anchored on certain points and certain assumptions right, uh, you may still make those cognitive errors.

I, I don’t know whether that makes sense because system two thinks, means that you think deep and you think more in depth but if you still make certain assumptions uh, you may still uh, you may still um, uh, uh, fall into that cognitive error.

00:43:29

EW Okay. If possible could you maybe give an illustration?

PA3 So, so for example, certain assumptions like for example if um, the assumption that I just give a very erroneous example ah, that means, if you assume that all young people with chest pain uh, cannot be cardiac. So no matter how deeply you think about it right, but if the assump, that assumption is very, very deeply ingrained in the way you think, then you’re still going to make that error because you, you accord that assumption as a very, very high uh, value. Uh, high clinical value.

But of course that’s wrong lah. But, but if your assumption is wrong and you anchor it as and you accord it a, a great amount of value, then you will still make that cognitive error.

EW Okay, thank you, participant three. Um, anybody wants to add to add to what Participant 3 has said? Okay, if not then we will um, proceed uh, with the next question. Um, so to what, to what extent do you feel that recognising and overcoming cognitive errors are the responsibility of the emergency physician? Or are they likely to be a system-level problem that is beyond the control of the emergency physician?

00:45:00

I will repeat the question. To what extent do you feel recognising and overcoming cognitive errors are the responsibility of the emergency physician? Or are they likely to be a system-level problem that is beyond the control of the emergency physician?

PA1 Um, Participant 1 here. Um, so uh, interesting question. Um, I, I’d break it down into bits. So, there’s the personal responsibility of the physician. Um, that uh, we need to be mindful of how we make errors. And to be aware of our uh, the, both our own personal um, uh, errors that we can make and certainly in terms of reviewing others um, troubleshooting for our juniors, be aware of what errors the, the common traps that they may fall into.

Um, in terms of uh, systems based. So certainly in terms of how, from a deeper mental approach, um, we put in guidelines, pathways, um, specifically to try and reduce um, and flag up certain errors that we make that we, we may be prone to make.

Um, and then in terms of uh, um, in terms of a larger sort of policy um, things that we do, uh, we try and reduce the distractions um, uh, and try to reduce an environment or produce an environment where errors are less likely to be facilitated. Um, so I think it’s, it’s a multi-pronged approach. Thank you.

EW Thank you. Participant 4, you have your hand raised.

PA4 Yeah I do agree with Participant 1. It’s an interesting question to want to accord like a percentage, how much is it self versus a system issue. And uh, I guess for cognitive errors, the, the self should play a role. I’m not too sure whether it’s going to be a large responsibility versus a system because I think it’s only in an exercise of self-reflection can you identify the errors that you’ve made and therefore try to correct them.

00:47:41

Uh, or let’s say now with regards to system, I think the system or the department can also play a part. One example would be the usage of let’s say checklists. So they forcefully tell you to slow down, think about all the things you need to prepare before an intubation perhaps. Let’s say for the RSI checklist that we have.

Another example would be the Syringe Brake device which is a heart stop kind of a check that forces you to make sure you brake off the correct tabs, go through the steps that’s required before administrating the drug, which is usually morphine. So yeah, so both, both does have a role. Both do have a role. Yeah.

EW Thank you. Uh, Participant 2.

PA2 So uh, I agree with Participant 1 and Participant 4 with regards to the responsibility. I think it’s definitely shared when it comes to you know, who, who should bear more responsible over the cognitive errors, I think as a physician, we should do self-reflection. Like we should. So then with that in mind, we should reduce our personal cognitive errors after reflection with possible mitigation uh, mitigating ways to actually maximise like our. I mean the part that we can be responsible for, we should really do it to our fullest and make sure we do that bit.

00:49:26

For the rest of it like what Participant 4 has mentioned, and 1, checklists. Things that are critical. It takes, in fact it actually helps us, yeah, with the cognitive load. Uh, certain things that are critical but yet can be standardised across, and it doesn’t depend on the sole physician to get it correct, and there’s double checking, checklists are very, very important.

Uh, and of course when you come to externally like time, distraction, things like that, resources if can be brought in, uh, that will also help. But eliminating I think primarily the physician has to take full responsibility uh, while working within a system. At least what the physician can do on his or her part. Uh, that means constantly during the shift be mindful and reflective. Uh, and checking and keep checking that you, you avoid, avoid these errors. Yup. Thanks.

PA3 Okay, Participant 3 here. Uh, agree with whatever has been said lah. It, it, it’s two parts to the puzzle. Uh, there has to be self um, there has to be the inclusion of uh, uh, self-awareness and self-reflection as was what was mentioned. And two, uh, the system must allow, must support it as well. Uh, checklists have been mentioned and, and, and, hard stops have been mentioned as well.

Just one additional thing I think would be manpower. So one very specific example would be um, let’s say you have a, a, very, very sick trauma patient and then you, you, you don’t have the manpower to do the procedures that needs to be done. So what happens is that you get sucked into it. You do it. But when you are doing the procedure, you are develop, you are devoting your bandwidth completely to the procedure and you may not be able to see the big picture of what’s going on out of it.

00:51:39

So if you have enough manpower, of course you can afford for one person to just hands off and look at everything that’s going on, coordinate care, make sure that the priorities are being take care of. And uh, that helps to reduce uh, cognitive error as well because now you have more bandwidth to, to fully employ your system two to think about what’s going on, suck in all the data points that you need, and, and I think that will reduce uh, uh, cognitive error in a more specific way. So in summary it’s both lah. Both system as well as self.

EW Okay, thanks. Uh, thank you. Um, so uh, following up then, so uh, what cognitive errors should new specialists be aware of and what advice would you give them? So what cognitive errors should new specialists be aware of? And what advice would you give them?

SGG I think uh, if I can just tap in, I feel like um, we have like a, especially when I, I listen to Participant 2, um, there was some mention about how uh, we’ve been trained uh, during residency or during our uh, EM postings to learn how to take risks and learn how to take risks safely. Um, and then even at the point that you are EP, that’s still uh, is in your psyche. It’s still in your, you know, your genetic makeup when you come to work.

00:53:20

Does that change over time? Do you guys think that uh, it evolves over time? So as an EP now, as a trained specialist, do you feel that that risk taking has changed in a way? Does that affect the kind of cognitive errors that you make? Versus let’s say when you, if you think back in time when you were a resident or when you were a, MO. Has that, has that uh, has the kind of cognitive errors that you made changed over, over that period?

EW I think you have to repeat that question because it’s uh, kind of long. Yeah.

SGG Yeah so um, is there a difference in the kind of cognitive errors that you are making now versus uh, when you were uh, MO or resident?

PA3 Okay maybe I will start lah. I, I, I would like to think that the cognitive error rate is less the older you get. Um, of course I don’t have data to back myself up and uh, but, but, and I totally and I feel that, that is so.

Um, the, the, part of the problem is also because when you are younger and you don’t really have that much experience, so you don’t know um, you may not know that uh, you, how do I put this? That, that means you may not see the error of your ways until you are a little bit older, in the sense that because along the way you made errors and you see the consequence of your errors. And during that time then you realise that um, okay, certain things should not be done, certain risks are not acceptable.

00:55:08

And risks um, are subject to the, the, the, the probability. That means that if let’s say something that you did ah, um, uh, resulted in a consequence that only happens in a very, very, very rare circumstance. But because you, to you it’s 100% even though in the general population it’s 0.01%. So you might be super careful henceforth with regards to a certain subset of patients that you see. And, and, and um, that might uh, also make you less, or rather more risk-averse and less willing to take risks because of the consequence that you had to face. Uh, uh, after that lah.

Um, I’m not sure whether I’m answering your question uh, but I do think that yes, with experience, it, it changes your risk appetite lah in that sense because of the consequence that you had to face.

PA1 Um, par, Participant 1 here. Um, yes. Echoing what was said before, uh, partly I think it is um, uh, as we gain more experience, then we become more aware of risks and consequences. Um, and so uh, I personally find that I am a bit more risk-averse um, uh, um, uh, the, the more I, the more I see.

Um, and I feel that that is reflected um, again, I have no hard data for this, but um, uh, a general observation is that we tend to be a bit more risk-averse the more senior we get. Um, uh, and uh, yeah. So yes um, that’s the answer. Thank you.

EW Thank you.

00:57:17

PA2 Um, Participant 2. Uh, as EPs I think uh, echoing whoever mentioned previously that our practice, I think Participant 1, our practice is very broad. So similarly I would like to think that because it’s so broad, um, even after we finish training, even though we are wired in certain way, we are still learning a lot through clinical experience after having exited. And thereby making some more new connections and thereby leading us to being more risk-averse. Because the more we see, we see the consequences presenting at M&Ms and things like that.

But so, so, I’m not sure if it’s just us as emergency physicians because then we haven’t seen everything in our training and we continue to see after exiting. I would also want the actually in other disciplines, especially the surgical disciplines where it’s quite procedural, you know, and then do they you know, when they get older and more time in that specialty, are they opposite of us?

Because then they are doing the same things again and again and again. Are they like actually less risk-averse because they’ve done the thing so many times? So I’m not sure. I’m just like yeah, tying to put it out there. I don’t know whether it’s unique to EP that as we grow older. Because Participant 1 and 3 said they are more risk-averse as they’re older. And so am I. So that’s like three of us already. Yeah. That’s, that’s just a random thought.

EW Okay just coming back to the question. Uh, the question is uh, um, not being, not about risk, being risk-averse. Um, but rather you know, have you changed, has the cognitive errors that you may have committed when you were younger as a trainee versus now as a specialist, um, have, have they changed over time? Yeah.

00:59:41

PA4 Um, yup so with regards to that question. I, I guess I’m probably the one who is uh, the youngest and therefore can recall my trainee days and anything. Um, so I think at least during my, when I was in training, cognitive errors is a relatively new term and I was probably introduced to it only when I exited. So not during my training. And I think perhaps it could’ve been useful then.

So if I didn’t even know what a cognitive error was, I can’t really label it. So I can’t say for sure whether I have got a cognitive error unless my supervisor tells me so. And I would therefore think that when I was in training, um, it’s more likely that some errors that I made would be due to a lack of medical knowledge, purely because I’m still in training as compared to it truly being a cognitive error. But I guess the counterargument to that is if I didn’t even know what’s a cognitive error, how will I know I’ve committed it?

Um, moving on, I guess when I exited and knowing that there was such a term called a cognitive error and looking back, then I could therefore now label the errors that I’ve made previously when I was training. And because now I know about this and I’m more aware, then I’m less likely to make similar errors. Doesn’t mean I don’t make errors but the similar errors might, might be fewer. Yup. So I think there is a change as you gain more experience.

And on top of that I guess it’s very useful once you’ve exited the departmental meetings such as M&Ms, serves as a very useful platform to highlight these issues. Thank you.

01:01:21

EW Thank you. Uh, does uh, system mod, moderator have any other question?

SGG No, I’m good.

EW Okay. Uh, did, have they answered your question? Okay, all right. So maybe I’ll go back to the other question. Uh, the other yeah, a few more questions that we have. Uh, what cognitive errors uh, should new specialists be aware of and what advice would you give them? Yes, Participant 1.

PA1 Uh, um, the most important advice I would give to a new specialist um, uh, uh, number one, you are going to make an error. . Um, uh, and uh, be aware of it but don’t be scared of it. Um, uh, to avoid um, avoid distractions.

Um, and part of it is situational awareness. Um, realising that if something isn’t quite going right, you sometimes need to have a step back and rethink because that’s usually when you’ve committed an error. Um, where you think okay, this is the case, it’s going to go in this direction, and there’s just a few little things that aren’t quite going right. Um, some of the parameters haven’t changed the way you would expect them to.

And then you have to think okay, have I got the right diagnosis? Have I anchored my diagnosis? And so I think awareness of those situations is where a lot of um, uh, newer trainees uh, and uh, uh, people make their errors.

01:03:32

Um, the other thing is uh, it, it’s both ends of the spectrum. There is the overconfidence um, uh, and certainly uh, um, yeah, I’m sometimes guilty of this, uh, that you think okay, I can, I can do this. And actually no, uh, maybe you need someone else to come in and help.

Um, and then there’s this. The other thing is what’s called the zebra effect. Uh, uh, which is if you’re faced with a rare diagnosis, you’re less likely to interpret it as such. Uh, and so we tend to try and think oh no, it can’t be that. Um, uh, classically things like um, what we all worry about is things like aorta dissection, pulmonary embolism things like that where it’s like nah, it’s not going to be that, um, that’s really rare. Um, and so it’s uh, that, that’s the other thing that if the data is leading to it, it might be rare but you need to consider it.

And so certainly from when I was training to now, we now say if it’s part of your differential, you are duty bound to exclude it. Um, and so uh, meaning when I was trainee, if you wanted to do any sort of uh, 3D imaging, CT scans or things like that, there was a massive battle that you had to do with that radiology department. So again, coming back to the resource. And that tended to make you less willing to commit to that diagnosis. The rare uh, condition.

Um, nowadays, it’s a lot easier and I think that has reduced that error rate. But again, it is something to consider certainly for the, for both the new trainee and the newly qualified um, uh, senior. So yeah, that would be my advice. Thank you.

EW Thank you. Participant 2?

01:05:43

PA2 I think my advice uh, to them would be um, to practice mind, mindfully. Um, especially the more chaotic it is, the more time pressed you are, the more deliberate you have to be in your thought processes. Uh, put the ego aside and ask for help, even as a specialist when uh, our resources are stretched. But if um, we feel we need another pair or hands, another brain uh, sometimes that is what we need to get us out of something that we, we find ourselves like really quite deep in. Yup, thank you.

EW Thank you.

PA3 Uh, Participant 3. I think the uh, advice that we can give is probably just to make them aware that um, certain cognitive errors tend to be common.. And I think like what been said before lah, if you don’t even know about it right, then it’s very unlikely for you to be able to do something about it.

So if we can alert them or you know, educate them what cognitive errors are, and uh, how people can be prone to them, uh, especially in a certain stage of their lives because pride comes before fall, uh, when they exit, they may have a false sense of security or false of confidence lah. So I think just helping them be aware of these things may hopefully help to prevent some of these. Yup.

01:07:41

EW Thank you.

PA4 Um, what I wanted to say was very similar to what uh, Participant 3 has said. So I guess the first part of the question was with regards to which of the errors they are most likely to make or something like that. Is that correct?

EW Yes, what’s most likely, uh what cognitive errors should new specialists be aware of and what advice.

PA4 Yes I think it’s a very broad question because they are prone to, they are still prone to all cognitive errors. And I think that’s uh, links to why. And the why is because there, there might be a, a feeling of superiority once you’ve exited. Because you’ve passed the major exam and now you are all powerful. And, and that confidence might lead you to commit these errors.

And therefore the advice would be very similar what Participant 3 has said. Um, not only should be very careful once you’ve exited but at the same time, be, don’t be shy to obtain feedback from people. Don’t be shy to ask for help. Yup, thank you.

EW Thanks. Okay, Participant 3 said to um, you know, help the new specialist learn about what a cognitive errors are. Um, when should any you know, physician learn about cognitive errors?

01:09:08

PA3 I think as early as possible.. So alluding to what Participant 4 had mentioned that during uh, his time of training he wasn’t quite aware of these things. So if we can bring this awareness early on and uh, allow them to understand that is something that humans are prone to uh, then they can be more deliberate in their practice lah, to try to avoid these things. And perhaps if you avoid it long enough, the, the measures that you take become second nature. And perhaps that might help you to avoid it in the future as well.

The other thing that uh, sorry, I just go back to that, that, the previous question, right? Another, another thing which may help is that, because once you exit right, um, you don’t really have anybody to look over you the way that they did during residency. So which means that the amount of feedback that you get is a lot less.

So if you can, if we can set up uh, a, a network of peers to use, employ peer coaching or peer feedback techniques right, that could be better. Because it’s very hard, once you graduate right, to have people to give you feedback. And then the tendency is that why are you doing this to me, you know? Uh, are you out to get me, that kind of thing.

But if we have the understanding that okay, you know, you have a group of peers or friends who are there because they want to help each other improve, then perhaps that may also help with prevention of cognitive error. It’s because then the feedback may be less of a knowledge type of a feedback, patient-care type of a feedback, but it could be more targeting towards cognitive errors. Because uh, hopefully by that, when you exit the MK and the PC part of thing should be less of a problem already and we could focus a bit more about how you think, how you anchor certain things in your mind and, and things like that. That could help but I don’t know if it will.

01:11:08

EW Thank you. Participant 2. Sorry, Participant 3, what is MK and CP? PC.

PA3 Sorry, sorry. MK is medical knowledge and PC is uh, patient care.

EW I see, thank you for clarification.

PA3 Sorry.

SGG Yup um, so Participant 3 says uh, they should learn about cognitive errors as early as possible but how early is early? Yeah.

PA3 If I had a, if I had a choice, it would be at undergrad. .

PA1 Participant 1 here, agree. It should be at medical school..

PA2 Participant 2, as early as possible. .

PA4 I disagree though. I, I think that if you don’t have your MK built up properly, it’s very hard for you to appreciate the cognitive errors also. Because you’ll be struggling to toggle between both, to appreciate how to recognise the cognitive error and solve that cognitive error. Um, so if anything, I would think it’s good to introduce such a thing in residency training perhaps in the SR years or in Year 3, as compared to the junior residency years. That’s my take on it.

01:12:24

EW Okay. Um, does anybody else um, Participant 1, 2, or 3 um, have any different, differing views and why?

PA3 Um, maybe I just add on a little bit. I, I felt that because cognitive errors may be independent of uh, knowledge, because it’s a method of how you think and how you assimilate information. It may not be specifically medical as well which is why I think having it early is good but it doesn’t mean that we stop there. Uh, there should be revisits to it at different stages of a practice lah.

I, I guess the first stage is just to let them know that there is such a thing. And it can happen um, outside of medicine as well, when you make decisions um, buying a house, buying a car. That, that kind of thing. You want as much information as you can, et cetera, et cetera lah. Or, or even making decisions in life. But, but what I’m, what I’m saying is that you know, you can start early but the engagement doesn’t stop there. It can proceed as um, things change at different stages of your life lah.

EW Yes, Participant 1.

PA1 The how cognitive errors are made, I appreciate what uh, Participant 4 is saying. Um, yes that it’s at a certain point in training it becomes a higher thing but I think also at the start of training it is an independent process from the medical knowledge. It’s uh, it’s understanding decision making and how decisions are made. And I think that insight into that process and certainly one of the key uh, uh, talents of uh, physicians in all specialties, is the decision-making process. And I think understanding that and having insight into that um, is a key skill. And I think it should be taught as early as possible to have that insight.

01:14:51

Now I agree that yes it should be revisited again in the senior years of training. . Once you have the medical knowledge you can then have further insight into how some of those processes come into play in each specialty. Um, and I think yes, that should be a rest of the uh, um, at the higher uh, years in, in residency training. Uh, but I think certainly at its basic level, it should be uh, presented very early on um, in understanding how decisions are made. Thank you.

EW So um, Participant 1, very early on meaning? How soon?

PA1 Med school.

EW Medical school.

P1 During medical school. So understanding how. And understanding it in a board spectrum within all specialties. It’s not just um, emergency medicine that is uh, that can fall into these cognitive errors. Um surgical specialties, public health, there’s a whole lot of decision making to be made and can be prone to cognitive errors. Uh, um, general practice, pathologies specifically. Um, uh, and there’s understanding how these decisions are made, how errors are made I think is one of those things.

Certainly when I went to med school in the last century, um, uh, there was no, there was, this concept was not taught. Um, and I happened to come across this years later as a trainee because of some psychologists um, and, and uh, educationalists. Um, so uh, yes, I think it is important and I feel that knowing this as a medical student, as a uh, houseman say junior resident, um, understanding this may have uh, may have given me more insight into that practice at that earlier stage. Thank you.

01:16:51

EW Thank you.

PA2 Participant 2.

EW Yes.

PA2 So I agree with Participant 1 and 3 that it has to be taught at least in theory the concept uh, in undergraduate years. . The appreciation would probably come later when your MK is all buffed up. But knowing it is important just so that like what Participant 1 has alluded.

No matter what discipline you go in, all you know with that in mind, they can actually identify as they go along. And then after when it’s revisited in their traineeship or residency, be it junior or senior level, the appreciation is, is deeper, is greater. And uh, it wouldn’t be like something that is that is suddenly introduced to them mid way and that they have to adjust you know, their wiring in that sense. Thank you.

01:17:50

EW Thanks. All right. So um, I have one, another last question um, so I’d like to ask you all um, what strategies do you use to help you mitigate um, cognitive, uh, mitigate and prevent uh, cognitive errors? What strategies do you use to help you mitigate and prevent cognitive errors? Yes, four, Participant 4.

PA4 Um, so I guess for me one of the things is I am uh, more consistently saying no to things now. And the reason is that uh, we know that there’s a lot of distractions in our department or at least the, the place that we work at.

And so one example could be I’m trying to vet a case from MO, in comes an ECG. I will say no, I’m going to finish vetting the case first before I do anything with the ECG instead of dealing with the ECG. So this allows me to focus on the task at hand which is to vet the case and uh, assimilate whatever clinical information is provided. Yeah.

Uh, that’s the same thing as someone comes to you and say that could you come over to the triage to review this patient because uh, or whatever it is. Uh, there’s a rash, do I need to bring to fever? There are things I guess I will probably sometimes delegate this particular task to another one of my colleagues. Eh, actually can you go and see this, sign this ECG for me first? So yes, saying no is one thing.

The second thing is I will purposely slow down when therapy does not work the way that I expect it to be. And start reconsidering everything from the start. Seeing whether there’s any uh, steps that I’ve missed along the way, any errors in terms of the clinical decision that I’ve made along the way.

01:19:52

Last but not least um, I’ve also uh, constantly try to ask for help from my colleagues when I have questions with regards to imaging, investigations. I’m not too sure whether I’m seeing what I’m seeing is correct or not. And this feedback is very useful. Either to help me uh, as a group effort get the diagnosis or to change what I initially thought was the diagnosis. Thank you.

EW Thanks.

PA2 Participant 2. Uh, I think Participant 4 just said everything that I wanted to say. And I think he, Participant 4 has uh, I think said started saying no which is great because I’ve only started saying my first no two weeks ago to an ECG [laughs]. When I was in discuss. So yes, um, that will help with the distractions. Uh, trying to say no, not all the time, but really when uh, things are coming to a head and yes, I think we need to learn how to say no.

But then again, we also have to bear in mind when we say no, it comes back to us later. . Uh, and we also need to balance that, that no that we actually did just delivered and what if it’s, it’s an ST elevation kind of thing. So yes, it, it has its merits just saying no but then I think I’m still hesitant sometimes because of the possibility of delaying something else.

Um, two, uh, like what the same advice as I would give a new uh, EP who has just exited. I go to work telling myself I want to practice mindfully and like what Participant 4 said. The crazier it gets, well, in not those words, the crazier it gets, the more deliberate, the more I want to think things through and properly. But not in a very slow manner. But in a way that I try.

01:22:01

Because you know that you’re going to probably do a lot of premature closures. And then you may still do them but you knowingly do them with uh, certain risk. And sometimes you may be because knowing that, you may revisit it like uh, review, like after you have actually premature closed it when the opportunity comes.

And then um, what was the last thing Participant 4 said? Yeah. Uh, I have no ego so that’s great. Uh, I love calling a friend. Yeah. So then I think fortunately um, I think our work culture, our workplace, uh, encourages or does not uh, put to shame any or at least from what, how I feel, you know, I can, everybody is friendly. And if and when I am, I can’t sort certain things out, the pieces don’t match, I’ll always um, you know, bounce off somebody else just to see exactly like what Participant 4 said. Am I missing something? This is why, why doesn’t it click you know? Am I, should I consider something else? So yes, that’s all. Thank you.

PA3 Participant 3 here. Uh, most of it has been said. Just one thing else is that um, I try to ask myself a few questions. One, what else can it be? What am I missing? So I just keep constantly asking myself this and uh, through this I don’t know, self-interrogation right, I hope to try to avoid cognitive errors lah. Yeah so that’s, that’s, that’s all.

01:23:55

PA1 All’s been said uh, the other thing that I would add uh, is that I try um, try, uh to be as open as possible. Um, if I’ve made an error, uh, um, I try and let people know yes, that is an error, this is what I’d done, this is how it went wrong. Um, and try and be self-reflective.

Um, I still make errors. Um, uh, but I think it’s important that we are, and certainly we are as a department um, we are quite open. We have a very good reflection uh, and feedback process um, uh, that is collaborative uh, rather than uh, confrontational. Uh, and I think that really helps um, with uh, certainly it has helped me reflect on practice, change practice where it’s been required. Um, uh, but I think that process is also important. We are going to make errors uh, but I think part of it is being open to it um, and helping each other through.

Um, yeah, shout for help. Uh, um, uh, constantly seeking second opinions and um, uh, just going through. Sometimes I find if I’m stuck, if I can get a second pair of eyes or a second, just second person to okay, let’s just go through this case again, what am I missing um, that’s a, another thing that yeah, I do mention quite regularly. Um, uh, yeah, thank you very much.

PA2 Sorry, Participant 2, just a little bit more to add. Uh, Participant 3 was saying what can it be, what can it be, keep asking myself that. So I think having the practice of having um, three to five differentials for every case that I go through um, helps me when so-called surprises come up such that you know, um, I’m not fixated on the currently diagnosis.

01:26:11

Um, and the other thing about asking for help uh, second opinion things like that. Um, I find that even the, the MOs who is presenting to me um, I try uh, to also have a discussion rather than “I tell you to do this”. I ask them for their opinion and bounce things off them. After all they are the people who have spoken to the patient and their relatives and gone through all the notes.

Um sometimes you know, I feel maybe some of us uh, as I’ve observed at work, after the MOs goes like three to five lines and they scroll the computer note okay, this is this, you do this, you do that and then uh, wrap up and then post. So I find uh, even uh, bouncing off the MO who is presenting the case to me, they may have something valuable to add, even though they’re not my peer but they are the person, the people who actually saw the patient, uh, first-hand. They may have something valuable to add, something that I, I wouldn’t have realised. So that I find is helpful to me on the ground. Thank you.

EW Thank you. So I thank everybody for being here. Uh I just want to know if anybody else has any um, burning thing to say about cognitive errors that we may not have covered, uh, including the moderators. You know, perhaps you feel that you have not been heard uh, in a certain area, um, or we’ve not asked you the right question to bring that out.

01:27:47

NM Hi, I’m just wondering uh, I think Participant 3, you mentioned earlier that um, a lot of it is also related to interaction with colleagues, I understand. So just wondering um, because a lot of times it’s, there’s a lot of semantic qualifiers like when the medical officer presents a case and they use words like this is um, painless jaundice uh, for one month. Then immediately um, we go down a certain track, but there might be a bit of pain.

Or, or so, so could it be that we have already been ingraining into our medical officers that they need to sell a particular diagnosis and they are doing such a good job with all this description that it ends up that it becomes inevitable for us to commit the errors because they are already framing it in such a way? Or, or, or could it be still something that we can do on our part to mitigate this?

PA3 Um.

NM Sorry, very long question.

PA3 I, I, I think entirely relevant and uh, I think you are right in the sense that you know, because patient care starts from uh, medical officers in the case, and at the very end it will be someone who is vetting it. Whether that person will go retake the entire history, and redo the physical exam - of course it’s uh another story lah.

So if the patient’s condition is already heuristically framed in a way that there is uh, that, that, that leads you towards a cognitive error, then of course you are setting it, it’s a set-up lah in, in another words. Like uh, uh, a famous quotation I heard yesterday is that if you are a hammer right, everything will look like a nail. So if you are trying very hard to um, if, if you have a certain framework in your head and you are trying very hard to squeeze it to fit in a particular shape, right, then of course it will have uh, significant downstream effects lah.

01:29:45

Which again you know, there, there’s a bit of a cultural thing going on, because if you are not able to make things fit in a way, then you have a problem after that. Where, where um, you, even a pain score of 0.5 you would say that that is not, that is pain.

And, and you know that certain symptoms are very subjective. Pain, discomfort, you know, how many patients who, who come in with MI tell you that they have no chest pain but they have a little bit of chest discomfort. So um, I, I, I don’t really have an answer to that actually.

PA1 Um, Participant 1 here. Um, uh, just to add something else. So one of the things I do particularly when I’m speaking to the medical officers um, is a common question I ask them is, what is your diagnosis. Because one of the things that a lot of them have been taught is to although they collate the evidence, they are still uh, presenters rather than interpreters. So you’re trying to go through that interpretation process with them.

And by trying to go through that process, by having a formal process, by formalising that process, that slows you down and that sometimes prevents um, some of the cognitive errors from seeping in. Um, so that, that’s one of the things I tend to find helps. Um, and then also by going through it, you will recognise that oh, actually, often as you’re going through it they’ll say oh, I didn’t think of that or I didn’t do that um, certain uh, key um, uh, findings. So that, that I find helps me quite a lot. Thank you.

01:31:46

SGG Thank you. Thank you everyone.

EW Does anybody else have uh, any other you know um, points to share? Or something that we may not have covered but you feel that you know, it’s important for us to understand about cognitive errors?

PA3 Participant 3 here. Uh, I, I think it is quite interesting being part of this interview lah and I, I appreciate being invited for it. And I think uh, one of the take homes that I have as well right, coming out of this is that um, even you know, uh, uh, when we, when we are vetting cases or we are supervising our MOs, it’s one thing to be aware of uh, our own cognitive bias but it is also important to help the junior ones, the MOs and all that recognise that this can be also a problem.

And I think one of the things is brought, that was brought up is that it’s good that if we could educate them earlier in their uh, careers about this. And I think opportunistically we can do that and we can also uh, share with them some of the methods or some of the experience that we had which resulted in a bad outcome. Or examples of cognitive errors that we or uh, others in the department have made to make them more aware. And uh, also some of the things that we employ to help ourselves avoid these cognitive errors lah. Like what was mentioned, the deliberately slowing down, da, da, da, all those things, and sharing it with them, I think that will also help uh, with regards to the bigger uh, picture and bigger scheme of things lah. That’s all I have to say.

01:33:23

EW Thank you. Do the moderators have any other questions to ask? Okay, so um, thank you all so much for your time. Uh, and we appreciate your uh, honest sharing with us. Um, we assure you that uh, your responses are um, strictly confidential. And um, yeah. So um, yeah, if there’s nothing else, um, that anybody has to share, then we will call this meeting a close and thank you all again.

PA2 Thank you so much for having us.

PA3 Thank you.

PA4 Thank you

PA1 Thank you, that was quite insightful. Thanks very much.

PA2 Thanks everyone.
