# Supplementary material for: Dropping the baton: Cognitive biases in emergency physicians
Source: PLoS One. 2025 Jan 2;20(1):e0316361. doi: 10.1371/journal.pone.0316361 (PMC11694980; doi:10.1371/journal.pone.0316361)
Supplement: S3 File — (ZIP) [file pone.0316361.s003.zip › Transcripts/FGD 2.docx]

FGD 2

Speaker Key:

VRM11 Pek Jen Heng

EW Evelyn Wong

PA Participant

00:00:00

So can we start by asking each of you, um, how long you have been an emergency physician? So you can tell us less than five years, five to ten years, or more than ten years. Okay? So maybe we have participant number five go ahead first. Just remember to unmute.

PA5 Participant five. So as a resident, uh, five years. As a specialist, uh, for four years.

00:02:12

VRM11 Thank you. Participant number six.

PA6 Hi. Participant six. Uh, specialist, uh, 14 years.

VRM11 Thank you. Participant number seven.

PA7 Uh, as a specialist for more than four years. Yeah. Four to five years. Yeah.

VRM11 Four to five years. Okay. Thank you, participant seven. And participant number eight.

PA8 Participant number eight. Uh, five to ten years.

VRM11 Okay. Thank you very much. All right, so, um, I hope it’s not too awkward for everyone. It will slowly get a bit more comfortable as we start, uh, going through the discussion. So I’m just going to get started proper.

Okay, think back on some of the medical errors committed by other emergency physicians or yourself after existing as a specialist. So we are really focusing on, um, you as a specialist, or your colleagues as specialists. What are some of the common circumstances or factors that lead to medical error among specialists? That’s the first question. Repeat again. What are some of the common circumstances or factors that lead to medical errors among specialist emergency physicians? Anybody can answer.

00:03:24

PA5 Participant five. Uh, one of the common things that I notice, uh, is that when a diagnosis or somebody has evaluated a patient and given, uh, that information, that information tend to stick with the second or the third provider, and they follow through with the same assessment without making an independent assessment. . Uh, that’s number one.

Number two is that sometimes, um, when the evidence is not complete, when there’s like partial information, and they already pigeon hole the patient into a particular diagnosis, and they use only half the information and not the complete information. Um, that’s all I can think of for now. Thanks.

VRM11 Thank you very much, participant number five. Anybody else have anything to add?

PA6 Participant six. For me, it’s, um, like things like manpower constraints, um, having to multitask as a result, uh, of that, and sometimes in, in multiple areas and zones, different priority. That is not safe, I feel, and that makes us make mistakes, um, and our focus is not solely on one thing at one time. Um, it’s difficult to achieve given our work, but there’s only so much you can stretch, and beyond that, it becomes unsafe and dangerous, and then we make mistake.

VRM11 Thank you very much, participant number six. Do we have anybody else that want to share on this particular question?

PA8 Uh, participant number eight. Um, so for me, when the shift is very busy and I don’t have enough time to allocate to each patient that, uh, is under my care, sometimes I trust the junior doctors more… And sometimes we can become anchored, especially if we don’t have time to eyeball the patient and see them ourselves. That’s number one.

00:05:30

Number two. Uh, when cases are handed over to us, and, uh, the case may be partially worked up, and if it’s not handed over properly, um, we might be anchored that, okay, the case has been settled. Uh, I don’t want to do too much because there are new patients for me to take care of. Hence, uh, the case left to stew on their own, with minimal follow up. So that’s also another potential, uh, risk that may happen. Over.

VRM11 Thank you, participant number eight. I will now move on to the next question, if nobody has anything else to add. Okay. We heard a little bit about how our ED circumstances, uh, help to contribute to cognitive error. I just want to know, um, to what extent do you feel cognitive errors play a big part in committing some of these, uh, medical errors, focusing on cognitive, the way we think. Um, anybody? Participant number eight, you are unmuted. You want to say something?

PA8 Sorry, forgot, forgot to mute.

VRM11 Oh, okay.

PA6 Actually, I don’t understand the question. Can you say again? [Overtalking].

VRM11 Maybe I… [Laughs]. Don’t worry. Maybe I rephrase it a little bit. Like, um, we, we, we hear a spectrum of, um, errors, uh, medical errors being committed. Um, some of these, as you have shared, is due to our circumstances, like a busy ED we work in, uh, limited manpower. How much do you think it’s, um, due to cognitive error? Like maybe a small portion, moderate amount, 50% of the errors, or something higher. How do you all feel?

00:07:20

PA5 Participant five. I, I feel that it does contribute, but it’s not, uh, not a very high percentage, because there are also other causes for medical errors, um, which are not due to cognitive bias. Other than, uh, being busy and having a lot of patients, I would say, probably, 20%. I cannot give an exact figure. But in my practice, even though there might be some cognitive bias, but the error, it’s not a big error. It’s probably just, uh, differential diagnosis or some partial issues that are overlooked that, uh, usually, the medical team can follow up. Over.

VRM11 Right. Thank you, participant number five. Anybody else? Um, what…

PA8 Uh, participant number eight now.

VRM11 Please go.

PA8 Um, definitely, I feel that, uh, if the ED is very busy, there are a lot of lodges. You come to shift, and you see like three patients in your area waiting for bed, and the whole area is very congested. Cognitively, you already feel very burdened, even before your shift start. So even if you have adequate manpower, you know, um, the fact that there is people in your line of sight asking for help, asking to go to the washroom, calling the doctor, calling the nurses, that adds a layer of burden to the physician’s mind..

00:08:46

Number two. I feel that maybe each doctor has capacity to maybe see about, I don’t know, 40 to 80 poly patients in a shift. And if the shift is… Uh, if you have very trained manpower, who can help to do 80% of evaluation right from the get-go, your capacity can be stretched bigger. But conversely, if your manpower is short, everyone is very junior, there is a heavier cognitive… You need to spend more time evaluating the cases. Your capacity will drop.

And, and this is how I feel that, um, all these things eventually… Eventually, when your patients exceed your cognitive… Your, your, your, your capacity, your mental capacity, eventually, um, mistakes will be… There will be a higher chance for mistakes to happen. Over.

PA5 Participant five.

VRM11 Go ahead.

PA5 Sorry. Um, actually, I’m just trying to distinguish between cognitive load and cognitive bias and… But from the sound of, uh, participant eight, actually it sounds more like it’s a lot of patients, a lot of lodges, and that is actually contributing to the cognitive load. Um, I’m, I’m not sure whether that is equivalent to a cognitive bias. Over.

00:10:01

VRM11 All right. I think that will be brought up later on, as we go into a little bit of discussion. Um, but I think at this point in time, it’s good enough to say that, um, if you think that anything that can affect the way you think, be it because, uh, of bias or because of load, that have resulted in some form of errors being made because of the way we are thinking, we will accept all that now at this point. So feel free to… I hope that clarifies a little bit, and you, and you can feel free to contribute in this, uh, [inaudible].

PA5 Participant five. Then I think, uh, the amount of error probably is higher than what I previously quoted. Over.

VRM11 Okay. Roughly, how much higher, if you mind to clarify a little bit?

PA5 Uh, as high as, probably, 80%.

VRM11 Okay. Okay. Thank you very much. Do we have any other input from the other participants on this?

PA6 Hi. Participant six. Um, I think that also, um, the amount of stress that you feel when you come into that situation that participant eight mentioned, where your… You know, you have lodges overflowing in your zone, and then instead of 18 beds, you have six beds with which to see all the new patients for the next nine hours, that sort of situation puts you in a different stress level.

And the, and the, the need to move the patients, settle them, and get them out of those six beds because the next six are at the door and in the corridors, that stress level itself makes you have a different way of thinking, and your cognitive load and bias is, is more.

00:11:49

And that can lead to errors, or it can be… It may not always lead, lead to errors, but it does add to the load and the, the, the, the bias lah. Sometimes, you just try to get the, the fastest, easiest diagnosis, and that will be the default, rather than putting in more, you know, like time and evaluating further and finding one or two other options. Just that whole situation of not… You know, of, of, um, yeah, that additional stress of lack of space and of overcrowding.

VRM11 Okay. Thank you very much. Um, I’m just going to move on to the next, uh, question. I think we have heard, uh, some of the circumstances that cognitive, uh, load, uh, would lead to cognitive error, um, and us having some biases. I just want to find out if anybody is able to share, uh, some real-life examples of cognitive errors leading to medical error. Um, maybe something more specific than what has been brought up earlier.

Um, for this, may I get participant number seven to just start sharing first? I’m not… We’re just wanting to hear from everybody, and that’s why I’m going to you, if you don’t mind, participant number seven.

PA7 Participant seven. Uh, as in what do you want me to share about?

VRM11 Uh, any real-life examples of, uh, cognitive errors that led to medical errors that you have experienced in your work, either from your colleagues or from yourself.

00:13:23

PA7 Okay. So, uh, let me try to think of one now. Wait ah.

VRM11 [Overtalking].

PA7 Can you ask somebody else first while I, I think of one? Yeah.

VRM11 Can. Um, anybody, uh, has something that they are ready to share?

PA5 Participant five.

VRM11 Please go ahead.

PA5 Yeah. So, uh, some of the examples, for example, uh, I have a patient who was being triaged as allergy, who claims to have taken some, uh, NSAID, uh, and then came in with a bit of hypotension. Uh, the, the… It’s very easy to put the error and bias to say that, yeah, this is true, the hypotension is due to the allergy, because it’s all labelled. There is some information and evidence, however it’s not, uh, the complete picture. It turns out, uh, if you do a full…

For that patient, I did a full assessment and found a triple A, and that was the reason that patient took the NSAID. It’s actually for back pain. Uh, and if the evaluation… If you do not start from scratch and from, uh, the basics and re-evaluate hypotension of all, all possible differentials, we will miss, uh, this particular diagnosis.

00:14:34

Um, that’s for cognitive bias. Another example is that, uh, where I have a patient who’s being brought to another area because the area was full. It was a lot of patients. Um, actually, because of cognitive load, they tend to push patient to another zone, and which is an inappropriate area. For example, like the Iso area. And then subsequently, patient collapse, uh, because the evaluation was not, uh, adequate. And that is an example of a cognitive load leading to error. Thank you. Over..

VRM11 Right. Thank you very much, participant five. I think that’s really, uh, very insightful. Uh, participant seven, I saw that you unmuted. You want to share?

PA7 Yeah. So, uh, one example I’d like to share is the… So it’s an example of either availability bias or recent case bias. So because, um, of, uh, multiple patients at resus, who are all quite similar.. Come in for shortness of breath, noncommunicative, or…

So we, uh… I, I tended to think then, this one was probably another case of, uh, pneumonia, but it turned out, actually, it wasn’t pneumonia in the end, because I, I wasn’t searching hard enough for the, the, uh, likely cause. Turned out that later actually the patient turned out to have intracranial haemorrhage, and the fever was, uh, just probably a red herring, and the, the crepitations that we heard were probably something that was long standing lah.

So it’s like it was only later we realised, actually, when you examine the patient properly, there was already… There was gait deviation to one side, which wasn’t picked up earlier, which was what led to the subsequent, uh, scan finding the massive ICH, which eventually led to the patient’s demise lah. Yeah.

00:16:21

But in, in the end, it wasn’t, uh… It was a bad outcome for the patient, but it didn’t lead to any repercussions per se ah, but it, it challenged my, my, my, my, uh, sense of that, eh, actually, I wasn’t doing right by the patient then. Yeah.

VRM11 Thank you for that very honest sharing, uh, participant number seven. Um, just want to welcome participant number nine, who just joined us. Um, just to keep, um, participation… Participant number seven up to date, we are just going through, um, some questions as part of the focus group discussion, and the purpose is really to understand, um, cognitive errors, and how, uh, they can affect us as emergency physicians. Okay?

Um, just a little bit, um, of things. Uh, when you are answering, just mention your participant number, and then start talking, so that it’s easier for the, um, data transcription later on, and also that this is being recorded to help with the data transcription. Okay? So, uh, I think that is the background. We will continue the conversation. And if you have anything, um, that you want to chime in, just, uh, feel free to. Uh, we also have a co-facilitator, uh, Dr Evelyn from SGH, uh, who’s here to just exist, and she may clarify further, um, if needed. Okay? All right.

So, um, we are currently on a topic of real-life examples of cognitive errors, and we have heard from participant, um, five and participant number seven. Anybody else want to share anything?

00:18:03

All right. If not, I think we are happy to move on to the next part. Um, before we go, uh, on further, um, since participant nine just joined us, maybe this is a good time to ask this question. Participant nine, do you mind, um, briefly sharing with us what is your understanding of cognitive error?

PA9 Okay. According to my understanding, I think it’s when you have too much of things, uh, of, uh, things to, uh, remember, where, uh, that’s where you tend to find… Uh, to have make mistake from there..

VRM11 Okay. Any, anything else you want to add?

PA9 Basically, that’s it.

VRM11 Okay. Thank you very much. I just want to find out, um, whether our other participants have any other thoughts on what cognitive error is. It can be the same as what participant number nine mentioned or something very different. Anybody want to share their thoughts on what they think is a cognitive error? Or everybody agrees?

Can I at least get an indication whether I think everybody agree or everybody, um, don’t agree with what, uh, participant number nine’s, uh, understanding is? Maybe I check with participant number eight. Um, do you agree with what participant number nine has said?

PA8 Uh, yes, I agree.

00:19:39

VRM11 Anything else you want to add or subtract from it?

PA8 Give me some time.

VRM11 Okay, don’t worry. Um, what about participant number seven? I’m just going to go one round, uh, just to be fair. Thank you.

PA7 Participant seven. Uh, what is the question again?

VRM11 Uh, can you briefly share with us what is your understanding of cognitive error? Yeah.

PA7 What’s my understanding of cognitive error.

VRM11 Mm.

PA7 Um, I, I can give examples lah, but, um, trying to… If you want me to give a definition of cognitive error, it’s… [Laughs]. I, I, I can’t, I can’t give a, I can’t give a textbook definition [overtalking].

VRM11 No need a textbook definition, but, uh, maybe just describe in a few words. If somebody ask you, what is cognitive error? How would you describe it?

PA7 It’s, it’s, um, an error from your thought process, which is not something that you intend to do so, but yeah.

00:20:54

VRM11 Okay.

PA7 [Laughs]. I’m not very good with the definition.

VRM11 No. No. But I think, I think that, that, that, uh, does translate, um, to some, um, of what you feel cognitive error is. So thank you very much for taking that. I think participant number six was asking, uh, participant number nine to repeat again. Do you mind repeating your answer again, participant number nine, so, uh, participant number six can, uh, potentially add on it?

PA9 Oh, sure. Sure. So I was saying when you have like too much information that you need to, uh, memorise, that will tend to cause error from there.

VRM11 Okay. Thank you very much. Um, participant [inaudible] want to… Have any, um, things to respond to that?

PA6 Yeah. For me, participant six, for me, cognitive errors are mistakes that we make, uh, when there’s too much going on in our mind lah, for various reasons..

VRM11 Okay. Anything else you want to add to that, or you are good? All right. Thank you very much, participant number six. Participant number five, um, maybe we’ll just hear from you before I move on.

PA5 Participant number five. Uh, agree with the definition given by various participants. Mostly, I think it’s just a mistake in the thought process, probably interjected by something from the external source or from the internal source that’s causing, uh, the eventual error. Over.

00:22:26

VRM11 All right. Thank you very much, everyone. I’m just going to share a little bit more about this.

PA6 Can I just ask participant five? Sorry. What do you… Uh, may you give some examples of internal source and external source for our understanding? Thank you.

PA5 Participant five. Uh, internal source is probably like your own amount of stress or your internal, uh, stereotyping, uh, things that you already internalise and you tend to put this, uh, label on the patient. External source, uh, is probably, uh, external stimulus. Like, for example, um, somebody said something, and, uh, you tend to use that, uh, anchor that, uh, that, uh, differential diagnosis to the patient. Over.

PA6 Okay, thank you.

VRM11 Okay. Thank you for that. I’m just going to move on to the next part to just share a little bit of what, uh, with you, cognitive errors are, and maybe that will help us to enter a little bit better for our subsequent, uh, discussion.

So cognitive shortcuts are often used to help us along our decision making and make a complex decision easier to manage. However, these shortcuts or heuristics can sometimes lead us astray and make us commit a grave cognitive error.

00:23:51

Cognitive errors are actually very common in clinical medicine, and everybody, regardless of seniority, is vulnerable to them. Moreover, they’re insidious and difficult to recognise, which makes overcoming them not so easy. So we really hope that today, we can work together with all of you to better understand what we can do to mitigate cognitive errors among, um, as emergency physicians.

Okay, with that background being laid, it’s very similar to what, um, you all have, um, mentioned, um, discussed to what your understanding is. Um, some, some of these questions may be a bit repetitive from our earlier exchange, but it’s really so that we can hear, um, and help us identify what are the main sources and responses that help to… Help us to reach saturation in our data. Okay?

So the next question I’m going to go into is, um, why do you think EP commit cognitive errors? You may have answered part of it previously, but feel free to just, um, share again. Okay? So the question is, why do you think EP commit cognitive errors?

PA5 Participant five. Uh, as mentioned, I think we all, all alluded to that, uh, when the load is heavy, we tend to use, uh, shortcuts lah, and these shortcuts, as I previous, uh, also felt, was actually something internal. Like we already have this particular model or whatever, uh, stereotype or, um, or shortcut that we feel that… Heuristics that we felt that this patient will probably fit into, and we use that to speed up our process because there was just too many patients. Uh, and because of that, uh, it's prone to cognitive error. . Over.

00:25:33

VRM11 Thank you, participant number five. Um, anybody else? If not, can I get participant number nine to just share on this? Because, um, we haven’t heard about, uh, this from your earlier, since you came in a bit later, and see whether you have any new points that the group is not aware of.

PA9 Okay. So I think, uh, load is definitely one thing, because we, uh… Once you have too many things to handle, you tend to take shortcut, and then tends to, uh, do whatever, as in jumping into so-called stereotyping bias lah. Then you will think that, you know, certain, uh, demographic of people, uh, will have certain presentation for a certain condition, and then you can… So you just narrow your mind..

VRM11 Okay. Thank you for that, participant number nine. Um, anybody else, uh, want to add anything on why do you think emergency physicians commit cognitive error? We’ve heard about the load. We’ve thought about anchoring. Any other reasons?

PA6 Hi. Participant six. I think it’s all related, but one specific thing is interruptions as well. So sometimes you have a thought process, and you are… As you are doing something else, your next process, thought comes to you, and you’re going to address that, and then you get interrupted, multiple interruptions, and then you forget about what that thing was that you’re going to do..

And that, um, may be something like checking the result of, of someone, or informing someone of something important regarding a test or a result, and then, you know, it leads to, um, you know, some clinical delays or unwanted outcomes. The interruptions is, is part of it. It’s of course all related to load, but it’s also part of what we do on a normal day as well, in a non-crazy day.

00:27:36

VRM11 That’s a very interesting perspective about interruptions that’s been just brought up. And thank you for that, participant number six. Um, anybody else got any, um, things to add? Participant number eight, I see you unmute.

PA8 Yes. Um, participant number eight. To add on to participant number six, um, points, right, about being distracted. So distractions can come in many forms, not only being interrupted, but it might be, um, the EP having a bad day. Maybe they’re feeling signs and symptoms of burnout. Uh, something is happening in their family, you know..

Um, so not only things happening at work, but what’s happening out of work as well, may affect, you know, um, thought processes of the EP, which can all lead to them having, um, interrupted thoughts and being unable to focus on a patient and give them the full attention. . Over.

VRM11 I think that’s also a very, very interesting perspective, whereby we now are looking beyond, um, our work environment, um, into the EP as an individual. So thanks for bringing that up, um, participant number eight. I just want to see whether the rest of the group have anything else that, um, they might want to share particularly with this, with regards to this interruption and these, um, these, um, personal baggage that an EP can come with. If it does resonate with you a little bit more, you want to share, please feel free, too.

00:29:01

PA6 Participant six. Actually, as, as participant nine or seven mentioned that, I was thinking that, uh… I mean, I was wondering… This is not the observation, but a question, um, whether EPs, do we generally need more time to decompress, uh, and reboot ourselves before our next shift, uh, to be optimal? Because I don’t think there’s any other… I mean, not, not many other disciplines where we go home with so much baggage, and we have so much going on in a day, um, at the kind of speed and constancy that we do at work.

So yeah, it’s just a thought that, you know, we, we, we bring home things that we need to, to deposit at the door, and, you know, we need time to decompress before we are functional at work the next day. Or sometimes not even the next day. Can be, you know, the same day, [laughs] later in the day.

VRM11 Yeah.

PA5 Participant five.

VRM11 Go ahead.

PA5 I feel then, uh, actually shift work also does contribute, uh, in terms of that baggage of distraction and internal, uh, stressors. For example, after a night shift, when next day morning, I’m definitely quite burnt out. It’s not a good idea to actually do a morning after a night so I… That’s my personal, uh, feel, and that makes me more prone to errors. Whereas if I do an evening shift or, or, uh, rest one day and do the next following day, probably might be better. Over.

00:30:41

VRM11 Thanks for that, participant number five and six. I definitely resonate with the [laughs] doing a morning shift after a night shift. It’s very challenging for me, too. But, um, participant six, I just want to say that you’re actually thinking ahead because, eventually, we will reach there about some strategies which you think might be helpful. So I’m going to park that in the parking lot first and, um, get back a little bit about that, um, distressing and unloading, um, as a strategy that may help us.

Before I move into that, um, anybody else wants to add on anything about why you think EP commit, uh, cognitive error? Okay, if not, I’m going to just, um, lead us to the next part of the discussion, which is a deeper dive, um, into our role as EP, and how we are, uh, responsible in recognising and, uh, overcoming cognitive error. Um, do you think this is… This recognising and overcoming cognitive error is something that is within the EP’s control, or it is actually more of a system level problem which the EP cannot control? So it’s really…

You know, we have talked about what cognitive errors are, and why we commit them. Now it’s really asking you, do you think it’s something that you can take control of as an EP, or it’s something that is really beyond your control? You just have to react to it, for instance. And what is your take on this? Anyone can get started.

00:32:13

PA6 Participant six. Is this EP in general or EP as in individual?

VRM11 Um, I think it’s EP both in general, like, um, should all EPs be able to do it, and then from there you can break it down based on if, if we do expect all EPs to do it, what’s your take on it as an individual? Do you think it’s something that’s manageable and obtainable? Yeah. Does that answer your question?

PA5 Participant five.

VRM11 Please go ahead.

PA5 Yeah. So I feel that it’s actually 50/50. Uh, 50 because the system actually does, uh, cause this problem because the load is due to shortages of manpower, is due to poor layout, uh, poor organisation of the, uh, EP shop floor. There’s actually, uh, there’s actually a lot of, uh, problems lah with the system, but… Which we cannot address, uh, at our level. The other 50% comes from internal. Like for example, I cannot do…

I mean, uh, if I had to do a morning after a night off, and that might be just particular to myself, that causes me to have, uh… Commit all this, uh, error as well. Uh, or, for example, if I, uh, if I… I’m not just saying for myself. I mean, in general, if, uh, if you, if you feel that, uh, things move on faster, you tend to create shortcuts, uh, and that’s actually sort of, uh, understandable lah from a human and individual perspective. Over.

00:33:51

VRM11 I definitely agree with you, participant five, that there’s definitely a lot of human component through it. Um, thanks for sharing that. Anybody else? Do you think, um, EP is responsible for recognising it, or it’s really something that is beyond EP’s control when it comes to, um, recognising and overcoming, uh, cognitive error?

PA9 Uh, participant nine. I think I do agree with participant five that, you know, there are certain factors that we cannot control and those, uh, that we can. So things like, um, load, and then like, you know, ambulance diversion, where one ED was overwhelmed because of, you know, this diversion. These are beyond the individual, right? Because the patient just come.

And, uh, so, uh, things that we can control. So depending on your personality also, because different EP will work differently. So, so some of us tend to go into very detail for a certain patient’s condition. Whereas some of us, you know, we do whatever that we think is suitable just at ED level, and then the rest, leave it to patient, so long patient doesn’t die on us. So it also depend on our, you know, appetite, risk appetite, uh, how… That will also affect how we handle the cognitive error.

VRM11 Okay. Okay. I, I hear you on that, participant number nine. Thanks for sharing that, um, perspective. Um, anybody else? Maybe I can get participant number eight to just share. What do you feel about this, um, aspect?

00:35:17

PA8 I think, um… Participant number eight here. So, uh, I think I just have one point, which is I just feel as I grow older, uh, in age, right? The ability to handle large, uh, large crowds seems to have diminished ah. The ability to also adjust to, um, sleep patterns also diminished. Uh, risk appetite also drops somewhat as well.

So I’m just not sure in terms of, uh, career longevity why… As one ages, you know, um, it just makes it very difficult. And I would think that as I grow older, unless my experience, um, grows in tandem as well, uh, such that it can actually make up for all, all my shortcomings, otherwise I think I will be very prone to making errors as well. Over.

VRM11 That’s actually a very interesting perspective coming in from, um, how we grow older in terms of our physical age, um, and its impact on our functions, uh, on the ground. And that’s really beyond the building experience part, because with time, we should, as you say, get more experience, and how it actually works in a different way. I really think that is very interesting. So thanks for bringing that up, uh, participant number eight. Um, participant number six, I saw that you unmuted yourself a little bit. Do you want to say something?

PA6 Uh, yes. Uh, can, can you just, uh, spell out the question again, please? What was exactly the question you asked?

VRM11 Exact question ah? Uh, yes it’s in the chatbox.

PA6 Do you think the EP is responsible for recognising and overcoming? Okay. So I feel that we are not, um, we are not able to recognise it as much as we would want to. Uh, are we responsible for it? I feel no.

00:37:12

If we are not able to, how are we responsible for it? Who has taught us how to recognise it, you know, uh, at, at every point, in every part of the day? So a lot of this is in the background, and we, and we, you know, we, we only see this in… You know, with the retrospect scope quite often.

Um, you may feel it. Because of your previous experiences and mistakes, you may feel that, eh, the situation is coming again. You know, it’s that kind of day. Um, and, and, and so you may know that today is a risky day, so then you have strategies that you create for yourself over time to deal with those kind of days, which may be to, you know, um, you know, slow down certain parts of your decision making so that you don’t make the mistake. Delegate or whatever, you know, your process is to deal with it.

But for us to, um… Wait ah. I’m going to your question. Recognise it as it’s happening or when it has just happened, sometimes it’s hard. Um, yeah. So overcoming it, I feel that it is… The responsibility of the system I feel is heavier than on the individual. I will say it is both, but the…

If, if, if, if the system improvements happen, right, um, it will help more lah than the individual improvements. So therefore the responsibility, uh, on the system is heavier. But can anything be done about the system? Over the years, I, I feel it is less and less. And so therefore the, the weight has to come onto the individual, which actually adds to on the stress that we have.

00:39:08

PA5 Participant five. Hello.

VRM11 Go ahead.

PA5 Yeah. Sorry, actually, I feel that even though the system does have, um, quite a big role and causing the loads to be high with the diversions and all this, but even on a day-to-day, in peacetime, there will be surges, uh, particular around mealtimes, and that is sort of a baseline, I feel, not during this crisis, and, and how the EP handles that, uh, depends on the… Some strategies lah, internal strategies.

And I feel like, for example, I know that, uh, triaging has a flaw, and I cannot fully trust on it, and that will also cause me or cause my colleagues to have bias. And I try to compensate that by, uh, looking through the triage again and trying to spot, uh, discrepancies, trying to re-triage, trying to go for the sicker patients first and try to have them be seen or up-triaged if necessary, and that’s one way, I feel, that can mitigate, uh, the cognitive errors. Over.

VRM11 Okay. Thank you very much for that, participant number five. I think number six wants, uh, to add on something. Over to you, number six.

PA6 Yep. Thank you. Um, yeah, so I, I, I totally agree. And, um, so we all develop strategies, whatever they may be, internal to us. So that’s how the EP copes. When the EP recognise just before it’s happening, probably not, I feel, um, but, um, but we are aware that we’re in that situation.

00:40:47

Regarding the baseline thing, I don’t know whether this answers this question. It may come up some other part of this talk, but because it was mentioned, I thought I would say that being able to deal with surges, uh, on a normal day is like, you know, you, you, you have a day, you go for a jog. You come back. You know, you run, you stop. You run, you eat. You know, you, you, you, you, you go up and down.

But when you have to go up and run the whole day until you go home and abandon everything else, then it’s no longer a relaxing, nice jog that you would like to do. It is, it is like, you know, like you’re in a wartime, you know, and that, that… The load on the mind, the stress, the catecholamines everything and, and, and how your body reacts is completely different, and you are far from being your optimal self.

VRM11 All right.

EW Can I ask a follow-up question? Um, so if you remove all this distraction, let’s say you’re on a very lull day, and there are hardly any patients coming in. Maybe Chinese New Year Eve, uh, everybody is having reunion dinner, something like that, and no… Hardly any patient come in. So do you think that, you know, this would… I mean, such a situation would reduce, uh, cognitive errors? So cognitive errors may be removed, um, when, you know, the situation, the system, uh, is, uh, less loaded.

PA6 For me, personally, participant six, for me, personally, I feel yes. Um, it is, it is a luxury when you have more time with each patient and when you can evaluate them yourself.

00:42:32

Sometimes, you just don’t have time to evaluate every single patient yourself. And, um, and usually, when I go and talk to a patient, I will find a little bit more than what, um, was shared at the presentation or some sort. So whether it’s, uh, it’s a, um, a social situation, or additional symptoms, or more related symptoms that may change my differential. It could be any of those.

But you, you, you, you, you will have more information, um, and a particularly more enjoyable, relaxing shift if you have less, less load, uh, around you, and, and you are also better able to deal with the surges when you have times of, you know, of peace.

VRM11 Right. Thanks for the additional, um, clarification. So, um, I think we do hear a little bit about, um, and get a little bit more understanding about, um, how you all feel, um, whether it’s more on the EP… Whether these, these, uh, cognitive errors are something within the EP control or something that’s beyond.

I think with that, um, I just want to move on to the next part of the discussion. Okay? And this is something that, um, is going to be interesting, because I think we do have, uh, EPs of varying seniority here. This question is really, what advice would you give to a new specialist about cognitive error, about cognitive errors that they should be aware of? I repeat again. What advice would you give a new specialist, cognitive errors that they should be aware of? Participant number seven, you unmuted. Do you want to say something?

00:44:13

PA7 Yes. So, uh, one of my pieces of advice I would like to give to new ACs is to first be aware that there are such things as cognitive bias, because if you don’t know what you don’t know, then you won’t even be aware of it lah. So knowing some examples of, uh, I’m sure having gone through Residency, they will have gone through, uh, PRLs, M&Ms. Maybe they’ll have, uh, made the mistakes themselves and find out what is it that actually cause those errors to happen.

So being, being aware is the first step. Then, uh, next is to, to try to reduce cognitive error, to, to always, uh, sound… Try to… If you’re unsure or if there’s a particularly complex case, to have a second, uh, opinion, second pair of eyes, or a second brain, you know, to discuss a case with, to go through what the, uh, the differentials are, the management, so that, you know, you can, uh, you can have another opinion.

And hopefully the other person doesn’t just, uh, echo what you say and is more like a devil’s advocate, who can tell you actually what you may be missing lah, because, uh, if the other person just parrots along, then both might be going down the, the wrong path lah. Yeah. That’s one of my advice I will give.

VRM11 Right. Thanks, participant number seven. I think that’s very sound advice. Um, anybody have any other advice that you may want to share with a new specialist?

00:46:00

PA9 Participant nine. Uh, I agree with participant seven. Then to add on, I think, I will advise them to… For, you know, every case that they see, treat them as they are taking like, you know, the case that come up in their viva, okay, where they will think, you know, laterally, think of the worst thing, things like that, and so that, you know, they don’t fall into the comfort zone of taking shortcuts. Yeah.

VRM11 Okay. Thank you for that, uh, participant number nine. It’s really just to keep that broad-based approach still, um, there, even though exits is over already. Um, participant number eight, I think you unmuted. Please go ahead.

PA8 Participant number eight. Just to echo some of the other participants’ points. Um, it’s okay when you are stumped, uh, to ask for a second take, because two eyes are better than one, and especially from the eyes of someone who’s more experienced.

Uh, number two. Okay, it’s also important for the EP to know their team members’ capabilities. So not only the junior doctors, but also the nurses as well, especially if you’re working resus, uh, so that you know, number one, when to compensate, when to overcompensate, um, as well as you know how much, um, entrustment you can place in your junior doctors, um, especially when they may range from very, very seasoned, very, very junior HO turn MOs, um, whom you probably need to review all of their cases, especially for the first few weeks of their posting.

Number three. Uh, again, ask for help when they are overwhelmed. I don’t think it’s… I think it’s quite understated lah, but we don’t ask for help enough, especially in the Asian context, where asking for help may seem like weakness..

00:47:45

But sometimes, when you are already overwhelmed, the doctors are overwhelmed, the nurses are overwhelmed, it’s okay to just ask for help so that other areas can send help over. And lastly, uh, if the shift is really, really very busy, uh, and you don’t have enough time, okay, uh, at least eyeball the patient, okay, because at least you know, based on your instinct, based on your gut feel, based on your years of training, you have an idea of how sick the patient is. Over.

VRM11 Right, thank you, participant number eight. Participant number seven, you want to say something?

PA7 Okay. Uh, participant seven. So I’d like to add to what, uh, participant eight had mentioned about asking for help. To add on to that, in order to ask for help, there must first be a culture of, of like being able to get help when you need to.

So it should start with, uh, yourself, and if you proactively help other, uh… Your colleagues… Like maybe you see that, eh, resus, there are multiple patients, or you suddenly see a patient who’s intubated, and no one has actually typed out anything, and you think, how come no one is like asking for help? Then, uh, I, I think it’s good to be proactive to extend your help, because then that helps to create a culture. And hopefully, with this culture, the other party might do likewise for you in the future when you need help yourself..

00:49:14

VRM11 Right. I think that’s a very nice support system and, uh, culture that you are… Point that you have brought up. I think that’s… I really appreciate that. Um, any other, um, advise that you might think will be relevant to share with new specialists about cognitive errors?

PA5 Participant five. Uh, I feel that, uh, don’t, don’t trust, uh, what was handed over 100%. Uh, especially handovers, need to re-evaluate from the start lah. . Yeah. Over.

VRM11 Okay. Thank you very much. I think there’s also a thing about handover that’s been brought up. So, uh, any other things?

PA6 Hi. Participant six.

VRM11 Please go ahead.

PA6 I, I agree with everything that’s been said. In addition, I, I would also have a bit of a warning. I don’t know whether it’s advice or warning. Somewhere in between maybe. Is that because they are fresh and they have learnt a whole lot of work and just passed exam, right? There’s a lot of, uh, hero complex. You know, like you, you, you, you know, you think you’re very good at everything now.

And, um, you need to be aware of, um, the fact that we are all going to make mistakes. It’s just a matter of time. And, um, you know, um, what they call it? You know, have, have that humility lah to understand that, um, people make mistakes, and everybody will make mistakes at some point in time. It’s just a matter of when..

00:50:41

So, um, don’t be so like, you know, uh, I know everything, and everything I do is right, because, um, definitely, [inaudible] you won’t feel that way. Something like that, but with a better spin on it lah. I’m just off the top of my head now. Because I feel that over the years, you know, when give advice, um, sometimes advice is taken wrongly, and advice is, um, looked at… Looking upon as weakness.

And, um, and part of it is because, um, they are, they are at the point in their career where although, internally, they may have fears, uh, and don’t want to ask or, you know, fears about being able to handle the worst kind of patients or whatever it is, um, there is a lot of overlap of, um, the high feeling. Um, and, and that, over the years, will, will be, will be, uh, adjusted down lah with, with experiences, yeah, and other things will be, will be adjusted up..

So, um, so sometimes the, the advice that needs to be given, for it to be heard, right, it needs to be the right timing, um, and sometimes I feel right after exams is not the right time.

VRM11 Okay. Thanks for sharing that. I think the element of timing about when this advice should be given out is also useful. I think the reason why this question is there is because we want to lead on to the next part. It’s really about, um, asking you all two things. First is, do you think education regarding cognitive errors is necessary? And number two, if you think it is necessary, when should it start? Okay, so number one, do you think education regarding cognitive error is necessary? Number two, when should it start? Um, so anybody?

00:52:44

PA9 Participant nine. Uh, I think it’s necessary. I think it’s good to, uh, remind people, uh, to tell them, you know, there is such a thing going on. It’s not, you know, whose fault and point fingers and all that. But we must be able to recognise it, and I think you should start very early. In fact, as early as you are in the medical school. That’s where you should, uh, have this teaching, this education, so that, you know, it will be, it will be, uh, culture. As in it will be brought forward, and then when you become a junior doctor, then you become a specialist. So you must start early.

VRM11 Okay, I’m hearing from participant number nine it being necessary and should start early at medical school. Anybody else have a different take on that, or you all feel the same?

PA8 Uh, participant number eight. Uh, yes, I agree. Um, and, um, I feel that the maximal impact probably would be the time when they have had some working experience, because, uh, I just feel as a student, it’s important to learn the concepts, but as you work, then you probably would be better able to articulate the idea of cognitive errors when you’re on the shop floor with a lot of stressors happening around you, and then you can learn from your experiences..

Because, uh, the medical students do not have that experiential learning per se, short of their SIPs, um, as well as their medical postings, uh, attachments. So I think, um, most probably, as a, as a junior doctor, that’s when it should be focused on, so that they carry that culture, like what participant number nine correctly pointed out.

00:54:18

Um, and maybe, maybe at the end of each, um, PRL, for example, because, um, mistakes… Sometimes there, there are errors that has happened, and, and you just run through again, just reinforce that, hey, this, this, this, this, this, this, uh, you know, um, cognitive error happened, so let’s just be mindful for future sessions, so on and so forth. Uh, over.

VRM11 Thank you for that. Anybody else got anything to share about this, um, education, and when should it start? Okay, if not…

PA5 Participant…

VRM11 Go ahead, participant five.

PA5 Participant five. Uh, I feel that it should start before they start their senior resident, after their junior residency. And they need a bit of example and a bit of a feel of how is it like to go through a senior kind of position to feel that they actually might be prone to, uh, cognitive bias. And I feel that it’s, uh, important to be included lah, but they need to have a bit of a foundation to start off. Over.

VRM11 Okay, we are hearing a different layer, and I’m actually quite interested in that, because, um, we, we do hear that, you know, it’s, it’s important. We should start early in the medical.

00:55:39

But I think, um, what participant number five just brought up is the layer of context. Like, you know, maybe, um, medical school, just know that it exists. Subsequently, when they go into residency, it’s more specific to the ED setting. And I think what, um, participant number five allude to is, you know, um, at the senior residency, you’re now in a senior position, and the cognitive errors that they might be exposed to might be very different.

Um, anybody have anything to add to this, that was being shared? Okay, if not, I think I’m going to move on to some ending questions, uh, very quickly. Um, just now, we put the strategies, um, in the parking lot. Now I’m coming back to them again. Um, participant six earlier mentioned that, um, strategies like, uh, knowing how to, uh, unload yourself at the end of the day, um, and get refreshed, um, are important.

So I’m just going to ask everyone, what strategies do you personally use to help you mitigate and prevent cognitive errors? Okay, and these errors, uh, I think, earlier on, just to recap and refresh everybody’s mind, we say that some of these errors stem from the individual. Some of these, uh, stems from the system. So what strategies would you personally use to help you mitigate, prevent cognitive errors? Anybody can start.

PA5 Participant five.

PA7 Participant… Oh. Uh, never mind. Participant five, go ahead.

00:57:22

PA5 Yeah. Uh, I feel that, actually, first of all, need to have insight. Need to acknowledge that, that, that this exists, uh, in terms of training, in terms of, uh, being on the, on the ground, to know that when you need to watch out for these things happening, especially when the load is heavy. In situations like that, need to be particularly careful.

Um, and I think, uh, another strategy is probably to compensate a little. For example, if I know that, uh, that, uh, it’s a Monday, the shift is going to be busy, need to, um, be, um… Yeah, so I, I will need to, uh, rest up. Uh, have adequate rest. And also maybe, uh, try to ask for more help. . Over.

VRM11 All right, thank you very much. Um, I think number seven, you wanted to say something.

PA7 Yeah. Participant seven. So, uh, so on top of, uh, what I said earlier in the interview about asking for, uh, help, asking for a sounding board, it’s to also to when evaluating a patient, be it a simple or complex patient, to always routinely have, uh, differentials.

It might, you know, seem really clear cut or like, oh, it’s just another case of, uh, pneumonia or gastritis, but, uh, I, I would say that we always should try hard to think of, uh, differentials, and, uh, what are the dangerous differentials, and always attempt to disconfirm for those dangerous differentials, either through your history or through your examination or your investigations, because, uh, that, that, that will force you to, you know, not to, to tick off like what is the, the, the easiest, uh, diagnosis. So that’s one strategy.

00:59:19

VRM11 Thanks for that, participant number seven. Uh, I think earlier on, I also saw participant number six, uh, unmuting. You want to share something?

PA2 Hi. Yep. Um, participant six. So I feel that, okay, so, um, specific to what we do at work lah, I’ll answer in that way. Um, I, I work on the element of distrust, let’s put it that way. So when the story is being told to you, you, you listen. You take what they’re saying, but you have to… I mean, I lah. I have to like make sure I didn’t miss any clues that were out there, that may have been overlooked.

So I will always look at the vital signs, uh, every component, and see if that fits the story. Look at the triage note again. Um, and if there’s, you know, a medical history, we will see whether there’s anything there, because the, uh, the assumption, there’s a lot of assumptions happening, you know, when junior doctors are rushing, and they’re always rushing.

Uh, so if there’s pneumonia… If there’s heart failure before most SOBs, they will think a heart failure, you know, that sort of… So we have to really look for all the clues that were there to say that maybe it’s not. So we always work with differentials on my mind, even if our juniors do not, and, um, we look for the clues… We scrub for the clues, I would say. And that last part of the scrub is being able to talk to the patient lah, which you can, good [laughs], but it’s sometimes very difficult. Yeah.

01:00:54

VRM11 Right. Thank you very much for that, participant number six. I think, uh, we have yet to hear on participant number eight and nine on this. So I think it’d be nice to get everybody’s perspective about, uh, strategies, so we can see what we can… Uh, what are some of the recommendations we can put forward. So over to you, participant number eight.

PA8 Yeah. So participant number eight, um, over here. So I personally practice or at least try to practice mindfulness on a regular basis, and I feel that it helps with my ability to focus without being distracted. So as alluded earlier, uh, distractions help… Makes us very… Makes it very difficult for us to focus with a clear line of thought.

So by practicing mindfulness, I feel I’m better able to, uh, maintain, maintain clarity as well as focus on the patients under, under my care, without being unnecessarily distracted by my own thoughts, by my own feelings, or by the other people around me. Over.

VRM11 Thank you very much. Uh, over to you, participant number nine.

PA9 Yeah, participant nine. I was about to say something very similar, but mine is like, uh, doing self-check. So basically, it’s like, you know, despite the load, despite all this, always maintain the, so-called, the… Your own internal peace.

Uh, you know, with such a heavy load, and then the, the team will be overload, and then they will always come down and want you to change discipline and things like that. Uh, as much I want to entertain them, I will also stick to… You know, be firm, uh, and, and, and, and, uh, will ensure that I not be… Not to be too accommodating, but yet not to be too angry with things and, and… So that at least I may get my own inner peace, so that I will not bring the emotion over to the next patient or the next, uh, case.

01:02:45

VRM11 Great. Thank you very much, um, for sharing your strategies. Um, at this point in time, I just want to check with my co-facilitator whether she has anything else, uh, that she want to bring up or clarify.

EW Um, yeah, I just want to ask you guys. Uh, is there anything that can be done in the system’s perspective, you know, such that cognitive errors can be eliminated? Or is it not possible to have a, you know, complete, uh, removal of, uh, cognitive errors?

PA9 Participant nine. I would say it’s not possible to eliminate totally, but there is definitely room to reduce it, uh, to, you know, to, to control it. Uh, one good thing as in to start with is definitely manpower. So, you know, such a busy load and things like that, and we’re always short of manpower, and that will actually… It’s the main factor that, that affect, uh… That link to cognitive load.

EW Okay. Does anybody else want to add, uh, or share? So even though if we may make the system better, but, uh, cognitive errors may still happen, or…? And if we were to, uh, make sure that the manpower and other, uh, external resources are available, um, you know, to… Um, then to what extent do you think that, uh, cognitive errors can be controlled? Anybody want to share their thoughts?

01:04:47

PA6 You mean if manpower is optimal, is it?

EW If manpower, all these external things that have been said lah, um, surges and manpower and space, whatever, uh, you know, are dealt with.

PA6 Oh, I think you’ll make a significant impact on our, on our error… Cognitive errors. Uh, at least, at least 40%, 50% reduction, because we are… We go from wartime to peacetime. And in peacetime, we think better, do better. We find things we didn’t expect to find.

EW Okay, thank you.

PA5 Participant five. I feel that, uh, even with… Even though system may improve, uh, during peacetime, I feel that sometimes that, like what, uh, participant nine said, the error cannot be completely eliminated, although it can be reduced. Uh, but there are also situations where I noticed that when the load is relatively light, uh, patients still do not get, uh, adequately evaluated if, if that internal bias still exist, that cognitive bias, uh, and especially if you just want to get the patient, uh, out of the dispo, you know.

And sometimes, I, uh, I even notice like, sometimes, uh, seniors may go, or, or juniors may go around, uh, chatting or, or drinking coffee, rather than, uh, spending the extra time with the patient lah. I mean, it’s really on, on an individual case by case, not really, uh, saying everyone does this. Over.

01:06:37

EW Does anybody else, uh, have… Uh, resonate with this or, you know, who would like to share more about this aspect that has just been shared? Which I hear is, uh, you know, even when, you know, um, it’s not so busy, uh, the, you know, the clinicians, you know, may still not, um, spend that time, uh, evaluating the patient, and, you know, that may or may not lead to any errors.

PA7 Participant seven. I think it’s a fine balance, because, um, I, I recognise that during a shift, I only have that much cognitive bandwidth. To, to me, that’s like my health bar. So like, you know, during, um… When it’s not so busy, I [unclear] maybe, if I’m fortunate enough, maybe the first hour or so, if I am, uh, able to preserve my, uh, my health bar, I like to save it for when I anticipate it’s going to be really bad.

So I, I load up on like, um, on, on being able to, to, to do the not so cognitively taxing things, like maybe acknowledging results or whatever. And then later on, when I know that it’s going to be bad, I’m already mentally prepared, and I later have bandwidth, cognitive bandwidth, to spare for later. I mean, that’s my personal strategy lah. Not sure if it resonates with the other participants. Over.

PA5 Participant five. Uh, I agree with participant seven in that because towards the end of the shift, the mind tends to, uh, come to termination, and that, that is an inevitable outcome lah that, uh, cognitive bias will tend to happen towards the tail end of the shift, when… Especially you know that you’re going to hand over to someone that, uh, patient is not fully evaluated. Over.

01:08:53

VRM11 Okay. Thanks for that. Uh, I’m just going to go one round, um, to everyone to see whether you have anything else you want to bring up, um, before closing the session, because we also want to be cognisant of the time. Um, I will just start with participant number five. Um, anything else you want to bring up, um, that you think you have not mentioned?

PA5 Participant five. Uh, that will be all. Over.

VRM11 Thank you very much. Over to participant six. Do you have anything else you want to bring up?

PA6 No, nothing.

VRM11 Thank you. Participant number seven.

PA7 Participant seven. Nothing further to add.

VRM11 Thank you. Participant number eight.

PA8 Participant number eight. Nothing further to add. Over.

VRM11 Thank you. Participant number nine.

PA9 Uh, just one last thing. I think, sometimes, uh, we also must be careful. Like certain things that we do when trying to reduce cognitive load and error, we may actually feed into the whole system of a vicious cycle. Say, for example, you know, with IT and all that technology.

01:09:48

We are quite, um, familiar that, you know, all the, all the drug allergy, things like all this check, check, check thing, multiple clicks, right? It’s supposed to reduce this cognitive error, that, you know, so that at least we are more careful. We, we look into it more carefully before we do some, uh, submission for the medication, or even, you know, one ICM will close if you’re going to move one patient, then next patient.

But that reduce our, um… Slow, slow, slow us down, and that actually will also make us less, less efficient, and that actually may also fit into our cognitive error. Yeah. So it’s like, you know, what is a better solution here?

VRM11 Right. Thanks, uh, participant nine. I think good to just remind us as well, we are looking for solutions. We are hopefully not in the, um, spirit to generate even more problems downstream. Yeah. I think, um, that’s all that we have to go through for this, um, focus group discussion. Um, I just want to thank everyone, um, very much once again for your time and your, uh, open sharing. It’s really very, um, rich, and I think we’ve got a lot of materials that we can look into.

Once again, um, I just want to reassure you that, um, your responses will be kept strictly confidential and only used for the purpose for the… Of the research, and, um, any, um, information used will be, um, deidentified. Okay, um, I just want to remind participant, uh, that has not sent me your consent form, to send it to me. I’ve sent it to your work, work email. So, um, you can assess it at work. And if you need me to send it to you in your personal email to fill it up, um, I can also do that. So just drop me a message.

01:11:28

Okay, um, I think that’s the end of this, um, focus group discussion. I’m going to stop recording and end the session here. Thank you, everyone, and have a good day ahead.

PA9 Thank you.

PA5 Thank you.

PA7 Thank you.

01:11:41
