# Supplementary material for: Dropping the baton: Cognitive biases in emergency physicians
Source: PLoS One. 2025 Jan 2;20(1):e0316361. doi: 10.1371/journal.pone.0316361 (PMC11694980; doi:10.1371/journal.pone.0316361)
Supplement: S3 File — (ZIP) [file pone.0316361.s003.zip › Transcripts/FGD 3.docx]

FGD 3

Speaker Key:

SI Sim

CF Co-facilitator

PA Participant

00:00:00

SI Okay. Yeah, so I, I know that some of you had concerns about participating in this group. So I’m really thankful and appreciative, and I hope to reassure you that, um, it’s going to be a safe environment. And then, uh, everything that you say is going to be confidential. It’s not going to be used in any way against you.

Um, so I’m just going to start by, by saying that so we’re a group of emergency physicians, uh, four of us, and we’re using qualitative methods to learn about cognitive factors that lead to, uh, emergency physicians specifically, uh, committing errors. So we understand that, um, cognitive errors can potentially be a very sensitive topic. We sincerely hope that you can contribute freely and honestly.

Um, we need to maintain anonymity, given the sensitive nature of the topic. So, um, thanks for changing your display names. So avoid revealing your personal identity, uh, in the course of the discussion and refer to yourself by your designated participant, uh, ID instead. So before you start talking, can you say, I’m participant ten, or I’m participant 12?

00:01:17

Yeah. Um, we seek your understanding that we are going to record this discussion, um, for purpose of research, uh, to maintain the accuracy of the transcription and data collection process. Um, but as I said, please be assured this is a safe environment, and your responses will be kept confidential and anonymised. All information will be used only for the purpose of the study, and your, your, your seniors, your bosses and HODs will not be privy to the data collected, and it will also not affect your performance appraisals.

Um, lastly, we ask that you… Everybody be respectful and treat everyone as equals. Um, because it is a Zoom meeting, we need to take turns to talk and wait for each other to finish and not talk over one another. So, uh, yeah, indicate your participant number before you start speaking.

Um, in view of the time constraints and to give everyone opportunities to share, we may have to stop you, um, during the conversation, but we will come, come back to you, definitely, if the time permits. So as you can see, we have three other people… Four participants, and then we have two other… Three other people in the room. So, um, Jenheng is our, uh, co-facilitator, and there are two other, uh, research observers. Okay, so they will be assisting me to do any clarifications, um, if necessary.

00:02:49

So, uh, as a start, just want to ask the participants, um, how long have you been working as an emergency physician? That means from, uh, as a specialist. Any of the participants want to start? Uh, can tell me like, uh, in range lah, like less than five years, five to ten years, more than ten years.

PA11 Uh, about eight years. Eight to nine years.

PA12 Um, more than ten years.

PA13 More than ten years.

SI Okay. Participant ten? Counting.

PA10 Yeah, counting.

SI Agar-agar can already.

PA10 Uh, based on participant 11, then, uh, seven years. Six, seven years.

SI Okay. Uh, I think there’s a question about using the chat. Uh, you can. Uh, but for the questions that we are going to, uh, ask and discuss, uh, probably better to vocalise it. Yeah. But yes, we will also keep the, we will also keep the, keep the chat open, and we will try and make sure that we answer all the, all the comments inside.

00:04:16

Okay, so for the next question, just wanted to ask about medical errors. So think about some of the medical errors that you or other people have, uh, committed as specialist emergency physicians. Um, what are some common factors or circumstances do you think that lead to medical errors as a specialist? What are some common factors that lead a specialist, uh, emergency physician to commit errors? Medical errors.

PA10 Uh, participant ten. Can I just clarify the scope of medical errors?

SI Medical errors, um…

PA10 Why, why, why, sorry, why I have the confusion is because, uh, right now, there’s a lot of emphasis on, uh, things like mislabelling and all that. That’s also considered, is it, or…?

SI Yes. So like anything like medication errors, or, let’s say, diagnostic, uh, errors, or errors in management. Any part of that patient care journey.

PA10 So in general lah.

SI Yes.

PA10 Okay. Uh, I will, I will offer two. Uh, fatigue and multitasking.

SI Okay.

PA11 Participant 11. Anchoring bias.

00:05:47

SI Anchoring. What do you mean by, what do you… Can you just let us know a bit more about this anchoring bias?

PA11 Like, um, just depending on, uh, pattern recognition or, or initial assessment and, uh, past experience to determine what you think the patient has and, uh, sticking to that diagnosis and not really, um, wanting to commit to a change of diagnosis subsequently, either for convenience or because of the bias. . Yeah.

SI Ah, okay. Great.

PA12 Uh, participant 12. Uh, manpower issues. And when we try to do things, uh, on a haste, you know, uh, it can actually lead to errors.

PA11 Uh.

SI Participant 13, anything to add?

PA11 IT issues.

SI IT issues.

PA11 Um, not familiarity with, uh, everchanging, uh, IT need in a department, leading to delay in treatment, uh, wrong treatment.

SI Mm. Okay. Uh, participant 13, anything to add?

00:07:28

PA13 No, not really. I think it has been… I think multitasking is a big thing, especially in ED. Um, when there’s too much on our plates and try and do multiple things at the same time lah, uh, that can be… Can contribute to errors and… Yeah. Yeah. Especially nowadays, when the manpower issue [laughs], uh, is, is exacerbated, and we’re taking on even more tasks, beyond what a doctor is to do.

SI Yeah. Okay, agree, agree. So all very important factors. And I think you all… I mean, all of you have touched on, on very pertinent and timely issues in the ED. Um, the, the next question is actually a little bit more, um, probing about what you think, um… What do you… What’s your understanding of cognitive errors? So when somebody says, cognitive errors, what is your understanding? What is, what, what is cognitive errors to you?

PA13 Participant 13. So similar to what, um, I think ten said, right, or 11, I cannot remember [laughs], about anchoring bias, uh, yeah, when you’re anchored to a diagnosis, that’s an example of cognitive bias loh. Yeah.

PA11 Oh. To add to that, um, basically, to base your decisions based on, uh, past bad experiences, or past experiences, yeah lah, experiences that burnt you, or, or things like, I always do it this way, so I will continue to do it this way, uh, rather than, um, to actually embrace evidence..

PA12 Participant 12. Yeah. Uh, I have a similar idea. Basically, we have pre-formed, uh, perception or something, or knowledge on something, and then which might affect our decision making lah, eventually.

00:09:40

SI Okay.

PA10 Participant ten. Um, I look at it in… Cognitive bias just means brain problem. So it’s really about the person’s thinking. So whatever factors that have, uh, led to that person making, uh… It’s a thought error. Error of thought that, um… What do you say? Like you, um… It makes you make the wrong decisions in, uh, it could be, in retrospect. Actually, if, uh, if you recognise… If you look back, you recognise the error, and then you realise that you will have made a different decision, uh, whether… Yeah, and, and, and, and, uh, that, that will be… That will have been better lah.

So, uh, I think collecting everyone’s, uh, input, uh, I think we talk a lot about anchoring and confirmation because that happens more often, but, uh, I think there are more, uh, biases and errors that we can commit, and we could probably divide them into, uh, pre, uh, during, and post.

Essentially, uh, pre will be… Just now, you mentioned a lot of pre-formed ideas, pre-formed perceptions, pre-formed culture, pre-formed, uh, bad experiences that, that influence us. Then during would be whatever stressors that, uh, nudge us or make us, uh, think in a different way from what, uh, will have been better, would have been ideal.

And then, uh, post will be something like, uh, outcome bias, which will influence the next decision. So like you, you, you did… You chose to do A, and then, uh, patient deteriorated, and then next time, wah, should I do A again or not? Because A was so…

00:11:34

A is correct. I think it’s the correct thing. But now the patient deteriorate, so… And then bad outcome. So should I have done otherwise, although my… Uh, just now participant 11 talk about evidence. Although my evidence and all that says that 1,000 people out of 1,000 people, they will all benefit from A, but I do A, and then the patient had bad outcome, so next time I should maybe don’t do A anymore kind of thing.

SI Okay. That sounds… That’s, that’s very clear. Thank you. Um, [inaudible].

PA13 Um, participant 13. So I think the alternative is also a confirmation bias. When the MO comes and tells you, this is the diagnosis, and then we kind of are anchored onto the diagnosis, and, yeah, and sometimes it can be totally wrong, but we never go and think about other things because we are already down that line lah. It can happen between specialists or anybody else. Yeah.

SI Okay. So thanks, everyone, for sharing their perspective. Um, we would like to share that, um… So when we make decisions, uh, we think and consider various options. We weigh the various pros and cons before committing to a decision. So this thinking process is our cognition in action. So sometimes if, let’s say, we’re lacking information, or we perhaps have the wrong perception, or we are taking shortcuts, uh, it can lead us astray, and we can make a skewed decision.

00:13:14

So this results in cognitive error. And actually, cognitive errors are very common in clinical medicine, and everyone in the healthcare, regardless of seniority, is vulnerable. Um, they are insidious. They are difficult to recognise sometimes, and it makes overcoming them, uh, difficult. So we hope that we can work together with you to find out and better understand, uh, cognitive errors, and what we can do to mitigate, uh, errors, especially in the emergency department.

So the next question, um, that we’re going to discuss is how, how much or to what extent do you think cognitive errors play a part, uh, in EPs committing medical errors. So when an emergency physician commits an error, to what extent do you think, uh, cognition, cognitive errors play a part? So how much is cognitive? How much is, uh, other factors? So you might want to think about some real-life examples or past experiences for yourself or even for other people. Uh, emergency physicians. Yes, participant 12, you unmuted.

PA12 Uh, if you ask for percentage-wise, it’s, um, it’s probably low. Yeah. Um, probably about 5% or less. Yep.

SI Okay. Why, why do you, why do you say that? Would you like to…

PA12 Um.

SI Explore that a bit more?

00:15:13

PA12 I mean, because, um, you know, on the ground, when we, when we see, um, errors happening, uh, when you contribute to, uh, you know… When you, when you note down the factors which contributes, um, you know, sometimes it’s other factors which affects, uh, you know, uh, the error, rather than, um, you know, not too much of cognitive, um, you know, bias, you know. I don’t know. That’s what I feel. Yeah.

SI Yeah. What are the, what… What do you think are the other… So you said 5%, right?

PA12 Mm. Mm.

SI [Overtalking] 5%. What, what, what are the rest of the factors, the non-cognitive factors that you think… What’s [overtalking] factor [overtalking]?

PA12 Like, like things that was mentioned here just now, you know. Um, you know, multitasking, you know, um, IT issues, or, you know, um, technical issues. Okay, manpower crunch. I mean, I think everything as, you know, as a whole, um, contributes as well. So I feel that there might be majority of it.

SI Okay. Yep, thanks for clarifying. Anybody else wants to chip in?

PA13 Can I clarify? So we’re saying that multitasking, everything doesn’t contribute to cognitive error, or are we saying it’s part of cognitive error as well? Because it is right. Because errors, I mean, generally, it’s whether it’s a true knowledge or technical skills problem, or it’s, uh, cognitive error, or it’s a systems-based problem, right, which is all… Which is your manpower shortage, IT shortage and everything. So are we putting multitasking as a contribution to cognitive error and a systems… And also a contribution to systems-based problem? Yeah. Yeah.

00:17:17

SI I think that’s why the… That’s why discussion is so rich, because then you see that everybody has a different, um, view. So that’s why we also want to understand, uh… We want you guys to, to talk and discuss about this. And, and so with… Even like with participant 12, and between participant 12 and 13, there’s a little bit of difference already. But not to say that, you know, anybody is right or wrong, uh, but how you view your… What your understanding of cognitive errors is, and then how it pertains to, uh, the factors that contribute to error.

So like participant 13 said, if, let’s say, manpower. You feel that, you know, when there’s a manpower issue, or if there’s an IT issue, and it contributes to your cognitive error, then you would count that in, then that also… That is also a rational… It’s, it’s a very reasonable, uh, argument.

CF2 Hi. This is cofacilitator 2 jumping in here to expand on this a little bit. Um, I think we want to find out how multitasking, for instance, affect the way you think. It is, uh, it is a contributor, but how does multitasking affect the way you think such that you commit an error? Is it that, that you’re multitasking with less time, you don’t analyse as deep, and therefore you go by the first instinct, and that’s why the error happened? Or is it another reason?

So the same contributor, like multitasking, can people… Uh, different people committing errors differently in the way they think. And I think this is where you want to tease out.

00:19:02

Um, that’s why, uh, if the participants can share a little bit more in detail about how their thinking is affected by all these processes, I think it’d be really helpful. Thank you.

SI Yeah.

PA10 Uh, ten, ten, ten here. Ten here. Just, uh, to talk about that, that part, uh, you mentioned about 12 and 13. So, um… And, and just to slightly answer co-facilitator two. So the thing about, um… Let’s just take multitasking for example. So it, it just adds, uh, what we call cognitive load, right? I think we’re all very familiar with that term.

So, uh, when we have to multitask, then there are many things that, uh, load our brain, and then… And when our brain hits a certain load, we don’t, we don’t automatically make errors, but we try to take shortcuts. We try to short-circuit the process. We try to, uh, fit things into protocols so that I can move on to my next task. All these are what you call temptations lah to do so.

We, we don’t make errors outright, but we, uh, start to exhibit what, what I would call high-risk cognitive behaviour, where you will try to, uh, minimise the time needed for a task. And, uh, I also look at multitasking in two ways. Uh, one is multitasking, uh, by choice. Another is multitasking, uh… Coerced multitasking. Meaning, um, you are, you…

00:20:33

Multitasking by choice means I come to work, okay, I need to do this, do three things, and then I go and choose to do them together. That is multitasking by choice. Multitasking, uh, by coercion is I’m trying to log on to my computer because I… Okay, so for example, just, uh, amalgamate a scenario. So like I have a… I, I log on to my computer. Then I go and see a patient. I talk to the patient. I listen to the lungs. Then I come back. I want to order things. Then my IT system is logged off. So now I must log on again.

Now I log on, I log on. It takes two minutes to log on, as the timer keeps spinning. Then I, I finally log on. Between those… In those two minutes, I have down-triage two patients. I have, uh, maybe vetted a case with my MO. And then now I’m log on. And then, uh, I try to click ABC patient’s name so that I can order something. Then in between that, uh, someone tell me that this one… This other patient’s normal saline was not given.

So I have to… Okay, then that’s where I click DEF, and then I start ordering my bloods. I order the blood for the wrong person because now I’m at normal saline, and then I go and click something else.

Uh, or more common is I go to the correct patient A, B, C, and then why am I log on here ah? I’m supposed to do something, but I forgot. Yeah, that is more common than, uh… Yeah. Or maybe I gave some medicine, and then I’m supposed to chart it. Of course I’m supposed to chart it and then give lah, you know, like…

00:22:16

But sometimes you have to give the medicine, uh, expeditiously, and then, yeah, now I’m trying to chart. Oh, uh, okay, why am I here? Yeah, it’s, it’s a very common thing. Why am I here? I log on, right? Cannot remember what I’m doing. Or, or did I want to order something? What blood test did I want to order? Yeah, then it will be error of omission because I then forget to order ketones, etc. So that answers this part.

Uh, the, the, the… Just to answer, uh, the facilitator’s question about, uh, thinking about how many percent of your errors are cognitive. I think that process is also fraught with bias, because, uh, there is recall bias, because, uh, you… When you ask me to think about things that happen in the past, uh, there is a huge chunk of undiscovered cognitive errors that we got away with.

Uh, so tie that with outcome bias lah, that, that those errors were of low consequence, and therefore, oops, no one found out. Great. Oh, I didn’t even know. No one tell me. Because hand over next, next team or admitted, so no one will tell me that. So those are… So I, I feel that whatever number we come up with, the, the number is actually bigger in truth, and, uh, by, by… Because by safety nets, by whatever, then, uh, things get, uh, sort of, uh, salvaged lah.

Uh, other, other errors that come in, that are not cognitive, I, I, I classify as… I mean, what else is there? That’s your body loh, your physical error. So you, uh, you really just go and poke the wrong vessel. That’s the error. And then, uh, if you… But those are quite apparent lah.

00:23:52

And then there’s another group called unknown ah. We never found out why there was, there was an error. But actually, if you go and dig, dig, dig, dig, dig, then you found out someone gave the wrong drug or something. Those are unknown because we… Sometimes, root cause analysis found no root. So, yeah, so that kind of things can happen lah.

So, uh, yeah loh. So I, I would think, uh, if you ask me to estimate, I would say 30%. Just that, uh, 5%, which participant 12 quoted, is the bad outcome, then everyone, hor, everyone say, oh, why like that? Why like that? Then that’s 5% loh. And then, oh, why you think like that? You know, we, we could find out.

And most of the cognitive errors do occur, uh, or are discovered, uh, in a junior-senior interaction. That’s my, my, my view from my, my, my sample lah. It’s a junior-senior. That means the junior conduct a case with you, then you, why you think like that? Then that’s how we found out cognitive… If it’s a senior error, it is a bit less likely to be found out, yeah, unless there was a handover process.

SI Okay.

PA10 Or the patient was deteriorated during the particular senior’s shift lah. Yeah.

SI Okay. Thanks. I think that’s really interesting, um, and especially your, your, your comment about the recall, uh, bias for the question. Yeah, so that’s interesting. Um, can I get participant, uh… What’s that? 13 or… Eh. Uh, 11 to, uh, voice your opinion. So like to what extent do cognitive errors play a part?

00:25:38

PA11 I, I, uh, I think when I actually do sit down and, uh, reflect on these cases, they are mostly the severe cases, as what, uh, what… I forgot the previous participant’s, uh…

SI Yeah.

PA11 Yeah, they’re mostly the severe cases. So I, I, I… When I do have, um, do sit down and reflect on these cases, actually there, there is, uh, often a, uh, a cognitive error or component involved lah. Yeah. So I, I think, uh, I, I actually thought it was high. Yeah, high. But, uh, there may be other contributing causes to these cognitive errors lah. As in the root cause may not be this, but rather, um, for example, lack of time, need to move on to other patients, things like that. So there’s only a finite amount of time you can spend per patient, for example. Yeah.

SI So when you say… Sorry, I want to clarify. If you say high…

PA11 High.

SI What is the… What do you mean by high?

PA11 I don’t know. Maybe half.

SI Half. More than half?

PA11 Yeah.

00:26:49

SI Okay. Any, any other comments about this, about this, uh, this question, about the extent of cognitive errors? Okay, or anybody wants to share some, uh, real-life examples? Okay. Uh, if not, I was thinking, actually, when, just now, when participant ten talked about how, uh… You know, he was describing the process of, uh, switching on his computer, and then getting a lot of, uh, other tasks in between. Um, so he was illustrating like multitasking. Do you feel that, uh…

So specifically for specialists or EPs, do you feel that this is more, uh, pertinent or more, uh, of an important factor, uh, or it affects you more than the, the junior doctors? How do you feel? Because I suppose it’s the same environment. Everybody is, uh, multitasking and, uh, time-strapped, and the manpower strain affects everybody. But do you feel that, uh, is there a difference in how this affects the emergency physician in terms of cognitive errors versus, uh, a non-specialist?

PA10 Uh, participant ten. So I just… Just reflecting. I, I will like to… I mean, just comparing myself with when I was a medical officer, I would like to think that I’m wiser, but I’m definitely slower and less adept at multitasking. So I tend to… My plate now is… My, my, my plate now, what you call, the, the, the cognitive plate is smaller. So things drop out from the plate more easily. Yeah, when I was a MO, I could probably… I hold very big, 50 cm diameter plate. Now is 10 cm diameter. Everything drops out, and I forget stuff, and I can’t multitask as well.

SI But can I clarify? Why do you, why do you feel your plate has gotten smaller?

00:29:09

PA10 I think there may be some, uh… What do you call? Cognitive decline and… Yeah. I, I think also because, uh, I, I think it’s because of the nature of the work as well, because if you work as a junior doctor, you are much more task oriented. And then as you are put into more senior, supervisory roles, you become more cognitive oriented.

That means you have to think about how the patient is and what it is a bit more than the medical officer, who sometimes goes into an information collecting role, and then an executive kind of role when asked to carry out tasks lah, uh, yeah, to… And carry out treatment loh, because, uh, when they vet with you, then you, you take over the… You, you, you are not supposed to, but you do take over a lot of the thinking.

So, uh, as time goes on, I think as, uh, as, as you, uh, go more… As you spend more time in that role, then the task orientation part will decline. But I don’t know what the other participants think.

SI Yeah, what do the other participants think?

PA13 I think, as a junior doctor, uh, participant 13, sometimes they cannot, um, they… Either they think their plate is very big and they, and they don’t stop and put and tell people, eh, I cannot… You’re telling me ten things. I cannot do ten things at the same time. They don’t know how to stop people. Or they feel that they cannot do it because they’re junior. They cannot tell the nurse and the, uh, patient to stop disturbing them because they need to concentrate on this one patient and this one task.

00:31:01

Um, and maybe as a senior or somebody, yeah, somebody who is older, who we know our plate cannot be so big, we cannot eat so much, uh, that we need to stop people from filling up that plate, so we deliberately stop people and say, eh, can you just let me log on the computer first? Don’t, don’t ask me whether you want to down-triage or not. I need to log on the computer first, then I can answer your question.

Uh, yeah, maybe it’s our position also. We are more senior. We can push people away or ask them to wait a while, uh, and, yeah, and don’t fill our plate first. Let me concentrate on this task. Or let me send my bloods first before you ask me the next question, because I need to label my, my blood tubes properly, right, before you ask me to vet, uh, the next patient or something like that lah.

Uh, yeah, so… And perhaps we are more aware of whether… From previous experiences, um, that we know that, um, this is a potential problem, that, uh, we possibly would label, maybe, label the, uh, blood tubes, uh, wrongly if we get, uh, too distracted by everything lah. Then we deliberately stop and do that task first loh. Then after that, move on to the next task. Yeah.

PA12 Um, yeah, uh, participant 12. I agree with, um, participant 13. Um, you know, as a senior, we can actually ask them to stop. You know, uh, don’t ask me anything first. And then, you know, I usually, uh, do the things that I need to do before I can actually proceed and, uh, you know, answer their questions.

00:32:39

Um, but if you ask the junior, I’m sure the junior will say, eh, you know, I do a lot of multitasking as well. You know, uh, there’s a lot of things that I have to do. Not only see patients. I have to… Someone comes up to me and ask me, eh, can you print the discharge summary? And then another nurse will come, eh, can you order this, uh, drug? You’ve forgotten to order this drug, you know. So they are also interrupted many times. So they will be also multitasking. So there are… You know, different roles have different kinds of multitasking lah, you know.

So as a senior, you know, like, uh, participant ten said, you know, uh, you know, while you are logging in and down-triaging, you know, at the same time, you know, I have to… Someone comes and ask me about a patient, how to manage a patient. There are a lot of things, uh, that will affect, um, you know, our work. Yeah.

PA11 Yeah. I think that we, both juniors and seniors, are both exposed to, um, similar… These, uh, issues lah, with need for multitasking. Um, every day, you go to, um, the ambulatory area, you see them being loaded with multiple things to do at the same time. So, um, I, I would like to think that, um, the seniors, um, have an additional, um, maturity to, to, um, see through, um, these tasks, um, compared with juniors, who are more vulnerable to, um… Who I, I think are probably more vulnerable to, um, missing out things as the tasks, tasks switch lah. Yeah.

SI Great. Uh, thanks for the sharing. Um, so moving on to the next, uh, question. So for EPs who commit cognitive errors, um, why do you think EPs commit cognitive errors? So this is solely concentrating on if, let’s say, the EP, uh, commits a cognitive error, what do you think are the factors? Like [overtalking].

00:34:45

PA10 Uh, [overtalking].

SI Or errors in decision.

PA10 Participant ten. So, uh, stress. That means stress for time. Um, huge patient load. Uh, one, one particular I want to mention because I think the others may not is subspecialty.

PA11 Knowledge ah, right?

PA10 Yeah, knowledge. So, so if you’re a particular, I’ll just explain, if you’re a particular subspecialty ah, yeah, uh, you, you will consider the diagnosis that your subspecialty, uh, is… How do I say it? That is within your subspecialty ah, and therefore that, that, that cognitive error is very strong. Do I need more concrete example? Okay. Should I… Can say, right? Okay. Safe space, right?

SI Yes.

PA10 [Laughs]. So, uh, for example, a 25-year-old woman comes in. Um, found, found, uh, found at 6 AM. Supposed to wake up, but did not wake up. And then, uh, found in bedroom. Soiled her bed and, uh, with, uh… Can’t move, can’t move her body. And then, uh, pinpoint pupils. And then, uh, the patient is like 25 year old. And then the differential diagnosis is, uh, posterior circulation stroke.

00:36:14

And then the patient is stroke activated, and then, uh, intubated because couldn’t protect her airway and go to ICU lah. Yeah, and then thereafter, the neurologist reviewed the patient and said, oh, this is an organic… Organophosphate poisoning. So yeah, it’s the… Which was the diagnosis. So, so that’s the kind of, um, subspecialty, uh, cognitive error loh that I can think of.

So those, those are… You’re asking for factors, right? So yeah, I think that’s a factor loh, or, or participant 11 said knowledge loh, which I think, uh, it’s not a matter of lacking of knowledge, but just the fact that, uh, I am subspecialising in this one, so I know this one more, and is more familiar, and therefore I will go that way. Yeah.

SI Yep. Uh, anybody else wants to add on? Like let’s say if you have, uh, errors in diagnosis or disposition you discharge, admit, or, or wrong level of care, what are the factors that, uh, lead you to commit those cognitive errors?

PA11 I, I also… I mean, the first thing that came to my mind was time lah. Time pressure. Yeah. Or lack of it lah.

SI Lack of time pressure?

PA11 Lack of time. Sorry. Lack of time. And, uh, sometimes, it’s knowledge also lah. Yeah. No, no, uh, not sometimes. Often enough. Often enough.

SI Participant 12.

00:37:56

PA12 [Laughs]. Okay. Yeah, okay, participant 12. Yeah. Okay. Um, okay, for me, uh, like he said, you know, it’s, it’s time. More like time-sensitive, uh, issues, um, and with… Those with protocols. Okay, for example, stroke. Okay. Right? So when, uh, when we have this, uh, stroke protocol. Okay, a patient comes in. Uh, oh, you do a FAST, but, you know, it’s a plus-minus. You’re not sure.

Um, you know, and then you, okay, because of the time-sensitive pressure, I will just stroke standby, you know. All right. And then, uh, after that, the patient goes for a scan or whatsoever.

Um, okay, I have a typical case lah. For example, um, you know, I have this lady, uh, who was brought in, and she had, um… When I spoke to her, uh, she said, um, you know, she’s just feeling weak. So I tried to get up… Uh, get her to walk, and then, um, she was very unstable. And when I asked her to lift her arm, she had weakness of the right side of the body. Okay.

So, um, I was thinking of stroke and, you know, quickly brought her in, uh, examined her, um, ask her, uh, for the, um, the time of onset, and then, uh, stroke standby. Um, after, um, the whole examination and stuff, you know, when we touch her, she had some pain, but she said that she had, uh, this pain for a long time, but the weakness is new. So, you know, I still had to go through the protocol and call it a stroke, and stroke standby, stroke activate and everything. Okay.

00:39:35

So subsequently, the stroke team came, and then they examine. Okay, this was, um, after office hours. So, um, you know, the registrar, the neuro registrar couldn’t make a call, and he said, okay, you transfer this patient to SGH. And subsequently, he called back to say, eh, you know, it’s more like a pain rather than weakness. But what we saw was weakness, okay, but what he got from the patient was more pain.

So, you know, because of all these time-sensitive protocols that we have, maybe sometimes we are pressured, you know, because, uh, the next day, you know, if you, if you don’t… If you, if you, if you… If there’s a delay in activating all these things, uh, there’s an email from, you know, my, um, counterparts, eh, you know, why was there a delay? You know, yeah. So those kinds of time pressure and, you know, all these protocols, time-sensitive protocols, uh, I think, yeah, will affect.

SI Okay. Yeah. Participant ten, you raised your hand.

PA10 So since participant 12 mentioned her case, uh, it made me recall one other case. But I just want to say, participant 12 was suffering from a fishbowl effect there, where people, uh, standing outside the fishbowl judge your actions retrospectively lah. So yeah, that, that can, uh, affect us cognitively as well, because then you’ll be, uh, I’ll be judged by my actions. I did, I, I did something wrong because someone thought so.

Uh, and, and, uh, looking at her example, I have another example for which I had to… It was a settlement. Okay, it went to money to settle with the patient. Uh, so it was a, essentially, a spontaneous, uh, epidural hematoma that, uh, got thrombolysed ah.

00:41:26

Okay, so the patient had some neck pain, then, uh, also had some, uh, weakness come on, and his onset of symptoms is three hours. This is, this is in the old stroke system, where we had to tele-stroke, I think, yeah. And then, uh, we had a 4.5 hour cut-off window, and the patient came in at three and a half hours. So you had one hour.

And then, uh, the errors just kept piling up because, firstly, we are like, uh, wow, this is very weird for a stroke because there’s only limb weakness, and then he complains of some neck pain. So we’re like, is it something else, like your dissection, etc? So we went through that. We had to stroke activate quickly because there’s only one hour left, or else we will not get thrombolysis. And then, uh, the CT brain was, uh, reported by the registrar as MCA infarct, and then thereafter corrected by the consultant as a normal CT brain.

And, uh, and then, uh, so we, we… When we spoke to the stroke team on the phone, we said, this is a very, um, weird presentation for a stroke because he also has neck pain, and then the pain is like not very, uh… The, the, the weakness, can’t really put a time on it. It’s like, uh, about three and a half hours ago kind of thing.

Then, uh, stroke team, uh, say, okay. Then they look at the NIHSS. It was four. So they offered thrombolysis, and thrombolysis was given lah. Then after that, patient went to the ward.

00:42:55

MRA brain normal. And then they started suspecting that he was doing some psychiatric thing and malingering lah. So the patient AORed and went to NUH and got his surgery done ah, the epidural hematoma and the cervical spine. So… And then thereafter, we settled loh, because mediation and settlement.

So, uh, a lot of time pressure. Um, a lot of, uh, need to respect specialist opinion, because now there are other people, and then you have to figure out… And, and you… If you recall, uh, that time when we had all this, the, the neurologist will blame us if we didn’t stroke standby, and then patient had a bad outcome.

And then the neurologist will offer thrombolysis. And then when… And, and then you are like, okay, the neurologist say thrombolysis, right? They have to give what. And then if, uh, if something bad happens, the neurologist will say, eh, you are the, you are the doctor on the ground. You should… You can still decide. Which is, which is the thing lah. You get all the good, and I get all the bad lah. And that, that is how we felt at that, at that point loh, in, in that time lah.

I don’t know how, how we feel now, but, probably, because now the stroke team interacts with the patient directly, and then there are some, uh, there are some more responsibility… But back then, those were the days where it was like, okay loh, I, I, I, I, I defer to you. I have to defer to you, right? But know when bad things happen is you, you are the primary physician…

00:44:24

So all of this, uh, unclear responsibility will also contribute lah. And, and the fact that this protocolised care happens, because of, uh, time sensitivity, will, will, will just, will just make you make errors loh. You have to throw dice. You’re essentially throwing dice. What, what can you do? Could you have asked for MRI C spine in that one hour? Then, oh, MRI C spine normal, so, uh, okay, we cannot give thrombolysis already. It’s your fault, because you chose MRI C spine. [Laughs].

SI Okay. Um, yeah, so I’m, I’m hearing a lot about, uh, time pressure. I think everybody has mentioned it. That it forces you to commit errors. Do you feel then the reverse is true? If, let’s say, you had a good shift, and your… For some reason, nobody is pressuring you, or you don’t actually have to multitask and you can take your time, do you feel that cognitive errors are less likely or will not occur if you didn’t have time pressure?

PA13 Participant 13. I think to say that it will not happen is stretching it a little bit too much, because, I mean, yeah, like what participant ten said, right, it’s a problem of the brain, right? So [laughs]… Or, or rather our thinking process. Uh, and yeah, so just to take… Count away the time, um, on multitasking, that time pressure, and say that we won’t commit cognitive errors, I, I don’t think is right lah.

Um, yeah, I think one of the thing is also to be aware that there are such cognitive error, like, uh, participant ten said, um, that subspecialty, because of our interest, maybe we are anchoring everything to a diagnosis or to a subspecialty interest diagnosis lah.

00:46:12

That one cannot be discounted by multitasking. You give me one patient. I will still say it’s a stroke for that [laughs] patient. You know what I mean? Uh, even if there are no other patient, that one patient come in, because my subspecialty interest is stroke, I will still say it’s a stroke kind of thing lah. Um, so I guess to be aware that there are cognitive error, and it’s, it’s one step to preventing it lah. Yeah loh. Yeah.

SI Anybody else? If, if you have no time pressure, you can, uh, take, take your time with, with the patients or with the patient care during your shift, do you feel that your level of cognitive errors will decrease?

PA11 I think if you are… If we are talking about extremes, then I think either extreme will be not good lah. Means no time pressure at all or, or too much pressure. Um, yeah, because on, on the other extreme…

SI [Overtalking].

PA11 Sorry?

SI What will happen if you have no time pressure?

PA11 You see, there will be other issues that will come up. Uh, human, natural human behaviour is you go and do other things. Take out your computer to use. Or basically, you, you might, um, just step down a notch, la, and start, uh, you know, your… Start thinking about… Not thinking about this case so much because you are in a more relaxed state. Yeah, so I, I do think that some pressure is, is required to drive performance and improvement. But, um, on the extremes on either side, it will not be good lah. Yeah.

00:47:59

SI Participant 12, anything to add about that? If, if, if the time pressure is, uh, reduced [overtalking].

PA12 I think, uh, it may or may not lah. Okay. Yeah. There’s no clear line to it. Okay. So in that case, uh, that I was talking about, um, if I had the time, uh, you know, I would have actually slowed down and thought of other things, like a spinal issue, you know, maybe exclude all those things, you know, before I come to a, you know, diagnosis. Yeah..

SI Okay. Um, the, the next, uh, question that we wanted to…

CF1 Sorry, June. I just want to ask, uh, something. Hi. Participant ten, thanks for sharing the very, uh… The story, because it really resonated with me also. Um, but in hindsight, would there be anything that… I mean, because it’s something that can happen to anybody, right? So would there be anything that you’d do differently to prevent this error or that you’d advise other people to do in future?

PA10 Uh, thanks for the question. Uh, this case was presented at a department meeting, as part of M&M, and the response was silence. And then the question then… The, the first person who asked the question said, what is there that we can do to prevent this from happening again? Then the response is silence. Okay, that, that is how, how it… How bad it was.

00:49:44

Uh, in retrospect, uh… Yeah, so, uh, much reflection has gone into it. So, uh, in, in retrospect, uh, there were a few key points. Can I don’t stroke activate? Uh, can I don’t stroke standby? Yes or no. I’d say it’s, no, I can’t. Obviously, client has, has, has, has a stroke syndrome, and, uh, yeah, you don’t stroke standby, the patient really has a stroke, then, orh hor, so cannot.

So the next question is, uh, could, could I have… Uh, will I have done the scans, uh, differently? No, I can’t. So, uh, what I’ll have done differently, I, I might have, uh, spoken to the stroke team on the phone, by myself, instead of through my medical officer, and then I, uh, will have, uh, maybe documented very clearly our communication. Yeah. And, and then, uh…

But I think, uh, given the sequence of events, it’s very hard not to offer the patient thrombolysis, because, uh, the, the final diagnosis was found out on day three. So unless I had some way of finding out the, the formal diagnosis, before the decision, then I have a fighting chance. Otherwise, it’s just the cost of doing business, isn’t it? You just have to work on what you have and then, uh, willingly commit the cognitive error [laughs], because, yeah, any way you look at it, it’s a trap ah. There’s no, uh, way out of it, and, and, and you have to do it loh.

Sometimes, that, that does happen, and this is a very good example of, uh… Even with retrospectoscope, can you, uh, really undo everything? Sometimes, the answer might be no. Yeah.

00:51:57

SI Participant ten, thank you for the honest sharing. I think, I think we really appreciate your, your, your reflections, and, yeah, we, I mean, we, we feel that, we feel that that complexity of the case as well with you. Um, then we should just… I mean, naturally, we should… I want to ask about what kind of strategies… Uh, so besides this case, I mean, in, in your, in your daily work, what kind of strategies do you personally use to help prevent cognitive errors? Anybody wants to share what kind of strategies…

PA10 Uh, ten, ten.

SI Yeah.

PA10 Ten. Uh, I will pick the low-hanging fruit. So already mentioned already that, uh, you will, uh… When you, when you realise you’re multitasking, you will put your hand out and say, stop, let me do this first. . Yeah.

SI Okay. Anybody else? I’m sure we [inaudible] our strategies.

PA13 Yeah, participant 13. To be aware lah of that, that, uh, we do have biases. Like an alcoholic who comes in, or a young girl who doesn’t want to wake up, um, that to be aware that, hey, already I’m judging this person for drinking alcohol and getting drunk. Already I’m judging this young lady for not waking up. Maybe, perhaps, there could be something else that I’m missing lah. So I think to stop ourselves and be aware of our own biasness. . And second, and, yeah, and try to minimise that loh.

00:53:46

PA11 I kind of use the same strategy also. Firstly is to recognise the issue that I have. So, um, constant reminders to myself not, not to be, um… Not to, for example, have, uh… Form early, uh, example, early biases when seeing a patient.

SI Is there an example that you wanted to, to tell us? [Overtalking].

PA11 Um, for example, when, um, when I’m on shift, and I was a standby for EMS, somehow, somehow, somehow, um, medical staff around me will, will say always it’s a toxicology case. So, um, I’ll try not to, to narrow down to that diagnosis first and try and keep reminding myself, need to go broad first ah, you know, to prevent that anchoring. Yeah.

SI Great.

PA12 Participant 12. Yeah. Okay. So, um, for example, when I’m in resus, and I’m like overwhelmed, okay, so I, I try to actually, um, get my junior doctors to see the cases, so that I have a bird’s eye view of the cases and, uh, make sure that, you know, um, they do the… You know, give the correct management or treatment for the patient. Okay.

And, um, like when I’m in the ambulatory area, for example, you know, um, the junior doctors are the ones who are, you know coming back to you and telling you about the case, and if I feel that, you know, there is an issue or if I’m not sure, you know, I’ll just go down and see the patient myself, okay, so to try to prevent errors lah. That’s what I do.

00:55:36

SI Thanks for all your strategies. Um, so in your opinion, if, let’s say, uh, in recognising and overcoming cognitive errors, is it an emergency physician’s responsibility? Or do you feel that it’s beyond the emergency physician’s control? So is it… Is the EP responsible for recognising and overcoming cognitive errors? Or you think, in our environment, it’s something that’s beyond control?

PA10 Uh, participant ten. I think, uh, the, the first statement we should, uh, say is, um, we should not aim for 0% cognitive errors because that’s probably not possible, but, uh, what is acceptable risk is something we need to decide. Secondly. Secondly is what we, uh… Can we improve the ways we recognise cognitive errors? Uh, because I think recognition, uh, will tell us how bad it is, how, how, how bad the problem is, and then we can then therefore try to think about whether we can tackle it, and how we tackle it.

And, uh, from the outset, we… If you, if you just say that, aiyah, this is multifactorial, you’re probably right. It’s going to be multifactorial. And, uh, the EP should not… I think the EP should be responsible for cognitive… Uh, for lowering cognitive error rate, but, uh, they should not be solely responsible. It should be, uh… What do you call? Uh, team, so-called team approach, or even a hospital approach, because, uh, the, the, the factors that we already mentioned come from many sources. And, and some are changeable, some are not. For example…

PA12 Participant… Sorry.

00:57:29

PA10 Manpower is changeable, but, but… Manpower is changeable, but, uh, patient coming in with very little history is not. Yeah, so that, that kind of thing.

SI Okay. Yeah, participant 12.

PA12 Okay. Um, it’s, it’s good to have an insight. You know, if you have an insight that you have, you have made an error because of some cognitive issue, um, and, you know, you accept it, it’s fine. Um, but, however, if, um, you know, if someone points out to you, um, we should be able to, uh, take it and not think as a punitive kind of, uh, you know… Yeah.

SI Okay. So do you think the EP is responsible for, uh, recognising and overcoming these cognitive errors?

PA12 I think he’s not the only one who’s responsible. Yeah.

PA13 Participant 13. I’m not sure whether I understand your question. What do you mean by EP is responsible for minimising cognitive error? Um, as in are we saying that it’s the, the hospital should minimise it for us, or what’s the alternative to blame? [Laughs]. Or who else should be responsible?

SI No, as in it might be beyond the, beyond the control of the EP. So like…

PA12 Participant 12. Sorry, can I say something?

00:59:02

SI Yeah.

PA12 Yeah. So if, if it’s like, for example, you know, there’s a bed crunch going on, and it affects the way I practice and all that, so it’s beyond the EP’s control, and then it should be the hospital who should intervene and, you know, help. You know, yeah. So it’s not just the EP’s responsibility here. So it depends lah. Yeah.

PA13 Okay. Okay. Yeah, I get the question. As in is it beyond our control? Or is it something that we can control? Um, I think we can contribute, uh, and try to minimise some cognitive error. But some things, yeah, I agree with participant 12 lah, that it’s beyond our control lah. Yeah. But we can try to mitigate. I think we should try to mitigate. Yeah.

SI If no other comments, we move on. Okay, so, um, there’s, there’s maybe two more questions left. So we are going to, uh, ask, if, let’s say, you have a new specialist, what advice would you give to him or her about cognitive errors, regarding cognitive errors, that they should be aware of as they start their career? What advice for new EP regarding cognitive errors?

PA10 Ten. Cognitive errors are unavoidable. You will make your first one soon, uh, but don’t let that affect you. Recognise them. Accept them. Move ahead..

PA11 I think this topic should be talked about or even taught or explored, discussed during training years, residency years, rather than, uh, as an EP. Um, the EP ideally should, um, have the necessary skill set to, uh, recognise, uh, these errors.

01:01:50

SI And what, what advice would you give to the new, uh, specialist, the new EP?

PA11 Uh, no, no different than ten.

SI Okay.

PA13 I think, in general, I remember one of the profs telling us that when we… When there’s a transition, whether it’s to a senior resident or registrar at our time, and to an AC, um, that’s when most or… That’s when we’re most vulnerable to commit errors lah. Um, and I think to be aware of that, um… It was not specific to cognitive error, but perhaps, yeah, to just tell them that, uh, that’s the vulnerable time, during transitions, uh, when we take on more responsible, and to be aware that there’s such a thing.

Um, yeah, but similar to participant ten, uh, don’t be too discouraged by it. Uh, it doesn’t mean you’re not good enough to be an EP. Um, and, yeah, really, the first time you call MPS will probably be… You’ll probably be distraught, but, but, yes, it will, it will mean you have seen enough patients lah, maybe. Yeah.

SI It’s interesting. I wanted to ask you, uh, why, why do you feel that the, the change in the responsibility, why, why does that make you the most vulnerable at that point?

PA13 I think that’s, that’s what one of the profs told us, when we changed. I think changing… The transition is when we are kind of, um, trying to find our place. Like from being an MO to suddenly being a senior resident or registrar, then, um, it’s…

01:03:39

Our job description changes, right? Our job scope is different. We need to deal with different things. Uh, same as being a registrar, to be an AC. I think there is a change of job scope, and things that we need to do, and things we need to manage. Um, and perhaps it’s that struggle that, that predisposes us or makes us a little bit more vulnerable to committing error lah. Yeah. I, I… Yeah, that’s my guess. [Laughs].

SI Do you feel that way when you were in transition? Or were there any [overtalking] want to share, when, when you were in that transition zone yourself?

PA13 I think in that transition zone myself, I was afraid loh. It’s scary, right? Suddenly, and in our time, probably not as much handholding. Registrar, you’re really the… You can be the most senior on the shift already. There’s no consultant to call sometimes. Um, and, and I think that, perhaps, that fear kept me on my toes to, to say, eh, uh, are you sure you want to do that? Um, yeah, to be extra careful to make sure that I don’t make mistakes lah. Yeah.

SI Anybody else feels the same way, that during the transition period, you are more at risk? So we’re just going to segue into… Segue a little bit into that. Then I’ll get back to participant 12, okay? I think participant 12 wanted to, to, to share. [Overtalking].

01:05:11

PA12 Okay. Yeah. Uh, so, uh, adding to, uh, to the other participants’, uh, you know, thoughts, uh, yeah, as a… You know, not so much from an AC to a C or whatever, but for… When I became a registrar, I had that kind of fear. Okay, so when I was on the ground, for example, you know, there was another registrar, and I had a problem with, uh, disposition, like, you know, which discipline to admit this patient to.

So when I asked the other, uh, senior, uh, you know, and then that person said, eh, uh, you know, you are the registrar now, you should know how to manage. You know, I shouldn’t be teaching you, and then she walked away. So, um, I felt [laughs], you know, pressured, and, you know, I was upset. Yeah. So, you know, we tend to go into a shell and, uh, you know, and, uh, try not to ask for help, okay, when you really need it. You try to handle it yourself. Uh, I think it’s quite stressful. Yeah.

PA10 Uh, participant ten. So my first registrar shift was a night shift, and then, uh, within 35 minutes, I made my first error. After intubating a patient, then I wanted to sedate him. Uh, so he’s like intubated already. Infusion pump running. Then I gave him a nice bolus of 200 milligrams of propofol instead of 20 mg. So yeah, [laughs], yes, it’s a stressful time.

And then your, your, your other senior, your, your other senior who’s a consultant come over, and then he look at the BP and say, why is this patient, uh, DNR? [Laughs]. Why is the BP 24 and you’re not doing anything? [Laughs]. So yeah, it was a time of forced growth, yes.

01:07:11

SI Participant 11, want to share anything about, uh, at that transition point, from… To becoming a specialist? Did you, did you feel that you were more vulnerable to cognitive errors?

PA11 Yeah, I, I just wanted to say I think it’s also very dependent on the, the faculty that… Just recalling what, what happened then lah. It was very dependent on the faculty or the senior con or con that you were on with. Um, some of them, uh, are more approachable, while others are not.

Um, if you meet with someone who has this, you’re a registrar already, how come you don’t know, kind of attitude, you’d be more inclined to hide your mistakes or, or not share them and just, just, just trial and error lah and see if, if things go your way for fear of being judged or being, um, being, uh, scolded lah in that way. Yeah. So that, that is potentially one, one, uh, area that it can go, that can cause the error loh.

SI Okay. Uh, I just wanted to get back to… I think on the chat, participant 13, you mention confidence and overconfidence. Do you mind just letting us know what you mean?

PA13 Um, like they are the registrar already [laughs], so they are kind of given a certificate that you can do anything or everything, if you want. Uh, and perhaps, perhaps, for some lah, that, that overconfidence saying, I pass exam already, I can do anything I want now, uh, yeah, that, that could lead to errors as well lah..

01:08:55

SI Okay. Thanks. Thanks for clarifying. Uh, co-facilitator.

CF2 Hi. This is co-facilitator two. I just want to clarify a little bit, this is really interesting, about this transition timing. Um, I think you all cited some examples about why it would affect, um, at this transition time, like, you know, you pass exam already, or you are a registrar already.

But I’m going to take it deeper by asking, how does it actually affect the way you think? Are you exposed to… Are you forced to think in different way as a registrar compared to MO? Is it because of new, new circumstances or new decisions that you were not used to make? Or is it some other way, you know, that your thinking might’ve been affected? Um, or it’s just because now you’re in a new role, that’s why you’re more vulnerable to take make more, more errors? Yeah, I just want to find out why, why… How does that really affect you committing the error. Yeah.

PA10 Uh, participant ten. So, um, my favourite example is, uh, you’re in a room, the fan is on and pointed at you, then you pull down your shorts, and then the fan blow your butt. Yeah, that’s how it feels. You think the same way, but there’s no longer anyone covering you, you know, like, yeah, and then it’s just that bad feeling that you are, you are out of a shell, you know. Like that’s, that’s how I feel lah. Cold tingle, you know.

CF2 Co-facilitator two here. Sounds like it’s about being more [inaudible] probably the way you now make decisions and [unclear] translated also shows that your thinking is now in a more… Your thinking is being translated in a more tangible and exposed manner. Uh, I get what you mean. Thanks for sharing that [inaudible].

01:10:52

PA12 Participant 12. Yeah. So, um, basically, you know, you are just thrown, um, you know, to, to, you know, either swim or drown lah. Okay. All right. So when you’re in that situation, um, you know, so you, you tend to like, you know, keep to yourself and, you know, try to do things yourself and don’t ask for help, and in, in turn, you can actually make mistakes.

So, um, that’s why, you know, when new, you know, new registrars or ACs or Cs come up, you know, um, my first advice is like, you know, don’t hesitate to ask for help. So it actually changed my mentality on, you know, um, asking for help, you know, yeah, because I couldn’t, you know. So I, I hope that younger EPs will come forward to ask for help, instead of just, uh, you know, uh, keeping to themselves and making mistakes, you know. So that’s always my advice to them. Yeah.

PA13 I guess to add to that, because… Maybe because they are registrar, or they pass exam already [inaudible] sometimes they might think they are not allowed to ask for help, and I guess that’s something that we should tell new ACs lah. We’re always learning, and there’s always things that we don’t know, uh, and it’s okay to ask when you don’t know, uh, rather than assume that I pass exam, I must know everything, kind of thing lah.

SI Okay, uh, I, I just want to cut in ah. Because I think everybody is talking about registrar, right? But, uh, we are talking about, uh, EPs. So like specialist. So at the AC level versus the registrar level, is there a difference, or you think it’s the, it’s the same kind of transition?

01:12:37

PA10 Uh, participant ten. I, I, I note your point, but, um, the reason why we now also move on to talking about registrars is because, um, what we found as registrars has now been pushed back to AC, if you get what I mean. That means, uh, we, we used to feel the transition at registrar level, and now… But because now of, uh… Now we become a bit more, uh, careful in handling registrars. I don’t know about the other hospitals, but, but…

SI Sorry, senior residents, is it?

PA10 Yeah. So, uh, now we, we are… We, we give them more cover. There’s more assessment and all that kind of thing. So it, it seems that the transition has, has slowly moved towards AC lah.

SI Interesting.

PA10 Yeah.

PA12 Yeah, I think it’s because of the residency programme, maybe, you know, because we still consider them as just residents. So it’s only when they become like an AC, that’s when, you know, we feel that there’s a transition now.

SI I see participant 13 is agreeing. Uh, participant 11, any, any thoughts? Do you feel like the resident… The, the… As a senior resident or as an AC, uh, that means the transition to AC or transition to senior resident, is that transition about the same, or it’s a different thing now?

01:14:07

PA11 Um, I, I think it’s… We seem to be talking about handholding and, uh, being able to, um, approach the seniors. Um, I don’t know whether that is the… What, what you were trying to get at. As in how does that make a difference in you, uh, making a cognitive error when analysing a case? Or are we drifting a little bit already?

SI Sorry?

PA11 As in now, we seem to be talking about, um, supervision rather than cognitive errors.

SI Mm.

PA11 Is that where we’re going towards? As in where…

SI [Overtalking]. Uh, so like for…

PA11 Level of supervision.

SI For committing cognitive errors, do you feel that… And, uh, participant 13 was talking about, about the transition zone being a more vulnerable time to commit cognitive errors. Do you feel that the, maybe, the MO to, uh… Or junior resident to senior resident transition was more vulnerable? Or do you feel that the senior resident to AC transition is more vulnerable? Or do you feel that there’s no difference?

01:15:27

PA11 I actually think, uh, okay, as… I think both are vulnerable, because JR to SR, right, um, there’s a big difference in terms of your responsibility towards a case. SR to AC, you are a senior. You are a senior, and then you are still senior. The level of supervision is different. Whereas JR to SR, you are junior. You take bloods. You take history.

SI Yeah.

PA11 JR to SR, the responsibility changes from…

SI Okay.

PA11 I am minion, to I am, uh, overall in charge of the case now. Of course, I know there will be someone supervising me, right, but now I take on new cognitive load already. Uh, I need to critically analyse the case. I need to vet MOs. So, um, I think, uh, they, they are vulnerable lah, JR to SR stage. Yeah.

SI Okay. Uh, okay, let’s get back to just now. I think we were left with participant 12. Is there anything else to add with regards to… So the question was, what advice will you give to new EPs, new specialists regarding…

PA12 Uh, yeah. Okay. So like I said, you know, uh, yeah, first of all, my advice will be, don’t hesitate to ask for help in the initial phase of your transition. And, uh, I tell them, it’s okay to make mistakes, and then learn from your mistakes and move on. That’s my, you know, advice to them always. Yeah.

01:16:52

SI Thank you. Okay, um, I think for the, probably, the last question, I think… And everybody has sort of alluded to it already, especially participant 11. We were talking about, um, yeah, talking about education regarding, uh, cognitive errors. Do you feel that education regarding cognitive errors is necessary as a formal, like, a formal education? And when should it start if, if, let’s say, it’s necessary? And probably the last part. What kind of, what kind of skill set, what kind of education in cognitive errors, uh, would you suggest?

So the… Okay, let’s ask the first part first. I think it’s a, it’s a long question. Do you think education regarding cognitive errors is necessary?

PA12 Participant 12. Yes.

SI Yes. Anybody else?

PA13 13. Yes, and as early as possible. [Laughs].

SI As early as possible. Who else? Uh, 13, uh, 10, and 11. When… Do you think education regarding cognitive errors is necessary?

PA10 Same as 13.

SI Huh? Sorry? Couldn’t hear.

01:18:08

PA11 Yes for 11.

SI Okay. What did ten say?

PA10 Uh, same answer as 13.

SI Okay. And okay, so the second part of the question. When should it start? As early as possible, but, um, what, what is that? Which part of the, the education journey?

PA13 R2.

SI R2. Okay.

PA13 Anyone else want to answer?

PA10 Medical school.

SI Medical school. Which year?

PA10 Uh, year… Clinical years.

SI Clinical years. Okay.

PA11 Can I ask why R2? I’m very curious.

PA13 Because, I don’t know, just I thought just give them one… The first year to, to, to accentuate on their medical knowledge, and perhaps, year two, they can start learning about a little bit more…

01:19:02

But I do see where participant ten is coming from, and maybe, yeah, perhaps, in medical school, they should be made, uh, aware of such a thing as well. Yeah. So maybe medical school, then I change my [laughs] answer. I think it was about EP training, so medical school wasn’t part of the choices.

SI [Overtalking].

PA12 Yeah. For me, it’s, um, maybe early part of residency. And, uh, for the others who have already become EPs, I think it’s good to have a workshop for them as well. I think it will be nice to know more about cognitive bias, and also an introduction to, uh, the medical officers as well lah. I think, um, yeah.

PA11 I think, I think it can be introduced to all levels. Uh, undergrad, postgrad, department level teaching also. Yeah. And, uh, it’s also going to be introduced in the residency teaching. Yeah, so I, I think we kind of recognise its importance, and, uh, it will be, it will be incorporated.

SI Okay. Okay. So, um, the third part of the question, last part of the question. So if… Since everybody agrees that cognitive, uh, error education is necessary, what kind, what kind of, what kind of format, or what kind of skill set do you think this, this education should take? Most cognitive errors is quite a broad, uh, thing. So how, how, how do you think the, the education should be structured? What kind of [overtalking] want to teach?

01:20:49

PA11 Simulation.

SI Simulation.

PA13 I have to agree with participant 11.

PA12 Yeah. [Laughs]. Yeah, uh, participant 12. Yeah, the same. Um, it, it could be two, you know. I mean, um, one is simulation, okay, so that they, uh… You know, we can actually, uh, teach them, you know, uh, what kind of… I mean, cognitive error is such a broad, you know, thing. And, uh, so give them an idea of what it is and, uh, you know, how to overcome, and then maybe some simulation where they can learn the intricacy of, um, you know, all these, uh, cognitive errors lah, which can happen, uh, in our hospital settings, yeah, will be good.good.

SI Okay.

PA10 Uh, 10. So as, uh, the other participant has said already, the… It, uh, it could be a tiered approach, where there’s didactic, and then after that, there’s pattern recognition. Meaning we give them some cases, and then ask them, what… Do you think there’s an error here? What do you think the error is? And then lastly, simulation. You’re put in the, you’re put in the flesh and blood of things, where your amygdala releases stress hormones and all that, hopefully, and then you see how you bear up against that kind of, uh, cognitive, uh, challenges lah..

SI Okay. Okay, um, so that was the last, uh, sort of formal question. But I just want to end off with, uh, any questions from the moderators first. Any… Or the co-facilitators have any comments or any queries.

01:22:28

CF1 I’m just going to ask, uh, something. Um, so I think it was quite, it was quite unanimous that, um, cognitive biases is important, and that it should be included in education, and, um, ideally, as early as possible. But just to play the devil’s advocate. I mean, because right now, we train our medical… We train our residents to give us a very concise history, like, you know, painless jaundice, um, or, uh, hypo, or, or hyponatremia, euvolemic hyponatremia. So we give them very fixed kind of, um, scripts to sell to us.

So if we start teaching them cognitive biases, would that, um, would that result in a lot of, um, inefficiencies? Like they will come to us with a history of abdo pain, but the pain score is, um, 0.5 to 1.0. Um, and, and, um, I know that I have a very long list of differentials. And, um, it’s like our referral letter from the GPs, where, you know, epigastric pain can be triple A, can be AMI, can be a lot of other problems. So how would that translate to efficiency on the ground?

PA13 I think as a junior, even though we do, we do teach them to, uh, come up with a unifying diagnosis, but they have to know why, uh… Which part… Like a chest history, right? This chest pain history is, uh, typical chest pain. Why do I say typical? And which parts of it is atypical? Kind of thing. I think it’s important for them to, to be able to differentiate, even though we want them to be… To come up with a diagnosis or a unifying… Think of a unifying diagnosis.

01:24:03

They have to be aware of which parts of the history doesn’t quite fit in, and which part of it fits in, and kind of debate within themselves lah, perhaps within themselves, uh, as to, is it this diagnosis, or is it not? I think it’s important to have knowledge lah. Yeah.

PA10 Uh, ten. I thought that is what, uh, vetting is about. It’s not just about, mm, mm, yeah, you meet my standard. No, but it’s about, um, you also have to give back and help the person grow, uh, in terms of whether they lack differentials or they need to narrow it down with some important questions. So I, I, I think that, that is where that, that, that education and innovative process, uh, occurs lah.

Like, uh, I give you an example. Sometime, on, on some days, I do… I’m very hard to work with because, um, the MO will come and tell, this, this diagnosis, then I will say, why this one not dissection? Totally throw them off. Why this one, not this one?

Then, um, then, uh, for example, when they are doing a CVC I will walk in and say, eh, you’re in, you’re, you’re in the carotid, yeah, then they freeze. Yeah, I’m, I’m a very tiring person to work with, but, yeah, that’s what I do to help them with cognition loh.

PA13 Yeah, so I don’t agree that teaching them cognitive bias will make them less efficient. Yeah.

CF1 Thank you.

01:25:48

SI Any of the, any of the other co-facilitators want to comment?

PA12 Participant 12. Yeah. So on the ground, I always, uh, encourage my juniors to have a set of differential diagnoses, and, you know, go by exclusion, and ask them you know, why not this? Why not that? You know, before I agree on the diagnosis and management plan, because sometimes like, you know, uh… Most of the time, you don’t see the patient. You just get their history from the junior. So, you know, you want your juniors to be safe, as well as safe yourself. So, you know, yeah, it’s good to educate. Yep.

SI I think, uh, I think if the facilitators have no more questions, then I’m just going to invite any last comments, anything that the… The four participants, anything that you want to add, last minutes?

PA11 Do the participants get a free goodie bag?

SI [Laughs].

CF2 Better. You get a voucher. [Laughs].

SI [Laughs]. Voucher [overtalking].

PA12 But a shopping voucher. Participant 11. [Laughs]. Okay, thanks a lot, you know. Uh, yeah, so I think it was a bit of an eye-opener. I had to do some reading about cognitive bias before I came. [Laughs]. Okay. All right. Yeah. So [overtalking].

PA11 You did homework.

01:27:14

PA12 [Laughs]. I did. So anyway, you know, uh, I get to share some of, uh, you know, my experiences, which was I was holding it for many years. Thank you so much. [Laughs].

PA10 Yeah, thanks, thanks for doing this. I think it is very important. And I, I think both parties benefited, uh, not just your collection of data, but as participant 12 say, we also had some counselling ah. Like we actually relive our experience by recounting and… Uh, perhaps, after holding it for many years, because now we are many years down the road, we have new perspectives, and we will ask some hard questions as well. Yeah, so those are good. Uh, that was a good experience..

SI Thanks. I, I, I think we also learnt a lot from all the, all the focus group participants. Yeah, I think it’s good sharing. Mm, okay, I think we are good. Uh, thanks for your time. Just a little bit overtime. Sorry for the, sorry for the delay. Uh, thank you for your kind sharing. And obviously, we want to reassure you once more that your responses will be strictly confidential. The recording is only used for research and transcription purpose, and it will all be anonymised. Okay? Uh, thanks. Thanks, everyone.

PA12 Thank you. Thanks.

01:28:39
