# Supplementary material for: Dropping the baton: Cognitive biases in emergency physicians
Source: PLoS One. 2025 Jan 2;20(1):e0316361. doi: 10.1371/journal.pone.0316361 (PMC11694980; doi:10.1371/journal.pone.0316361)
Supplement: S3 File — (ZIP) [file pone.0316361.s003.zip › Transcripts/FGD 4.docx]

FGD 4

Speaker Key:

MW Interviewer

CF Co-facilitator

PA Participant/s

00:02:18

MW So as we start our first question, very easy one, uh, can we start off by asking each of you how long you have been an emergency physician? So uh, how long has it been since uh, exiting? Yeah, so uh, just a range lah, like you know, less than five years, five and ten years, more than ten years, et cetera. Thank you. We can start with Participant 14. Or 15?

PA14 Sorry, sorry, 14, is it?

MW Yeah uh, and I’m sorry, uh, try not to use the chat uh, group also because.

PA14 Oh, I must talk, is it? Okay, okay.

MW Yeah, must talk. I’m sorry.

PA14 Sorry, sorry. How many years I’ve been?

00:03:04

MW Uh, years since exiting specialist.

PA14 Oh, okay. I’m Participant 14. I’ve been a specialist since, for five, five years.

MW Five years, thank you.

PA14 Thank you.

PA15 Uh, I’m 15. Then I’m four years. [Laughs].

PA16 I’m Participant 16, one year.

MW Okay. Um, and uh, so try to just think back on some of the errors, medical errors that you committed, either you yourself committed or other EPs around you committed. And only in the period after exiting as a specialist so those errors that you committed before exiting um, don’t count lah, all right? So what are some of the common factors or circumstances that lead to medical errors among um, specialists? Um.

PA14 Uh, I’m Participant 14, I think it, it, we tend to make errors when it’s busy and then there’s constant interruptions.. interruptions… interruption. Yup. So sometimes uh, um, like when you know, uh, we get ECGs thrown in our face, I’m sorry. We get ECGs uh, given to us in, in the middle of uh, probably discussing a case with a medical officer or reviewing a patient. Then I think sometimes uh, uh, maybe the ECG doesn’t get uh, enough thought or, or we are looking at it halfway then we get asked a question or a patient turns unwell. Then, then subsequently I think those are the situations. That’s one situation in which we tend to make errors.

00:04:47

PA15 Uh, okay, I’m 15. I’m trying to remember the uh, any of them but I, I, I must be forgetting some. I think probably the most common, I can’t really remember. But I, I, I think, I think I might have mislabelled some things at some point [laughs]. Some blood tube specimen and again I think a reason for that is probably the same thing, when you get busy then you don’t check. Um, so it’s either busy, uh, getting very busy and uh, too many interruptions.

MW Mm. Participant 16?

PA16 I’m not sure if you count uh, near-miss a medical error. I think um, patient factors uh, especially patients who are deemed to be um, you know, altered mental state. So uh, for example, I think recently we had a case of uh, a guy who had a history of epilepsy but very long has no breakthrough seizure. He was brought in by SCDF uh, who I think brought in by SCDF to uh, say query uh, post seizure. Eh, was it post-seizure or? It wasn’t a stand-by case. It was brought into critical care area.

And that guy actually was uh, was a bit altered and we scanned the brain then after that, he’s okay. Then after uh, that the MO see first lah. Then after that we went to check the guy, he started shouting for quite a while in the CC and, because it was busy and everybody ignored him. There were three seniors mah. Three CC seniors, everybody ignored him.

00:06:39

And then finally we cannot take the shouting and MO went to check on him then he say [laughs], I think you know the case also. Then he say oh, he has a, uh, don’t touch me, I have pain. Uh no, he was shouting, then after that say where is your pain? Then he said “I don’t have, I don’t know, I just need to shout”. It’s very, it’s a very, very weird case um, and we couldn’t really uh, identify what was his premorbid lah and all that because couldn’t find the family members.

At the end of it all, it appeared that um, uh, he had a breakthrough seizure for uh, unknown reason. And that he has a seizure that caused a, he has OPLL as well, cervical spon uh, OPLL of his neck. I think there’s some uh, after the seizure he had a, he had a, he had a, uh, was it a cervical fracture or something? A fragment got compressed into the spinal cord or something like that. I can’t really, I didn’t really follow though the case lah.

We thought it was central cord uh, after we uh, after, after it all lah but it wasn’t. It was some um, uh, it was some like, some of the fragments compressing on the cervical cord. But his shouting was actually a urinary retention. So we actually took a step-wise approach to uh, insert an IDC and then put on the c-collar and then went for the cervical uh, CT scan.

There was a delay for about two hours uh, during that period because it was brought in as a non-stand-by case. Uh, it was triaged and then the work up was like a postictal drowsiness so you thought it could be a Todd’s paralysis. Then after that patient started shouting and then it was really ignored for like [laughs], three seniors ignored him for about half hour to one hour in the CC. Then after we went to talk to the patient, and he couldn’t express where his pain was.

00:08:33

Then finally we tried to examine him then we realised that uh, actually he has a ARU. He couldn’t even say he has ARU. And to be fair, he was actually an IT specialist uh, engineer and all that. Um, uh, he has some brain pathology which caused a lot of calcification in his brain as well but he’s actually a normal guy. So we tend to uh, write off patients quite early for those who have uh, some neurological history. When they started shouting, we just ignore them. Yeah so I think that’s one of it as well.

Uh, the other incident I have was with a medical officer but actually not um, uh, myself. Uh, where there was this very unkempt guy who uh, who always drinks alcohol and then um, always come in uh, for various reasons and all that. [Clears throat]. And the medical officer was like uh, we can actually discharge this patient, I think he’s a regular comer.

But actually, he has a lot of [laughs], he has a lot of um, medical issue. He, he, because he was so unkempt and he’s not uh, able to take care of himself, he actually came in with severe dehydration. His normal blood pressure was like 160 but he came in with a blood pressure of about 100 to 110 but it looks normal to us. Like normal, pseudo normal.

00:09:49

And then uh, he say he has weakness and all that but actually he has uh, a severe, he has AOCKD, he has metabolic, he has uh, I think hyperglycaemia not in crisis. And then he has severe postural drop as well. But the medical officer wanted to write it off straightaway lah because this guy regular attender and all that.

So I think one of the uh, factors is actually from the patient himself. Patients who are unable to express themselves well, uh, we tend to write them off quite early. Yeah, that’s uh, the two recent um, memory I have lah. Because you limit to one year, right? So I [laughs] [unclear] I really think about uh, residency years I have a lot. Yeah.

MW Mm. Um, participant um, uh, so, so why, why would doctors want to ignore such patients then? As in um, because normally we don’t ignore patients, right? So then the patient is screaming away um, what, what would make you write them off as being um, uh, psychiatric or you know? What, what would make a difference in this case?

PA16 I think if you are less busy, uh, you might pay more attention to such patient. I mean we also had a recent, another case that escalate suddenly remembered is the, is the guy who uh, alcohol intoxicated, fell, and lacerated his lip. Again, he was shouting at us, saying “what, what emergency is this, half an hour here, nobody see me, I’m bleeding to death”. And then there were three seniors again and three seniors were busy with like sicker patients who have diverticular bleed, hypotension and all that sort of stuff. And he was shouting and then fighting with nurses and shout and shout. And it was chaotic lah. Even with three seniors on the ground, we were trying to manage as well.

00:11:41

I think in the end when I reviewed the patient, he actually has a superficial lingual artery laceration. We needed to temporise with a suture and call OMS in. Um, again, it’s really the supply over demand thing. Like there are more patients than we can cope and relative sickness wise, there are patients who are more ill than others. Everybody is a uh, is uh, urgent case or emergent case. And you need to prioritise lah. And people, and patients who irritate you makes you even more likely to ignore them. [Laughs].

CF Oh, okay, thanks uh, Participant uh, 16. Uh, we’d like to hear from the others also. What do you think of what Participant 16 has said? Uh, you know, do you agree or, or um you know, um, about what, what she has described there? Maybe Participant 15?

PA15 I know everybody here so it’s very funny too. [Laughs].

PA16 [Laughs].

CF It’s okay, yeah.

PA15 I can recognise.

CF But please uh, maintain your [overtalking] confidentiality and others as well, thanks.

00:12:56

PA15 Uh so, what was the question again?

MW Um, what do you think of what uh, Participant 16 said.

PA15 Uh, yeah uh, about, about why we ignore patients, is it?

MW Mm.

CF Yes, uh, ignore certain patients.

PA15 Yeah, I agree, if the patient is being uh, quite uh, uh, being annoying or frustrating sometimes uh, that makes it easier to try and block off that patient and try uh, you know, ignore them. And sometimes you, if you are able to, to uh, go through a mental checklist still and make sure that he patient although annoying is still uh, is still okay or still safe, like I mean you have a, you are able to safety ignore the patient [laughs].

But um, I think again it’s the same issue with if the um, if it’s very busy and you have a lot of interruptions going on, then you are not able to reach your, this mental checklist or is the patient safe to ignore [laughs] kind of thing. And then um, you again, you end up ignoring the patient and missing something lah. Yeah.

I, I think um, if even if the, if the shift, even if the patient is extremely annoying but the shift is not that busy and you are not faced with interruptions, you will still be able to assess the patient um, because I think it’s still, you are still able to, to kind of like you know, identify that yes I am, I am frustrated with the patient because the patient is shouting. Um, but it is the rest of the, the stuff that causes um, that makes it difficult to um, give your full attention to that patient lah. I mean you don’t have that much bandwidth to deal with this irritant anymore if everything is very busy and you’ve got a lot of interruptions.

00:14:35

CF Okay, thank you. So if you have no interruptions at all, then would all these patients who are supposedly irritating or difficult to handle, um you know, um, would they be ignored? Would they or, otherwise?

PA15 I think that, I think that if you don’t, if you have enough bandwidth, you don’t have that kind of, you don’t have so many interruptions and you don’t have, and it’s not that busy, and you don’t have a lot of other things on your mind, I think you have more bandwidth to be able to at least um, you know, look at the patient lah.

CF Okay, thank you. Uh, would Participant 14 like to say anything or add to this?

PA14 Yeah, I agree. I think that uh, the part of, the part about being too stretched is, adds a really big component to um, whether you know, whether safely ignoring patients or whether it’s uh. The other thing is also sometimes it’s the information presented to us. Sometimes we are walking around the you know, the, the ED, the department and we may be attending to a patient, taking bloods, et cetera. And then the nurse you know, or somebody may come up to you and present to you a case and ask you a question.

00:15:45

But uh, and it seems like very complete set of uh, information that they’ve given you and think you can make the decision, downtriage, uptriage, or admit, you know? Uh, but actually if later on then you find out that there was like some very crucial information that was documented or on the system that it was not highlighted verbally to you and then it sounds, it makes you feel very uh, betrayed [laughs].

But then it, it, you know, it can be potentially unsafe also. So I think if we’re not like um, being distracted or we are actually we can slowly make, take our time, review the patient, review all the necessary information, we’re not interrupted, we do things one, one step at a time, then I think most of us will reach a more you know, conclusions that are safer lah.

But sometimes in the, in the haze and, and when you are trying to multitask, not presented with all the information and you feel that, or it sounds as if you don’t have to, there’s not more information that you require, then I think sometimes yeah, that leads to decisions that we would not otherwise have made. Yup.

MW Participant 14, in that case, can I ask you, do you think that um, this is the responsibility of the EP because the specialist is the one who oversees? And end of the day the EP, the specialist is the one who makes decision that this information is enough? Or is it the responsibility of the medical officer presenting to, to present properly because, because end of the day, the medical officer may not recognise that this is a crucial piece of information in that sense.

00:17:27

PA14 Yeah, correct. So I, I think, I’m not sure but I think in the eyes of the law it probably is the specialist uh, up to the specialist to decide. and that specialist holds the final uh, responsibility lah. But I feel that actually it should be, it’s a system thing. You can’t say that the specialist is, well I feel, that we don’t intend to be negligent. It’s just we’re trying to keep other patients safe as well, right?

When it’s like CC is like blocked up and choked up and there are patients screaming everywhere. We are also trying to make sure that other patients are safe, and we have limited attention and time. Um, you know, fully going through case by case would be also, and taking like one hour for each patient would also be dangerous for other patients. So it’s a balance I feel.

Um, of course you can, you know, if we say it’s the onus is on the medical officer, then it’s a bit unfair also. It may also be unfair uh, you know, unless you are intentionally trying to withhold information which I don’t think most doctors would do, or most healthcare workers would do lah.

But whether it’s just a recognition or knowledge problem, then it’s hard to sort of put the blame on them as well. So I, I don’t know [laughs]. I don’t have a straightforward answer to it. I but I unfortunately I think legally as far as I understand it, it’s the responsibility of the specialist which makes it all the more scary.

00:18:58

MW Um, what do Participants 15 and 16 think?

PA15 Um, I also agree that uh, I mean it’s the same thing essentially uh, when you’re very stretched out and then uh, you have to, it’s the management of resources and management of your mental resources as well. So if you put all your mental resources in one patient, you may be, you know, it may be missing in some patient elsewhere as well.

So um, it’s important to kind of you know, well I don’t think it’s a problem that can be solved based, just based on the fact that somebody is screaming in the corner and you, you’re trying to solve that screaming patient lah. But um, uh, I think probably the, must look at the system, see whether there’s any other systems in place in terms of whether or not the team can also help uh, to assess patients who are you know, who are a bit more annoying [laughs].

MW Mm, okay. Uh, what about um, Participant 16?

PA16 So I think when we are busy and overwhelmed, we have mental heuristics that we use. I call it like mental shortcuts lah.

MW Mm.

PA16 That we tend to um, use to kind of manage say manage a P3 queue or manage a CC queue. Um, sometimes these mental shortcuts would um, lead to certain uh, will save majority of cases, then minority of cases you might miss. For example, you say uh, 30-year-old chest pain is a nonsense case for example. Unlikely to be a, a, heart attack in this type of case. And aiya, just do a troponin and ECG and get rid of the fellow.

00:20:56

Then some, turn out to be some dissection case then you won’t know. But they are the rare ones. So you, like what um, Participant 14 say, you can’t spend all your resources on that one, trying to catch that uh, rare case.

And, and sometimes the mental heuristic will fail you and that’s why it’s a team uh, teamwork lah. It’s not just the doctors, you have nurses as well as junior doctors as well um, to, to constantly help each other.

Or uh, we have, we make notes. Let’s say we right-site a patient out of critical care area, we do uh, notes to say why we do that and this was the brief history given by us by SCDF. So the message doesn’t get lost in trans, um, translation. I guess there are things that we can help us to catch the, the, the minority odd cases and, and while still manage the majority uh, sick cases lah. Yeah.

MW But do you think that um, when that happens um, do you think that there is warning? Like do you, when you make a decision that is wrong, because people say that when you make a decision that is wrong, it feels that, that decision is right also. Well, what we call blank spot bias when, when you don’t know that you are wrong because people would not know, not purposely commit an error in the sense, right?

00:22:12

So in that case, how do you identify that this patient is a rare case? And, and since like you said, we cannot throw all the resources into managing um, uh, a relatively well 30-year-old with chest pain?

PA16 What’s your question again?

MW Sorry, very long question.

PA16 How you’ll not miss it ah?

MW Uh, as in since we don’t know that when you are the one making the decision right, when you are the one making, even if uh, even if it turns out to the be a wrong decision, at that point when you make the decision, it feels like it’s the correct decision, right?

So how then would you identify that this person is you know, not so straightforward? I need to take a step back; I need to think slowly. Or, or when will you say go ahead, this patient is a straightforward case that um, you know, it’s nonsense case, I just um, do a one troponine and discharge in that sense?

PA16 I guess it comes with uh, clinical experience plus attend more M&M with the M&M committee. So [laughs] then you adjust your heuristic more. Uh, I think usually it’s first by presenting complaint then the past medical history, and then the vital signs. These three are the things that we have at the start when we’re trying to right side a patient here and there.

00:23:33

And then when case is discussed with you, you get the detailed history and then you get the labs and investigation and you try to uh, see if anything doesn’t match lah. I mean you don’t know what you don’t know um, uh, unconscious incompetence can become conscious incompetence as the end of a lousy case lah.

So [laughs], it’s a cons, I mean I can’t, I don’t think I have a solution for that because you really don’t know what you don’t know until a more senior person highlight it to you. Or um, or uh, someone uh, so-called uh, catch it lah before you miss it lah. Yeah, that’s my point of view, I’m not sure what the other two participants think.

PA14 I, I think the mental heuristic part uh, I mean just from some cases that I’ve gone through, I feel that uh, I agree with oh no, I lost track of which participant it is, the previous participant, uh, what she said about the, what she said about the, the part where um, when it doesn’t match what you think it’s supposed. When, when new information comes in and it doesn’t match what you think it should be or it doesn’t fit then uh, you should change the, you know, change as in it should alert you. So I find that that is very true.

And if uh, we, we always do investigations and we give treatment and we usually have some sort of uh, provisional diagnosis so we expect certain investigations to come back in a certain way or we expect the patients to respond to treatment in a certain way. And when it doesn’t fit, then I think um, it’s time to relook at the whole case.

00:25:09

So two things, I think one is that um, if yeah, so it’s just to relook at everything from the start and to slow down. Because as we were saying, we take a lot of sort of mental shortcuts uh, when it comes to the run-of-the-mill patients we pattern recognise quite a lot. But one, when things don’t fit then I think it’s important to, or I, I try to tell myself that it’s time to slow down and uh, relook at everything as a whole from the start.

The other thing I find useful is to get a second pair of eyes also. So I get a colleague or phone for help uh, actually. And then uh, sort of run through the case with somebody who um, maybe my peer or maybe my senior, even more senior, you know? And I think that sometimes just the objectivity and the fact that they haven’t uh, gone down sort of a mental rabbit hole makes it easier for them to consider other things that you know, maybe, we may be blinded to as the primary provider. .

And then of course the unconscious incompetence uh, is true also. I mean we can’t know everything I feel uh, no matter how much we know. But I find that sometimes um, I still think that it’s always okay to ask for help when. So certain things may not fit and uh, you may not know why. And uh, maybe like a really rare condition that we don’t know about and, but I think we know enough to try to stabilise the patient according to first principles and then try to at least decide who the relevant person to call is. .

00:26:49

And uh, then I think you know, the inpatient specialist have their role also lah because they know all these like you know, rare stuff and you know, a little bit more of the stuff that we don’t commonly see. And they manage you know, certain conditions that are recalcitrant to common treatment. So they may come down with like, they may come down with um, alternative treatment modalities that may be useful that we may not have hard about but then subsequent we learn. Yup.

MW Participant 14, can I uh, trouble you? Could you give me example of like, like what you mentioned earlier? Um, you know, a case where a second pair of eyes changed things or when things didn’t make sense and you know, you, you, you um, took a step back and, and that’s solved or, or allowed you to identify what was the real problem?

PA14 Yeah, I, it’s, it was in resus. I can’t remember the case exactly but it was a very, very busy shift and all the patients were very complicated. And then it was a stand-by for, I think it was a standby for stroke. Um, so the patient came in uh, obviously not a very good historian. Uh, complained of one-sided numbness and weakness, complained of some headache. And then just as, um, the BP was very high and just as we were about to send for uh, the CT scan prior to neural review, the patient then complained of a chest pain and abdominal pain again.

00:28:21

And then uh, you know, I, I was, I had already ordered. There was a similar case prior that and I had already ordered a CT, aortogram because I was concerned about the neurological deficits and the chest pain. And the abdominal pain and the headache and the neck pain. [Laughs]. And I was just you know, wondering to myself how, how, how? Am I missing something, or do I really need to order an aortogram? Is it, am I being too, is it, am I just being influenced by the previous case? Am I being too careful? I can’t you know, or should I really just order the aortogram?

I was like very; it just threw me into a loop. And um, and then I, I sort of sort of uh, sort of just asked lah another senior who was on shift. Uh, just to, I was very honest and I said I, I just need a fresh like you know, I just need to pick, pick your brains um, and what would you do because I have many patients who were very complex and I think my mental, my cognitive load was really there lah. I couldn’t probably I couldn’t take very much more.

Yup, so I sort of just wanted to make sure that I was okay, I wasn’t missing anything, being too careful, or being overly zealous in my investigations, yup. But that was fine. And then um, you know, he, he then said yeah, I think you know, the cases are indeed quite complex, and you can’t tell. And given the severity of the chest pain and all that, yeah, he agreed with my plan lah. But I, I felt like I needed somebody to sort of agree with me. Yeah.

00:29:59

But I mean I don’t do this with every patient but once when, when I think once we’ve reached our mental sort of cognitive load, the threshold, the limit of it, then I think that’s when uh, I would, that’s something that I found to be helpful.

MW Mm, I’ll move on to the next question. So to what extent do you all think that cognitive errors actually play a part in um, specialists, EPs committing medical errors? Um, maybe Participant 15?

PA15 Okay, it’s still the same question as just now, is it? What are the factors that lead to medical errors?

MW Um, but not so much what you think are the factors but do you think it’s the only thing? I mean or the, the biggest part of it? Or do you think um, because you all mentioned various factors right? Like um, uh, busy shift, disturbances, knowledge deficits, cogni, uh, cognitive errors. So is cognitive error the biggest thing, or is it a small thing, or is it part of it only? Like just a, you know, one of many other factors?

PA15 Um, I think they all combine to each other actually I don’t think there’s one factor that is greater than the other. It’s a dynamic thin, right? Because let’s say, let’s say I have cognitive error. I’m aware of my cognitive error. But that awareness or I’m mindful of the cognitive error let’s say, or I’m mindful of the fact that I’m tired or I’m, I’m burnt out or something on shift.

But that awareness is going to drop when your bandwidth drops. The more bandwidth you have, the more uh, you are going to be able to be aware of your shortcomings. But if you, if you don’t have that bandwidth, you won’t be able to be aware of it.

00:31:51

But if you put it the other way around, let’s say you have a lot of bandwidth but you’re not aware of this stuff, then also no difference. It’ll be the same thing. So I don’t think it’s like uh, one is bigger than the other or one weighs more heavily than the other.

You kind of need both the awareness of um, your own limitations as well as the cognitive errors that you are prone to, as well as the uh, the bandwidth available lah. You need all of them for you to have a favourable outcome. You lose any one of them, your outcome is probably going to be less favourable than you have wanted it to be.

MW Mm. Uh, what about participant 16? Will you agree with what uh, Participant 14 said?

PA16 I think uh, I would say it’s like as a Swiss cheese model where everything aligns. So like patient factor, environment factor, system factor, the physician factor. When, when an error happens, there’s always a contributing factor. Like resus was too busy. Uh, the, the, it’s just, it’s just everything gets joined together then the error happens. So uh, it’s not just one like I mean cognitive might be the med, the bulk contributing cause um, but there are other factors that plays a part as well. Yeah.

00:33:05

Like how system error for, system factor for example. ECG always being signed by CC senior from a, and, and the, when the CC senior ask for a repeat or someone else asks for repeat, the person who asks for repeat doesn’t see the repeat ECG. It’s someone else being asked to sign, for example. And most of the time we do that.

And it’s a system thing like oh, CC senior the, the, the M2, the E2, or C2 is the biggest and therefore have to sign. But shouldn’t be, shouldn’t it be the physician who is managing the patient ask for the repeat ECG be the one who sign? Or the patient who is being seen in the consult area or what being the one who sign? But it’s a system-based thing lah I guess.

So um, you being interrupted to sign an ECG while you’re clearing cases uh, with a junior and trying to think about a, a patient in more depth, then you commit an error for that patient because of some unrelated systemic uh, factor you cannot control.

Uh, for example, or uh, the way our P2 and P3 queues are being collapsed in the ambulatory area. After triage, you got to re-triage yourself again um, to make sure that the sicker patients are being seen first so that they won’t be a bomb in the five-hour waiting queue.

So then after that when we are trying to do this, someone clears a case with you. Again, then you, you miss out or this just boom and then you forgot where you last went off or. So it’s a system thing that affects your decision like uh, trying to when you try and watch a case or something like clear a case, think about a case and things like that.

00:34:36

So it might related, unrelated to the physician um, certain things like interruptions are system factors mah. Uh, physician cognition, uh, physician knowledge gap or whatever is a physician factor. Then patient itself making noise, the repeat attenders, the high-frequency flyer that AIs, that tends to be missed as well. Yeah.

So I think it’s a Swiss cheese that combines all together when something happens. Uh, it’s not just one individual. I can’t say whether all will happen at the same time or uh, so certainly there will be some factor which is the major contributing one. Yeah.

MW Mm, how about, what about um, uh, Participant 15?

PA14 I think it’s me, 14. I think.

MW 14, sorry.

PA14 Yeah, yeah, so um, I agree of course that there are multiple factors uh that come into play uh, that contributes to an error. And ob, obviously I think depending on the individual error then the contrib, the amount uh, to which each factor contributes to the final error will vary. I think it feels of course sometimes it feels like cognitive errors are the bulk of it because I think on a particular shift, any particular one shift uh, it’s probably the factor that we can control the most in a way. We can’t really change system factors very, in a very quick way. Uh, patient factors, hard to control. Environmental factors again takes time to sort of uh, modify the environment, modify the system. .

00:36:08

So I think it’s the only thing that we can sort of change and um, lessen. That’s within our power to lessen while we’re actually on shift lah. So I think it feels that way but it might not actually be lah.

MW Let’s say you have more time right, then would you, would you still commit um, cognitive error? Um, given that you have more time since I mean like what you said in your earlier case that um, because uh, you know, you already knew that this was um, you, you had ruled out a dissection. But because um, uh, you, you had a previous case like that so you’re worried whether um, you know, um, your, your mind of thought was framed by the previous case. And therefore, you felt less uh, reassured and you wanted to get clearance. Or you wanted to check with, check in with another senior first.

So what I mean is that um, if you have more time, do you think that there’s a chance that you would overthink things and then commit an error as well? Or because if you have more time then it’ll be less likely to commit an error?

00:37:11

PA14 That’s an interesting uh, that’s an interesting thought. Uh, if don’t know because I don’t feel like I always, I don’t, I don’t really feel I have a lot of time mostly [laughs]. But that’s interesting thought. I’m not too sure. Um, I think um, there may be an, there may be an instance where we over investigate, and we sort of confuse ourselves in away.

For example, like ordering tests. Maybe ordering a troponin when actually it didn’t need to be done and then you’re stuck with troponinitis, semi-half-baked raised troponin that we don’t know what to do with. So I think, I think having too much information may potentially uh, confuse things as well. .

But I think all in all, um, especially if the patient could be admitted, especially if the patient is particularly ill, I don’t think that that’s a big problem actually. The over-investigation. I feel like it’s not so much of a problem. It’s probably the patients who are really well whom have all this incidental findings that, that will confuse the picture and lead to sort of cognitive errors in a way. .

Um, I think uh, of course I think it’s just a propensity thing so if, if we’re busy, then the propensity to commit cognitive errors are higher. But of course, it won’t, it doesn’t mean that having all the time in the world will be perfect lah. It also depends on what type of cognitive errors you’re talking about. For example, anchoring biases and all that, it may be just that you know, no matter how free you are, you have anchored on a diagnosis and uh, and then you just commit that error.

00:38:51

I don’t think there are certain errors that are not uh, so dependent on sort of attention or having enough time. But I guess as a whole, then yes, having more time would, there would be a lower propensity I think to uh, commit cognitive errors. Yup.

MW Mm, Participant 15 um, what do you think. Or um, do you have any examples or things like that?

PA15 Examples of?

MW Um, cases where um, you felt that you know, with more time then um, um, probably you, you may not have made that, uh, cognitive error or.

PA15 Uh, give me some time to think about it. I don’t have any off the top of my head.

CF No, we’re wondering whether there are other examples of you know, if you so much, if you have enough time to think about the situation, uh, yet you know, cognitive errors can occur. Um, yeah.

PA15 Like a personal example? Personal example I need to think about it.

CF Either personal or other examples that you have encountered with other colleagues lah. Uh, as Participant 14 has mentioned.

PA14 Yeah I mean you know patient improving, pain going away is sort of uh, bit of a cognitive error that we all, actually we rely on it in a way but then also at the same time it may contribute to cognitive error.

00:40:25

For example, like a headache that you know, resolves with analgesia but actually they’re red flags, right? Then sometimes uh, I think if we don’t yeah, it might not be so dependent on uh, being busy. Uh, we sort of observe and then if patient feels well, feels comfortable, they look well, look comfortable, then we just discharge them. But I think that, that um, is something, is sort of a mental thing that we do. But it may not be that the patient is uh, really well. Yeah. And I think that, that doesn’t really depend on the time because we observe them for a really long time. And then when they improve, we just discharge them.

PA15 Yeah, I also agree with, with that um, example as well. Um I think, I think the main thing is really just how aware, self aware the individual is with regards to when you’re not on shift, you should be aware of your limitations in that sense. As in what are the areas in which, what are your blind spots and what the areas in which you are not confident in.

Uh, what are the areas where you are more likely to cause problems? What are the situations in which you are more likely to you know, make an error lah? So that when you are on shift, you can be, you can recognise those things. Because the issue is the recognition of whether or not you have uh, recognition of um, the situation and recognition of any errors, right? If you’re not aware of any errors, you’re going to maybe commit them most likely.

So I think you have to, there are two factors involved if you’re not talk about time uh, how much time you have on shift, how busy it is on shift, then you need to first of all be aware um, before you start shift. Or rather in your, before you are in the ED about what are the errors you tend to make. And then when you are on shift, you need to actively work to avoid those errors uh, and actively work to, to um, uh, pay attention to what exactly you are doing in that moment lah.

00:42:39

So when you see an individual, see a particular patient, you every time you see a patient and you come up with a diagnosis, and come with a management plan then you need to, you need to be able to relook at your diagnosis, not just when your management is not going the way you want it. Because like Participant 14 was saying, if you rely on your management plan to go the way you want it to go um, as a way to substantiate your diagnosis, then you can make an error in that way lah if it just so happens that your, your answer is correct, but the working is wrong.

But um, uh, if you, if you have a way to kind of substantiate your diagnosis, then that may reduce that chance of having that error lah. So whether it is, involves just forcing yourself to come up with other differentials or rather I think it’s more important to force yourself to disprove other differentials or prove your, your current diagnosis is the top differential as opposed to just coming up with differential, uh differentials for that individual patient. .

And so every time you see, when you’re looking at your management plan and you’re looking at the answer to as in how your management plan is turning up, you can at least compare back to the different differentials that you have and see whether or not you might be missing something in that case lah. Does that make sense?

00:44:00

MW Yup.

PA15 Okay.

MW Thanks uh, thanks participant 15. Uh, I think you brought up a few points also about um, ruling out other differentials um, in addition to. So like whether what, what I think some people call it forward and backward thinking. So forward thinking will be this guy has a fever therefore he has UTI. Whereas the other one is I need, before I say UTI, I need to run ammonia, diarrhoea, da, da, da, da, da. All right but um, I just um, sorry um, co-facilitator, you have any questions or anything before I go on?

CF Yeah, yeah, can go on.

MW Okay, okay. Um, so um, just uh, coming to the next part, so um, what advice would you all give to new specialists given that you all have quite a bit of experience now. Um, what advice would you all give to new specialists about um, cognitive errors that they should be aware of? Maybe participant 16?

PA16 I’m a new specialist ah? So I think.

MW No, a newer specialist.

00:45:11

PA16 15 and 14 should answer this question. [Laughs].

MW Okay, okay, maybe 15 or 16 first then. Or 15 or 14 first then.

PA15 I mean, I mean I think it’s the same in the sense that uh, it is you, you need to know yourself lah. You need to know what are the areas in which you, you have to make. If for example, if your issue is premature closure, I think you need to be hyper aware that every single case that you clear or every single case that you see, that you might be making premature closure, this error and you should pay attention to it.

And every time like what we were talking about earlier on about anchoring biases and stuff like that, every time you see a patient, you should question yourself like what uh, Participant 14 was doing lah. Is it really this? Is it? Is this really the case or am I just overthinking things? And, and if you’re not sure, you get a different, you get another set of eyes on it.

Um, and also be aware of the issues uh, what are your personal limitations. So for example if you know that you can only handle three things at once, like the maximum number of interruptions you can handle is three interruptions. Okay, that’s it. So the moment three interruptions come, the next one, next one, you have to say, you have to say no. . No matter what uh, what the interruption is, you need to say no because there’s always going to be another senior on shift because you work in a team.

00:46:24

So you need to direct them to someone else at that moment unless they’re, they’re shouting at you and saying ah, the patient has collapsed, dying or whatever. Then, and you’re the only senior there but barring that, you need to say, learn how to say no to interruptions.

And recognise when is your limit. Some people the limit is five, some people it’s four, some people is one, you know? So interesting to recognise what is your limit. And when you reach that limit, you need to say no until you’ve cleared your current task list or else you’re going to miss things out.

And then the other thing would be what are the situations in which you tend to make errors. So the usual situations like what we were saying if it’s an annoying patient or we are personally very frustrated or we come on shift and it’s not a good day. So you need to recognise that you are in a not so good situation or not so good um, what do you call it, uh, uh, your, your state lah. .

Your state of being when you come on shift is not very good. You’re sick or you’re not feeling well, you’ve got pain somewhere, et cetera, you’ve been injured, whatever it is, you know that’s going to reduce your bandwidth then you should be hyperaware of that. So that, so with that in mind when you see cases and when you clear cases, you might have to either slow down a little bit or pay more attention to what you’re doing. . And you know, do that lah.

And if you really cannot stop check yourself, if you cannot have mental, mental you know, stop gates in place to check yourself, then you should come up with a physical way of, of stopping yourself lah. So you know, that can be things like having a, alarm system. I mean I used to do that when I was a registrar. I put an alarm on my phone every hour to ring because I couldn’t, I couldn’t mentally remember to check. .

00:48:01

So I just put alarm on my phone every hour so that it will ring and when it rings that’s when I stop and go okay, is there anything that I’m missing? Is there anything that I need to review and look at? Um and you just have to build it in and after a while it becomes a habit, and you learn how to stop check yourself and you don’t have to do it physically. But I don’t think there’s any issue with doing it physically.

And uh, also asking for help lah. Yeah, I don’t think there’s any problem with asking for help. I think people need to be comfortable with that even if they come up as a new specialist. Because sometimes it’s, it’s daunting to ask for help when suddenly people think of you as you exited already, you should know your stuff. But actually, it doesn’t matter, just ask. [Laughs].

MW What about Participant 14?

PA14 Uh, I agree with the asking for help bit. I think you know usually it’s, like I think I don’t know about other people but uh, sometimes when you jump a stage then you feel like oh, now I should know this, I should know everything. And people expect you to also know more things or they, I think sometimes people expect you to know everything because, just because you have exited.

00:49:04

And sometimes it’s hard because to ask for help also because then sometimes you are the most senior person on shift so it can be quite difficult. But then I realise that there’s always like, there are always people who have different, similar to what I was saying just now, people have different uh, strengths, right?

So um, for example, the inpatient specialist registrar may be a registrar but sometimes they also have specialist knowledge that you uh, you may not be familiar with lah. So there’s always room to ask for help. And if, if necessary then there’s always a consultant on call also to ask for help with. So I think asking for help is definitely something.

Of course, then, but I guess it, with regards to other cognitive errors, then I think I agree with Participant 15 that there’s uh, everybody has sort of, they tend to commit different errors. So I think there should just be a continuation of learning that should have started in residency in a way. Yeah. So I feel that it, it’s just a continuation or knowing what errors exist in the first place. And knowing which ones you’re prone to and knowing why or what situations you are especially prone to them.

And then I hope by then they will have developed some ways of sort of minimising the errors or coping or keeping track or something like that lah, certain strategies. And I think it’s just a lifelong thing. I don’t think, I mean I don’t think that I have, I don’t think that I’m new to cognitive errors even now. I, yeah, it’s just that I’ve maybe a bit more strategies to kind of deal with them. Maybe. Yeah. But it may not work for me, or may not work for other people, may work for me. So also, I guess it’s about being willing to be open to share um, you know, difficulties and, and try to learn from other people how they cope with similar difficulties and, and how they avoid cognitive errors um, that they face. I think that’s quite interesting as well.

00:51:19

So recently I went for a course overseas uh, where there was simulation and it was uh, you know, for specialists. And they actually made the scenarios very difficult uh, with a lot of interruptions, a lot of sort of social stuff to it as well. And then you find out that actually everybody, it was all specialists and, and everybody actually also struggles with um, trying to avoid cognitive errors.

Uh, and they struggle with, with similar problems actually. And it’s very interesting and I feel it’s very useful to know how other people deal with it. And then you have a little bit more ideas. And then subsequently then you can try it out and see whether it works for you. Yeah so I think being open to sharing about it is also a good strategy.

MW What were some of the strategies that they suggested or proposed or?

PA14 Um so they actually used those in way, a little bit more, uh, and also breathing. So I.

MW Breathing?

00:52:27

PA14 Interesting. Yeah, so, so they said that uh, you know, actually quite a few of them says sometimes they just slow down and then you just breathe like give themselves five breaths. Just count five breaths and slowly breathe five breaths and it just suddenly, things just become a lot like more calm they feel. Yeah. So it’s very interesting. And then like it’s so simple..

MW [Laughs].

PA14 I never would’ve thought about it.

PA15 Yes, actually the breathing exercise is actually mindfulness breathing exercise. A short mindfulness exercise.

PA14 Yeah.

PA15 That you can do in the moment. And there are many different exercises but it’s one of the things that is recommended. And I think if it works for you, it works and you should try it, yeah. So I also agree. Just slowing down in general is, is a good thing I think um, when you, when you’re trying to check yourself.

PA14 Yeah, yeah. And then also like the whole thing about um, being, so there was a, a, for example, there was a case where there were two, two teams of doctors and two patients. And then each team was trying to sort out their own patient and uh, nobody, nobody sort of, we noticed but nobody sort of uh, processed the fact that there were other confederate nurses and staff who started uh, quote-unquote, collapsing. And then they just kind of stepped over them to get the drugs themselves.

00:53:53

MW [Laughs].

PA14 [Laughs] yeah but then later figure out environmental exposure toxic kind of thing. Yeah. So definitely a cognitive error I feel [laughs]. They’re very focused on the patients and stuff but then uh, yeah so, so it’s interesting lah and it’s interesting to see that you know people are vulnerable also.

And then we talked about it and then we said oh, actually you know, we should, we should, we should say eh, how come like that side like you know, they are also having a res patient. And then eh, how come? Like there should be somebody who, who is stepping away and managing the both patients as a sort of overall, overall, overall in charge of the entire resus. Then that person would then realise that some nurses have gone missing. Yeah.

MW Thank you, thank you. Wow, that’s very interesting points. Um.

CF How about Participant 16? Um, I’m sure with your uh, even uh, one year of experience, there’s something to share with like incoming new AC. What do you think?

PA16 [Laughs]. Okay, I think AC just doesn’t really mean much other than salary increase by a lot. Um, the, the need to uh, constantly learn and this time around is different because after residency, residency is structured learning. Now is your own self-driven learning. Um, so have to create a structure for yourself to replace the weekly ENCC that is missing right now in the life.

00:55:25

And uh, I do personal reflections of every shift. Uh, what was down well, what was not done well. Uh, for me it works lah. Reflections. Some people with mindfulness breathing. For me it’s uh, self-reflection, a lot of on certain behaviours, certain feedback from people. .

Um, I think the most difficult part of being a new AC is when you have to supervise with such shift of someone who is merely per, probably half year to one year in rank junior to you. And now you have to juggle between their education and uh, the patient’s safety.

I find it the most difficult part and I don’t find that I’m equipped for that pretty much well. Because um, in resus, the, the instructions are given by the bedside to the nurses and the nurses act on it. So now this instruction come from the SR or the new AC [laughs], uh, then you will be have some conflict ah. Whereas in CC it’s slightly more time in P3 also slightly more time to play with the patient where in resus, I find it more challenging.

Um, uh, so uh, for that, I think being open and saying that I’m new in this supervisory role, uh, although I exited, I think um, uh, uh, to, to also be open about it that um, there are certain things I, I do is in patients’ uh, safety first and your education second.

00:56:51

Um, um, including uh, the other facts that uh, if I can’t handle it, then I ask for help. Ask for help is a, is a um, is a, is a, it’s, I find it’s a character of a good clinician lah. Uh, be, whether you are con, or senior con, you need help, you just ask for help. There’s no shame in it I feel.

Uh, yeah. So these are the few things that I think of lah when you say what is the new AC advice for the even newer AC [laughs]. Yeah. The learning part ah, is a, constant learning part. Now you have to drive your own learning. That’s the key thing. And patient safety is always first, uh, ego second. And um, yeah, that’s the few key points. Yeah.

MW Mm, then I think you all brought up um, several times awareness you know, awareness of one’s weaknesses, awareness of one’s limitations and all these are important. So, so do you think education regarding cognitive errors um, do you think it helps? Do you think it’s necessary? And um, if, if we really go down this path of education about cognitive errors, then when should it start? If at all. Yeah. The question is um, education regarding cognitive errors, is it necessary? And if it should start, if at all, then when should it start? When will be a good time to start?

00:58:18

PA15 I think it should start when you are in regship. When you become a new reg because that’s when the responsibility starts. Um, and you should, you need that time in regship to learn the strategies to deal with the cognitive errors that are unique to yourself. So you need that trial and error process. And you cannot do that trial and error process when there’s no one looking over your shoulder for patient safety. So it should be done in, in regship.

And yeah, uh, it should continue on but you, once you exit as an AC, I think you should con, you should do self checks like what everyone has mentioned. Some form of reflection. And some form of, of continued learning after you exit. Before you exit, definitely it should start. At the regship, at the very least at the regship level. If not even earlier.

PA16 I find it should start as a MO level. Um, HO still trying to learn the grounds of the public hospital system, how to work things around. MO level you are start, you are given supervisory role for the HO when they are on call already. You cut new cases and your differential matters and what is being done for the patient overnight. So I feel it should start young which is young in career, in MO level. Yeah because you are talking about cognitive biases, right?

MW Mm.

PA16 Form good habit, don’t write off patient [laughs], and then you know, oh all this a prone, I don’t care, a stone, I don’t care. Uh, that’s a very bad terms to give to patients lah. Yeah. So we should start earlier. .

MW What about um, Participant 14?

01:00:01

PA14 Uh, I think actually it should start early but then uh, it, I think it should be sort of contextualised to uh, whatever level that um, the audience is at lah. So I mean actually I think even HOs uh, house officer should be you know, they should be aware because actually they’re quite vulnerable also right? They are like tired all the time and they’ve many things to do. And sometimes um, you may pick up, you may miss things which you know, may actually be quite significant or they may accidently do something that may be potentially dangerous lah like medication errors and stuff.

So I think as long as they are in clinical practice. So maybe I would say at least HOs or uh, towards the end of medical school. . I think there needs to be some clinical exposure because otherwise I think none of it will make sense to them. But then um, once they are ready to step into the wards, I feel that there should be some education about it. But of course, the examples and the type of cognitive errors would, should be tailored to, you know, the more common ones that they encounter at their level.

And then of course as uh, as, as the other participants have said, at MO-ship then again, it’s a difference subset of examples. Uh, they take on different responsibilities, they clerk the patients overnight, sometimes in the ward um, I mean this is not just limited to ED MOs in the ward, uh, the ward MOs also uh, clerk patients and sometimes, that’s it, they don’t, the reg doesn’t see the patient if the patient is well overnight, right? Yeah so, so they do have some responsibility. Those, and, and it should be tailored to the situations they encounter.

01:01:47

And similarly as a registrar, and then finally uh, hopefully post exit, most of it would have been caught. I think as, I think most consultants uh, would know of these cognitive errors. They would know what the cognitive errors are and I think hopefully most of them will know which at least a little bit of which ones they are a prone to and already formed some strategies to prevent them.

But I think that as I was mentioning, maybe it’s sort of quote-unquote education is more tailored towards strategies to avoid it lah. So I think it can start early but it depends on how it’s being delivered. Yeah.

PA14 Yeah, I think um, because I think some of the medical schools are already teaching about cognitive errors.

MW Mm.

PA14 But I noticed that the, the, the attitude towards this topic of cognitive errors at the medical, at least from what I’ve seen, at the medical student level versus at the MO level, versus at the reg level, um, it tends to be dismissed quite easily I think at the medical student level. It seems to be something that’s mentioned so often to the point where they just like oh, yeah, yeah, yeah, premature closure, yeah, that’s right.

Then I don’t, because I think it’s they’ve not had the responsibility or that, that experience where you know, it has happened before. The fear or something like that. So they are kind of protected by this you know, air or naiveté or ignorance in that sense.

01:03:17

So I, I wonder whether or not if we, if we start the conversation too early, or rather if we present it in a way that you know, we keep talking about it, it becomes something like, like they’re just being nagged about it. Whether they tend to forget it and just automatically switch off. It becomes like the screaming patient in the corner, right? The school and the consultant and everyone is just screaming about cognitive errors and then they’re like yeah, yeah, okay, okay, it’s not going to happen to me, it’s going to happen to someone else. I always thinks about it, that’s when you come up with four differentials.

But they don’t, they still, it doesn’t seem to, there’s no connection between I might make a cognitive error therefore I should come up with three differentials and I should challenge each differential. Or challenge myself to, to prioritise a differentials. It doesn’t, this connection isn’t there.

And then at the MO level, um, you pointed out to them but a lot of times at the MO level, they are also like oh, okay but anyway the diagnosis is not I make one, it’s the boss make one. Or you know, the plan is not I make one, boss make one. So this doesn’t apply to me either.

So I find that the, the time when it is really taken seriously is actually at the reg level when suddenly they are saddled with the responsibility and then we realise that oh shit, I have to do it and now this thing becomes intimately important to me and then they pay attention.

01:04:32

But I, I have noticed that the attitude to it at the, the medical student level tends to be pretty frequent. At the MO level tends to be like oh yeah, okay but it doesn’t apply to me, a bit, a bit nonchalant about it. And it’s only at the reg level when people start freaking out. I mean yeah, so I don’t know whether or not there’s a side effect of teaching too early or whether it’s the way it’s presented.

MW Thank you, thank you. I think those competing points are quite um, quite interesting. Um, co-facilitator, you got any?

CF Um, no, I don’t have any further um, questions.

MW Um, okay. So uh, participant, I’m just going to ask a few uh, random questions, sorry. So Participant 16, earlier you mentioned um, you try to do reflection after your shift, right? To see where you could have improved um, where uh, what, what things you could have done.

Then um, how do you, do you know that there’s a cognitive error when you have made it? Like is it apparent immediately after the shift? Or, or usually it’s somebody after that come back and tell you, you know, um, there was an error made um, and then you, you think back, you know? But, but, but how do you reflect if you don’t even know that? Because the error may not be that obvious at the time as well.

01:05:54

PA16 Yeah, correct. So, so uh, sometimes uh, after the shift doesn’t mean immediately after. You trace patient after you admitted and you see what happens to them. Sometimes um, you receive feedback from faculties who are more senior that you then they will tell you things. I think it’s not just uh, immediately after a shift lah. I mean, after a shift. Um, so I mean resus shift then of course then you will reflect what could have been done better for that patient, that tricky case that was done.

So again as I mentioned, you don’t know what you don’t know so it’s very difficult to, to, to just use self-reflection um, to cover all grounds. You still need to look at other people’s cases, look at other, how people handle things to learn along the way.

Um, and you also rely on feedback. Um, uh, feedback from seniors are common but feedback from juniors are very uh, uncommon in our culture. Um, no MO dare to tell you that you are not a very good or clear, clear senior versus another senior who give me if A you do this, if B, you do this, you know? No juniors will dare to tell you that lah.

So, so feedback from seniors most of the time but the, the interesting thing to explore would be if juniors are able to give us feedback on how we senior clear cases with them. How would they have preferred us to do certain things? It might even help more um, in terms of self growth lah for a new AC. Yeah but it’s not um, it’s something to think about lah. But I haven’t found a way how to ask junior eh, what you think of my [laughs], yeah.

It’s a bit weird though, yeah. But uh, feedback from seniors come spontaneously. Um, self-reflection can only cover things that you know. Um yeah. And through tracing of results, tracing of patient, whether patient return and all that, then you also learn along the way.

01:07:49

MW I think you mentioned uh, this thing about culture, right? That um, I mean just, just bringing it further right, do you think it’s peculiar to emergency medicine this culture? Where, where in emergency medicine everyone is you know, we expect that people, we expect that emergency physicians should be able to process cases quickly, that we should be able to chop chop, admit the patient? And then we try to boast ACON, I mean the, the time that we took to process this patient was ten minutes only, we admitted within ten minutes, that kind of thing?

So is it a cultural thing that is very peculiar to emergency medicine or, or um, um, or do you think um, um, um, actually um, emergency medicine may not I mean, that actually predisposes us to um, cognitive errors?

PA16 I think it’s part of job description. You expect a surgeon to be able to do appendectomy let’s say in one hour. You expect an emergency physician to be able to pick up undifferentiated patient, the key life-threatening issue, whether it be save a life or save a limb. Um, that’s where we add the most value, being to, being able to suss out what is the issue of the patient uh, urgent and critical issue of the patient within the, within the limited investigation we have and limited time we have.

01:09:07

The, as to whether we do things fast enough, is part of the character of being a emergency physician. Part of the JD. If you are slow and you take history like a two-hour geriatrician, then you won’t be in e-med already mah. [Laughs]. It’s just like a surgeon, you, you, if you can’t do appendectomy in one hour then, then what you, how do you call yourself a specialist lah? Yeah. It’s part of your job description and this is what you are being paid for. Like the organisation to do, yeah.

MW But some cases like you say are undifferentiated right? Some cases would require more time in a sense like.

PA16 Yes, correct but you, you are supposed to be able to suss it out lah. That’s what you value add to a patient in his initial journey to the hospital. You are supposed to suss it out uh, what is this undifferentiated patient. I mean there are certain clear cut cases like if you have block, require dialysis thing, yeah, those actually should be taken out of emergency medicine. It’s a system issue. It should just go straight to some uh, some renal clinic or something like that lah, to just fast track. Because there’s not value add at all.

Whereas resus cases are the one that we actually value add. Critical care cases as well. Yeah. So, so that’s what I think um, is our job description. Yes, it’s an expectation and I don’t think it’s unfair, yeah.

01:10:24

PA15 Wait so what is the question actually? Are you asking about the culture? What, what is the culture in emergency medicine or are you asking is the culture of efficiency or the culture of you know um, just doing everything as quickly as possible or getting the work done it the shortest possible time a culture unique to emergency medicine?

MW Um, my, my, the question is uh, because in emergency medicine we have a culture of doing things as quickly as possible. So is that going to help cognitive errors? Because I mean um, is that um, is that a good thing to have? I mean is that something that uh, we cannot, we cannot change in that sense? Or um, is that something that we should change because um, it will affect cognitive errors?

PA15 Okay but I don’t really think that um, the culture of emergency medicine is doing things quickly. I think the culture of emergency medicine is um, maybe to, I think the crux of emergency medicine is resource management and risk management rather than completing something quickly and stuff like that. Whichever part of the department you’re in whether it’s in resus or, or in um, other places. If you’re talking about what is unique to emergency medicine, compared to elsewhere.

Because if you’re talking about resuscitation and stuff like that, there are other specialities that also do resuscitation. I mean I think it should be the top at resuscitation obviously but um, I don’t think that’s completely unique to emergency medicine.

01:11:55

But what is really unique to emergency medicine is the ability to handle a, a you know, a large number of patients or large number of issues with limited resources. Essentially you know, a mass-cas every day. .

MW [Laughs].

PA15 I think and then risk management as well. So I think that culture actually doesn’t, if we’re talking about risk management as a culture, I, I actually think that it doesn’t predispose to cognitive errors, makes you more aware of the errors that can happen. But unless the, okay, this perspective that emergency medicine is about risk management, it’s not common, I think. That’s my personal perspective lah.

Um, resource management-wise, yes, I think resource management if you, if you cannot mange resources well, uh, or your um, yeah you can’t manage to manage resources well or you’re so, you’re trying to manage the crowd more than the individual patient, the idea of having to try and manage a crowd is going to predispose you to cognitive errors because you’re going to lose sight of the patient.

So I think if it’s, you have to be aware of the resources that are required for each situation that you’re dealing with. Because emergency medicine is about dealing with multiple different situations that’s happening at the same time. . Right? So if you’re in resus, then you are managing resources, not just in resus but you’re imaging resus for the individual patient times six panels. And then that’s the same as when you are in CC or when you’re in P3. You are managing resources for an individual patient but times how many patients there are at present over there lah.

01:13:36

But at the same time, then you need to recognise from the third-level like a meta cognition level where how, how much resources are you sinking into an individual patient and how much uh, how much are you depriving another patient of those resources.

So I think that struggle if you cannot, if you are, especially when you, when you start, just start learning about how to manage those resources, um, if you’re not aware of what’s going on, if you cannot keep bird’s eye view of the whole thing, then you, or you can be sucked into an individual patient, or conversely on the other extreme, you don’t care about a single individual patient, then yes, uh, that, that idea of having, that stress of having managing resources can predispose you to cognitive errors. But I think the, the culture of risk management actually helps to avoid that.

MW Mm, anything to add uh, from Participant 14?

PA14 Um, yeah, I, actually I agree with although they seem to be differing views, but actually they are both true. Um, both views that have been brought out, I feel um. Being quick to act I think is part of emergency medicine. But actually I think it’s also um, the hallmark is that we as emergency physicians, we are able to recognise what is urgent and, and perform if required urgently. But also to recognise what is more not urgent. And recognise what um, is not uh, usual. Um, yeah.

01:15:13

So I, I think it’s not, it’s not just, the culture isn’t that we just, we are just fast, fast, fast, fast, fast. We can be fast when we need to and when we recognise the need to. Uh, but we also recognise that the need that you know, actually some things don’t need to be fast. So it’s about triaging I feel in a way and being able to then deliver according to uh, our priorities. And also unfortunately there’s also the whole resource management thing which uh, participant uh, 15?

MW 15.

PA16 Yeah, brought up. So, so I, I don’t really, I think um, of course sometimes because they are competing sort of um, priorities and also I guess we are all not perfect, uh, sometimes we, we go into, we tend to lean one way or the other. Sometimes we lean into trying to be too quick to act about everything, even stuff that doesn’t require urgent sort of uh, urgent action. And then we overload ourselves and hence cognitive errors happen, et cetera, et cetera.

So sometimes I think it’s just a, a try to walk that line is difficult lah, to always be perfect and walk in the exact right balance is difficult. So I think when we lean towards being too frazzled and too fast about everything, even when it doesn’t require that. Then yeah, then that’s where cognitive errors happen. But then also you know, when we are too like ugh, the queue is so long, we can’t do anything about it [laughs], then also sometimes errors happen lah, yeah.

01:16:51

So I, I think it’s just it’s, it’s not, there’s no, I don’t think there’s a culture that will be perfect actually, you know? It’s just what is required of the job and then trying to sort of handle the side effects of having that sort of uh, culture. So it, it’s about picking the culture that fits best for your job description. Uh, and for your department and for what you need to achieve. And then recognising that no matter what culture, at the end of the day you pick or you adopt or we adopt, there’s always going to be a sort of a flipside to it and then trying to manage it subsequently.

MW Mm. Uh, co-facilitator, any other things to add?

CF Uh, no, I don’t have, yeah.

MW Don’t have. Okay uh, so thank you everyone for your time.

01:17:40
