# Supplementary material for: Dropping the baton: Cognitive biases in emergency physicians
Source: PLoS One. 2025 Jan 2;20(1):e0316361. doi: 10.1371/journal.pone.0316361 (PMC11694980; doi:10.1371/journal.pone.0316361)
Supplement: S3 File — (ZIP) [file pone.0316361.s003.zip › Transcripts/FGD 5.docx]

FGD 5

Speaker Key:

HO Host

CF Co-Facilitator

P Participant

00:01:24

P17 Um, I’m participant 17. Um, my experience as an emergency physician is less than, uh, five years. Yeah.

HO 18?

P18 Um, I’m participant 18. So less than five years as an emergency physician.

P19 Hi. I’m participant 19. Less than five years.

HO Okay. Great. Thanks. Um, so just as an introduction, uh, we would like you to think of some of the medical errors that have been committed, that you know of, that have been committed by emergency physicians or even yourself after existing as a specialist. What are some of the common factors that lead to medical errors among emergency physicians? What do you think? What are the… What are some of the common factors that lead to medical errors that you have seen? Anyone can start.

P19 Um, hi. I’m 19. Um, off the top of my head, uh, if let’s say a patient comes in with a GP letter, and then the GP says that, you know, he’s concerned about this, and this is his, his, uh, his differential diagnosis, then I might anchor on that, and then I will not go through my own list of differentials lah. Yeah.

00:04:40

So for example, if I were to give, uh, a real-life case. So, uh, one day I have a patient who, who was referred to me by a GP for, um, gout of the ankle, and then, um, the foot did look, uh, quite red and swollen, and then there was a bit of, uh, acute kidney injury. So the GP also wrote, concerned that, you know, the colchicine made her vomit, and then she has dehydration, and then AKI.

So, so, uh, I, I, I, I thought that it could also be cellulitis, um, but then I also kind of, uh, fixated on the colchicine causing vomiting, causing AKI part. But then, eventually, what turned out was that the patient actually had nec fasc. So the pain was, uh… Um, maybe I could’ve given more, um, emphasis on the pain, and then, uh, thought deeper about why her, uh, bloods were so deranged, you know. Actually it was just severe sepsis lah.

HO What do you… Why do you feel that, uh, the, the GP’s letter made such a big impact for, for this case?

P19 So sometimes, uh, because actually I was doing a, uh, stepdown P2 shift, and it was pretty busy. So when you have a letter, and the letter actually gives you an idea of what the patient has, then sometimes to… You know, you are a bit rushed on time, and then, uh, the whole thing just contributes to, to, to, to you fixating on one issue lah, instead of, you know, going through the whole process, asking very detailed history, starting from the beginning, calling the daughter, etc., etc. Yeah.

00:06:27

HO Thanks. That’s a great sharing. Any of the other two participants?

P17 Um, so I’m participant 17. Uh, maybe I can just share a few of some of my examples. Um, so similarly, there’s, um, like anchoring biasness. Sometimes there’s this bit of a diagnostic momentum that forces me to think of a patient who have a certain diagnosis and totally missing the more obvious diagnosis lah.

Um, an example of this is that one of the patients that, uh, I, I helped to see, uh, apparently one of the senior resident thought that this could be an intestinal obstruction. So when I ultrasound the patient, I did find a large hollow viscus. So, uh, based on the anchoring bias that I had at the time, I thought this patient has… Yeah, looks like an IO. But it turns out to be a ruptured AAA. So I, I think part of the diagnostic momentum, that, you know, keep thinking that, oh, this is IO, this is IO, that totally miss the AAA diagnosis lah. Yeah.

Um, other things that, uh, I can think of is also to do with like premature closure. Sometimes, um, when you are very satisfied with a certain diagnostic case that confirms your suspicion of a diagnosis, you just stop there. So another example that I can give is that I had this patient with, uh, large vessel occlusion stroke, seen on CT scan.

00:08:03

So actually the CT multiphase covers all the way down to the root of aorta, and we totally missed the dissection that’s there. So subsequently, the patient was given RTPA and transferred to SGH. Um, but in doing so, because of the RTPA, the patient subsequently died because, uh, there is a massive cardiac tamponade as well. So, uh, also one of the things. Yeah.

Uh, I, I think other, other factors is also like ED is a very highly, uh, time pressured environment. Uh, there’s a lot of distractions as well. Um, so examples of being distracted, causing medical errors, is like, uh, usually, they’re medication errors. So, uh, if we check drug allergies with the patient on, on, on the system and administer the medicine without ordering on CPOE.

Um, other things is also like, uh, for procedures itself. Uh, while being interrupted in the middle of a procedure, may not have done certain steps which is, uh, to ensure patient safely lah, uh, and, uh, eventually, something went wrong, yeah, and patient has an injury. Yeah.

P19 Mm, maybe I’ll also add to the… Because the ED, sometimes we do a lot of handovers. So then like, for example, the junior hand to the senior or the consup (consultation supervisor) and then in between shifts, a shift hands to another shift. And then, uh, when things… When information gets, uh, passed on, sometimes like broken telephone, right? Then either information doesn’t get passed on or the wrong information gets passed on, and then you just assume whatever the person say was correct..

00:09:44

Yeah. And then because of, you know, the time constraint, the high pressure, you don’t go and check it thoroughly yourself. Yeah. So that’s another contributing factor.

HO Okay. Participant 18, anything to add?

P18 Yeah. So I’m participant 18. Um, so I think one of the personal experiences would be related to drug allergy. Um, so there, there are many ways to confirm if the patient has a drug allergy or not. So I think sometimes, um, we rely on one way that is the most convenient to us, but there are, um, other ways to confirm it.

So example. A personal experience. Uh, I saw this patient in a P2 shift, where I asked the patient, okay, do you have a drug allergy? Because the patient, uh, patient is quite new, doesn’t have any other medical records in the system, and he said, no. And I did serve the patient, um, some Panadol myself. When I reconfirmed, are you allergic to any medicine? He said, no. But, um… So anyway, the patient got discharged with some antibiotics and, um, later had an allergic reaction to it.

So what happened was the patient is known to a GP, a regular GP, and the GP did write a letter through, which I glanced through. I did, uh, get the, the complaint, and I think it was cellulitis. I can’t really remember. Uh, but something on the, on the line. Um, but there, he mentioned that the patient has an allergy, but I missed it because I’ve already asked the patient, uh, twice.

00:11:28

And then when I asked the patient on, on, uh… After calling him back, he’s also not very sure about the allergy status. But I, I, I did have something on the record that’s saying that he has an allergy. So, yeah, double confirming on that.

So number two would be, um, related to procedures. Um, double checking the patient’s, um, identity, and then also the size and the side of injury, etc., um, which, which we usually do very strictly in the OT room. But then in the, in the ED, if it’s like a procedure done by ourselves, um, I think it’s, it’s quite murky, and especially if it’s not your own patient. Example, uh, patient, uh… The procedure done by the HOP (handover and procedure doctor), and the patient seen by another, uh… Other medical officer, or a senior resident, or an EP.

Uh, and the other thing is when the junior ask you for help. So example. Uh, another personal example of recent. Um, my MO came and said, okay, the person for the MNR of the Colles fracture is on, on the, on the table, on the bed and ready. So I, so I walked in, and then I started talking to the patient, um, but I didn’t ask for the name. Uh, so, so this patient was also there for an MNR of the… Of a Colles fracture, uh, but it was a different hand, different site.

So only when I glanced down and I saw, oh, okay, this is the left hand, I was supposed to do the right hand, then only I, I asked for the name, and it wasn’t the patient. Yeah. Yeah. So I think that’s all. Because we are very busy. Like I had like about two or three. So I just wanted to get everything done on time, before I handover, because it’s not nice to handover procedures, so… But then, yes, um, yeah, so…

00:13:42

And then, uh, the other one would be, uh, premature closure. When you get handed over cases with, um, with plans or like some, you know, possible DDs (differential diagnoses), um, that MO also… The, the, the juniors also may be fixated on, oh, this is this, and then we also might get fixated, because it’s already discussed, or there’s already a plan, uh, drawn up.

And especially for people with dual pathologies, you know, because, uh, one would be, uh, handover case, where the patient was told to have, okay, have two issues. One is appendicitis, and two is, um, some leg cellulitis, also the right leg. But the, but the pain, everything, was in the foot, ankle part.

Um, so I, I, I was like, okay, so I was waiting a CT scan. I, I was… I saw the patient when the patient was being examined, and there was RIF tenderness, so I let it be. But then when the scans and, and, uh, things came up after many, many hours, she had like a psoas abscess going down to the leg, etc., and it was like, um, many hours later that… I mean, I think it needed further investigations, but then I, I didn’t even think of any other possible diagnoses at that time. Yeah.

HO Uh, so I just want to, uh, clarify something. Just now you… Uh, just a term. You mentioned a HOP MO. What is a HOP (handover and procedures)?

P18 Oh. Uh, MO doing procedures separately. So that, uh, that, that person’s, uh, is to do procedures only. We’ll get patients referred from… I mean, the, the primary providers who do… See the patients will send the patients over to this person to do the relevant procedures.

00:15:58

HO Okay. Okay. Thanks for the clarification.

P18 Okay.

HO I think just now somebody mentioned, uh… P17, you mentioned about… You, you brought up a lot of terms. You said, uh… I think you mentioned anchoring. You mentioned diagnostic momentum. You mentioned premature closure. I think just to be… Uh, to have everybody on the same page, would you like to just let us know what you mean by those terms? What’s, what’s your understanding of those terms when used, when you mentioned them?

P17 Oh. Um, so premature closure is basically, uh, there’s… Basically, when you haven’t completed the, the entire search for other causes or other problems of your patient. You’re just very happy with whatever you find, and then you don’t look at the bigger things, and you miss out other, other aspects of the case lah.

So the example I gave in this case was a patient that presented with an altered mental state, for which we did a CT, uh, brain at multiphase for this patient. We found a large vessel occlusion. Uh, we were satisfied to say that this is a patient with stroke. But if you were to complete the entire search or, or examination of the renal imaging, we will have noticed that there was a Stanford A dissection as well. Yeah, so that’s premature closure.

00:17:26

Um, anchoring basically means that you are very fixated on a certain aspect of a case, like the diagnosis, for example. Uh, in this case, I gave an example of a patient with a ruptured AAA, with the initial, uh, differential being, uh, intestinal obstruction. Uh, because I’m anchored on the fact that this patient has intestinal obstruction, when I see a large anechoic tubular structure in this patient presenting with abdominal pain, I will think that this is a dilated intestine, rather than abdominal aneurysm. Yeah. So that’s like anchoring.

And because the diagnostic momentum is towards intestinal obstruction, the subsequent management of this patient relate towards intestinal obstruction. Um, so, yeah, we, we didn’t come back until the patient deteriorated further. Yeah. So that’s what… Yeah.

HO Thanks. Thanks so much for the clarification. And also thanks, everybody. I, I realise that everybody was sharing their own, you know, even personal cases. So thank you for the honesty, uh, and willing to… Being willing to be vulnerable. Uh, we really appreciate that.

Um, the next question that we wanted to ask was how much do you think cognitive… How… To what extent do you think cognitive errors play a part, uh, in emergency physicians committing these medical errors? So to what, to what extent do you think cognitive errors play a part? Because we mentioned a lot of factors just now about what are the common circumstances. Um, so now the, the question is, to what extent do you think cognitive errors contribute?

00:19:43

P17 Uh, maybe I start first. I’m participant 17. Um, so I, I feel that, uh, even though there are many other factors, uh, like time pressure, like patient not being forthcoming, or, um, distractions, uh, in the department itself, uh, I find that the final pathway is still, um, the discipline to actually, you know, be self-aware of all the cognitive biases and blind spot that the EP has lah. So I would say that, uh, it plays about maybe up to 50%, you know, of the cause of errors, ultimately.

P19 Yeah. Hi, I’m 19. Uh, I would agree that, um, cognitive errors do play a large part in medical, uh, errors in the ED lah, because the ED environment is so predisposed to, um, us committing errors, because we don’t have the luxury of, you know, taking our own sweet time.

Like, for example, every, every bed in a ward has a ward number. In the ED, you just put one trolley there, and then the trolley is like… You have to go and find a number and put there. So we have… All these things will contribute to, say, uh, wrong patient, you know. So the environment already is so risky. And if you’re not aware of your own, uh, cognitive, uh… Of your own, uh, capacity for cognitive error, then it’s very, very easy to just fall into that trap.

HO So you would say a large proportion of it, of it is due to like external circumstances, or you feel that a lot of it is due to cognitive errors, or it’s like half, half?

00:21:42

P19 I feel is, I feel is probably half, half, but the… It just builds on each other loh. So you have, first, you have the environmental thing that sets the, the… Sets a very fertile ground for you to commit cognitive errors.

P18 Hi. I’m participant 18. Um, so I agree with, um, what was said by the other two participants. I think, I think it’s around 50%. There’s a lot of, um, other external factors also. But, um, yeah, cognitive, um, factors, probably around 50%, from what I think.

HO Okay. Okay, so any, any, any questions from the facilitators at this point? The other… The co-facilitators.

CO1 No, none from me.

HO Okay. Okay. So, um, it’s interesting because we… Just now participant 19 said, oh, you know, the… We don’t… We cannot take our own sweet time to, to see cases, and then the, you know, the beds keep popping up, and we, we have no control over the, uh, the, the incoming load, workload. So the, the thing is let’s say if you had an ideal environment where you could take as much time as you wanted to see the, the A&E patients, do you feel that the… Then we would be able to get rid of the, uh, cognitive errors?

P19 Hi. I’m 19. So I think that, of course, you can never absolutely remove, um, cognitive errors. But with luxury of time and a more controlled environment, then, um, the risk is lower lah. So like, like maybe if I were to give you an example.

00:23:54

There was one case there was in extremis in respiratory distress, and they wanted to intubate her. So let’s say this patient was like in OT, and it was an elective case, then, you know, you will go through everything. You will have the anaesthetist do their airway assessment, assess the patient, come down and do a preop, etc., etc. But here, we had to compress all these things into like five minutes, and then we ended up not noticing that actually she was a tracheostomy patient, and she had a laryngectomy before.

So we went to try and tube her, and obviously we failed lah. And then only after we failed, then we realised that… We pull down her shirt then, aiyoh, there’s a hole in the neck. Yeah. So the patient didn’t have a good outcome..

But then I think maybe this might illustrate what, what I meant, because if you have time, then you can actually go through everything. And by checking and by having, having systems in place lah, like, for example, checklist and, and, and… Yeah, so then this will, this will kind of eliminate the human factor that, that leads to cognitive errors.

P17 Um, hi. This is 17. Um, so, uh, I, I think there are other aspects, rather than just time pressure and acuity of cases. Um, ED is after all a place whereby all patients are rather undifferentiated. So sometimes, uh, it’s a bit difficult when we evaluate patients, uh, because of the atypical presentations that they have, or because of things like diagnostic momentum and anchoring biasness, um, that actually steers us in a, in a way that, you know, we cannot escape our cognitive errors lah for these patients. Yeah.

00:25:41

So I still think that despite time… Uh, eliminating of time pressures, more controlled environment, cognitive errors are still, uh… Can still play a part lah. Yeah.

P18 Yeah. Hi. I’m participant 18. Um, so I think at the emergency department, it’s, um, very difficult to have like an extremely controlled environment. But even if you take like, uh, a lot of factors out, like, um, time pressure, etc., you still will have undifferentiated patients, uh, without previous, um, workup or diagnosis made. And also you most likely will be juggling at least two or more patients at the same time.

Um, and so, um, however we try to control the external factors, I think cognitive factors will still, uh, play at least 50% of, um, yeah, the… Will play at least half, half, uh, when it comes to medical errors.

HO Yeah. So co-facilitator two, you raised your hand.

CO2 Yeah. Um, thank you all for sharing. I was just wondering, um, if we just give you a scenario, for example, if an alcoholic comes to the department and, you know, and, um, and, uh, as an example lah, um, and one of the, uh, physicians who attended, you know, um, sort of like just treats him as a regular patient and, and, um, and then, you know, maybe lodges the patient overnight, and the next day there’s a bad outcome, something was missed, um, why do you think, you know, um, that could’ve happened?

00:27:44

Uh, you know, or do you think that can happen at all? And if it does, and if you do think so, then, you know, you know, why do you think that this particular action can happen?

P17 Uh, so I’m participant 17. Uh, so, um, your example, alcoholic, uh, patient came in, um, and the next day has a bad outcome, after being lodged. So can I just assume that this patient basically presents with drowsiness or altered mental state that we think that it’s alcohol intoxication, and therefore, uh, we just lodge and wait for sober?

CO2 Yes. Yes. You can say that, yeah.

P17 Okay. Okay. Yeah. Yeah. Okay. Um, so I, I guess it’s, it’s a little bit of repeated pattern recognition and a bit of premature closure as well. I can see how it can happen. Uh, basically, we have a possible diagnosis, right? That, okay, this patient has alcohol intox (intoxication), doesn’t look like there’s obvious head injury. So this kind of explains why he’s altered. Therefore, I decide to just sit on him because I do not have… I, I think there’s a bit of obstacles to doing a CT brain for all alcohol intox patients, right?

So I guess in, in a way, that could happen. Um, how to mitigate this? I suppose more frequent re-examination of the patient. But again time pressure, premature closures can be a bit difficult. Yeah. So I, I, I can see how that outcome could have happened ah. Yeah.

00:29:19

CO2 Yeah. So, uh, besides premature closure, do you think any other factors may play a part, you know, in, in somebody, you know… Perhaps not yourself, but, you know, someone, you know, seeing a frequent flyer come into the department, uh, and, uh, not doing a full investigation, but just, uh, maybe observing the patient, that kind of thing.

P17 So I, I, I also believe that, uh, there could be an element of, uh… A bit of, uh, biasness towards these patients because of their social setup, because of like personal beliefs that, you know, these kinds of patients… Not to, not to say that everybody feels the same way, but, uh, can be a bit, uh, use up ED resources a bit unnecessarily sometimes lah. So there’s a bit of, I guess, social judgement, yeah, that’s affecting the, um, cognitive thinking of the physician. Yeah.

CO2 Okay. Thank you. Would anybody else want to, uh, weigh in on this or say anything about such an example of a patient?

P19 Hello. I’m 19. Yeah, I think, I think this is not… May not only be, be limited to ED physicians lah. But I think we all are human, right? And therefore, we all have our own, uh, biasness, and we judge patients also. So, um, if, let’s say, a patient keeps coming to ED for the same thing, and you are seeing him for the tenth time, then, you know, uh, there’s not much, uh, motivation for you to go and re-examine him and, and, and redo your whole list of differentials, when he has already come in for nine times before, and it’s always alcohol intox..

00:31:09

Yeah. So it’s, um… I think it’s a human thing lah, and it may not only be limited to EPs. But perhaps in the ED setting, it might be a little bit more relevant, because they never get past the ED door most of the time, if they are not seriously ill. It’s more evident lah, ED.

CO2 Thank you. And, and any… Would participant 18 like to add anything?

P18 Yes. Yeah, I’m participant 18. I also think, um, yes, there is, um, the biases, the premature closures which comes to play in this. But also when it comes to… Other external factors also play a role. Uh, meaning this patient obviously needs to be reviewed at least a few times before he leaves the, um, the emergency department, or we make a final conclusion that this is just alcohol intoxication and nothing else.

So that takes up quite a lot of time, and, and it may be overlooked because of all the, the busyness, the time constraints, etc. Even though that the review was planned and known that needs to be done, it may be pushed back. And when you are faced with like five or ten tasks, it probably will… May be put as a lower priority than the other tasks at hand. Also contributed by the, the judgements, the biases, etc. Yes.

CO2 Okay. Yeah, thank you. Uh, just another scenario, a, a slightly different one, which is, uh, if a, for example, a patient has been seen already and has been, uh, diagnosed as pneumonia.

00:33:04

So then after that, uh, and the CURB score is high. But yet the physician may decide to not admit to a higher, uh, monitored bed, uh, you know. But in the end, because the patient was admitted to a, say, a general ward, the patient had a bad outcome. Do you think, you know, this could also be a cognitive error, uh, or something else? You know, a, a decision form of error.

What do you think was in the… Might have been in the thought process of such a person, if you feel that sometimes you may be tempted to, uh, do something similar? So instead of admitting a person with a high CURB score, uh, to monitored bed, uh, decides to admit, you know, to, say, a general ward, for example.

P17 Um, yeah, so I’m participant 17. Um, so, um, I think sometimes, uh, while scoring system is there to help us in determining, um, acuity of care, sometimes when we look at the patient, they look rather well, vitals otherwise okay, we are very tempted to take the mental shortcut into admitting patient into a general ward, right? Partly because of the difficulty in logistics and trying to convince the inpatient colleagues that this patient does require more monitored setting lah. Yeah.

And if you find that it’s very difficult to convince ourselves, sometimes it’s also a bit hard to convince the inpatient team. So there’s a little bit of, uh, too much obstacle, so I’m just going to take the path of least resistance. . I think that’s, that’s, that’s how I can see this happening lah.

00:35:26

CO2 Thank you.

P19 Um, hi. This is 19. I think everybody has different risk threshold, and their risk threshold is probably shared by their prior experience of whatever clinical patients they have seen before. So, um, I mean, you know, if somebody probably is maybe very senior and has seen many of such similar cases, and in his vast experience, he feels that, oh, I think this patient looks okay, and then he may be a little bit more of a risk-taker. Or else maybe somebody who is a bit more junior, uh, he might want to, you know, play, play, play safe a little bit more lah.

So, um, I, I guess in ED, you’re always struggling with decisions as to where to right-site your patients, right? So if, let’s say, you are senior, and you have more, uh, bravado, then, uh, you also at the same time conscious of the bed situation, then you might want to go ahead and take the risk. Yeah.

P18 Yeah, I’m participant 18. So I think, yes, um, experience plays a big role in our… In making decisions like this. And also despite, um, the, the scoring systems, uh, we, we always assess the patient clinically as to how they are. Uh, and also in the ED, we might, um… How they respond to our treatment as well. And also, um, the bed availability upstairs, and how loaded, uh, the upstairs, um, beds or people are.

Um, so I think all of this will, will probably make, uh… Play a role in, in how we decide to, um, send our patients to a higher or… I mean, lower… Higher acuity bed, or whether it’s a general ward, with the early review, etc.

00:37:34

P19 Sorry, can I just, um, qualify my statement earlier? I’m 19. So seniority, seniority can go both ways, right? So if you are senior, you might be a little bit more a risk-taker. But also if you are senior, and you have seen people deteriorate, then you might be a bit more careful. If you are junior, you must be more safe. Or if you’re junior, and you don’t know enough, you also can take unnecessary risk. So I’m just saying that seniority and your prior experience come into play, but of course, uh, different people might respond differently lah. Yeah.

CO2 Thank you very much. Back to you.

HO Yeah. Yeah, so, uh, participant 19, I was, I was going to ask you whether you felt that… You know, what’s the relationship between, uh, your risk appetite and, let’s say, uh, your… Versus your skill or versus your experience level. I think you, you’ve clarified that for us. Uh, anybody else wants to weigh in? Do you feel that, you know, is it risk, risk appetite is proportional to years of experience, or risk appetite is proportional to… What, what are the factors that, that affect…

P17 Uh, so I’m participant 17. Uh, I think there is a curve somewhere. Uh, the Dunning-Kruger curve or something. Yeah, so…

HO What, what is that?

P17 Uh, so it’s actually, uh… It’s basically a curve that talk about confidence and competence, right? So if you realy know nothing, and you’re very confident about yourself, then I think your risk-taking appetite is very, very high lah. Uh, then, of course, as you progress and you realise that, you know, there’s… Your competence is not so good, then your confidence start to drop, then your risk-taking appetite becomes very, very low.

00:39:27

Then after you pass through this stage, as you build more and more competence, you know, then your confidence also build up, uh, proportionally lah. So your ability to take risk also increases lah. So yeah.

HO Okay. That’s, uh, that’s, that’s interesting. Thanks for introducing us to this, uh, curve. Uh, I think that was a good, uh, segue to… And then thanks to facilitator two for bringing up some vignettes to, to further illustrate points and bring, bring up more, more discussion, more, more, um… Generate more points. Yeah.

Um, okay, so we are just going to move on to the next question. Uh, would you… I think we already shared quite a few examples of, uh, cognitive errors leading to medical errors. Uh, just going back, uh, to the original point about cognitive errors, would each of you like to share with us briefly, what is your understanding of cognitive errors? So to you, what is a cognitive error?

P17 Uh, I’m participant 17. So maybe I’ll start first. Um, so I think cognitive errors is whereby, I mean, there are some thought processes that don’t really match up with, um, what is really happening, without us realising. So, uh, that, that makes it very [unclear] to actually make mistakes. Yeah. Yeah.

00:41:24

P19 So I’m 19. Uh, to me, a cognitive error is a way of thinking about a problem that, uh, might be coloured by external factors, uh, that influence your thinking in a… Such that your… The, the, the solution you arrive at is, uh, inaccurate lah or, or not correct.

P18 Hi. I’m participant 18. So, to me, I think cognitive errors mean, um, something, you know, uh, like our thought process which makes a decision, uh, is, um, doesn’t match what’s really going on. So like you have enough knowledge, etc., to, to know what, what we should do, and how the, the decision-making process should happen, but other external factors or other subconscious factors, they, they, they influence it, so that it’s, it’s not really correct, so errors are made. Thank you.

HO I think both, uh, participant 18 and 19, when you all mention, uh, your definitions of cognitive errors, uh, both of you mentioned external factors. Would you, uh, like to just, um, expand on that? What do you mean by external factors?

P19 So, uh, this is 19. Uh, maybe in the case of the alcoholic example, the external factor would be your prior contact with him, uh, with the same patient, and every single time he was full of ETOH. So that’s an external factor. Uh, another example might be, um, the GP letter. So the GP letter, uh, is an external factor that leads you to, um, approach a case differently, yeah, than you would have lah without the letter.

HO Okay.

P18 Um, participant 18. So adding on to that, uh, what participant 19 said. Also other factors like time limits, um, interruptions, multiple, uh, patients, handling multiple patients at one time. Yeah, those also play a… They also, um, influence what we call cognitive errors.

00:44:15

HO Okay. So we talked about external factors. Are there… So besides external factors, then does that mean that… What are the internal factors? Are there internal factors that cause cognitive errors?

P17 Um, I’m participant 17. Um, I’m not sure whether… I mean, like personally, if I’m working for the past 20 days straight, [laughs] I probably have a lot of cognitive errors. So I’m not sure like whether fatigue is one of the things that, you know… Because that’s an internal factor. Yeah.

HO So how do you, how do you say, uh… How do you differentiate between what’s an internal and what’s an external factor to you?

P17 Uh, to me ah? Okay. Uh, so, um, not sure whether this is correct, but to me, like internal factors are…

HO I think it’s a very individual thing. Like whatever…

P17 Yeah. [Overtalking]

HO [Unclear] is, is, is what you feel.

P17 Okay. Yeah. So, um, internal factors, basically, to me, feels more like things to do with myself, like whether I’m tired, whether I need to use the washroom, whether I have, whether I have like…

00:45:24

There’s no biasness against this patient that I’m seeing. Whereas external factors is like things that you can’t really control, um, like, uh, whether or not like that day happened to be a very, very busy shift. Um, whether or not, uh, you know… What was the stuff that we talked about? Uh, yeah, whether or not like, you know, the patient is in a very bad mood, and therefore is like not telling me a lot of things, so I can’t, I can’t really have a good feeling of what this patient has. Yeah, things like that. Yeah. Things that are out of my control. Yeah.

HO Okay.

P19 So this is 19. Okay, to me, when I say external factors, right, I meant like, um, things that are not like factual. So, uh, whatever, um, 17 said lah. For, for… Okay, I don’t know whether internal, external is the correct term to use, but the opposite of external, what I was referring to is the facts on a case. So like, for example, the blood test shows this, then the, the, the clinical scoring shows this. That one, you cannot dispute.

But then everything else, whether it’s your, your own individual fatigue, or way of thinking, or previous experience, or systemic things like the environment, etc., those are all belong to external factors. So this is how I personally will define it.

P18 Hi. Participant 18 here. So, um, I’m also not really sure whether the term external is correct or not.

00:46:57

But what I meant was things coming from, um, outside, not, not within you, within you. So within you would be your own, um, burnout, fatigue, um, yeah, things like that. But other things like, you know, environment, busy shift, multiple patients, uh, interruptions, all that, I, I, I did consider as coming from not within me, so outside. So yeah. Biases can be your own bias. So it might be an internal factor, I think, if that’s the correct word. Yeah. So that’s basically what I meant.

HO Thanks. I, I, I think it’s, it’s, it’s such a rich discussion because everybody comes from a different, uh, perspective. And like even for this, this question, uh, the, the definition of an internal factor or an external factor is, is… It already… You know, even at this point, you, you, you do realise that everybody has a little bit… A slightly different view.

So I think that’s why we are so happy to have, uh, different people come in for the focus group discussion, and it provides us with a lot of, um, food for thought. And also, if you can imagine, uh, you know, in developing like future programmes or whatever for, for, for the junior doctors, how useful this will be. So thank you very much for contributing. Uh, I think co-facilitator is raising hand.

CO1 Sorry. Um, I’m just muted. Uh, yeah, sorry. So can I just check with you? Because, I mean, both of you all… I mean, all of you all have mentioned external, internal factors, right? So external factors, I mean, um, some of you all brought up that external factors is things in your environment, things that you…

00:48:50

But, but even things in the environment are within your control. For instance, if people give you distractions, you can say no to the distractions. So, so which do you think is more important? Would it be the internal factors, um, because, um, external factors can be controlled? Or would it be the external factors because internal, uh, factors, um, are not within, um… Are not something that you can change that easily? I mean, my question… My answer… My question is just, uh, which do you think is more important, internal or external factors?

P17 Uh, so I’m participant 17. Actually, I feel that both internal and external plays an equal amount of role lah. Right. Because, um, how you… Essentially, how we see the world is a reflection of what the world… Uh, it’s, it’s basically like what the world is in itself, and how we perceive it. So what the world is in itself is the external part, then how we perceive it is the internal part. So it kind of, it kind of weighs in on each other.

Um, so I don’t think it’s, uh, I don’t think it’s, uh… One is more important than the other. Sure, I mean, like if within your control, you can, you can eliminate things that cause you to have biasness in an external environment, that’s good lah, but sometimes these external factors are not even very evident. It may come… It, it becomes very difficult as well.

And of course, if you are aware of our own internal biasness, uh, I mean, then we can also work around it as well. So I don’t think it’s really like whether external is more important or whether internal is more important. I think they both play a role in negative outcomes lah, eventually. . Yeah.

00:50:39

P19 Hello. This is 19. Uh, if you are defining external as environmental and internal factors as within the person himself, then I would think that, actually, external factors might be easier to, uh… How to say? To control. Whereas… Because, because external factors, you can, uh, come up with workflows and processes to kind of mitigate the risk..

Whereas, internally, you cannot control how people think, right? So all you can do is educate. But when it comes down to the decision making, you cannot interfere with how the person actually think. So that one boils down to the individual lah. So in terms of importance, I feel that both are obviously very important, but then maybe it’s easier to address the external factors.

P18 Yeah. I’m participant 18. So I, I think, yes, both external and internal factors are maybe equally important. Um, so some, some external factors, um, probably we can come up with ways to control, but then sometimes they’re, they’re not very controllable by, by ourselves.

For example, um, the facilitator mentioned if, if somebody comes and interrupts you, you can say no, but then the interruption has already been done. Um, how do I… Say like, example, um, for now, we get, um, priority TigerText messages to, to triage. From the triage, for our ECGs.

00:52:25

So it can come at any time. Most often, if, if you are talking to a patient or talking to another junior or, or coming up with a management plan, you get the message. Yes, you can close it or say, I will reply later, but, but the message has already… The interruption to your thought process already has been done. Yes.

So we, we need to train ourselves to, to… How we, um, cope with it and deal with it, and, and how we, we manage these distractions. Where those things come with, um, time, we need time for that. We need our experience for that, etc. But, um, yeah, I think they, they are both… Both internal and external factors are important.

CO1 Thank you. Thank you.

HO Okay, so I, I, I think this discussion is great, because we, we, we somehow… Even though I haven’t been asking the questions formally, the, the answers have been developing almost according to plan, because you already mentioned… Uh, one of the questions was about why, why do you think, uh, EPs commit cognitive errors? And then we’ve already talked about… We’ve already touched on the internal and external factors.

And then, uh, I think the, the natural follow-on after, after that question, after we talk about internal, external factors, and, and then when, uh, the co-facilitator talk about whether we, we are able to control those factors, and how much, um… What kind of role do you think the EPs have in recognising and overcoming these, these, uh, factors? So, uh, the question is, is, do you feel that the A&E physician is responsible for recognising and overcoming, uh, the factors leading to cognitive errors? Or do you think much of it is a system-level error and is out of our control?

00:54:38

P17 Uh, so I’m participant 17. Um, I, I feel that, uh, if the system is designed such that it helps to highlight certain very dangerous things, uh, then it is actually very good for patient safety. Uh, either way, if you have to override it by yourself, then you have to go through this additional cognitive barrier, which will make you double check yourself lah, before you commit a grave error..

So a very clear example of this is drug allergies, right? Um, so you may not know that certain drug belongs to the same class, but because of the drug allergy alert, you may not have prescribed a drug that could lead your patient to having anaphylaxis. So I believe that with good system that’s built up, um, it can actually help to save a lot of patients from medical errors ah.

So a large part of us preventing errors is basically building up the system to support the juniors, as well as the other EPs on this. Uh, while it’s good to have some insight into your own deficiencies, but I think as an organisation level, um, I think system, systems are more important. Yeah.

P18 Hi. Participant 18. Um, so I think, yes, we are responsible, uh, to… When it comes to handling these cognitive, um, errors. Um, these… So one thing for ourselves, we, we, we have to build up, uh, how to, how to manage them, which methods to cope with them, overcome them, etc. And also when it comes to the system, we are also part of the system.

00:56:46

Um, and, yeah, so, um, there are like a few things which are like out of our, our control, like the patient workload, etc., but also we, we know that if this can happen, and how this can happen, etc. So I think we are also responsible to come up with ways, plans, um, that we can, uh, we can meet… We can, uh, intra-implement [?] to, to mitigate these errors. Yeah.

So examples would be, yes, the drug allergy alert, all the, the, the, um… How do you, how do you call it? The checklists that we have come up with over time. Um, the, the workbooks that we have with, uh, pathways, etc., which helps us to, um, know what, what the, the… What to do, uh, which shows us the, the, the, the clinical ways that I should… Could… Should be done. The, the… All the resources that’s available online, etc.

P19 This is 19. Uh, I feel that human behaviour is unpredictable, and that is just a fact of life we have to accept. And therefore, uh, I agree when 17 said that, you know, uh, any organisation or any system, we have to have some, some, uh, hard rules or hard stops in place lah to kind of, uh, eliminate as much as this human, uh, bias or error as we can.

And, um, and, and, and I think when you put systems in place, then actually you only… You not only protect, um, patients from EPs. You also protect patients from other humans, like nurses or other allied health, etc. So, um, yeah, I, I think, uh, the, the rules and everything are extremely important. Probably the most effective way to, to, uh, mitigate cognitive errors, because you can’t completely get rid of it.

00:58:48

HO Okay, so we are coming almost to the last half an hour. Uh, we’re just going to end off with some questions about, uh, education and, and advice to incoming new specialists. So if let’s say you have a new, uh, AC, uh, associate consultant, coming into your department, what… And let’s say you were supposed to give advice about cognitive errors, um, what kind of advice would you give the new ACs about cognitive errors that they should be aware of?

P17 Um, so I’m participant 17. I suppose I would be more concerned about premature closure, um, if I’m a new AC. Uh, the reason is because, uh, now, because you’re the overall supervisor of the floor. Uh, it’s… The onus is on you to scrutinise the case such that, uh, there is no… Nothing is being overlooked. Um, so that, that will be my number one advice. Just be aware of premature closure. Yeah.

HO Anyone else wants to give advice to the new incoming AC?

P19 Uh, well, as the AC, I will assume that he or she has much experience with all this, um, cognitive error. So actually I will ask… My approach will be to ask them, uh, what, what biases or cognitive biases they think they are most, um, vulnerable to, and what they have personally committed before, and then ask them to try and tell me why… What, what might’ve contributed to those errors, and then identify those, and then try and, uh, link it to our current, uh, ED environment lah. So what has been done to, uh, reduce the risk of those happening again?

01:01:10

And then therefore, they might… From our discussion, then they might be more conscious of, uh, what they themselves personally are more vulnerable to, and then they can own self go and take steps to, uh, prevent this from happening. Because like I said, I think system things, they are all there, but it’s the internal decision-making process that, uh, we cannot control. So that one is up to the individual to have to be aware lah.

P18 Participant 18 here. Yeah, so I think a new AC, the new, new to the… New… The new AC will still have gone through a lot of years of training and practice, etc. So they will also have their own ways of, uh, dealing with these cognitive errors. What I would, uh, advise them on is, um, the, the factors that, that… The system based, or, or the, the department… The things in the department that can, you know, uh, contribute to cause these kinds of, um, errors.

Um, so how do I say? Okay, so example. Uh, if we… Like where the patients are located, located or placed, so that, you know, if you need to review, you may need to make an extra effort. Sometimes they might be in two different levels or far away from you. So if you know that… Example, the alcoholic patient. So if you need to review, yes, you need to keep time for this. If not, you will miss it.

Um, and also if the procedure room does not have a nurse who checks in the patient ID and the name before bringing the patient in, yes, you have to do it. Uh, so, so things like that. So I will, I will share with them what I feel is the… That’s in the, in the system that can contribute to such errors, so that they can look out for them, and they can, they can, um, be aware of them and, and, uh, deal with, uh, those instances if they come up. Thank you.

01:03:24

HO So actually that brought up some interesting… To me, it’s an interesting point, because now we’re talking about, uh… If let’s say a new… So best advice that you will give to a new specialist, and you talk about how you will highlight the, the issues with the system, etc. So if, if let’s say you…

Do you, do you, do you guys feel that there is a difference in the kind of cognitive errors that, uh, let’s say junior specialist commit versus senior specialist? So we’re talking about like, uh, the new incoming ACs, or ACs as a group. Do you feel that the errors that… The cognitive errors that are being committed by the, the… Those junior EPs are different from, let’s say, if you’ve practiced more than like 15 years or 20 years. Do you feel that the type of cognitive error is different?

P17 Um, so I’m participant 17. Uh, so the question whether a more experienced specialist versus a less experienced specialist, whether the kind of cognitive errors is different. I, I think there will be difference, um, partly because of what, uh, experience has taught the more senior physicians. Um, they may err on the side of caution and may over-investigate. I mean, over-investigation by itself is also a form of harm. So if you think of it that way, then, yeah, there will be, there will be some differences ah. Um, so…

HO Sorry, can I clarify who is the, who, who is the, the, who is the over-investigating…

01:05:11

P17 Oh, okay. Uh, I mean, like for certain physician who because of, you know, of all the experience with bad outcomes, they may tend to over-investigate their patients more than the…

HO Ok so the ones, the ones who are more experienced will…

P17 Yeah.

HO Maybe over… Okay.

P17 Yeah. Yeah. Yeah. Compared to the more, um, junior specialist, who’s like don’t really have a lot of bad outcomes experiences, right? So they may not investigate as much.

The converse can be true sometimes, where because of someone’s, um, experience and comfort with ambiguity, they may feel that certain presentations may not be very high risk, and therefore they don’t investigate enough, resulting in bad outcomes. Versus the junior physician, who’s a bit more, uh, unable to deal with the uncertainty, and therefore over-investigates the patient and may find something that prevented a bad outcome.

HO Anybody else wants to add on? What’s the… Do you feel that there’s a difference between, um, more experienced or less experienced EPs?

P19 Hi. This is 19. I feel that this is very difficult to answer because, because, aiyah, I mean, seniority and experience alone, I think I cannot say for sure, because it will swing both ways.

01:06:40

You can have experienced clinicians who are risk-takers, yet you have seniors who are very risk-averse. So I, I, I, I don’t know. I don’t have an answer, actually. But definitely, the key difference is knowledge lah. So, I mean, if a junior doesn’t have the, the prerequisite knowledge, then they, they won’t know what they don’t know, and therefore then there will be more areas for every kind of cognitive bias. . Yeah.

HO Okay. That’s fine. I, I, I’m so happy to hear that, because like it’s not as if everything has a clear answer, and we are also not expecting you all to say that, you know, there, there’s always a definite for everything. So it’s, it’s good to discuss all this and explore together. Okay, so let’s move on. Um, the next question is, uh…

CO2 Uh, wait. Um, participant, uh, 18 wanted to say something, but yeah.

HO Oh.

P19 Yes. So, uh, participant 18 here. Um, for the last question that was asked, yeah. So I think it’s, it’s very, very difficult to say whether seniority has, uh… Will affect on how it, uh, affects these cognitive errors. Um, it, it really depends on that exact moment what happens and, and how things turned out.

01:08:05

Yes, uh, your experience and, and your knowledge will play a part on it, but, yeah, but there, as we discussed all this time, there are so many factors, I think, and, and the years of your practice that you have is just only one of them.

Yeah. But, um, maybe the external factors are maybe controlled, uh, to very, very seniors, where, you know, like, um, other, others around you may not interrupt you very often [laughs] compared to a very junior, uh, AC, where everybody around you will approach. . Um, but that’s just one, one, one factor alone. Yeah. So I think it’s very difficult to say, you know, this is how it’s going to be. It will depend on that exact moment and all the things that happened around it.

HO Participant 18, I need to clarify. Why, why do you say that some, some senior EPs will not get as many interruptions?

P19 Okay. So, uh…

HO [unclear]

P19 Sorry?

HO Why do you feel that?

P19 Oh, okay. So because, um, a young, a very new AC may be very new to that place of work or the system as well. Um, the, uh, the, the, the more senior ones may be more well, well known, uh, or, or the others may know how they, they function better, or they might, um… And, and the uncertainty may not be there with the very senior, uh, EPs versus the very junior ACs. Um, yeah, so it’s things like that.

01:09:59

HO Uh, okay. So, um, the next question is regarding education. Do you feel that education regarding cognitive errors is necessary? Yes or no. And then if so, when do you think this education should start?

P17 So I’m participant 17. Uh, I think that education is very important. It helps to build insight into what the underlying cognitive biasness that each of us possess. Uh, I think that training should begin, uh, I mean, very early on, preferably junior residency, so that I think the junior residents are a bit more aware of their own biasness, and therefore would be a bit better adjusted when they take on to more senior positions.

P19 So it’s 19. Yeah, I agree that, um, the earlier you are exposed to knowledge about cognitive bias, the better. And I also feel that the education about cognitive bias should be rooted in, in, in real life lah.

So not only like, like, oh, uh, what does anchoring bias mean? What does premature closure mean? But whenever there’s an opportunity, get the juniors involved in, um, like maybe RCA, if, if possible, or, or M&Ms, that kind of thing, so that they can, you know, they can link all this theory with what actually happens, and then, um, get discussions going on, so that they can go through their own thought process, their own real-life cases, and then that makes it a bit more relevant in meaning.

01:11:35

HO When do you think it should start?

P19 Uh, I mean, I, I think to have all this kind of training in place, you need to have the patients… I mean, the, the, the, the, the doctors in a kind of programme. So the earliest that will be, will be junior residency loh. I think MOship might be a bit hard lah, because there’s no structured programme, then… Yeah. But as early as you can, the better. So, yeah, at least JR or, or even earlier, if possible. Actually, when maybe med school. Um, wow, med school. Maybe med school years. I think med school might be, might be, um, a good time to introduce the concepts. Yes. Yeah.

P18 Yeah, participant 18 here. Um, so I think, yes, education is a must. Um, when you should start training, probably from med school. Though, um, as a, as a medical student, um, one may not be able to really appreciate it. I think the, the sooner we start drilling… Putting this, this, this, this concept into their minds, um, would be the better. So by the time they start, uh, patient interactions and start training or, or advancing in their careers, they can build up on it.

And I think the training should also be ongoing like, you know, like, um, at various stages, uh, of your training or your career, and, and also… Yeah. Yeah, that’s it. Thank you.

HO Okay. So, uh, we’re almost there. Um, the last question, formal question that we are going to ask is, personally, what strategies do you use to help mitigate and prevent cognitive errors? So your own personal, uh, your own personal, um… From your own personal strategies.

01:14:01

P17 Um, sorry, can you just repeat the question again?

HO What, what, what are your personal, uh, weapons against cognitive errors? What do you do to mitigate cognitive errors?

P17 Okay. I’m participant 17. Um, so I always, uh, I always… I guess, for me, uh, I just think that, um, there may be something I’m missing, so I’ll just keep double checking myself. Yeah. And if everybody keep telling me otherwise, then I also introspectively look at, um, whether or not there are aspects of the case they have not considered. So, yeah, that’s, that’s my thing. Just own self check own self. Yeah.

P19 Hello. I’m 19. Uh, I recognise that the times when I’m most prone to, to making cognitive errors will be when I’m very tired, never sleep. Or if I am very angry, like a patient family just yell at me. Or if the case is like very sick, and I feel like I’m pressured into making a decision sooner than, than I like to, like I don’t have enough time to think through everything. Um, or if, um, or if the case is just presenting in a very atypical way lah, you know.

So if I can… If I recognise that this is, this is happening, then I will try to, um, take conscious steps to, to stop myself, and then, uh, ask for other people’s opinion, other seniors’ opinions, and then, uh, maybe even call in patient, get more heads to think about it.

01:15:47

And then, of course, for my own personal thing. If I’m angry, then I don’t make decision now. I ask somebody else, or I just go and step outside and calm down. Or if I’m tired, the I make sure I sleep properly the night before, things like that. So, yeah, I think it’s recognition of what, what you are most vulnerable to, and then you try to, uh, mitigate that first.

P18 Yeah, participant 18. Um, so number one for me would be to, to recognise that these errors can happen, and also the, the times and, and, uh, the circumstances that they can happen. So I know, you know, this kind of things might… The interruptions will come. If I’m…

As participant 19 said, if you are very angry, or you had like a very challenging patient or a family with you, then you may not want to make like a very, um… You may not want to make a decision like just ten seconds after that. You may need time, like at least few seconds, to, uh, recover from that, uh, from the previous event.

Uh, and also have my, um, own ways of, of, uh, avoiding very, um… The common, um, common errors that, that can happen in… For, for us physically. Like, you know, what to do to, to check the drug allergies. What to do to make sure there’s no like, um, error in ordering the medication. How to, how to check, uh, check for the patient. Like having the conscious, um… Making a conscious effort to, you know, double check the patient’s IC, even if it was done by someone before. Um, yeah, things like that. So, uh, what else? Yeah, I think that’s, that’s it.

01:17:54

P19 Yeah, sorry, 19 again. Just to add. Don’t take shortcut. So respect what the system has already put in place. So if you got a checklist, RSI checklist, right? Must go through the whole RSI checklist. . And then, uh… Yeah loh. And if want to ask for drug allergies, go to SCM and check and check. Ask the patient. And if the family is there, ask the family. Yeah, so don’t take shortcut. Yeah. Thanks.

HO Okay. Thanks, everyone for your sharing.

01:18:24
