# Supplementary material for: Dropping the baton: Cognitive biases in emergency physicians
Source: PLoS One. 2025 Jan 2;20(1):e0316361. doi: 10.1371/journal.pone.0316361 (PMC11694980; doi:10.1371/journal.pone.0316361)
Supplement: S3 File — (ZIP) [file pone.0316361.s003.zip › Transcripts/FGD 6.docx]

FGD 6

Speaker Key:

MO Moderator

P21 Participant 21

P22 Participant 22

P20 Participant 20

00:00:00

MO1 All right, um, great. So, to start off, uh, maybe we can ask each of you, um, how long you have been an emergency physician. Uh, maybe just give a rough estimate. Less than five years? Like, or ten, more than ten years? More than 20 years?

P22 15-20 years.

P21 Uh, 21 here. About 20-30 years.

P20 Same as Participant 21, about 20-30 years.

MO1 Okay, thank you. What do you understand by cognitive errors?

P21 So, like, uh, okay, so like I say, uh, it’s, uh, emotion, uh, or some other influence, uh, factor that will not allow you, uh, to make a clear judgement, but you’re not conscious of it. Uh, and it can be either uh, uh, cultural, uh, emotional, or also be, uh, caused by, uh, uh, pressures of time, uh, or mental work.

MO Hmm. Thank you. What about, uh, Participant, um, 22 or 20?

P22 Participant 22. Also, I also have, uh, similar views to Participant 21. What I think also cognitive bias is a bit, uh, also a bit of overlap with, uh, fixation bias, lah. So they, they already have some preconceived, uh, ideas and, and what are the common practices that they, that they usually do or they believe in, you know? Uh, so it’s very similar to what Participant 21 has said, yeah.

MO Hmm.

00:01:11

P20 I think… Uh, Participant 20. I think cognitive, uh, errors or bias, uh, are because of, uh, uh… An error, lah, is probably a, a mistake or, uh, an outcome that happens after, uh, a faulty way of, uh, clinical reasoning has happened, uh, which may be influenced by various factors. Yeah.

MO Thank you, everyone. Yeah, so, um, um, I think… Yeah, so a bit of, uh, every… If you just put everything together, I guess, uh, it’s something like a cognitive shortcut that we use to help us along in our decision-making process. But, uh, sometimes all these shortcuts are heuristics, you know. Um, uh, they are faulty, and then they make us, uh, commit mistakes and errors.

And actually, it’s, uh, pretty common in, uh, clinical medicine. Uh, everybody, you know, regardless of, uh, how senior, uh, we are, uh, are vulnerable to them, lah. And it can be very difficult to recognise as well. So, um, we hope that we can work together with everybody to try to find out ways we can mitigate all these, uh, cognitive biases as, uh, emergency physicians.

So, um, the next question, right. Um, so what are the most common, um, cognitive errors that, that emergency physicians make? I think, uh, Participant 22 talked about fixation biases, right, but, um, you know, uh, in what context and, and what do you think are the most common factors? Are there any other errors that, uh, cognitive, uh, cognitive errors that emergency physicians make?

00:02:59

P21 Uh, Participant 21. So other kind of errors, uh, you, you mean outside of cognitive biases, huh?

MO As in, uh, what are the common, uh, cognitive errors or biases that, uh, emergency physicians are most vulnerable to, lah?

P21 Oh, I, I think most are, are… Okay, okay, I think that it’s usually, uh, uh, confirmation bias, okay? Uh, then I, I… Uh, then the other one, uh, is, uh… Uh, confirmation bias means you fixate, lah, on a, a, a certain, uh, uh, diagnosis, uh, and then…

MO Hmm.

P21 After that, you try to find evidence to, to, to confirm your suspicion. And I think in the ED with the time pressure, uh, that’s usually the most common that we go for.

MO Hmm.

P21 Thank you.

MO Thank you.

00:03:57

P22 So for Participant 22, I, I think one of the other things is not… I, I don’t really know what to say what bias is it, but I think it’s about the… Sometimes, it’s about the training, it’s about the system-based practices, lah. So, uh, like, uh, the different type of patients, your, your experiences will be different in different institutions, and you mould all these together. And, and that’s why, uh, a lot of the biases, I think, uh, overlap one, like physician confirmation, because you practice differently in practice certain areas.

MO Hmm.

P22 And I think also, it’s the way the… Our guys are, are, are getting information now these days, lah. They, they, you know, they are, they are getting not only from research papers, they are quoting, um, uh, like, level 4 evidence, type of thing, you know, like, like, for… From, from podcasts or, or, or, or, uh… I, I, I’m sorry, I’m not, not very good at this. But, yeah, so, so these are the few things that… Because a lot of these things are based on people’s experiences, so whatever other experts, or whether you’ve got expert speakers or what have… The, the… Whatever biases they have…

MO Hmm.

P22 Will be brought over to the people’s… To, to, to the clinicians also, lah, they are…

MO Hmm.

P22 Using this information. So, uh, these are a few of the things that, that, that I think is happening, uh, on the ground, lah. Yeah.

00:05:08

MO Hmm. Thank you.

P20 Participant 20 here. So I think, uh, I mean, uh, there are many, many different kinds of biases. I think very commonly, what I face when I feel… I mean, when I’m on the ground is, uh, uh, a framing bias, uh.

So when during a handover, um, somebody will show me something, they have done something, it is like this, you know, for example. Uh, they’ve already done a ECG, they’ve done troponins, they’ve done, uh, um, some other tests. And then now I’m waiting for a, a CT, uh, uh, PA. So, you know, please look out for a PE.

So after that, you know, okay, I’m looking for a PE. And it’s already been trained after all, all the work that has been done by my preceding consultant, you know. So, uh, then you know, it’s been framed, look out for a PE. So, uh, then I, you know, just look out for the PE, lah, you know?

Then after that, only to find out, hey, actually, the person didn’t… Already, you know, had the cognitive error, and then after that, transmitted that error to me. And then after that, you know, I continued with the error for a few… An hour or two until I realised and picked myself up, and then, you know, by which time, the, the acute coronary syndrome has had a delay.

MO Hmm.

00:06:36

P20 So framing bias is, uh, uh, one of those things. Because when the MO also comes and talks to me, then he frames it in a certain way to me, and then I end up continuing the frame. And then that may be a possibility. So I think that is very common, uh, framing bias.

Uh, another one is systems, uh… I think it’s related to the system, lah. Like, for example, I’ve had many, you know, um, uh, incidents when, you know, when I did M&M, and I, I… You know, and, uh, the, the error was, why was the patient, uh, admitted to a general ward, and then sent to, uh, ICU within, how many, two hours or one hour.

MO Hmm.

P20 And then it’s because, uh, oh, this patient got pneumonia. And then the person, uh, already knew that the CURB score was high, but yet, because of the system, no bed in ICU, don’t want to talk to the, the medical registrar on call, very difficult, um, communications and also bed situation, then for… So it’s a system problem that makes him, even though he knows the diagnosis, this is, uh, pneumonia with a high CURB score, still decides to send to a general ward because of all the, the other thoughts that come in to influence him.

MO Hmm.

00:07:53

P20 So, yeah, there are many, many kinds of, uh, uh, errors, uh, that happened, uh, both, I think, by… From the way that we think, and also by the way that the system makes us, um, uh… Leads us to, um, make certain decisions in a certain way. Thank you.

MO Thank you. So I think, everybody, we talked about different, um, aspects, right? Uh, Participant 21 was sharing about time pressures. Participant 22 was talking about how we get information from various sources. And then, uh, Participant 20 was talking about the knowledge, um, even though knowledge is good, but then, uh, there are systems, uh, practices and all this.

So now, I mean, because of actually, I mean, uh, all of you as, um, HODs and ex-HODs surely have encountered, uh, various examples of errors, medical errors in general, lah, medical errors that are committed by, um, specialists, you know. So, so to what extent do you think these were due to cognitive biases? So is it a large proportion due to cognitive biases, or is it due to other things? Like, is it a IT system problem, or is it a knowledge gap, or due to, uh, you know, uh, uh, the, the, the environmental factors or what? Yeah?

P21 Uh, Participant 21. Uh, uh, we don’t… Uh, okay, I don’t have actual numbers or figures, uh, ‘cause we never, uh, exactly, uh, uh, uh, uh, study. But from the cases that I’ve been involved in, uh, COI and also M&M, lah, a lot of time, uh, uh, it is a, a, a combination. Uh, you know, these are, uh, these are, uh, vulnerable times, for example, where the hospital, uh, uh, system, uh, system, uh… The hospital is short of beds, uh, or when people are about to go off, and then some other are coming on, and you hand over, but you don’t do it properly.

00:09:55

Or sometimes, I also see a IT system, uh, letting us down, lah.

MO Hmm.

P21 For example, uh, uh, the IT system, uh, uh, when you have one printer, okay, uh, shared between, let’s say, three or four, uh, uh, uh, desktops, you know? And then, uh, when you print out and you grab different, uh, uh, people’s, uh, uh, papers, uh, come out together. So these are the type of system error, lah. Okay?

Uh, human error, I find it becoming less and less because a lot of the time, we start to work in teams. And juniors…

MO Hmm.

P21 Are, are also, uh, encouraged. In, in fact, uh, some… Like this, we’ve mandate, lah, the new juniors, uh, must vet every case with a senior, okay?

MO Hmm.

P21 So, uh, if the senior, uh, uh, takes away the framing effect, like, like Participant 20 said.

MO Hmm.

00:10:47

P21 Okay? Then usually, uh, uh, you, you’d be quite okay, lah. So I find human error, lah, individual human error becoming less and less. Uh, and then finally, yeah, there’s those atypical presentation that no one can, uh, do anything about it.

MO Hmm.

P21 And when we as a department, uh, uh, do… And we looked at it during M&M, uh. And we said, this is really atypical. We just cannot make that kind of diagnosis in the A&E.

MO Mm-hmm.

P21 And, uh, so this, I think… The last one is the least likely, lah. So more and more, uh, I, I feel, lah, it’s usually the system. It’s a very complex system we are working now, you know, with big shortage every day, uh. Uh, uh, uh, patients are… Not being able to track patients, not knowing where your patients are anymore.

MO Hmm.

P21 So that’s my ranking, lah. Number one, uh, system. Uh, number two, uh, uh, uh, uh, human error. And then number three which is like very atypical, uh, very rare, very atypical kinds of cases. Thank you.

MO Thank you.

00:11:47

P22 Uh, Participant 22. So, uh, I, I also, uh, have, uh… Some of my thoughts are similar to, to 21. Uh, so, uh, uh, but I think it’s difficult to rank, lah. So I think, to me I was speaking to, you know, it’s, it’s the physician error as… Uh, physician or staff error, um, you know, the system error, as well as the patient is… Patient themselves, lah.

MO Hmm.

P22 So I say, because sometimes the patients very… Give very, very, uh, different history at each different point, or they present in a different time, or, or, or they don’t share the relevant information which will…

MO Hmm.

P22 Lead us to go down a different track, lah. Okay, that, that one is, you know, something that you have… I think I, I acknowledge that there are these things. Uh, I think the systems are, are, are really coming, uh, under a lot of siege, uh, you know. Uh, so I think you had to build in things to, to mitigate some of the errors that comes on, uh, because that is the… That is the thing that we have to… We have to live with. Uh, the system will never be perfect, right?

The other one is the physician. So I, I think the physician one, what I, what I personally have seen, I unfortunately don’t think it’s, it’s decreasing. I don’t think it’s really increasing. I think it’s always there.

MO Hmm.

P22 Uh, and it’s… You can see the… Uh, and I think it varies according… It’s, it’s a very big spectrum of, uh, of the seniority of the physician. So, for example, like, what I tend to notice is that the, the younger the physicians are, uh, the way they ask history-taking, all these type of things, are very, very different from how a senior physician asks.

00:13:14

MO Hmm.

P22 So, for example, the junior physicians, lah, they will only ask what they think they know. If you, you understand what I’m trying to say. That means that, for example, if they say a back pain is a back… Like a MSK pain, they don’t think of it, their, their, their questions will be geared towards a certain, certain type of history-taking to get what history they want.

You know, uh, uh, but a more experienced, uh, physician, then, the experienced physician actually comes two, two, two things, lah. One is they, they are too over-confident, then they, they, they do what they want to do. Like, you know, they, they, they ask questions according to what they believe. Like, what you all thought about framing bias or these other thing. But the other one, the, the, the more… The, the more experienced some of them are, they actually can… They ask more… They ask… They have a broader differential diagnosis to us, lah.

MO Hmm.

00:13:57

P22 So in other words, you will only know… You will only… You, you, you, you also can… You also will make errors based on what is your… What type of information you have at that point of time.

MO Hmm.

P22 Or, or whatever, whatever cognitive law you have in your mind at that point of time, lah, right? So, so that is one of the, um, uh, the things, lah.

And I think the… As… I think, like, what I also mentioned is that, you know, that we do see some of the… Some more experienced physicians, uh, that, um, they, when they become too over-confident, lah, then they tend to take shortcuts or they… Like, like, they, they, they have the… They, they, they already come out with a preconceived idea what the patient…

MO Hmm.

P22 Is when they see certain type of patterns. But we all know that… We all know that people are complex, lah, in the sense, lah. So, so that’s, that’s my… That’s my take, lah. Yeah.

MO Thank you.

P20 Hi, can… Uh, Participant 20. Can I… Can you ask you question again? ‘Cause, uh, yeah.

MO Uh, so, uh, because, uh, all of us actually encountered, you know, examples of medical errors that are committed by either, uh, emergency physicians or by ourselves, um, as specialists. So to what extent do we think that, you know, these, uh, medical errors are due to cognitive biases or whether they are due to other things.

00:15:13

P20 Okay, uh, Participant 20. So I think that, um, uh, a lot of times, we have, uh… Nowadays, we have put in systems to help us, uh, mitigate cognitive errors. So, like, uh, when we intubate a patient, you know, we have a RSI checklist. Or when, you know, we have, uh, some checklists, lah, uh, and, uh, some systems that we put in, like a observation ward, um, so that it gives time for certain, um, diseases to manifest itself over time.

MO Hmm.

P20 So, uh, yeah. So systems have been put in because we recognise that the humans, uh, I mean, do make errors and maybe cognitive or other kinds of errors, lah. So I think a, a good proportion, um, of, uh, cognitive errors by specialists, not, not, not by, um, junior doctors and all, lah. Uh, for junior doctors, I think most of them are maybe… Many of them may be cognitive, but also due to lack of medical knowledge. Uh, so, so they have a, a high proportion of mixture of medical knowledge and cognitive errors.

But for the, um, uh, specialists, I think a lot of them are less medical knowledge errors, not that there aren’t any. Because, um, medical science has improved, and in the times when we were learning last time, uh, we didn’t know anything about… Very much about transplant problems, diseases…

00:16:46

MO Hmm.

P20 Caused by transplants and drugs and other complicated, uh, conditions. So even then, we will have medical knowledge errors.

MO Hmm.

P20 But, um, I think that, uh, in general, um, for the run-of-the-mill cases, it would more likely be cognitive errors, um, versus medical knowledge errors, if it is due to, uh, uh, the common, uh, typical presentation, uh, kind of, uh, uh, error.

MO Mm-hmm.

P20 Yeah. So I, I, I think that for the specialists, uh, that would be, uh, yeah, more, more of the cognitive errors. And a lot of the system errors, I think, uh, we have been trying to mitigate with, uh, uh, putting in place, uh, some, some checklists and, and, uh, measures to minimise these as much as we can. I’m sure that every department will continue to do that, lah, once they figure out how to do it.

MO Thank you. So I think we, we mentioned a bit about, you know, uh… All the participants so far talked about, you know, systems, um, how systems and the individual have a role to play, right? Because, uh, on one hand, we have systems, uh, environmental factors that, uh, predispose us to making errors, and on the other hand, we have, you know, uh, human, uh, factors as well.

00:18:14

So, so, in your opinion, what do you think, to what extent do you feel that, um, this is… Actually, all these cognitive errors, cognitive biases, are the responsibility of the individual emergency physician? Is it true the responsibility lie with the emergency physician to recognise that he’s making a, you know, cognitive bias, and then try to overcome it?

Or is it a systemic, uh, failure that should actually, um… You know, is beyond the control of the individual? It’s human to make errors, and therefore, anybody, uh, um, is likely to make a same error, given, um…. If put in that environment. So is it a, a individual problem, or is it a systemic, uh, team failure, environmental failure, that, that is responsible?

P22 Uh, Participant 22. So I think to just answer that, that, uh, I think we… I think the system or the healthcare system, uh, in ways, uh, recognises that everybody will definitely make mistakes, and, uh, nobody is perfect, lah. And to state before I go… Before we go forward, I think also we have to recognise that we have our own biases, lah. That’s why even when we see patients or even when we investigate cases, uh, you know, or, or deal with people, we have to be mindful of that.

00:19:36

So I think why… Uh, the way the system combats this by having all these, TeamSpeak, you know, SBAR, uh, uh, you know, and Just culture and all these type of things is because we want to encourage a team system where, where even the juniors are comfortable to, to inform the senior that something is wrong or, you know, or they don’t think it’s wrong, and then have a discussion at that point, lah.

So then it again depends on the individual physicians. When this system… When this system is done properly, whether or not, um, did they heed the advice, did they, did they slow down and take a checkpoint, take a, take a break or take a checkpoint at that point of time to make sure that what they think, what they think is correct, lah. So I think that is the system that… That is the thing that it’s done.

So if, so if, let’s say, if, uh, if, if, uh, this thing happened and the mistakes due, uh, mistakes due is done by the, by the individual physician, then the, the, the error will lie with the physician lah, because the system is clean, but the person…

MO Hmm.

P22 Chose to go ahead despite what has been taught to him. However, if we don’t… However, uh, this is, this is not practiced in a system in the… In the partner or in the system, then we have to look at it as a… Both, lah, that means as, uh, as the individual as well as the system itself to why was this… Why don’t we have this culture to, to, you know, to a safe environment for people to speak up to say there’s an error, lah. You know what I mean? Yeah.

MO Hmm. Thank you. Uh, Participant 21, what do you think of, um, what Participant 22 said?

P21 Okay. So in, in, in… Uh, how do I put it? Okay, so that brings a interesting point, uh, whether you comply with guidelines or not. Um, how should I put it? Okay, uh, so, uh, okay, in operating data, right? Operating…

00:21:30

MO Mm-hmm.

P21 Data, you… Everyone’s supposed to go through a checklist here.

MO Mm-hmm.

P21 Right site, right patient, uh, whose role is doing what. Okay. But in the ED, okay, uh, okay, let’s say, uh, uh, you are supposed to have a time out before you insert the chest tube, make sure you don’t go into the wrong side.

MO Hmm.

P21 And that’s also a good habit to have, lah. But let’s say, uh, like, uh, uh, something, uh, therefore, which there is no, uh, uh, uh, uh, guideline or protocol, okay? Uh, say, uh, uh, uh, you, you have a head injury, uh, you, you got to do C-spine clearance, you got a NEXUS criteria, or you got the…

MO Hmm.

00:22:08

P21 Canadian CT head rule. Uh, those are helpful, but of course not infallible, but they could help you, uh, uh, in terms of, uh, maybe giving you medicolegal protection versus saying, ah, I had good grounds to believe this scan is needed, etc.

MO Hmm.

P21 But, you know, that, uh, takes about 40%, 50%, lah. Then the rest are, all those things are ill-defined conditions. Uh, then clinical judgement, uh, will come to the fore, and that is… That’s where, uh, people always in retrospect, uh, if things go wrong. Because, hey, we are so obviously, uh, uh, uh XYZ diagnoses. How come you didn’t make it at the time?

Uh, so, so I think whenever we analyse these, uh, we must say, I must stop thinking, uh, from the retrospective view, but put myself in the shoes of their doctor or nurse, lah, even, lah, and say, why did they think that way in the first place? Then it makes, uh, uh, sense, lah, to, to, to, uh, to you, uh, why certain mistakes happen.

It really, uh, is, uh, uh, kind of a grey zone when it comes to conditions, uh, that do not fulfil any kind of guidelines or rules, uh, uh. Uh, syncope rules are helpful, uh, indications for scan of heads. But, uh, I really have no answer on those cases where there’s no good guidelines. Thank you.

00:23:35

MO Thank you.

P20 Uh, Participant 20. Uh, the question is about, uh, who’s responsible, right?

MO Yeah.

P20 For cognitive errors? So, um, I think that, uh, uh, most of the time, the specialists, uh, treating a patient, uh, physician, uh, has the responsibility, um, to be, um, careful and take responsibility for his decisions, lah.

So for example, um, when you have a patient who comes in and, uh, you know, he’s alcoholic, he’s disruptive, you are angry with him, you know, and have all the emotions, uh, because he, he’s, you know, making you, uh, emotional and all because he, you know… I mean, he, he… You just feel, like, assaulted, uh, emotionally and psychologically by him, and you want to, um, either hit him back or you, you want to do minimum for him.

But actually, this… You know, and he may have cried wolf many times by coming to the department, uh, saying he’s got chest pain, chest pain, chest pain. And every time you have been… He has been admitted many times, and discharged many times, and had all kinds of tests. And you’re sure that he’s wasting your time and resources and so forth.

00:24:57

And then you ignore him, and then you do the minimum for him. And you may one day find that he will wake… That he wakes up, you know… He doesn’t wake up from your observation ward, you know, because he had a bad outcome. You know, then who is responsible?

So I think, um, you know, you… Uh, so as a physician, I try to be mindful, especially these people who, who, uh, evoke certain kinds of emotions, um, and, uh, you know, I, I would… You know, um, uh, although I, I’m mindful that he may be wasting resources, but he also may have a genuine problem. So therefore, I try to, uh, avoid it. So I think I have that responsibility, and…

But unfortunately, you know, um, I think, uh, I think sometimes, because, for various reasons, uh, we may not, uh… Uh, we may minimise his symptoms after he’s visited us for the tenth time. So some people, you know, um, may ignore this patient and leave him alone, and, and therefore, you know, that’s where the bad outcome happens, lah.

Uh, and, um, so… And there are many reasons. I think that, uh, as physicians, we have a responsibility because… As specialists especially, because we are trained and we’ve had a lot of experience.

Um, but, uh, of course, if it’s junior, then it is for us to, um… When they come to us and ask us, then we have to also, you know, do our due diligence and be mindful and go and, um, uh, you know, check up on the patient as well, so as, you know, he may… Uh, just so as to mitigate his cognitive error or his medical knowledge deficit. So there… So it is a bit, uh, I would say a bit stressful in the sense that, uh, you have to not only, uh, control his errors, but you also don’t know whether he’s got enough medical knowledge to, um, make the correct diagnosis.

00:26:54

MO Hmm.

P20 So, um, yeah. So I think, uh, yeah, of course, the system has a part to play, um, because all of us are now, um, aware of the situations that happen in our department, and therefore we as human beings put in all the checklists and systems that… To mitigate all, all the potential errors that we know how to mitigate, lah.

So even, even the system doesn’t come from itself, but it comes from people. So it’s the people who realise, hey, these are potentially errors, whether cognitive or otherwise, um, and therefore, we ended up putting up all these systems to, uh, overcome it. But in the end, when it comes to one-on-one, um, when we see our own patients and we are responsible for the patients in our, you know, um, in our care on the shift, then we are generally the, the ones who I, who I feel, lah, are responsible.

Um, and of course, I also realise that a lot of things may happen, lah, like, um, distractions and stuff like that. So… But, um, overall, uh, as seniors, we hopefully have, uh, uh, developed within ourselves strategies to, to, um, overcome all these that we know are, um, stumbling blocks when we work and, uh, and, and, um, you know, and therefore, we hopefully have some strategy to come back to the situation. I, I think some, some people may have some strategies, but anyway, yeah, that’s, that’s all I have to say for the time.

00:28:35

MO Thank you. Uh, Participant 22, um, what do you think of what Participant 20 said?

P22 Uh, I mean, uh, what she said has, has a lot of good points. Uh, but I, I, I think one of things that I think the error part is about the coping strategies, lah. So unfortunately, we cannot… Uh, I, I feel no matter how senior it is, right, I mean, based on what I… Based on the M&Ms and all the, the things we had to go… We had to do, we had to… We had to investigate, right, that it is unfortunately, to me, quite clear that, uh, coping strategies are also people-dependent.

Meaning that a senior person may not have very good coping strategies compared to a more junior consultant because of the life experiences the person may have, and how, uh, how willing they are to accept, um, uh, uh, uh, tactile feedback or, or, or, or how they, how they react to… It’s situation awareness, lah. So…

MO Mm-hmm.

P22 You know, uh, uh, I, I personally have seen before, some, um, some, uh, people will say that, you know, uh, the, the, the, the, the reason which I also agree and I recognise as similar to Participant 20 and 21 that we are busy and, and, uh, there.

However, the… We, we, we… I think one of the… One of the things that… Why, I think, the errors may occur is because we use this as… We, are distractions. So we… I think what is… One of the things that we had to recognise, there is some situational issues or situational challenges that can affect we, we want to rush through to go through many cases at one time. So sometimes we may have to realise that it is important that even… That’s why the coping strategies are such, lah.

00:30:20

So, uh, I don’t think it’s part of your… Of this discussion per se, but coping strategies, we have to tell people that we are only human, and even how senior we are, we have to … have the insight to recognise that we, we… We might not be able to cope with certain situations, and we had to… That’s why the… That’s, that is the reason why, uh, the system is built with all these things involved, so that at least in times of very high stress, right, this system is supposed to kick in.

Or even in times when, when it’s good lah, that means that your, your load is not high. That is a good time to practice all these team systems, uh, that you’re supposed to. Like, like, you know, in Resus, we have a multidisciplinary team… We have a… We have a team system where the reg, the SR and the… And, and a senior can talk to each other.

MO Hmm.

P22 And they are supposed to feedback to each other that, that there’s something wrong, lah. I think… I, I think that that may be… I think part of it to also prevent errors is about having this… Like I say, you know, this culture to, to do this. And recognise that a senior actually cannot assume that a senior has coping strategies. Uh, uh, the more, I think…

00:31:19

MO Hmm.

P22 Uh, that is my, my rule of thumb, lah, my personal rule of thumb, yeah. You just had to recognise that everybody has different, has different experiences and different, different way of, uh, reacting to situations, uh, yeah.

MO Thank you. Actually, this leads on to my next, uh, question, also, very nicely. So, so the next question is actually, um, do you think there’s any relationship between cognitive biases and seniority? Like, do more senior, um, uh, specialists make different cognitive errors compared to junior, uh, specialists? Uh, anybody?

P21 Uh, Participant 21, uh. Uh, actually, the more senior you are, it can go two ways, uh. I’ve seen something that colleagues have become over-cautious in my opinion…

MO Hmm.

P21 Because there have been, uh, in many, uh, uh, uh, uh… They have seen many atypical presentations…

MO Hmm.

P21 As a result, lah. Uh, after that, they’ll just keep thinking, I must exclude this atypical presentation again, because uh, I have made the same error, lah. Uh, for example, uh, you, you’ve got this renal colic… and then one day, it turned out to be a triple A. And then from there on, they are always saying, uh, ultrasound, uh, the aorta before you’re send home any renal colic. Uh, then on the other hand, uh, there are seniors who say, uh, yeah, I did this so many times, the number of, uh, triple As are so low.

00:32:53

MO Hmm.

P21 Uh, and you, you don’t, uh, need to do it, uh, unless, uh, the person is, like, you know, the person has atherosclerosis or is over the age of, uh, uh, 50, for instance, uh. So, uh, it, it could go both ways. Uh, one become overly cautious, one become over-confident, uh, and, and, and, uh, I, I think the most important thing, uh, is to have, uh, healthy discussion, uh. Uh, then, uh, depends, uh, on how, uh, how much risk. Because A&E, I think, is all about risk management.

MO Hmm.

P21 How much risk, uh, the senior is willing to take, lah. Uh, I, I just, uh, feel, lah, that, uh, if the juniors check with the seniors and the seniors say, okay, let’s go for option A, and then the juniors say, uh, you know, but I feel there is some evidence, uh, that it could be option B diagnosis.

MO Hmm.

00:33:53

P21 And then the senior will say, okay, uh, and brush it off. I think I know more than you. Uh, that’s dangerous. Right, the senior says, okay, I think you could, could be right. Uh, convince me! And if you could make a good, uh, uh, uh, argument, we’ll follow. And, and I’ve… Honestly, many a time, uh, a junior has saved my life. You know, I, I don’t think it is your diagnosis, but you are quite sure, and there are atypical presentations which, in retrospect, uh, after I listened a bit more carefully, is, okay, I accept that. Let’s work it out. And then, hey, it’s true. They are correct!

So, uh, uh, uh, so like I say, lah, uh, you… It’s hard to have a middle ground between over-confidence and, uh, over-cautious. Uh, you got to manage risk. But I say, let’s listen to your colleagues or junior. Uh, they may have reasons to have the clinical gestalt, just tell, tell them differently.

MO Hmm.

P21 So if we, uh, do that, uh, uh, uh, uh, risk… Uh, I, I think less mistakes will occur, lah. Uh, uh, uh, uh, some stuff, I know, lah, for sure, they will always over-investigate, and then the juniors are like, I don’t really think so. But then the juniors are settled with the unpleasant task of having to order, you know, excessive…

MO Mm-hmm.

P21 Uh, scans and all that. And in the end, uh, turn out to be negative. You know, a, a 20-year-old head injury, but no LOC, nothing. So I, I, I, uh, I think there’s no easy answer. I think it can go both ways, uh. But just, uh, have a discussion with your junior. Or perhaps even, uh, I myself also ask another senior, what do you think?

MO Hmm.

00:35:39

P21 Uh, and I know some of them are experts in areas, and not… Okay? Uh, uh, and if you could, uh, listen to other people, uh, that would help a lot, lah. Uh, thank you.

MO Thank you. Thank you, Participant 21.

P21 Sorry, what? Sorry, what?

MO Uh, no, uh, thank you, Participant 21. Yeah.

P21 Oh.

MO Um, what about, um… Yeah?

P22 Can I just ask, uh, what’s your definition of senior, lah? SR is also senior?

MO Uh, this… I mean, when you’re talking about specialist. So senior as in a more…

P22 Oh.

MO Senior consultant, that kind. Yeah.

P22 Okay. So, uh, I think, uh, uh, based on what I see, the, the, the highest rate of, uh, errors, uh, that I… That we tend to see are among the juniors, and especially the… I feel, lah, that the most… The, the, the most dangerous period is when they first turn as a associate consultant.

MO Hmm.

00:36:42

P22 That is what I have, uh, noticed, as well as, uh, the, the one that… The junior consultant, that means first-year consultants, lah. I, I, I’m not very sure why is that so, but I think sometimes it’s, you know, when they first turn into a consultant, they, they, they feel more confident because they just passed their exits, you know, this type of thing.

MO Hmm.

P22 So, so we do see a, a, a, a… Not saying a significantly higher, but we do see a bit more, uh, high, uh, uh, uh, high-impact errors, uh, during this period of… during this period of time, lah. You know? And I think why… I think, like, what Participant 21 says, right, is that after they get hit by these, ah, they, they… All these, you know, they, they get safer as they grow more senior. Because, you know what I mean, they, they, they learn, uh, as it goes along, lah.

So, so, so that’s how I see. I, I… My own personal experience is, uh, in I, I think the more senior they are, they tend to… They tend to be a bit more… They, they tend to be a bit more, uh, conservative, lah. You know? And I think it’s because of our… Again, it’s because of life experiences. And, and, and that’s why, uh, uh, the, the, the… That’s, that’s, that’s why I think the lesser… We, we see lesser high-impact, uh, errors in the… In, in, in… When they are more senior.

They may make errors also. I’m not saying they don’t. But because I think they are more… They are more, uh, uh, uh… They have been beaten before. You know, they have experiences before, or they hear stories before, so they, they tend to be a bit safer in that sense. But they may still make, uh, errors, but the errors are not as, um…

00:38:25

MO Hmm.

P22 Very high-impact, or that can actually cause issues, lah. You know? Or that can cause very big repercussions, uh, to, to patients. That is what I… My, my personal experiences are. So, so that’s why usually the, the, the… They are a bit more, uh, risk-takers. They are a bit worse risk-takers, uh, when they are younger.

MO Hmm.

P22 But, uh, I think that what, uh, 21 has mentioned, right, is that, that’s a problem. So, so I think the, the… We, we had to balance to say that, um, that if a error is made, a cognitive error is made, for example, in any, any grade of physicians, then we will also have to see what are the issues, and, and rectify the situation. ‘Cause we also don’t want to create a system where, just because of a cognitive error out of one… Out of 5% or 1% of, of the patients that we see, then we start… Then we start implementing things in the system to make it more complex to get more errors.

00:39:25

MO Hmm.

P22 Because when the systems get more… Uh, like what 21 had mentioned, lah, when you make something that is meant to be very simple, uh, more and more complex, you’re actually inadvertently, uh, introducing more errors in the back in the…

MO Mm-hmm.

P22 Uh, that is one thing that we have to balance, lah, based on that, lah. So, so I think it’s about, it’s about, uh, being… It’s, it’s about balancing these two so that the… You don’t swing into a, a, a, a very safe… Extremely safe behaviour, and, and, and also don’t be a risk-taker. Yeah.

MO Thank you.

P20 Uh, Participant 20. I agree with, uh, what Participants, uh, 21 and 22 have said.

Um, yeah, I feel that, uh, when, in my term as a previous HOD, most of the serious impactful, uh, errors, uh, happened during junior AC, AC and junior consultant period. Yes, probably as rightly said by Participant 2, that they have, um, been given the licence and, uh, they have independence, and they have full confidence after passing exam. Um, not realising that actually, there’s so much more in the future to learn, uh, and that, um, they have become masters of, uh, typical presentations, but they have not become masters of atypical presentations.

And, and, uh, probably none of us would be masters of atypical presentations because, uh, I feel that, uh, I think, um, the whole of the… Perhaps the whole of the system has, since we were in medical school, uh, is a, is a, is a framing… We have been framed to, um, make everything, uh, very simple for the medical students. So chest pain, angina looks like this. Chest pain radiation diaphoresis… looks like this. But if a patient, um, and if the person encounters somebody with, uh, feeling lethargic, uh, some pain not in the chest, uh, maybe, you know, uh… And, um, some, you know, vague symptoms, then he doesn’t recognise it.

00:41:40

So it is of… I, I feel that, uh, perhaps, uh, cognitive biases have always been there, uh, not just medical knowledge. Medical knowledge is something that we learnt when we were in medical school, but we were no… And we were always the simplified, uh, straightforward, very typical things. So it has always been framed as typical. I mean, framed as is, like, chest angina, ACS, there’s a particular diagnosis. But we never learned the atypical parts of it much.

And so the emphasis over the years have been a… This very typical diagnosis. And therefore, when, when the atypical diagnosis or some atypical features come in, we don’t look at it, um, and we maybe miss it. Um, and then, uh, we, we call it something else like musculoskeletal chest pain or something else, uh, like, you know.

So, um, therefore, since, uh, medical school… Uh, so I feel that, uh, medical school, uh, sort of sets us up in a way, uh, to, um, to cognitive biases because it teaches us very simple ways. Because otherwise, we cannot get through medical school, which is only five years. And if we have too many variables in our learning, we end up, uh, having, um… We end up, uh, not being able to finish medical school for life. And medical school is actually for life, after we realise it as, you know, uh, as we exit and continue to learn. So…

00:43:10

But, to get through medical school, we learn all the typical presentations. And typical presentations end up being framing. We are framed to follow these things. So that’s why atypical things like posterior circulation stroke always gets missed or always delayed, because giddiness always gets replaced by some other diagnosis, and we learn by system-based… Uh, symptom-based approaches. So I think, uh, we have been framed.

And then, lah, I remember when I was a medical student, uh, you know, I was following a very famous physician. And then the famous physician… You know, I was a… I was a, like, third-year or something, doing surgery. And I was in the clinic. And then I was asked to take a history.

And I started to take a history in a very systematic way, only being cut off by the surgeon who said, don’t ask all these questions. Don’t… You know, just ask the typical questions. Do you have, uh, RHC pain? Is it worse when eating? You know, does it relate to the fact, you know, that you have a biliary colic there? So I learnt… So I learnt from my teachers and I learnt from role modelling of, uh, all my other teachers to get to the diagnosis, and then, and then, uh, you know, and that, sort of, made me do all this.

So as I was learning and being formative, and I don’t know what’s happening, uh, I’m just a blur medical student, I ended up, uh, you know, following the role models who were very strong in my life, uh, who, who made an impact, you know. So, uh, so that was one of them. So I think that’s how I… You know, I become very… Learn to become targeted in my history. And some other role models as well.

00:44:46

Then when I learned to… When some other discipline asked me to go and give how many differentials, you know, then I, I struggled. But nobody told me that’s a good, good thing that, you know, you need to have more differentials. So the one that gave me good feedback is the one that, when I showed him that I was very targeted, and he got the diagnosis. But the one that I was struggling with didn’t give me good feedback when I came with many differential diagnoses that didn’t make much, uh… I mean, which, at the point in time, and I was so junior, I couldn’t make much sense.

And then when I… You know, and then I, I think, uh, also, um, uh, now being… I mean, being a, a, a, a, an assessor and teacher, we make, uh, we make the lessons very simple and very targeted, and there must only be one diagnosis. And then we think… Tell people, students, common things occur commonly, so therefore, they don’t think of the uncommon things. So, uh, so we also end up framing for our juniors whom we teach.

00:45:47

And then in our exams, there’s also one diagnosis. We don’t play out… Because I make it very straightforward. We set the questions, uh, whether it’s medical school, it is very fixed. There’s one diagnosis, so they… You know, even though we ask them for some differentials, but actually, our patients in real life are more complex than having just one diagnosis. So we, we are one diagnosis, which is maybe heart failure, but we don’t forget that the heart failure patient also may have pneumonia, because we don’t test things so complicated, even in medical school, or maybe even in our exit exam.

So all along, I feel we have been trained, um, in a way that makes us, you know, um, be more efficient, and maybe in the simple way because it’s very hard to teach complicated things. And we hope that the complicated things, they will pick up along the way in their experience. So that is… So that is, uh… So I think… Yeah, so the… Sorry, uh, what was your question again?

MO Okay. The question was, uh, what’s the relationship between cognitive biases and seniority? Like do senior…

P20 Yeah.

MO Make different errors?

P20 Yeah. So, so, so I think as senior as we go, uh, we will also carry some of these biases with us because we have… We have been… So that’s why I was reading this, uh, new article on, uh, OMI, occlusive myocardial infarction. I think some of you know about it. And then why we keep missing all these OMIs.

So that’s, yeah, as, as, as… You know, um, the more senior we are, we, we may… It may be a hidden thing that, actually, we make a lot of cognitive biases, but the rest of the community don’t know enough to know that we are actually making that cognitive bias. So I think, uh, yeah, as, as most, like, the OMI, but, like, all of us are still making cognitive biases about occlusive myocardial infarction that doesn’t have ST elevation, right? So similarly, I think, uh, um, I think we continue to make a lot of cognitive errors, uh, even as we are senior, it’s just that we don’t know it.

00:47:43

Um, and then, of course, the, the common cognitive errors that we have is minimised because we learn, but there are new cognitive errors that we pick up or we don’t know until maybe some time in the future.

But the… I think the other thing is also, we learn to be safe, even though we realise that we make a cognitive error. Uh, we may… We allow ourselves to make cognitive error, but our decisions try to be safe. So, like, for example, I don’t know what’s wrong, this chest pain or this abdo pain.

MO Hmm.

P20 But then we learn to make the decision that, hey, because I don’t know, I’m going to either observe this patient further, or I’m going to admit this patient for further evaluation, for example.

MO Hmm.

00:48:22

P20 So we learn to be safe, even though we accept, I don’t know everything. So, so that is, uh… Yeah, uh, that’s what I’d like to say. But, uh, I, I’m just wondering whether the rest of the participants, um, you know, feel, or think, you know, similarly. Or differently.

P21 Uh, Participant 21 here. So, so that’s why it is a, a process of continuous learning, lah. Uh, you, you… If you look at certain conditions that appeared at M&M, uh, and you say that no one could have made these diagnoses.

Uh, and then, if, next time, if you see a similar patient, uh, where much more common diagnosis is, uh, possible, you’re not going to say, I’m going to exclude every rare… every person with this rare condition. Otherwise, your life will be, one, very miserable, and two, you actually, uh, will be over-investigating, too much resources and potentially a lot of false positives could come out. And, and you are subjecting a lot of patients to unnecessary processes, like specialist clinics, which are already overloaded.

Uh, of course, the other way, is Iike… Uh, uh, uh, uh, everything I disregard, also cannot lah. You will miss things, lah.

MO Hmm.

P21 So that’s why I say, uh, uh, you have… The, the really, uh, uh, master, uh… The only mastery, uh, uh, is to have a balance, uh, between both extremes. And that is where, uh, we have to distinguish ourselves, lah. Uh, because if we, uh, go… Swing to other extreme, uh, and our students as well as our juniors are looking at how we make a decision, uh, they’ll be quite confused, uh, you know. Uh, so then you’ll end up with a, a generation of doctors who either over or under investigate, lah. Uh, okay, thanks.

00:50:28

MO Thank you.

P22 I mean, I, I just answer, uh, 20, lah, uh, you know. So, uh, I agree, but I also have a different view, also that because, uh, this sort of thing may not apply to the question what I’m trying to say, is that the… I think… Because, like, like, you know, it’s about coping mechanism again lah. So some… Even young consultants, right, when they first see a complex case or what not. Uh, or, or even a senior resident or whoever it is, you know, that make that, lah, they tend to… they may go systematic because that is their coping mechanism first. While we…

While, when you are more senior, you get more and more targeted. And that is also the dangerous part when you get more and more targeted, because you, you actually… That is where the… We had to be aware, including ourselves, that that’s the framing bias and all this coming, lah, because we are now targeting to a, to a system immediately, based on gestalt.

So that’s why I think, in medicine, this is my own belief, that, that… And, uh, I, I apply it even, even when I, when I had to counsel people, right. That is why, um, those that do not have gestalt that means that when they don’t… they don’t have the experience, right? That’s why we’ve got evidence-based medicine and scores to come in to, to replace, to replace our experience with this to, to complement and trying our best to reduce bias based on the best evidence we can give to our physicians to arm them with, lah.

00:51:51

So I think, like what 20 has mentioned, I, I totally understand where it’s coming from because I, I also think, honestly, we are the, the, the younger… Because the way our… The medical education system has changed a lot as well. Last time, when we were, when we were, uh, trained, we were all about role… We were actually… If you… I think of it, right, it’s all about role modelling. We are following role models that we want to be, right? But then now, the way we are training in the residency is about… Is a system, is about being systematic.

And, and, and I think that’s, that’s why, uh, even for, for myself, I sometimes struggle to balance. Because I, I, I come from an era of even… I come from an era where we were taught about… By role modelling. I see what people do, I want to be like this person. I don’t know whether you understand what I mean. While the younger ones, right, are, are really very systematic. You can go and see how they do. Even the consultants, they are very systematic. I do this, A, B, C… You know, the, the… You know what I mean? The, the, the… Because that is the way they are taught, and it’s not wrong.

So I think, uh, in the end, right, um, uh, it is our… Uh, I… As the Heads also, I think it is our duty to try to balance these together, lah. And I think that is the way they are coping. The, the… That is the way… That’s the way the younger consultants try to… Try… The, the more, the more, the, the more experienced they are, right, they will tend to more towards to targeted one. So that is… For them, it’s a transition, when they transition over. I don’t know whether you are… Whether that makes sense, uh, but this is how I, I see it, lah.

00:53:20

But the… I’m not saying the, the young consultants are, are not experienced. Or, in fact, they, they are… Being systematic is not wrong. In fact, it’s maybe good because they are seeing it as a big picture. While for, while for us, that’s why we are to work in teams, right, to reduce biases.

Because for us, the more senior we are, we, we may have our… We, we… I, I take myself for example, I confirm we have biases. Uh, you know, because I have really… I am really relatively as a senior, I will definitely have some, some, uh, uh, views on certain type of cases I see because this is my experience.

But the… That’s why when we talk, when we discuss all this, uh, you know, when we get… When we discuss with a, with a team, everybody got different views, and we add our information together to reduce the… To reduce these errors, lah. You know, that, that, that is why there are team systems involved, lah. And I think medicine is moving towards that, instead of being a, let me say, a solo-physician-led, lah, to combat all these type of issues you see. Yeah.

MO Anything that anybody else wants to add?

00:54:27

P20 Uh, I… Participant 20. Yeah, I agree with, uh, what, um, Participants, uh, 21 and 22 have said. It’s not, uh, easy, and, uh, you know, and of course, uh, I think, uh, there is overwhelming knowledge, and therefore, you know, from the beginning, we simplify it and, uh, we… And, uh, we… I mean, of course, and in early medical school, we, we, we give very simplified, straightforward information to the students, and they pick that up. Uh, and then, of course, as they become associate consultants… Uh, sorry, registrars or residents, you add a bit more, and then they add a bit more with more experience and all.

Um, sometimes, we, uh, may or may not overcome the, the, the pre-existing biases, but, uh, but the way that we train, we become better and better at the typical things, lah. But we may not have, um… And we may occasionally meet with the atypical things. Uh, yeah, but they don’t hit us as much, maybe because, uh, as juniors, they get protected by the seniors.

But when the seniors, uh, become AC, then the atypical things may haunt them because the typical things don’t haunt them anymore. Although, they may occasionally, because of framing and all, be misled. Um, and then as a team, sometimes, also, you know, um, if they are not… I mean, if they are not, um, listening to one another, then, uh, then, you know, the… Maybe the one with the strongest voice will, will make the decision, and, and if the decision is wrong, then that will be an error as well, lah.

So I think yes, uh, I agree with all what they have said. And also, the coping part, lah, which is, uh, probably also everybody has different coping strategies. So, um, and, uh, yeah, and some maybe cope better than others. And, I don’t know, maybe we should think of how to teach people how to cope.

00:56:23

MO Thanks.

P21 Uh, hi, Participant 21 here. I, I myself, uh, uh, have, uh, tried, lah, to at least, uh, make it more, uh, practical so that everyone can have habits, uh, to bring into their working life after they leave the department. For example, if they go to fam med or…

MO Hmm.

P21 Ortho, GS, etc., lah. And that is, uh, to avoid, uh, uh, uh, just limiting yourself, uh, uh, or the many biases. Just remember particularly, number one, uh, let us exclude the life-threatening causes first…

MO Hmm.

P21 Uh, that are common. Okay? Uh, and then never limit yourself to one. Try to make at least three or four diagnoses. And you can exclude them even on reasonable clinical grounds that’s acceptable, lah. Then finally, there’s a group where you actually don’t have the diagnosis but, uh, you think you have likely excluded the life-threatening things. Then you make a informed, shared decision with the patient and say, look, when you go home, look out for these red flags.

MO Hmm.

00:57:34

P21 Okay, and in the meantime, we refer you. Uh, that, I think, uh, uh, is a kind of a, a, a working model, uh, uh, of a, of, a practice habit lah, that they can carry on into other specialties, uh-huh. Uh, don’t close yourself off. Think of about three or four, uh, diagnoses that need you also to exclude life-threatening common ones.

Then after that, lah, uh, if you can make a diagnosis, uh, good. If you cannot, and you think the patient don’t need to be admitted, uh, uh, then, like I said, lah, uh, shared discussion making, discuss the red flags. Those that are life-threatening, unstable, and you don’t know the diagnosis, okay, that one’s also all right. You, you admit to your colleagues. They will work it out, okay?

MO Hmm.

P21 So I think this is, uh, what I think is practical advice, lah. Uh, in A&E we cannot make any diagnoses, and there will be always be mistakes. Uh, if you show me a consultant who has never sent a patient home and died, uh, then the consultant probably has admitted everyone, uh, to the ward, uh, okay? Uh, uh, thank you, lah. Thank you.

MO Thank you. Uh, okay, so, uh, I think, uh, um, that was quite good advice. Um, this advice, I mean, uh, you, you say, uh, it’s advice that you share with the medical officers rotating through the department, uh, going on to fam med, GS, ortho, etc.

But, um, would there be any specific advice that you would give, uh, new specialists? Because I think we, we agreed that, you know, uh, during this transition phase when they become, um, uh, junior, uh, when they just get their licence and they become, uh, junior ACs, that’s when they are the most vulnerable. So, so would there be any specific advice that you will give, uh, this group of, um, specialists when they are vetting through, uh, or supervising cases?

00:59:29

P21 Uh, okay, so me again. So I know… I think I, I made this kind of joke with you guys before, lah, really, because specialists, uh, I wish I could give you a T-shirt, uh, that says, don’t be a hero. Okay? Just because you suddenly one day pass exam, uh, doesn’t mean you are super ready. No.

You still be cautious. You still don’t take unnecessary risks. Okay? Uh, still behave, uh, like a, a senior resident for the next few years, lah. Uh, then, uh, as you find your own balance, uh, of how much risks you are willing to take, uh, and then you’ll be happy, lah, with where, where you are in the ED, lah. Uh, uh, honestly, the ED, uh, our minds… Our way of thinking is quite different from the ward doctors, okay? Uh, uh, it’s all about managing risks.

So I still say, uh, still think, lah, like a resident, uh, when you become a specialist. And even when you are a super specialist, uh, listen again to your juniors, uh, uh, uh, and, and also the patient, lah. A patient may have concerns, uh, that, uh, suddenly, you realise, uh, hey, this is different from what we were told. Thank you.

01:00:43

MO Thank you. What do you all think?

P22 Um, 22 here. So I… For me, right, uh, my personal method is when I talk to incoming ACs, lah. Or when the SRs are going to the AC, I will be very frank to tell them that, that is your… That is the time where you have to be more careful. And, and I just tell them honestly that, that, uh, it’s because, uh, based on our experience, we have… Uh, we, we, we, we have, uh… We have seen incidents happen before during this period of time. It’s more of… You know?

And there could be many… I will tell them, lah, you know, basically, uh, in a… In… It can be, or whatever you call it, you know, more comfortable area to tell them that, yes, that’s because maybe you are more confident because you exit, like what 21 say. You know, but, uh, we just have to be more aware.

So I think it’s sometimes a… I, I think what I, what I would like to… What I’m trying to do for them is, they have to be more aware of this potential before. Uh, you know, and that’s how we… How I think we can try to, uh, protect them, lah, you know, in that sense, lah. Because they already have… They already have habits. They already have the way they wanna do things.

But it’s our… I think all we can do is give them, like what 21 said, is to give advice, uh, and to tell them that, during this period of time, just be a bit more cautious and, uh, have a better insight to what you are doing, lah. Uh, that’s the word. A better insight to your, your, your behaviour and your thought process, you know, uh, where you are doing certain things. Especially during this transitional phase, lah. Yeah, that, that’s, that’s, that’s, uh, thing. That’s what we can do. That’s what I do, lah. Yeah.

01:02:26

P20 Uh, Participant 20. Yeah, uh, yeah, when, uh, somebody asked me, uh, recently about, uh, you know, becoming a AC, you know, what’s it like, what should I do? So I, I did advise, lah, to the person that, you know, um, that she should, uh, I mean, um, be always learning and, uh, be aware that, you know, um, she still… I mean, she may have, uh, passed exams and all, but there’s still a lot of things that she may not know, and, uh, she probably doesn’t know, and it’s an ongoing process. So it’s a lifelong journey.

And also, um, to… You know, she will be… I mean, so some of the things that, a, a, a person may end up doing because, now that she’s gotten a licence, she may be over-confident because of her skills because she’s passed exam, people have given her a licence, people have, people have certified her as being independent.

So, so she, I mean, should be aware that… So awareness is very important that she may commit, uh, cognitive errors or, you know, she may make decisions, um, that, yeah, may, um, for various reasons, uh, that may be, uh, uh… I mean, uh, erroneous, and that may be due to many, many factors, lah, could be internal or environmental, uh, factors, lah, like being very distracted on the shift, having many ECGs’, uh, thrusted on you, and then, you know, having to… So I think she… So awareness that, uh, things will affect her in her decision, so therefore, she has to be mindful.

01:04:05

Uh, and also, if she’s not… And she was faced with, uh, conditions where, where she doesn’t actually know. She maybe knows something about the condition, but she doesn’t actually know the exact condition. So this may be, like, atypical presentations, uh, because, uh, now you are trained with all the typical presentations, but you still don’t know much about atypical things.

So to ask your fellow peers to… I mean, to discuss with them to have a second opinion with them, and don’t be afraid to ask. Because very often when you exit, you are afraid because what will people look at you… Uh, how will people look at you, because, uh, now, uh, you know, now you have exited, supposed to be independent, supposed to know a lot of things.

Um, but, uh, yeah, but to be humble and, uh, to ask if you are not sure, and ask, you know, ask to discuss with a peer or for a second eye or second opinion on any… Uh, on a condition or ECG or an x-ray, something like that, lah. So, uh, mindfulness, asking, uh, for help, uh, not being afraid to ask for help.

Yeah, and, uh, yeah, and then being aware, lah. So being aware, uh, uh, like, uh, of myself being… I also tell myself, be aware that I can… I’m prone to cognitive bias. So when I’m more aware that I am prone to cognitive biases, then I will go, go and disconfirm all the… You know, do some disconfirming of the, the things that, uh, that I may have originally, uh, made a diagnosis of, or in an impression in my mind. So, and have multiple differential diagnoses of different systems to look out for.

Uh, so yeah, and I, and I also tell them, uh, that, uh, it’s like a game, lah. So every day, we come to work, and it’s a game of trying to, uh, you know, uh, make the right diagnosis. So sometimes, I even find it as a team to, uh, see whether my diagnosis is different from a fellow senior. Uh, because, you know, it is interesting to disprove something that somebody else has, uh, has, uh, has made a diagnosis of. Like, you know, like a special ECG or for a special x-ray or, or some, some other… Especially the atypical conditions. So, um, yeah.

01:06:22

So, uh, uh, then that will, you know, not make it so… So coming to work as a game will not make it, uh… To play a game will not make it as, uh, so dreadful, lah, as some people might say, every time you come to shift, you feel terrible and, yeah, because all the numbers and all these things. So, I, I try to reframe by making it, uh, I’m coming to play a game. Uh, a serious game, but, uh, interesting to me, enough to, you know, want to play it, and, yeah, every time. Yeah, that’s… Thanks.

MO Thank you, Participant 20. Anything else to add? Uh, then we go on to the last question. Yeah, so, uh, I think we brought up, you know, about awareness, about having insight. So, so, uh, what would you say is the role of education, uh, about cognitive errors, cognitive biases? And also, uh, when would be the most appropriate time to do it? Would it be, you know, um, uh, in medical school, or during, uh, medical officer posting, or for the residents, or after they exit and then they transition to the vulnerable stage in AC?

Sorry, uh, uh, long question. Basically, what’s the role of education for cognitive biases, um, and then also, uh, when is the best time if, you know, if at all? Yeah.

01:07:55

P21 Yeah, okay, uh, uh, Participant 21, uh. I, I, uh, I, I think, uh, the best time is actually during the medical student days, uh, when they are being… Okay, we call it elementary clinics at that time. I don’t know what it is now. But when you are, uh, orientated to the wards, and you start to take history and physical and formally diagnoses, uh.

MO Hmm.

P21 Uh, that’s the time, I think, uh, uh, we should introduce to them, uh, the concepts, uh, of human factors, uh, in decision-making. Uh, ‘cause nowadays, uh, uh, everyone, uh, is, uh, going towards system-based practice, uh. Uh, you can do that so that, uh, when they are, uh, still, uh, formulating their diagnoses, uh, then they will… don’t hit on rare things, but, uh, what could affect their diagnoses, uh, the common things occur commonly, first, uh. So that’s the one time, lah.

Then probably another time, uh, when education could step in, lah, uh, is, uh, during, uh, their house… Just before they start housemanship, okay? Uh, one more time, all right. That should stand them in good stead, lah, when they go to professional life, uh, be it, uh, a GP or a resident, lah, and then future specialist. Because they are young and impressionable at that time. Uh, that’s my view.

And then you illustrate lah, with as many examples as you can from real life, lah. Okay, uh, including, uh, cases that have, uh, bad outcomes that they could help, uh, them see, oh, this is what happened when that specialist made a, a bias, uh… A diagnosis based on a bias. Uh, that would really hit them. Uh, that would really make an impression. You know, stories, uh, make a deep impression. Thank you.

01:09:44

P22 Uh, I, I, I think education is important. Uh, I think there are two factors here, lah. I’d say number one is how… Uh, sorry, three, uh. Number one is how the education is delivered, lah. That means, uh, it’s how we teach it, you know. And I agree with what 21 said, one is during house officer. One is doing… When, uh, just a reminder at, uh, when they turn HO, maybe the induction programme, you know, just to let them know. And then when they are in residency, there should…

But it’s a matter of how we deliver the … this human factors thing in, lah. Because if it’s too didactic, people cannot relate because it’s very, very dry. So, so, uh, I think we have to learn how to… We also have to find… The second part is how do we want to include this into the, uh, uh, curriculum, or, or how do we want to teach, uh, the method that we would teach it, lah.

The third thing, I think, is that… Is the, the people that we are supposed to educate. I think we, we can’t, we can’t only educate the young. We have to re-educate the, the more senior ones. The reason because the, the more senior ones have never been taught about human factors. As in, we… This concept, ah, we… The, the… Like, for example, now we are talking all these biases or whatever it is, I, I don’t know how many people have been taught about this previously. The answer is, I think, very little. Because this has never been a part of, of any, any of the more senior people’s curriculum.

01:11:12

And then the next question, and I think it’s the reason why it’s important, because as we, as we recognise this, it needs to be such that everybody recognises it together. Because, uh, if you let the young one recognise that they are in conflict with the older ones, it will get even worse. In fact, there will be more errors.

So, so I think we, we have to… We, we have to also, uh, have a more… Uh, have a education… Uh, we have to learn to educate. Again, it should not be too didactic. It should be something that, that is inculcated… Like, like, you know, like, like what 21 said. Like, maybe scenario-based or, or, like, you know, a, a, a person who is trained in human factors, that now there are some people that can… They are able to deliver it, uh, more succinctly and more relevant, lah, for day-to-day use, uh.

And, uh, the other group that has to be… Uh, I don’t know, the other group that has to be educated is who are the ones, the people who are going to educate these people, lah? So the reason why I say this is because, uh, not everybody can… I, I honestly think not everybody can, can teach human factors. Really, I, I think it’s a, it’s a very, very, uh, difficult topic. And it takes long.

So, so for example, you know, lah, in our department, right, there, there was, uh, I think, one of the… Uh, in fact, there was a few times where the human factor expert came in, came and, uh, uh, teach us, right. Uh, you know, I think one of our senior consultants, uh, invited some… One of them to come and talk. And I think the guy who taught, he was very relevant because he was able to… The way he was able to share the information with us, what is important. You know what I mean? Because he's able to put it in a case-based scenario. How to teach. Uh, no, how is it relevant, all this.

01:12:41

So, so, it is about trying to… When you educate, also, you must be cognitively accepted by people, you see. The… I, I think that is, that is a very important thing. Because, uh, you know, when you ask our residents, uh, when we start doing the…

I’ll give you an example, the best example I’ll give is wellness, lah. I, I’ve got to be honest. Like, in wellness, right, you go and talk to them, they cannot see the relevance in all these wellness things, you know, if you ask them. Because, because it’s not something you can educate them. You, you, you know what I mean? It’s something that has to be practiced by the department, be practiced at the workplace and practiced as a whole, lah. You know, you know what I mean? It’s, it’s not something I can teach you.

So I don’t know whether human factors is… Maybe it should be incorporated in the… Uh, they… What, what, what, what is it called? Uh, uh, this… Uh, I can’t remember. Cased-based, uh, uh, case-based discussion. Uh, you know what I mean? Uh, it’s the way you discuss, so that people can understand how these human factor things, uh, uh, are in play. You may not need to be… Uh, uh, so, so this is… I think that is the… That’s the most important thing.

01:13:39

It’s not, it’s not about teaching them didactics to tell them... You can tell them, lah, a bit, like, yeah, bias bias bias, then how? But I say, okay, everything is bias, then how can people understand? Do you know what I mean? It needs to be taught such that it is applicable on the ground, lah. Uh, you know? And people can understand the relational, yeah. That’s, that’s my two cents’ worth.

MO Thank you very much. Yeah.

P20 Uh, hi, Participant 20 here. I agree with, um, Participant 21 about when, uh. And I agree with Participant 22 about, uh, you know, uh, who. I, I, I just, uh, expand a, a little bit more, lah. Um, um, basically, yes, uh, I think, uh, there are few, um, groups of people or… That need, uh, uh, education. Firstly, like Participant 21 has said, uh, medical students, when they come in, uh, elementary clinics and so forth, um, that they are, you know, exposed to the idea. And, uh, how it’s done, of course, hopefully it’s done in, in, in a, in a way that they can, uh, relate to, and is relevant to them.

So I think, um, uh, I think also in the beginning of medical school, because when we enter medical school, I, I feel that those are formative years, we are very impressionable. So we need to be given a, a, a, an idea about what our life is like as a doctor from being in medical school right up to being a consultant so that we have an idea.

And for example, while I’m… We are learning these, and actually, it is phrased in a simple way so that you can absorb. But actually, there’s much more in medical… There’s much more in medical school and, and in medical knowledge, uh, that you will never, you know, finish learning. So, so there is no end to it, and you will… And you are building upon it. So that people don’t, like, finish medical school and think they know everything. And neither do they finish residency and think they know everything. But they are framed to see that there is a lot out there, and you are actually just building building blocks.

01:15:48

So, so, you know, uh, so don’t, uh, don’t be surprised that, you know, why you learn something new, why you suddenly make a cognitive error. It’s because you didn’t have either more knowledge, more experience, exposure, or you don’t know the atypical presentations, for example. So that is in a… So they need to know, uh, actually, it is a journey. And, uh, you know, this is not all that I need to know. Uh, this may be, uh, what I need to know for the first phase, but there are many phases in my career. So that is, that is that.

Then, number two. Um, I feel that the people who need education is, uh, the faculty too. Because the faculty are our role models. The students have nowhere else to go except to look at the faculty, especially in the beginning. When the students are senior, like, residents, senior residents can tell it’s a good faculty, this is a bad faculty, I don’t want to follow. You know? But the junior, when they are medical students, they don’t know, so whoever you put in front of them, you know, that’s who they follow.

01:16:42

So we need to train the faculty to be good role models, uh, so to not, like, in the early years, tell them, go and just take a few focused questions, and that’s the end of it. And then once you get a diagnosis, you’ve hit the mark, very good. You know? So I think, uh, the role, the role models have, have to really… You know, the, the teachers have to really be trained, because otherwise, they are making the juniors, the, the, their students be… Um, fall into all these errors, cognitive errors. Because we make them fall into errors by making… Training them in a certain way. So the faculty also need.

Because, unfortunately, uh, the evolution of medicine has been such that, uh, we’ve been picking up things. You know, uh, our early fathers of medicine, whether it’s Hippocrates or, or some other people, you know, they, they only learnt medicine in a certain way because they didn’t have investigations. They didn’t have x-ray, CT scan, blood results. So whatever the obvious symptoms, uh, the patient has, they have written it down, so it is a kind of framing that they have done for us in the next few generations after them.

But now there are new things like technology, uh, that gives us diagnoses much faster. Of course, now technology is expensive. You know, blood tests are relatively expensive. CT scans are relatively expensive. But one day, it will come, it’s gonna be very cheap and easy to do, and low-risk and safe. Okay, as, uh, we evolve, I think you need to also change the way medical education is delivered so that we have less cognitive errors. Uh, and, and, uh, if something can take away the error, it should.

01:18:25

So we may have to reframe the whole of medical school, uh, how we teach how we practice medicine. So, because, like, you know, last time we say it is acceptable to have a 30% negative appendicectomy rate. That means you… That means you would say, all right, I think your operate is, is negative. Is acceptable to have 30%. But now with technology, you do a CT scan, you have 100% or near 100%, you know, uh, positive rate. You know, that is so much better for the patient, right? The patient who really, uh, needs the surgery gets the surgery.

But the old people who have been trained in the old way keep saying, why is it that the patient needs to wait for a CT scan to, uh, to get the… Uh, before he gets a, a, a, an appendicectomy, where he so obviously has RIF pain and tenderness, right? So, so the, the, the older generation have been trained in a certain way, and they think of it in a certain way. So… And they think that it is acceptable that 30% are negatives, negative appendicectomies, uh, negative, uh, uh, surgeries for, for appendicitis, right?

Whereas, the, the… Now, with CT scan, you can have near 100%, uh, uh, success rate. So the person who doesn’t need the surgery doesn’t get it. So I think… So technology has come in to, to change the way medicine is practised and could perhaps also change the whole of, you know, medical, uh, teaching, uh, in the future. Because, um, we want to have less harm for the patient, lah, and we want to get the diagnosis quickly, efficiently, and treat the disease early. That is the… If you see us from the patient’s perspective, that is what they want.

01:20:09

Of course, the physicians are all… Uh, and, um, and the administrators are holding back because of other reasons like cost and, and other, uh, radiation reasons as well. But the paradigm, you have to shift. So I think… So the whole of medical training may have to change, maybe, in the near future.

But currently, what we have left, we need good role models as teachers, and we, we need to tell the students, you know, uh, in the future, uh, what the whole of, uh, how they should envisage their medical school life is. And at every different point, uh, along the way, you know, the teachers should… You know, could… If, if the, if the clinical teachers could bring up the rare examples that they face, that would be great. Uh, um, you know, uh, and of course, occasionally, they may have all these, uh, human factors teachers coming in to consolidate, um.

And, and maybe the emphasis should be along the way as clinicians, uh, by clinical teachers who will point out all, all the, the potential errors and errors that they have made. Um, and of course, uh… And maybe if that is done well, then they will not need special teaching sessions at various time points. But perhaps now we need special sessions at various time points like whether, just before a VPS, just before, uh, you, you finish residency or whatever.

MO Hmm.

P20 It’s because we don’t have those good teachers who would mentor them, uh, as role models along the way. So that is what I, I, I think, uh, yeah…

01:21:35

MO Hmm.

P20 About, uh, education.

MO Thanks, Participant 20. Uh, anybody else has anything else to add? Usually we, we have reached the end. Uh, any last, uh, things that anybody else wants to add or wants to bring up?

P21 Uh, we, we are only talking about cognitive bias, not, not, not system improvement, huh?

MO Um, both, I guess, because part of the… I mean, there have been system inventions that have been put in place to reduce cognitive errors, I guess. So it’s a bit of both. But the aim is… Uh, the focus is on cognitive errors, I guess.

P21 Oh, okay then. Okay, then I limit myself… Will limit myself to that. Thank you. Thank you.

MO Thank you. Yes?

P20 Uh, I want to bring up an interesting thing that I also thought about, which is spot diagnosis, uh, exams and all this. So it’s a kind of framing bias that we put ourselves in, and maybe the, the education system also. You see a patient, all, uh, these few things, you make a spot diagnosis, you know?

MO Hmm.

01:22:46

P20 So I think if we have all been trained to look for the spot diagnoses, and then, therefore, we, um, reinforced the ability, uh, or the… Our forming cognitive biases. So I think we sort of set ourselves up, also, for cognitive biases.

MO Hmm. Yes.

P21 Yeah. But actually, uh, uh, how should I put it? I, I think most of the times, spot diagnoses are made for relatively straightforward things, uh, that are based on images or physical features, lah, like a, like a, dislocated shoulder, ah, you can see the squaring, right? Or you see the, uh, woman, elderly woman fall down, and then dinner-fork (deformity), ah. Or, or a ECG shows a STEMI, uh, uh, those okay, okay to make a spot diagnosis, lah.

Uh, but when you have a abdo, uh, pain, or if you have a patient with, um, haematuria, uh, those, I don’t think we’ll adopt that thinking process. ‘Cause, ‘cause usually, like I say, lah, if there is a clear-cut clinical image or physical sign, lah, or, uh, ECG, lah. Uh, x-rays of course, lah. Uh, bowel obstruction can be a spot diagnosis if it’s volvulus, uh. Uh, okay, maybe I’m not… Maybe I’m not thinking the same way as Participant 20. Okay, thanks. Thanks.

P20 No, actually, I was thinking of the student… Uh, the patient that you put there, and then for the exams, and you walk around and make your spot diagnosis. So, you know, lah, whether the patient has, uh, you know, certain features and all these things.

01:24:44

Um, but then, you know, it’s sort of like, um… So we, we are learning also by, um, uh, pattern recognition and all this because of all of these emphasis, we sort of, you know, unwittingly make ourselves, you know, look out for all these things. But then it will also make us, uh, neglect to look for other things, lah. So I’m just saying that it may make… Frame us towards, uh, making, uh, cognitive errors because of the way we are trained.

P21 Oh, okay. Uh, then, yeah, okay, then I get what you mean. Thanks.

MO Okay, I…

P20 Uh, wait, I, I forgot to say… Oh yeah, as well, um, I, I just wanted to, uh, add that, uh, I think with, uh, environmental reasons also, um, can cause us to, um, make, uh, errors, lah, like, you know, very busy department and all this. So, uh, yeah, even if, as well trained as you may be and as aware of cognitive biases, sometimes there is a limit to…

MO Hmm.

P20 What anybody can do. And if you have three people lining up to, to, to get your attention, you know, and you are in a hurry and suddenly something happens and you need to attend to that immediately, then the other three things, you, you sort of, like… Yeah, you sort of, like, have… Uh, listen partially because of, uh… Then I, I think it is, uh, uh, uh, something that the… Hopefully the system can help, lah.

01:26:19

Because it’s not fair to the expert clinician to be overwhelmed with three or four tasks at the same time, and then… And blame him for a cognitive error, if that is what happened, uh. So I think, uh, then in that case, actually, the system has a part to play, because it is not possible, uh… We should not put an impossible task on, on the clinician. But to minimise it, lah.

MO Uh, would you say it becomes a excuse in that sense? Because, like, every time an M&M happened, then we say that, oh, but, um, resuscitation was, you know… The resuscitation area was very busy. There were…

P20 Hmm.

MO Four other sick patients. Or you sign an ECG wrongly and say, oh but then I had three other ECGs thrusted at me.

P20 Yeah, uh, yeah. So I think, uh, yeah, so it all depends. I think if you, uh, um… I mean, honestly, I would feel that there, there is some role to play, the busyness of it. But, uh, I think there will also be situations where you are not busy, and yet you still…

MO Hmm.

P20 Uh, you know, make the same cognitive error, um. So… And it’s because of the way we frame it. For example, if… Sometimes when you are not busy and you get the Occlusive MI ECG. That’s what happened to me, uh, and a consultant recently, that there’s a, a occlusive myocardial infarction ECG. There’s no typical ST elevation, but, uh, you know, but, uh… So the, the, the consultant saw it and then thought it was something else, and went down the PE path, um.

01:27:52

MO Hmm.

P20 So, yeah, and the person was not busy. And, uh, you know, and I was relatively not that busy, but I, I…

MO Hmm.

P20 Decided also, I don’t want to… Uh, at that point in time, I had the first started shift because there was a handover. I didn’t want to, uh, go and play the game of disproving my consultant.

MO [Laughs].

P20 Because I wanted to make sure that the shift was okay. And the, the consultant has gone down the path of so many things waiting for blood tests, so he was quite late in the diagnosis already. Uh, so it was like a diagnostic momentum. So he was so late in the diagnosis already that, uh, now order CTPA and asked me to wait for it. So I was not at that point in time playing my game.

So, so, later when I started realising it was one and a half hours later when the CTPA was reported. Then, uh, then the patient had altogether five hours of delayed PCI. Because after that, I activated the cath lab when I, when I looked back at everything.

01:28:49

So, yeah. I mean, at that time, yes, we’re a little bit busy. But I was not having three ECGs shoved in my face, and neither was that person. So yes, it still happens. So yeah.

But I think we should not use it as an excuse because, like, uh, recently also, I had a, a consultant friend who asked me, um, you know, about, uh, you know, a, a patient that she saw. And then she asked me… Then I said, oh, but you were busy. You had… Then she said, but busyness is not an excuse. She says busyness is a weak excuse.

So, I mean, I admire these fellow consultants. Uh, because I was trying to… I was trying to, uh, console this consultant. So I said, no, lah, you were busy. And then the consultant said, no, uh, this is… Uh, it is… Busyness is a weak excuse. So, yeah. So everybody knows whether they were busy or not busy, but it's only in their heart, yeah, that they will admit it or not. Thanks.

MO Uh, anybody want to add anything else? Otherwise, yeah, thank you very much, everyone, for your time and, uh, for joining us today. Um, lah, we’d like to assure you once more that the responses will be kept strictly confidential. Yeah, thank you very much.

P22 Okay, thanks, bye-bye.

MO Thank you.

P20 Thanks, thanks.

P21 Bye-bye.

MO I stop recording first.

01:30:17
