# Supplementary material for: Dropping the baton: Cognitive biases in emergency physicians
Source: PLoS One. 2025 Jan 2;20(1):e0316361. doi: 10.1371/journal.pone.0316361 (PMC11694980; doi:10.1371/journal.pone.0316361)
Supplement: S3 File — (ZIP) [file pone.0316361.s003.zip › Transcripts/FGD 7.docx]

FGD 7

Speaker Key:

MO Moderator/s

P Participant/s

00:00:04

MO1 Okay.

MO2 Yeah, I have started.

MO1 All right, um, great. So, to start off, uh, maybe we can ask each of you, um, how long you have been an emergency physician. Uh, maybe just give a rough estimate. Less than five years? Like, or ten, more than ten years? More than 20 years? Maybe Participant 24?

P24 Yeah. For me, uh, it’s about 20 years, lah. Coming to 20 years, yeah.

MO1 Okay.

P24 After exiting, it’s about 20 years.

MO1 Okay, thank you. Participant 23?

P23 Uh, 23 here. Um, I think about 30 years. I think 30 years.

MO1 Okay, thank you. Okay, we’re still waiting for Participant 25. Uh, okay. But, uh, perhaps, uh, to, also as an opening question, I would like to ask you what your understanding of cognitive errors are.

00:01:11

What do you understand by cognitive errors?

P24 I, uh… Maybe if it’s okay if I start first?

MO1 Yes, of course, Participant 24.

P24 Uh, I think when I hear about cognitive error, often I’m led to understand it from the perspective of, um, diagnosis, lah. Diagnosis-related, uh, decision. Uh, uh, I mean, determining a diagnosis.

Uh, but I think it’s a bit broader than that, because we are looking at also other, other clinical decisions that we make, lah. Right? Uh, for example, ordering a test or, uh, providing a certain treatment over another treatment, lah.

So, so I think error in the sense that we, we, we made a wrong decision. And, and the, the question is, uh, how did we end up doing that, lah? [Laughs]. So, uh, you know? So, that’s how I view cognitive error, yeah.

MO1 Yeah, okay. Good. Uh, Participant 23, maybe you can share with us your understanding of cognitive errors?

00:02:21

P23 Uh, 23. Um, so, um, I, I, I agree, it encompasses many different, uh, aspect of our work. Um, my, my own interpretation of cognitive is that, um, you know, um, in our line of work, that we, we, we do a lot of things instinctively or in a sort of reactive manner sometimes.

And, uh, System One’s sort of kind of a, you know, kind of decision-making and, and, and, and, and, and, and analysis. Whilst I, I, I think I, I would put cognitive, uh, you know, uh, uh, errors and, and, and, and, and everything else into the more system, System Two sort of thing, where there’s, where it goes into the brain.

The information has to go into the brain, and then the brain somehow has to process it, and, and, and I think that is the, the… I think, I think that’s the way I interpret cognitive, uh, you know, analysis and errors to be made. Over.

MO1 Okay. Thank you, Participant 23. Okay. Welcome, Participant 25. Uh, we’ve just started on our, uh, introductions only. So, um, just to, uh, briefly share with you that we are a group of emergency physicians, um, uh, doing, uh, research, qualitative research on cognitive errors, lah. And we thank you for your taking the time to come and sharing with us, uh, your insights.

So, um, as we are on Zoom, so there are limitations. So, and also, we would like to preserve, uh, anonymity. So, um, perhaps you could refer yourself, when you speak, as Participant 25.

00:04:05

Uh, everybody will refer to themselves by their numbers before they, they give their, um, uh, perspective. And also, we try to let everyone have a go at, um, speaking, so, uh, we will take turns and try not to speak over one another, because of the limitations. But we will definitely get back to everybody, uh, who, you know, has something to say, lah.

Okay. So, um, uh, the question that I just asked the two participants is, um, what is your understanding of cognitive errors? If, Participant 25, you’re ready, maybe you would like to share with us?

P25 Um, I think if you’re referring to cognitive errors from a healthcare worker’s perspective, then I think it is, uh, understanding whether, or, or trying to know whether the person understands, uh, uh, what, what is happening in the department, happening with patient care, and is aware, uh, of the environment. That means, uh, understands what’s happening in the environment, for the person to function effectively as a member of the staff.

MO1 Okay. Um, thank you. So, I, I will just give a brief, uh, like, um, description of what, uh, cognitive errors are. Um, okay, so cognitive, uh, shortcuts are often used to help us along with our decision-making, and, uh, this makes complex decisions, uh, easier to manage.

However, these, uh, shortcuts or heuristics can sometimes lead us astray and make us commit, uh, great cognitive errors. So, cognitive errors are actually very common in clinical medicine, and everyone regardless of seniority is vulnerable to them.

00:05:57

Uh, moreover, they are insidious and difficult to recognise, which is, which makes overcoming them not so easy.

Um, so we hope that, uh, we, today we can work together to, um, better understand about cognitive errors and what we can do to mitigate these errors, uh, in emergency physicians.

So, um, to start off, uh, everything starts from the beginning. So, we’d like to start from the beginning and, um, maybe have you, all of you, think of the time when you were in medical school or training in, as a medical student, uh, with didactic teaching or clinical postings. You know?

To what extent do you think that the medical school posting or attachment can have an effect on cognitive biases or cognitive errors?

P23 May I? May I start?

MO1 Yes, Participant 23.

P23 23, yeah. Um, if you… If, if I think back to my medical school days, or as a very junior, uh, healthcare worker and non-specialist, I would say that, um, from talking about from the student perspective, it’s that I, I don’t think, uh, as a student, uh, there was much emphasis, uh, on, uh, or, or, or sort of, you know, um, teaching on cognitive, uh, biases and, and errors.

00:07:32

And because? I think as medical students, the concentration is really on, uh, learning about classical presentation and, and, uh, you know, the, the standard textbook, uh, descriptors of, uh, diseases and, and disease processes, lah.

So, I think as a student, I wasn’t, uh, very well prepared, yeah. But having moved on to, uh, junior doctor, I think that’s where, where you’re exposed to clinical practices, yeah? And when errors are, are made and errors are discussed at M&M rounds, and so on and so forth.

I think that is the first sort of, like, intro. True introduction into, you know, uh, of biases and acknowledgement of biases. Over.

MO1 Thank you, Participant 23. Would anybody else like to speak next?

P24 Uh, maybe I will go next?

MO1 Oh, yeah. Yes, 24.

P24 Thank you. Um, I, I think as a medical student, uh, I agree with the previous participant. Uh, the focus is to, uh, gain knowledge and to understand disease condition and how they manifest, lah.

So, I think the teacher, uh, is quite important because, uh, the teacher may tell us about certain disease conditions, and, and for that moment, we feel that this is the most important condition in the room. Um, and, and thus, I think we are perhaps then biased for the next few cases we clump, that we are looking out for this particular condition, then.

00:09:09

So, uh, you know, in… I, I don’t think it is very well taught, that, uh, about this matter of biasness and how we can avoid it as a student. Uh, so I believe more can be done, uh, particularly maybe if…

And I’m already thinking of solutions, lah, [laughs]. And particularly if, let’s say, uh, the teacher can also include a bit of information about epidemiology, lah, and how often this thing happened, you know?

MO1 Mm.

P24 Versus, uh, yes, you need to know this. And, um, but then again, this might be a rare, uh, you know, peacock. And this is not a sparrow, you know? [Laughs]. Not something you see very often, yeah. Over.

MO1 Yes, Participant 25?

P25 Yes. Um, I feel that it is related to earlier, how I, not defined, but said what cognitive error is in healthcare practice, eh? Not, not amongst the patients.

Um, and as medical students, all right, we are not totally aware of the environment and the gravity of the environment in which we are operating. Okay? And therefore, uh, we tend to reproduce, or our, our thinking comes more from a textbook perspective.

00:10:33

I think there’s a difference between cognitive errors, error, and bias, yeah? Because bias is formed as a result of previous experiences and attitudes, and it is very difficult, or it’s more, much more difficult to address and correct.

While cognitive errors can be addressed easier, uh, through education, if the existence of cognitive errors, the awareness of it can be, uh, made known to us, how to address, uh, the presence of cognitive errors and how we can overcome these, if these can be taught to us.

And also, when we are taught clinical medicine, how do we, therefore, understand the setting in which clinical scenarios occur? If that can be taught better, then the likelihood of cognitive errors occurring, as medical students, I think will be much less.

I agree with, uh, the two earlier speakers, that in medical school, or at least in our time, we were not taught about this particular aspect of medical practice and of understanding, um, the errors that we can be prone to.

MO1 Thank you. So, um, I’d like to ask a follow-up question about this. Um, so how may…?

How does, uh, medical school or training, or how may it, uh, inculcate habits that make us prone to cognitive errors? How may medical school training, uh, inculcate habits that make us prone to any errors? Or the experience of the training.

00:12:33

P24 Um, is it okay if I go? Participant 24?

MO1 Sure. Yes, yes, definitely. Yes, please.

P24 Uh, I think, uh, as a follow up to what I mentioned earlier, um, I think perhaps medical teaching, medical teachers, clinical teachers, can throw a broader scope of differential diagnosis, uh, which perhaps sometimes is not, uh, within their domain of specialty or practice.

I’d say that, for example, uh, a, a surgeon who speaks about headaches may, may speak very differently from a medical, uh, medicine, uh, specialty, uh, about headaches. Right?

And, and I think, uh, if the, if both sides are willing to, you know, uh, provide a greater, uh, understanding or scope of the differentials of headache, then I think the medical student is better off, lah.

Of course, there are general practitioners and, and, and emergency physicians who kind of help to tie it all together, right? Uh, but I think the, um, I think we ourselves as teachers have, have to be very careful, uh, when we are also teaching, lah, uh, to know that we do have some, uh, biases ourselves, just by the mere fact of the area of practice we are in, and our environment, lah.

So, so I think just to have that open-mindedness, even as we teach, is, uh, is probably useful. Over.

00:14:11

MO1 Thank you, Participant 24. Does anybody else have, uh, anything to add about, uh, exposure to medical school, medical posting, inculcating habits?

P25 Uh, can I? Can, can, can?

MO1 Yeah?

P25 Uh, Participant 25 here.

MO1 Yes?

P25 Um, when, when we are taught clinical medicine and even preclinical medicine, uh, one way, uh, that the, the universities or the medical school, uh, can try to approach, uh, to decrease the tendency for, for us to develop, uh, cognitive errors, is to, um, is to, is to, uh, give us not, uh, the latest.

I mean, when they teach us basics, very often in clinical practice the scenarios that are given expects us to know, uh, the use of the most advanced technology in clinical diagnosis and treatment. You see? Uh, to reach a specific diagnosis that the tutor wants us to work towards.

Uh, and, and, and I would agree, uh, with the, with the earlier speaker, that, uh, tutors should allow for us to achieve a broader perspective on differential diagnosis.

Specialists who usually teach us tend to be, they are specialty oriented, uh, and do not use the breadth of medicine that they should be having, to teach us a basic approach to a particular problem.

00:15:58

And that contributes to some of the issues we see even amongst our House Officers, all right? Who are not able to appreciate the full scope of differential diagnosis, when they can’t consider when they’re faced with a patient with a particular complaint or with a particular set of complaints.

So, the setting in which we are taught clinical medicine has to be broad, uh, and, and if, if it is not provided that way, that tends to more narrow our thinking towards the area, the, the, the kind of discipline that we are working in, or the subject that we are being, being taught, when, when, you know, medicine is, can be very broad. All right? And complaints don’t have to come from one particular type of discipline.

MO1 Okay, thank you, 25.

P23 Uh, 23 here. Um, I think I, I would have benefited a bit more if I was taught to think more in a Systems Two perspective right from the beginning.

Uh, and, and, uh, and the clinical teacher shouldn’t, uh, try and push the student into a System One sort of thinking perspective.

Then, in the, in the sense that, you know, like, I agree with what 25 and, and 24 has mentioned.

00:17:27

That when one, someone is starting out, they should maintain a very wide perspective of a clinical presentation and complaint, so that they do not, um, prematurely sort of, uh, discard, uh, you know, certain, uh, certain diseases or certain disease processes.

And, and I think that is a good way of starting out, so that they’ll be less, I guess, chance of, you know, committing errors. Over.

MO1 Um, thank you. Participant 23, you mentioned System One and System Two. Maybe you could explain a bit more about what you understand by System or, and System Two? For everybody’s benefit.

P23 Uh, uh, 23 here. 23 here. My, my, my, my very, very, uh, very, very generic understanding is that, um, Systems One, it tends to be more, uh, intuitive, uh, you know? Uh, uh, uh, more sort of, like, you know, because of, uh, prior, many, many prior experiences, uh, a dove looks like a dove. No? You know? So, so it’s very, very instinctive. Uh, very, very reactive.

Uh, while Systems Two tend to, someone will tend to analyse a little bit more, need more information, uh, dissects and analyses the, the information more to arrive at a certain conclusion. So, it’s not, uh, so, so not so instinctive, not so reactive. It’s probably a bit more time consuming. Uh, but however, it is a, a, a learned and, and studied, well-studied sort of, uh, analysis of the problem. Over. I hope I’m right.

MO1 [Laughs]. Okay, thank you, uh, Participant 23. Okay, um, all right. So, um, let me just move, uh, a little bit further.

00:19:17

So, now the medical student, uh, becomes, uh, a junior, uh, uh, House Officer or a junior Medical Officer. How do you think, you know, he may have learnt, you know, um…? Or how, how do you think that he…? What would have led him to, uh, commit, uh, cognitive errors and cognitive biases in his practice of medicine, um, as a junior doctor?

P23 Uh, 23. Um, I, I will just fire the first salvo if I, if I may.

MO1 Yes.

P23 Uh, I, I think the, the, the junior doctor, while they are working in a team, I think the senior, more senior colleagues or the more experienced colleagues, actually have a, a very big role to play, I think. If, uh, if they, if they sense or sees a senior colleague, uh, you know, um, uh, acting and, and, and, and, and presenting themselves in a certain way, right? They will tend to follow because they model after them, right? It’s like a model. A modelling art, in fact.

So, so I can imagine if a senior colleague is, um, I won’t call it slipshod, but, you know, um, uh, just, you know, uh, just, uh, very effortlessly, you know, arrive at a, a certain common diagnosis and never entertain other, you know, other, um, uh, uh, uh, other possibilities and other perspectives of, of a patient, lah, you know, apart from just the medical diagnosis. You know?

00:20:53

Doesn’t entertain the social aspect. Doesn’t entertain the financial aspect. Doesn’t entertain, uh, other things. Then the junior doctor would invariably learn, and they would learn very fast because they model after the actions of the senior. Over.

MO1 Thank you.

P25 Um, Patient 25. Uh, Participant 25. [Laughs].

MO1 25, yes. Yeah. Can you speak a bit, uh, louder? Because we can’t hear you so well.

P25 Sure, okay. Um, uh, let’s take the example of a PGY1 that is a HO. Now, a PGY1, usually they have, they are moving into a new environment and, for the first time, they are assuming the responsibility.

And up till the end of medical school, while they may observe, they may track patients, they don’t take personal responsibility for any of those, those patients. And therefore, they live in a very different environment.

They’re, they’re changing their environment very quickly, and they find that environment threatening, very often. They do not know what their seniors are like. Should they only talk when spoken to? Should they only reply when asked questions? And should they better keep quiet?

And when working in that environment, it is a scary environment. You see? Uh, uh, I found it very scary when I was HO, initially. All right? And, and when they’re speaking, uh, working, working in that environment, it tends to restrict their thought process because they just want to get over with it, as quickly possible.

00:22:29

And then, I think it affects their cognitive thinking, because they want to give the first straight answer, not think too much.

And very often they are asked, nowadays especially, well, okay, what would be the differential diagnosis? And suddenly, they get stumped and they hum-hah. All right? Huh? And, and then, and, and that’s when they begin to get into trouble. So, this is where, uh, uh, I feel that, uh, uh, medical students need to be blended into clinical care in a much more gradual manner.

So, today, they have introduced SIP in the final year, uh, but it’s not universal across all disciplines. And therefore, for example, in the discipline of, of emergency medicine, uh, while we want, uh, our students to track as many patients as possible, but they don’t have to take individual responsibility for the patient. You know?

And, and one reason why they don’t do that is, we don’t give them free access to our clinical notes. They’re not a full member of the team. They’re by the way. They’re just observers.

And since there’s no feeling of ownership, uh, and of responsibility, uh, they don’t learn the clinical environment as medical students.

00:23:52

And therefore, the cultural shock for them when they become, it shows, or when they first come to a department like the Emergency Department, unless they have worked well enough in, in the wards and in other areas.

MO1 Thank you. Yes?

P24 Uh, Participant Number Four, yeah. I, I think the previous speaker, I fully agree, uh, because the onboarding and the immersion factors are, are very important, lah.

Um, I, I’d like to share an, uh, uh, uh, a somewhat separate point, is the bit about their work. Uh, the amount of work volume, you know? The, the, the factors that relates to the amount of time that they have to spend with each patient. And also, sometimes, uh, factors like how many hours they work, and how tired or fresh they are, uh, and so on.

So, because I, I think to be able to think well, uh, especially if you want to think well in the type two manner which, uh, the other speaker, uh, mentioned earlier, is that, uh, you, you really need to give them time to pause and then to perhaps reflect a bit and, and, and, and give the answers.

So, um, this, this factor is quite important I feel, uh, you know? That something that is, uh, maybe a bit lacking or not so much addressed, such that, you know, the junior doctors may then potentially make mistakes, lah, by not, uh, you know, having that, uh, uh, cognitive, uh, lightening, lah, in order to think better. Yeah. Over.

00:25:45

MO1 Thank you, um, everybody. Uh, so, uh, let’s, we’ll move on to the next question, if none of you, if none of you have any, uh, other further comments. Okay.

But we can always get back to you later, uh, if you want to say something, uh, related to, uh, what we have spoken earlier, uh, in case you have missed out something. So, now we come to the specialists, um, uh, in the department, in emergency medicine. Why do you think specialists in emergency medicine commit cognitive errors?

P25 Um, Participant Number 25. Perhaps do you want to give us an, an, an example of a type of cognitive error that…

MO1 Okay.

P25 Uh, an emergency physician would, would commit?

MO1 Okay.

P25 So that our discussion can be more focused?

MO1 Um, okay. Uh, actually we would like… Uh, or maybe, um, are you, uh…? Or maybe I’ll ask the rest, because I try not to be too directed. Uh, maybe then, uh, all of you could think of, uh, you know, during M&Ms or whatever, uh, or any incidents that you, as previous HODs, may have, uh, been aware of?

00:27:09

Um, if, you know, when a, a physician, you know, an emergency physician may have committed a cognitive error, like, what sort of errors do you think, uh, they may be?

P23 Uh...

MO1 Participant 23, yes.

P23 Uh, 23. May I?

MO1 Yes, yes.

P23 May I? Uh, um, um, I think one, one of the, um, one of the variables that, uh, that may, uh, make us more vulnerable, uh, could be, um, sometimes, um, uh, uh, what we call, um, when there are too many things happening at the same time, and sometimes, uh, too many, too many of the same kind happening [laughs] at the same time.

So, what, what I, what I mean by that, is that, for example, you know, uh, when you see, uh, you know, on the Saturday night, you know, four or five drunks who has come in, you know? And, and all of them are just basically drunk.

And, and you may treat all five of them the same way because you, you, you are really sort of thinking that, uh, all five of them are just, you know, having a good time while you are working very hard, you know, and all that. And, and I think these are just certain, uh, ingredients I know for, for error to, to brew around, lah, you know?

00:28:41

So, so one of, one of these, uh, variables are things like, you know, um, when you’re seeing, um, many of the same kinds. Many, many.

Uh, you know, like for example, a dime a dozen things, lah, where you, you tend to then, uh, treat them all in the same manner, and you’ll find that they are not the same thing. Over.

MO1 Okay. So, okay.

So, you’re mentioning somebody who may be, a couple of people who may be intoxicated, coming to the department and then maybe one of them may have something beyond just being intoxicated, but that was missed? Uh, are you, like, sort of…?

P23 Yes, yes. Very much so, yes. Yes, very much so.

MO1 Yes, okay. Yeah, thank you. Yeah. Mm, thank you. Uh, Participant 24?

P24 Um, yeah.

So, I, I want to share about the, because I think when we hear M&M, often the mistakes happen, uh, during the care-transition kind of phases and when, uh, patients are handed over, lah. Like, handoffs kind of situation.

00:29:51

I, I think the system is, is lacking with regards to supporting, uh, handoffs, because I, I, I think just to give an example, uh, we, we may hand over patients properly when, when they are boxed and cohorted in certain care areas like, you know, CCA. And then they’re physically in front of us, and we may hand over care plans and, and work out plans quite well.

However, there are also many other patients which we, uh, you know, provide a supervisorial kind of input with regards to their workout plan, that we don’t hand over very well [laughs]. And partly because maybe there’s no documentation means, or it is just too cumbersome, too time consuming to do it.

Therefore, um, we rely on memory. Like, you know, I may tell my colleague, oh, uh, there is this guy that I’m scanning, uh, belongs to so and so, and the reason why I do so is X, Y, Z.

Uh, but there are still the other, uh, [laughs] as the other participant mentioned, the other four to five persons which I may not really hand over the, the workout plan. And often that’s where things sometimes may fall through the cracks, because we don’t communicate well the, uh, intentions or the reasons, or perhaps the gestalt that led us to conduct certain advanced testing or imaging.

Um, so, so I, I think that whether the system can be improved, uh, and, and perhaps more can be do to address or help us to hand over better, lah, yeah. Uh, over.

00:31:38

MO1 Mm-hmm.

P25 Yes, um…

MO1 So, do you think, uh, Participant 24, that this is actually a cognitive error? Or it may be a system error which may not be cognitive?

P24 I, I think it is, uh, end, end of the day, I, I’m more, uh, focused on outcomes, lah. Right? And, and certainly system error is, is there. I mean, the, uh, I mentioned that it is a system that perhaps can help us.

But cognitive also in the sense that, uh, as a special, as a supervising clinician, I, I will have to make that mental effort, lah, to, to perhaps highlight, uh, certain cases, especially those in transition that I, I need to hand over.

So, not just focused on the physical in front [laughs], but more and more so, we are doing remote and telehealth. We need to be perhaps having a separate ledger of patients, uh, uh, to hand over, lah. So, it is still somewhat, uh, there’s still some physician responsibility to take note of this type of handovers, and to do it well, yeah. Over.

MO1 Thank you, Participant 24.

P25 Yeah. How about, uh, Participant 25? May I say something?

MO1 Yes, yes. Go on, please.

00:32:58

P25 Right, thanks. Um, I mean, cognitive errors amongst emergency physicians, what, what I’ve noticed over the years, they occur because we have, uh, we have some biases. All right? Uh? And certain set modes of, of thinking.

Um, and if a patient were to come in, all right, uh, who belongs to a particular set of preconditions, we tend to judge the patient within our preconceived notion of what should be the conditions, or what is wrong with the patient, based on that, those preconceived notions.

And, and that’s when we begin to make mistakes, and I call that a cognitive error. While if we were to look at every patient from a much more broader perspective, we’ll not have any of these preconceived notions, uh, of the type of conditions that particular patient could be suffering from.

And I’ll use the example of what Participant 23 gave, as a person who may have consumed alcohol. May have, all right? Not knowing whether he has or, or, or how much, uh, uh, he or she may have consumed.

Uh, I would, I would presume that they would fit into the realm of alcoholic intoxication, while the person may have, by the way, consumed alcohol, but the person’s also prone to all the full range of illnesses for which that particular clinical presentation, all right, could be part of.

And therefore, I think we tend to very quickly narrow or, or put, uh, patients into certain siloes. Uh, and, and, and that kind of siloed thinking is something which should be addressed in our training, even in residency training.

00:35:12

If we don’t do that, we call it Focused Assessment, but I’m afraid sometimes we tend to overdo that bit of focused thinking, in that we tend not to think of other possible likely causes, or how seriously do we take that particular patient’s complaints.

MO1 So, thank you.

P25 Yeah.

MO1 So, how do you, I mean, um…?

P23 Uh…

MO1 Yes, go on, 23.

P23 Sorry. 23.

MO1 Yes.

P23 Uh, may, may I just, um, continue on from where 24 have left off?

MO1 Yes, yes, yes.

P23 I, I agree. I agree that, um, that, um, handovers is a systemic, uh, you know, process problem.

00:35:54

But I would, I would like to, I would also like to add that, that actually, uh, during handover, which is, I think everybody agrees is the most, uh, dangerous time of the entire care process, is that, um, um, um, handing over a patient, um, uh, often doesn’t give the recipient the, um, the, um, um, the nudge or the push to change a label, even though new information may be available.

So, I think the, the labelling of a patient during handovers, um, by itself actually may, may introduce, uh, some form of, uh, biases and all that. Because obviously, you know, when you hand over, it’s because the information is not complete. That’s the reason why you hand over [laughs].

And, and oftentimes, uh, you know, uh, when the new information does become available, uh, you know, there’s sometimes no, uh, real, um, motivation or, or even incentive to change the label, for various reasons, lah. You know?

Of, of course if it’s very, very stuck, of course there’s then, you know, [laughs] it’s, it’s pretty obvious. But, but if for the, for the, uh, less, uh, you know, for the less stuck sort of, uh, yeah, information, lah, that, you know, they, they may… There may be biases, especially during the handover, and I agree with, uh, Participant 24. Yeah, over.

MO1 Thank you. So, coming back to the, um, original question of, uh, emergency physicians committing, uh, cognitive, uh, errors and why they do so. To what degree or to what extent do you think, you know, medical school training and the, and the process of going through postings and all, may have contributed to this person, um, you know, being, uh, uh, likely, uh, being more likely to commit cognitive errors?

00:37:53

Or do you think it’s entirely separate and, you know, just picked it up much, much later in life?

P25 Participant 25, uh.

MO1 Yes.

P25 Um, while I feel that medical school could have played some part, uh, in the way we think, but at the same time, uh, I think that we should not use that as an excuse for cognitive errors that may occur amongst emergency physicians.

And with, as part of residency training, uh, we should work towards ensuring that our, uh, soon-to-be emergency physicians approach every patient from a broader perspective, able to have an open, able to have an open mind regarding, uh, the likely cause of a patient’s presentation.

And also, in managing the patient, this is treatment of patient, they must also, uh, they must be, uh, able to appreciate various perspectives of the patient, not just the disease condition per se.

But where’s the patient going to go to after the disease? Is he going to go to the ward? Is he going to go to back home? What kind of environment? You know?

00:39:16

And, and be able to appreciate the patient holistically. And, and, and that’s important.

However, there are systemic factors that make it difficult for us, and we have to, uh, understand the role of systemic factors. Crowding is one major systemic factor. We don’t do our handovers very well, uh, when at, at the time of handover.

And, uh, uh, what Participant 24 mentioned about handovers, I agree, is that area prone to error. So, I think it is not necessarily cognitive, it’s more, uh, systems, uh, issue.

Uh, but, but at the same time, I think we need to think or we need to train our emergency physicians, and we have five years to get that done. And we need to train them to be able to approach every patient from a broad perspective, and have a very open mind about every patient’s presentation.

If we can repeatedly do that, then I’m sure we will be able to influence the way our emergency physicians think. And even if there have been limitations in the way training is conducted in medical school, I believe our emergency medicine residents will be able to overcome those limitations and move to a higher plane where, uh, they will take a much more holistic approach. Not just diagnosis, but also management of the patient.

MO1 Thank you.

00:40:50

P25 Over.

MO1 Yeah. So, co-facilitator, you want to ask a question, uh, or, or say something?

MO2 Um, yeah. Thank you very much, uh, Participant 24. Uh, uh, sorry, Participant 25. Um, I think earlier on, you mentioned something.

Uh, you mentioned that, you know, because nowadays with advancements in technology, everybody gets a CT, um, a lot earlier. And then that, you know, disrupts all this, uh, cognitive reasoning and cognitive, uh, thinking, because we are increasingly relying on, um, um, you know, imaging to, to, to, to, to bail us out.

But would you say that…? I mean, I’m just, uh, playing devil’s advocate, but do you, would you say that because of all this imaging, we actually, um, um, make less cognitive errors? Because usually, I mean, in the past, you know, we followed a very textbook, um, approach to things. Um, you know, RIF pain, then we do imaging.

But now, because people are doing imaging more liberally, so, um, um, you may not, we may not pick up, uh, uh, uh, some very large haemorrhage, for instance. But the CT may instantly find a tumour instead, which could be just as important to find out, um, and just as important to diagnose.

P25 Uh, well, no. I think we should not be practising accidental medicine, uh, but it should be thinking medicine.

00:42:27

Uh, and when to practise thinking medicine, then we must, uh, address this issue of cognitive error. You know, when we do more of, uh, imagings and all these things, we learn to… We, we can pick up more anatomical abnormalities, but they’re not very good for functional evaluation of the patient.

And functional evaluation of the patient requires clinical skills, basic clinical skills such as good history-taking. Uh, not just the clinical history. It includes their social and other histories. And also, careful systematic physical examination, which today has deteriorated in a major way, all right? In many areas. People take shortcuts.

A lot of the doctors don’t know how to do it, or are not confident enough doing colonoscopies. Uh, they’ll cover patients through the, uh, putting their stethoscope over the clothes. I’ve seen some who have missed, uh, fine wheezes suggestive of severe bronchospasm.

Uh, so, so these things do occur. So, we have to see, which is more important? Incidental medicine, or thinking medicine? And to practise thinking medicine, all right, which is then therefore less prone to cognitive error, then they have to be, uh, uh, adopt a very systematic approach, a scientific, systematic approach to, to medical thinking and medical practice.

And, and I feel that is what, what is happening nowadays, is that, is that we are increasingly failing in that particular respect. Um, to give you an example, I was teaching medical students, um, two medical students a few years ago at, at NUS, on a particular topic.

00:44:21

The scenario that I was given, um, was working up a patient who presented with high blood pressure, and immediately they were looking for an, an adrenal tumour and all those things at a very early stage.

And I said, why? What makes you think this is a pheochromocytoma that is the cause of the hypertension? And it’s because the case writer was wanting them to, to diagnose pheochromocytoma. Yet, I think medical students or even House Officers should not be diagnosing pheochromocytoma at first presentation. It’s wrong. You see, they should be able to work things out systematically.

And, and, and, and, and I, I asked. Is this what medical school is teaching, that you go for the, the most expensive tests, uh, without thinking of what could the other causes, what is the importance of physical examination?

Do you not want to see whether you can bail out the kidneys? Do you not want to look, listen for a renal bruit? Listen for a systolic murmur? To think of co-optation? Check the pulses? All those things. Because, you see, that is much more basic and you can get the answers at, at much more lower cost, all right? If you practise good clinical medicine.

So, willing to, our willingness to accept, uh, the, the, the, the, the lack of cognitive thinking is leading to very expensive medicine.

00:45:49

Uh, and, and I believe that if we continue in, in that, uh, in, in that mode, uh, medical care will suffer and, and doctors will, will end up getting a bad name. More mistakes will be made, uh, and, and we’ll be, uh, poorer as far as medical skills is concerned.

Because one day, the machines are going to fail us, and when the machines fail us, what is our backup? We don’t have those good medical skills anymore. We have lost it. So, as a good foundation, all of us should have a good, very strong base of good clinical medicine.

MO1 So, Participant 25, as a follow up to your, what you have just said, um, so about this case, about the medical students diagnosing pheochromocytoma right off the bat from, uh, a history of, of a patient with hypertension. So, what…? You know, do you think that medical school also somewhat contributes to, um, uh, uh, aiding and abetting, um, physicians becoming, having cognitive errors?

P25 Yes. Naturally, it does. Um, but that’s where we have to address this, during specialist training. And during specialist training, one of the basic tenets when a person comes into the specialist training is, we need to tell our residents that they must be role models for their discipline. And being a role model, they’ll have to demonstrate that, that, that very strong clinical base without depending so much on technology.

And whatever they may have learnt in medical school through all those expensive tests and Wi-Fi tech, technology, well, it’s, it’s good to have, uh, but more important than that, all right, you must use the initial period of residency training to polish up clinical skills.

00:47:53

And therefore, the initial assessments, formative assessments, all those things in residency training should be geared towards producing, ensuring that our residents are top-notch, uh, as far as basic clinical medicine is concerned.

See? And, and then, by the way, they’ll learn all the other things that specialists learn, but, but the basics must be in place and they must be very good, excellent basic physicians. Otherwise, they forget the basics and only, only learn the, the fine detail.

MO1 Okay, thank you to Participant 25. I, I just want to get back to 24 and 23, whether if you have anything to say about, uh, you know, uh, how ED physicians, um, and, um, their journey in medical school and training, you know, may have contributed to them, uh, um, becoming more prone to cognitive errors. 24?

P24 Yeah. Uh, 24. I, I agree with the Participant 25, that truly, um, basic clinical skills are most important and we can’t overemphasise it more, lah. You know? Uh, particularly when there is such a large, uh, barrage, uh, of investigative matters that is present.

For example, biochemical markers for inflammation, just as an example. You know?

00:49:21

Seeing, uh, junior doctors, uh, make orders for procalcitonin or lactate, which many a times may not be immediately necessary for us to make diagnosis of sepsis or even septic shock. You know?

So, I, I think, um, this type of education is important, so that we can overcome, lah, the challenges that we face in our work environment, when we are also thrown with, uh, guidelines, protocols and other sets, and, and help the, um…

I mean, with a strong foundation, I think the doctor will be better, uh, uh, equipped, lah, to manage, uh, all this different work environment kind of systems, lah. Because they can then put the evidence-based practice, uh, into their decision-making, and also be confident with their bedside findings, lah, to make sense of, uh, not just the, the care plan, but, uh, also to make sense of the way they will work out the patients.

And, and I think later on, as specialists, uh, we are responsible for putting such systems in place, you know? Like, uh, guidelines [laughs], protocols and other things. And, and we have to be very careful to communicate what we are trying to do with this, because it often takes away the thinking.

And we might hide behind a veil of, uh, you know, uh, of, of litigation, lah, or protection from litigation, just by following everything that is being recommended or outlined in those, uh, documents. But then again, we have to ask ourselves, [laughs] is this good medicine or not? And often the answer is, is unnecessary wastage, uh, and, and tests, and, and again, not, uh, patient-centric, lah, you know [laughs]?

00:51:26

Because we are trying to deliver patient-centric care, and here we are also perhaps, uh, uh, aggravating the situation, lah, yeah.

So, so I, I think, uh, I’m not saying that, uh, it is, it is, these systems are not, uh, important, but I think the thinking when we construct them should be, uh, careful, evidence based, and also maybe to communicate well, lah. Over.

MO1 Okay, thank you, Participant 24. 23, do you have anything?

P23 Oh, yeah. 23. Uh, 23 here. Um, I, I think 24 has, uh, uh, framed it very well, lah. Uh, yeah, the, the way I would put it, is that, um, actually a lot of, um, modern-day, uh, algorithmic, uh, protocolised, uh, medicine, right? Is, is founded actually on, uh, on, uh, you know, uh, on, on very specific cohorts, all right, of, of, of, of, of study participants.

You know, often, um, you know, it, it, it doesn’t really generalise, uh, very often, you know? You know, you can, you can go to MD Calculator there and look at all the myriad of, of scores, lah, for every single, you know, complaint you, uh, or single clinical, uh, presentation.

And I think the young, the young doctors are all very fixated on, you know, coming out with a score, and then finding out the risks of the, of this outcome. And then, after that, you know, address it.

00:52:59

But, but I, I think to the older clinician, lah, it’s actually about, uh, narrative medicine, where, yes, I, I do agree that evidence based, uh, you know, uh, if well practised into, uh, guidelines and protocols, obviously serves as the foundation, lah, you know?

But, but really, the practice of medicine is not about algorithms and, and recipe or cookbook medicine, but actually it’s about, uh, you know, it’s about understanding the narrative of the patient and applying the tools, lah, to help the patient.

So, I think, uh, that’s the way I would sum. Uh, uh, I think that could well be a source of biases, no? If you look purely at, uh, at scores and, uh, and algorithms and all that, sometimes our patients may fall through the cracks, lah. I can’t name you specific examples now, but I’m, I’m, I’m sure there are, uh, yeah.

And I, I, and I agree with 25. You know, it’s so much easier to hide behind an algorithm than to, you know, [laughs] than, than to, uh, than to be, um, brave enough to, to analyse it based on the patient’s narrative. Over.

MO1 Okay, uh, thank you for all your insights through sharing. Uh, I think it’s very rich. Uh, co-facilitator, do you have any other questions with regards to, uh, this?

MO2 No, thank you.

MO1 Okay. Um, so how…? So, I just want to further ask, um, perhaps, uh, you know, so when should, um, education in cognitive error, uh, start?

00:54:35

When, when do you think it should start? And, you know, and how should it be, you know, um, taught, actually?

P24 Um, Participant 24. Uh, I, I think it should start early and, um, definitely in the medical, uh, school years.

And the question of how it should be taught is probably, we, I, well, personally I learnt it best during M&M rounds [laughs], because you, you learn from the mistakes of others and, and hope that you don’t commit them.

Uh, so, but I think there may be grounds to share some of these stories with, um, medical students, such that they, they understand that, uh, no, it’s not about finger-pointing or, but it’s to understand, you know, uh, what… Uh, illustrate what biasness or sources of biasness might me, might be, uh, early, uh, to be exposed early in their, in their learning journey, lah. Over.

MO1 Mm, thank you.

P23 Uh, 23. Um, you know, uh, offhand, lah, this is, I mean, the, the topic now is about how, how and when can we introduce this into curriculum, right? So, one, one of… I mean, uh, I, I, I, I was just looking at, you know, uh, our emergency medicine practice. You know, that, that, that section of emergency practice, medical, uh, medicine practice. Uh, um, that, that, that, uh, article that is, you know, that, that is published, CME article that is published every month, right?

00:56:14

The most, actually the most interesting part, lah, is not, uh, [laughs] the front part. You know, the most interesting part is the, the pitfalls part. You know, the risk management part, where they now always highlight ten specific, um, um, uh, sort of like, um, you know, scenario or, or, or often, uh, encountered errors, lah.

And I think, I think the, the education will be so much more, um, richer, if it is balanced by a little bit of introduction into, of, of that, into the, you know, into the curriculum.

So, it’s not to introduce scary medicine, all right? It’s actually to, uh, actually, actually to, to balance things out, lah, you know? And, and, and to, to sort of, um, uh, keep the learners informed, lah, that, uh, uh, that, that, you know, there are risks out there. There are, there are, you know? Yeah. There are different, uh, you know, uh, problems with, with each, um, you know, uh, uh, uh, conclusion and so on. Over.

MO1 Okay, thank you. Um, Participant 25? Uh, you want to maybe, uh…?

P25 Yes. Yes, um…

MO1 Maybe you, you have said it before, but maybe you would like to repeat it, lah?

P25 Okay, because really, I think…

MO1 Like, what it is, lah?

00:57:30

P25 I, I agree with, uh, uh, with Participants 23 and 24, that education in, uh, cognitive errors should start early in medical school, but it should be reinforced during residency training. But even then, uh, just because we reinforce it as much as possible in residency training, it doesn’t mean that because they’ve exited, that’s the end of it. It should continue, uh, even after they’ve, they have exited.

Because, uh, as with many, many things, if you stop teaching something, there’ll be attrition, you may forget.

Um, one of the things I didn’t mention earlier, uh, which I think could also be one aspect of cognitive errors, uh, as you put it, uh, is that very often when we make a diagnosis, all right, uh, we are fixed on the diagnosis and we don’t think again, could it be something else? And we just manage the patient based on the protocol for that particular diagnosis.

And, uh, and, and I think we should, we should, uh, in our, uh cognitive, uh, thinking, uh, we should always teach our residents that just because I’ve made an initial diagnosis, that doesn’t mean that I now move on totally to just managing the patient, because of the diagnosis, lah.

I’m not managing, I’m not treating the diagnosis. I’m still treating the, the patient. So, I must still continue to maintain an, an open mind, that there could be other things, uh, that are present, that could influence the initial diagnosis that I’ve made, that could influence the management of the diagnosis.

n00:59:21

So, so therefore, uh, I think it should be taught at all levels, but it should not end at exit. It is a, a, a thing that we should continue teaching and emphasising during M&M rounds, and even having perhaps once in a while conduct a special teaching session on cognitive errors in emergency medicine. Uh, we should, we should think about it. Thank you.

MO1 Thank you, uh, everyone. Uh, so let me, uh, um, ask some further questions. So, in your opinion, um, is the emergency physician responsible for recognising and overcoming cognitive errors, or is it a system-level error that’s beyond their control, since they have been educated or not been educated adequately [laughs]? May not have been educated.

P25 Sorry, Participant 25 here. Could you repeat that question because there was a knock on my door?

MO1 So, in your opinion, is the emergency physician responsible for recognising and overcoming his own cognitive errors, or is it a system-level error that is beyond their control? Like maybe they were not educated, that may have been a system error. Or maybe the system, um, is so overwhelming or distracting, that it’s beyond his control. Therefore, it’s not his, it’s not his responsibility.

P23 Uh, 23. I think the, uh, the practice of self-reflection is a very important, uh, and crucial, uh, practice, lah, you know, that, that, that everybody, whether junior or senior has to, has to, um, uh, undertake. So, I think, yes, um, we may not be, I mean, we may not have been very well taught systematically in our formative years.

01:01:35

But I think all of us, I would daresay all of us, uh, through, through the passage of time, through, uh, systems, uh, you know, M&Ms, you know, and, and now we call peer-review learning and all that, right, yeah? Yeah. And, and, and, and, and various sort of other, uh, learning opportunities, right? Have actually picked up, you know, uh, where our blind spots and personal failings, uh, could, could, could… Uh, where our personal failings could lie.

So, I think, uh, yes, it, it may not be formalised. Uh, yes, we may not be well taught. But I think, um, everybody who is in, deep in, and, and, and invested in clinical practice, lah, would have obviously, uh, you know, uh, have, uh, you know, invested enough time, you know, uh, going through, you know, their own personal development in, in, uh, annulling or at least minimising, uh, cognitive biases. Over.

MO1 Thank you.

P24 Uh, Participant 24. Um, I, I think as a professional, uh, regardless of which industry, uh, there is always a need, uh, for the practitioner to be reflective, be humble, you know, and be perhaps able to work with others, to listen, to give feedback and to receive feedback properly [laughs]. And, and, and so these are all the basic, uh, tenets of becoming a good professional, lah.

01:03:08

Whatever we do, uh, to be observant and to use, uh, interpret data correctly and so on.

So, I, I think as much as the systems may have affected us to, uh, you know, make mistakes or, or caused us to be, contributed to our biasness, uh, I, I think the first step is to, uh, uh, tackle our internal factors, lah, before talking about the external, uh, factors at work, at the workplace. So, I…

MO1 Can you give us an example of internal factors, or what you, um, what you mean by internal factors?

P24 Um, so I mentioned being reflective. I think the, uh, addressing our own personal wellness, you know, is, is all, uh, important to consider that as somewhat a priority, even as we go about our daily business, um, of, of caring for others.

So, uh, uh, I think internal factors in that sense. Uh, internal factors of, uh, also like how we relate to other people, and having respect and humility, I feel those are characters that we want to develop. And, uh, and also help develop those in others as well, lah [laughs].

Because earlier, we hear about role modelling, right, [laughs] as senior physicians [laughs]? And, and I think, uh, that we have to be careful, yeah, uh, in the workplace as well, because people are often, you know, emulating and watching, uh, how the senior, senior physicians, uh, are working and how they, uh, uh, handle others and speak to others and so on. Yeah.

So, I, I, I think, uh, that might be something I feel quite important as a first step to, uh, address.

01:05:09

And then, uh, you know, and, and, and externally I think those factors sometimes we can influence, sometimes we can’t, lah.

MO1 Okay.

P24 How busy the department is, or how well staffed the department is, yeah. Over.

MO1 Okay, great.

P25 Um, Participant 25, lah. Uh, while I agree that self-reflection’s very important, um, but…

MO1 Uh, sorry, uh, we can’t hear you clearly. Can you speak a bit louder?

P25 I think that that, that’s not important because of…

MO1 Um, sorry, Participant 25, uh, you are quite muffled.

P25 All right.

MO1 Um, yeah, maybe you can speak closer to the speaker.

P25 Okay, I’ll come closer.

MO1 Hello?

01:05:58

P25 Uh, yes, yes. Can you hear me now?

MO1 Yes. Uh, yeah.

P25 Sorry. Um, while I feel that self-reflection is very important, but at the same time, cognitive errors, uh, the person making the cognitive error may not be totally aware that, uh, of the error that he has committed, to make up something that’s going to make up for that.

And therefore, every person, regardless of seniority, should be open to feedback, and feedback should… And that feedback should be non-threatening feedback. And, and, but for a person to be receptive to feedback, humility must become a very important characteristic of, of every physician. Uh, and, and, and that humility must be demonstrated in his daily activities, daily actions, uh, regardless of seniority.

Uh, and because even as Participant 24 mentioned, uh, uh, uh, that, that humility, uh, yeah, is a very important component of a senior physician. Uh, and, and I feel that once there is humility, then there’s less likelihood, uh, uh, for, for, for physicians now to accept feedback.

In the same vein, we must also be prepared to provide feedback to our colleagues, and such provision of feedback should be given in a non-threatening manner, so that the others also learn from, from this kind of feedback. I don’t think we have achieved that kind of motivation where we freely provide feedback, uh, and, and it’s something we need to think through, how we’ll do it, uh, and become comfortable with that.

01:08:17

While self-reflection… Therefore, while self-reflection is important, there must also be other mechanisms, uh, like other feedback mechanisms that can inform the person committing that so-called cognitive error, that, uh, he needs to really think about it.

Uh, and therefore, some form of self-education, uh, and, uh, I mean, self-education process, that’ll then better help that person remediate, so that the likelihood, uh, of that person committing similar cognitive errors would be much less. Yeah, thank you.

MO1 Thank you. So, can I summarise what you’ve just said? So, you feel…?

Do you feel that the emergency physician has some responsibility for recognising and overcoming his cognitive errors?

But at the same time, there’s also some peer responsibility to help one another, uh, recognise, uh, and overcome cognitive errors, uh, through feedback?

P25 Uh, well, I think…

MO1 Is, is that basically roughly what you’ve just said, yeah?

01:09:28

P25 I, I think by definition, if it’s a cognitive error, uh, self-recognition may not be evident initially, and, but, uh, the person, on realising that then this could be a cognitive issue, must then take steps to address it. And that’s where the reflection comes in very important.

And yes, I agree that all of us, uh, will also have to be, uh, prepared to learn, to provide feedback and receive feedback that is non, that is provided in a non-threatening manner.

aMO1 Do you think that…?

P23 Uh, 23.

MO1 Mm, yes, yes.

P23 Sorry, 23. May, may I?

MO1 Yes, yes, yes.

P23 Yeah, uh, okay. I, I, I think I, I, I heard, um, 25, uh… Sorry, no, I heard 24 [laughs] mention about internal, external causes, right? And then also 25 mentioned about, um, about, uh, about, um, uh, the, you know, it cannot just be totally dependent on self-reflection.

I, and I, I, I agree with all those points, um, but I just want to, I just want to revisit, uh, 25’s, um, previous contribution when, when he said about external factors. I, I, I think, um, you know, um, yes, uh, some internal factors, uh, if we have good powers of reflection, we may be, uh, we may be able to master some of these internal factors, right?

01:11:08

But however, I think external factors oftentimes are not within our direct control.

So, I think, uh Participant 25 had mentioned before, things like overcrowding, uh, things like, you know, uh, when, when you don’t have, um, space or privacy to do a DRE, and you don’t do a DRE.

So, is that because you are cognitively biased? No, I don’t think so. I think it’s because the, the, the situation and the external factors do not permit you to exercise your control. All right?

And I, and I want to say that, that ever since, like, the institution has started purchasing our medical indemnity insurance, right, it’s actually an admission then. And, and, and, and I, I, I, I, I, I, and I do, I, I, I do, uh, support them on that, lah, uh, paying for my medical indemnity.

But again, it’s a recognition of the fact that the individual clinician works in an environment that is dictated by the system, and therefore, you know, uh, you know, it’s, it’s a direct recognition to, to a certain extent.

And of course, there are many other reasons for that, but it’s a direct admission that the individual physician, right, uh, work under the conditions of external environment. Um, over. Yeah.

01:12:37

MO1 Okay, um, thank you very much for, um, all, all the discussion about, uh, the responsibilities. Um, so next I’ll go back, go on to the last two questions. So, I would like to ask you all, um, how do you personally, or what strategies do you use to mitigate cognitive errors, personally? Anyone can start.

P25 Can I start first?

MO1 Yeah, yeah. Sure, yeah.

P25 I’m, I’m Participant 25. Um, number one, self-reflection plays a big part, um, and, and, uh, and I think that that is, you can’t deny that. But at the same time, we must realise that there are also gaps in our knowledge, uh, and skills, uh, that sometimes results in us not, um, addressing particular areas which may be important in the management of the patient.

And we must, uh, then work out how we address those gaps in our knowledge and skills. Uh, with knowledge, then we have to work towards some form of self-education or education through some programme. With skills, uh, either learn those skills or ensure that those skills are available when we are, uh, in the departments.

And, and, uh, have the humility to, uh, get our colleagues who may have those skills, to use their special skills, so that, uh, patients’ needs can be addressed adequately. So, so I think that would, would be, that is the approach that, that I take.

01:14:42

MO1 Thank you.

P24 Um, Participant 24. I, I think two things for me. One is, uh, CME, lah, so educating myself continually.

Uh, the other, second thing I find useful is, uh, to be having a healthy dose of, uh, scepticism, lah, uh, with regards to how people tell me about the cases they, they, they hand over to me. And, and having the, uh… Taking a step back, you know, perhaps to, uh, to review the whole case with, with, with, with, uh, some fresh perspective.

Uh, so in other words, I think it’s a bit of a questioning attitude, lah, [laughs] yeah. So, I think these two things. Yeah, thank you.

MO1 Thanks. Participant 23?

P23 Uh, 23. Um, I, I would, I would, I would say that every single time we, uh, go to work, right, we, we have to, um, we have to be, be able to perform at the highest standards. So, how to perform at the highest standard?

Number one is that we have to, of course, you know, get the CME, like what Participant 24 has mentioned. We have to, um, you know, we, we, we have to prepare ourselves and all that. But, but, you know, that, that, that is I think the, the, the coaching part, you know?

01:16:12

Uh, you know, that, that comes right before. You know, the teaching, the learning, the coaching, the mentoring, uh, and all this, that part.

But then, when it comes to, um, the actual, uh, work during the eight hours or nine hours, I think the, the, the performer has to be prepped, lah. Okay? Uh, I mean, I always go back to, you know, high-performing sports, uh, you know, performers. They, they don’t, uh, they don’t just come on time to just go into the arena to play, right?

They, they have a warmup, you know [laughs]? They prepare themselves mentally, psychologically. They prepare themselves physiologically, you know? Well rested, no distraction, you know? No, you know, uh, doing, uh, uh, something else while you are doing your clinical work, and so on and so forth. You know?

You know, I remember when I was in, uh, MGH, ah, as a fellow, right? I remember that there was this ruling, lah, that no faculty can be seen in their offices during their entire clinical shift. Nobody ever dared to go up or down into their offices. Nobody. Nobody dared to do that, okay?

And, and, and it’s, I, I mean, it’s, it’s a reflection of the kind of, um, performer. I mean, the kind of, uh, you know, uh, performance that is required out of, uh, of us, lah, now I would say, yeah?

So, I think, I think, uh, I think Participant 24 has mentioned, lah, about wellness, right, lah?

01:17:43

I think the, the, the wellbeing part, the, the preparedness part, to come to be engaged with patients and, and care of patients, I think is very important as part of the foundation and preparatory work of, to lessen cognitive biases, lah. Yeah, over.

MO1 So, so 23, so what are the…? What, what, what strategies do you use?

P23 Well, this is coming, becoming personal, lah? [Laughs].

MO1 Yeah, yeah, yeah, definitely [laughs].

P23 Okay, okay, yeah. Okay, so, so personally, um, I don’t know whether, I, I don’t know whether you realised, lah, I, I usually come at least an hour before my shift. I will. You’ll never see me coming, uh, ten minutes before my shift. You will never ever see me doing that.

Of course, I mean, you know, if, if, if ever that happens, then it must become some kind of emergency and all that, because I, I, I need to get into the, the, the realm of things, lah. You know? I need to get myself into the realm of things, because I know that…

I, I don’t know about, I don’t know about the rest of you, lah. I, I know that when I come back from a two-week leave, right, my state of mind is different from, like, if I was working yesterday. It’s quite different.

01:18:56

I’m not up to speed, lah. I, I, I know that for sure, yeah, because it is just not quite there, yeah. So, the… I, I, I know, it’s just like, it’s just like playing, it’s just like playing, uh, uh, in the team sports, right?

If you haven’t been practising for [laughs], let’s say, two weeks because you’re out injured, right? You, you know that the next, the first game you come back, you’ll, you’ll be rusty, yeah. I, I, I feel that myself, so, so that’s from my personal sharing and perspective, lah. So, I think individual prep work is very important. Over.

MO1 Thank you. So, Participant 25, um, any personal tips? Or, okay, how about when a new AC comes in, just joins the department, your department, you know, what advice would you give them specifically, so as to help them, you know, um, not, you know, commit, uh, cognitive errors?

P25 It is, it is very difficult to find a formula, uh, for, uh, a new AC coming into the department, uh, because no single formula addresses the challenges that the new AC is going to face.

What we can do is adopt a very general approach, uh, a mother-henning approach in a way, by telling the new AC that this, you’re beginning, it’s a, I mean, a new path, uh, a new phase of your professional career where you’re beginning to take a higher level of responsibility.

And being in that new phase, you will, you will face many challenges. The thing is, you’ll be afraid, but not to let that fear undermine what good you can do for the patient.

01:21:08

And remind that new AC, it’s not the department that is important, it is the patient who is being managed. Every individual patient, they’re the most important, really, in the, the department.

And therefore, when you come in as a new AC, if at any time you feel overwhelmed, or you feel that you’re not sure how to deal with a particular situation, know you are now a specialist registered with the SAB.

The reason we have consultants and senior consultants in the department… And you may know more in terms of knowledge than your consultant or senior consultant. That’s not the point. The, the point is, you’re facing a challenging situation. What are you going to do about it?

And, and that’s when, okay, the need to, to consult a colleague, uh, should come in. Not to be afraid of that. And no one will see you in a bad, in a poor light because you thought you had to consult a colleague as to how to manage a particular situation. So, so I would, uh, I would give that bit of, of advice to a new AC.

MO1 Thank you.

P24 Uh…

MO1 So, the question is… Uh, yeah, yes, 24?

01:22:25

P24 Participant 24. I, I recall one specialist, and I won’t mention names, uh, who told me, hey, now that you are a specialist, lah, you can no longer ask questions. And [laughs] as a junior specialist, I was taken aback and I was very afraid [laughs]. And it’s the total opposite of what Participant 25 just mentioned [laughs].

And I, I think there is, like, potentially that kind of culture in some departments, lah, uh, or maybe specific to certain individuals, lah. Uh, but jokes aside, uh, and that, uh, sorry, it’s not a joke, it’s a truth, uh, it really did happen to, [laughs] to me.

MO1 Hmm.

P24 Uh, I, I, I think for me, uh, uh, the advice I will give is, is, is definitely, as Participant 25 has mentioned also, that, uh, have, have the courage, uh, to, to, to speak out, lah. You know? To speak out and to ask for help. Uh, because, uh, it is a team sport, it is a team sport, and, and by, you know, working together, then we can solve problems better and avoid the errors there. Yeah, over.

MO1 Thank you.

P23 Uh, 23. Um, I, I think, I think, I think I guess, um, uh, many of us may have sort of, you know, um, uh, gone through, uh, or has read, uh, you know, uh, papers that describe various kinds of cognitive, uh, biases and errors and so on and so forth.

So, the question now seems to be, uh, leading towards, is there a need to, uh, develop a, a, a formalised, uh, curriculum for the new AC when they come in? Because since the, the signs is out there, right? Or at least the description of the signs is, is out there. So, the… Uh, right?

01:24:28

So, so the thing is, uh, should there be? Is, is that what you’re asking? Should there be a, should there be a sort of like a formalised, uh, coursework or whatever and all that?

MO1 My question is, if an AC comes to you and asks you for advice.

P23 Ah.

MO1 You know, what advice would you give him or her?

P23 Mm-hmm.

MO1 With regards to, you know, um, avoiding cognitive errors or any error.

P23 Well, I don’t wanna have to spend many hours on that [laughs].

MO1 But in summary, how would you, you know, uh, advise, uh, this person?

P23 I would say, don’t worry, you’ll learn along the way, and hopefully not the hard way, yeah [laughs].

MO1 Okay, all right. Uh, does, uh, anybody else have anything to bring up about cognitive, uh, errors, uh, pertaining to emergency physicians? Um, you can bring up anything. Uh, I mean, outside of the questions that I’ve asked as well.

01:25:32

Or does the co-facilitator have any other thing to ask, or, or did I miss out any questions that I should have asked? Yeah? Okay. Uh, okay, so…

P25 Uh…

MO1 Yes?

P25 I, I remember earlier in the questions, you were talking about the new ACs.

MO1 Uh, sorry, we have trouble, uh, hearing you again.

P25 Oh, okay. Uh, once again, can you hear, uh, hear me now better?

MO1 Yes, better now.

P25 Okay, thank you. Um, I think we’ll need to always ensure that our new ACs, uh, though we may advise them to come and ask as frequently as possible, but they will have their fears. They will say, I’m now moving into a department where I’m amongst the seniors, the lowest of the, of the low, and I should not show my frailties at this very early stage.

So, that in self and everything may hinder this, and therefore there is a need for mentorship of our young ACs. And this must be an active mentoring process, that, uh, and that, that has to continue for quite some time to come.

And if we can, if that is the case, all right, then we’ll have a way to better ensure that our new ACs will be a much better consultant, or senior consultant, when the appropriate time comes.

01:27:11

So, I think mentoring is very important. I’m not sure how well we do it, or how we do it, uh, but, but, but it is important.

And we need to also self-reflect amongst ourselves, that, uh, if I were in the best of environments, how would I mentor my new AC? And try and, and move towards that, that goal of giving good mentorship to our younger colleagues. Thank you.

MO1 Thank you.

P23 Uh, 23. Uh, maybe, maybe I think if you ask me like that, lah, uh, when the AC approaches me, right? I think going forward in the future, all right, in the future, maybe the best thing I can possibly do is that, you know, to tell the AC that, you know, I’m, I’m not perfect and I’m still making the same errors as, as from day one. It’s just that I’m trying to make less of it, lah.

MO1 All right. Um, any other questions or comments? Okay. Uh, okay. So, thank you so much for spending, uh, so much time with us, and we appreciate your, uh, honest sharing, lah. And, uh, yeah, so if there are no further questions, you know, um, yeah, um, so, uh, we may, uh, leave now.

Thank you very much.

MO2 Thank you, everyone.
